# Identification of surface proteins in a clinical *Staphylococcus haemolyticus* isolate by bacterial surface shaving

**DOI:** 10.1186/s12866-020-01778-8

**Published:** 2020-04-07

**Authors:** Runa Wolden, Maria Pain, Roger Karlsson, Anders Karlsson, Elizabeth G. Aarag Fredheim, Jorunn Pauline Cavanagh

**Affiliations:** 1 Pediatric Research group, Department of Clinical Medicine, Faculty of Health Sciences, UiT The Arctic University of Norway, Tromsø, Norway; 2 Nanoxis Consulting AB, Gothenburg, Sweden; 3 Department of Infectious Diseases, Institute of Biomedicine, Sahlgrenska Academy, University of Gothenburg, Gothenburg, Sweden; 4 Department of Clinical Microbiology, Sahlgrenska University Hospital, SE-413 46 Gothenburg, Region Västra Götaland Sweden; 5 Microbial Pharmacology and Population Biology, Department of Pharmacy, Faculty of Health Sciences, UiT The Arctic University of Norway, Tromsø, Norway; 6 Department of Pediatrics, The University Hospital of North Norway, Tromsø, Norway

**Keywords:** *Staphylococcus haemolyticus*_*1*_, Surface protein_2_, Surface shaving_3_, biofilm_4_, adhesion_5_, virulence_6_, keratinocytes_7_, Host-microbe interaction_8_

## Abstract

**Background:**

The skin commensal *Staphylococcus haemolyticus* is an emerging nosocomial pathogen. Despite its clinical relevance, published information about *S. haemolyticus* virulence factors is scarce. In this study, the adhesive and biofilm forming properties of ten clinical and ten commensal *S. haemolyticus* strains were examined using standard adhesion and biofilm assays. One of the clinical strains was used to identify expressed surface proteins using bacterial surface shaving. Protein abundance was examined by a comparative analysis between bacterial protein expression after human keratinocyte (HaCaT) colonization and growth in cell culture media supplemented with serum. Relative protein quantification was performed by labeling peptides with tandem mass tags (TMT) prior to Mass Spectrometry analysis. Surface proteins can be used as novel targets for antimicrobial treatment and in diagnostics.

**Results:**

Adherence to fibronectin, collagen and plastic was low in all tested strains, but with significantly higher adhesion to fibronectin (*p* = 0.041) and collagen (*p* = 0.001) in the commensal strains. There was a trend towards higher degree of biofilm formation in the clinical strains (*p* = 0.059).

By using surface shaving, 325 proteins were detected, of which 65 were classified as surface proteins. Analyses showed that the abundance of nineteen (5.8%) proteins were significantly changed following HaCaT colonization. The bacterial Toll/interleukin-1 like (TIRs) domain containing protein (*p* = 0.04), the transglycosylase SceD (*p* = 0.01), and the bifunctional autolysin Atl (*p* = 0.04) showed a 1.4, 1.6- and 1.5-fold increased abundance. The staphylococcal secretory antigen (SsaA) (*p* = 0.04) was significantly downregulated (− 1.5 fold change) following HaCaT colonization.

Among the 65 surface proteins the elastin binding protein (Ebps), LPXAG and LPXSG domain containing proteins and five LPXTG domain containing proteins were identified; three Sdr-like proteins, the extracellular matrix binding protein Embp and a SasH-like protein.

**Conclusions:**

This study has provided novel knowledge about expression of *S. haemolyticus* surface proteins after direct contact with eukaryotic cells and in media supplemented with serum. We have identified surface proteins and immune evasive proteins previously only functionally described in other staphylococcal species. The identification of expressed proteins after host-microbe interaction offers a tool for the discovery and design of novel targets for antimicrobial treatment.

## Background

*Staphylococcus haemolyticus* is a coagulase-negative staphylococcus (CoNS) and a member of the skin microbiome. It is an increasing cause of nosocomial infections associated with indwelling medical devices, particularly affecting immunocompromised patients and premature babies [1–3]. A distinct characteristic of clinical *S. haemolyticus* strains is the ability to acquire resistance to several classes of antimicrobial agents [2]. The ability to colonize and form biofilms is regarded as the most important virulence trait for CoNS [4]. Adhesion is the first step to form biofilm on surfaces [5] and staphylococci express several adhesive surface molecules that interact with eukaryotic host cell receptors, abiotic surfaces or soluble macromolecules. The number of adhesive surface proteins varies among different staphylococcal species. In *Staphylococcus aureus*, 24 different cell wall anchored proteins have been identified, while CoNS express a smaller number [6]. Cell wall anchored (CWA) proteins are covalently attached to the peptidoglycan layer. The most prevalent CWA proteins are the microbial surface component recognizing adhesive matrix molecule (MSCRAMM) family. All CWA proteins contain an LPXTG motif (Leu-Pro-X-Thr-Gly; where X can be any amino acid) that anchor the protein to the cell wall [6]. The Sdr protein subfamily of MSCRAMMs contains a serine-aspartate repeat region [1, 6] and a signal peptide with an YSIRK motif. In *S. aureus* the majority (13/21) surface proteins harbors the YSIRK/GS signal sequence, allowing delivery of surface proteins to unique locations in the cell wall [7]. *Sdr-*like genes have previously been described in *S. haemolyticus* [8].

Another family of the CWA proteins is the Serine Rich Repeats Proteins family. Like the Sdr proteins, they have a serine repeat region, but with alanine, valine or threonine instead of aspartate [9]. Bacterial surface proteins can act as new targets in treatment and prevention of infections in multiresistant bacteria. One method to examine bacterial surface proteins is by surface shaving. Surface-shaving is a technique where peptides from bacterial surface proteins are cleaved off when protease treatment is applied followed by a Liquid Chromatography tandem Mass Spectrometry (LC-MS/MS) analysis [10]. The Lipid-based Protein Immobilization (LPI™) technology enables surface shaving of intact bacterial cells in a flow cell, and thus promotes detection of proteins expressed in the surface proteome over the highly abundant cytosolic proteins. The flow cell channels, binds intact cells by a passive process. As the surface is similar in each channel, the same number of cells are bound. Thus, combining the surface shaving approach with protocols for relative quantification, such as tandem mass tags (TMT), makes studies of low abundant virulence factors possible [11–17].

**Fig. 1:**
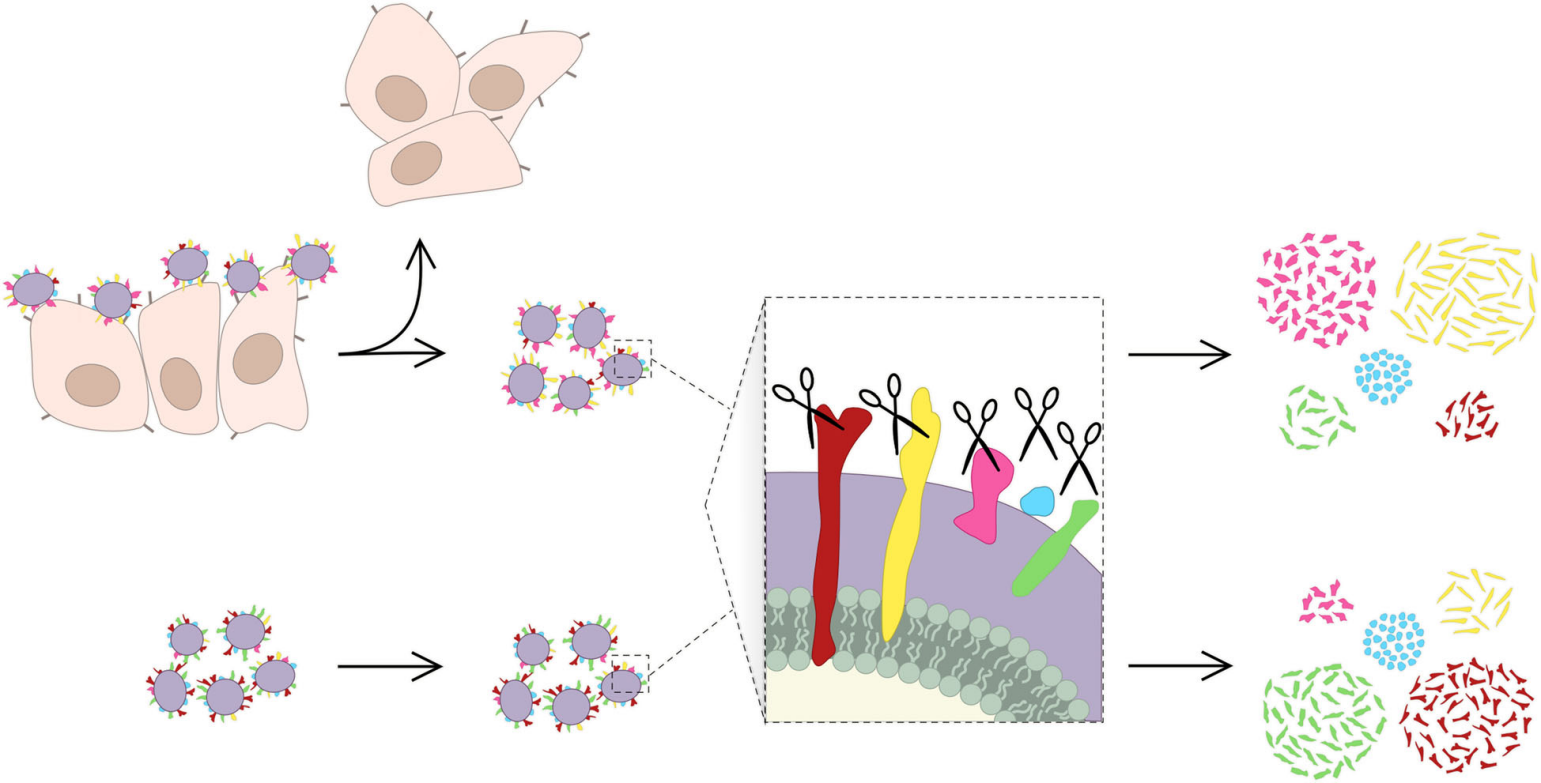
Bacterial surface protein shaving, graphical abstract. Comparison of S. haemolyticus surface protein expression after HaCaT colonization (top) and the control group (bottom). Bacterial surface proteins (multicolored) are degraded by the protease Trypsin (scissors)

Several studies on surface proteins and their relevance in host-pathogen interactions and virulence have been performed after bacterial growth in standard laboratory medium [18–22]. In order to mimic a more biological relevant host-microbe interaction, we developed a novel method to investigate expressed surface proteins of a clinical *S. haemolyticus* isolate after colonization of human keratinocytes (HaCaT) before bacterial surface shaving was performed (Fig. 1). To our knowledge surface protein shaving of bacteria subsequent to colonization of mammalian skin cells has never been described before.

In this study, we aimed to investigate the adhesive and biofilm forming abilities of ten commensal and ten clinical strains. We have previously shown that there are specific genetic signatures associated with clinical *S. haemolyticus* strains compared to commensal strains [23], thus we wanted to investigate if any functional differences in adhesive properties between commensal and clinical isolates could be observed. Furthermore, the expression of surface-associated proteins of one clinical *S. haemolyticus* strain was investigated by mass spectrometry and proteomics. The LPI surface shaving approach and relative quantification proteomics using TMT labels was employed to identify possible novel targets for treatment, prevention and biofilm formation.

## Results

We wanted to examine if commensal and clinical strains had different ability to interact and adhere to selected host proteins. The adhesive ability of ten commensal and ten clinical strains to both uncoated plastic and plastic coated with fibronectin and collagen was examined to determine if binding to fibronectin or collagen would enhance binding to plastic, as we observed that binding to plastic in its native form was generally low. Further the biofilm forming capacity was examined. Eventually, one isolate was selected for bacterial surface shaving.

### Adhesion to plastic and host matrix proteins

**Fig. 2:**
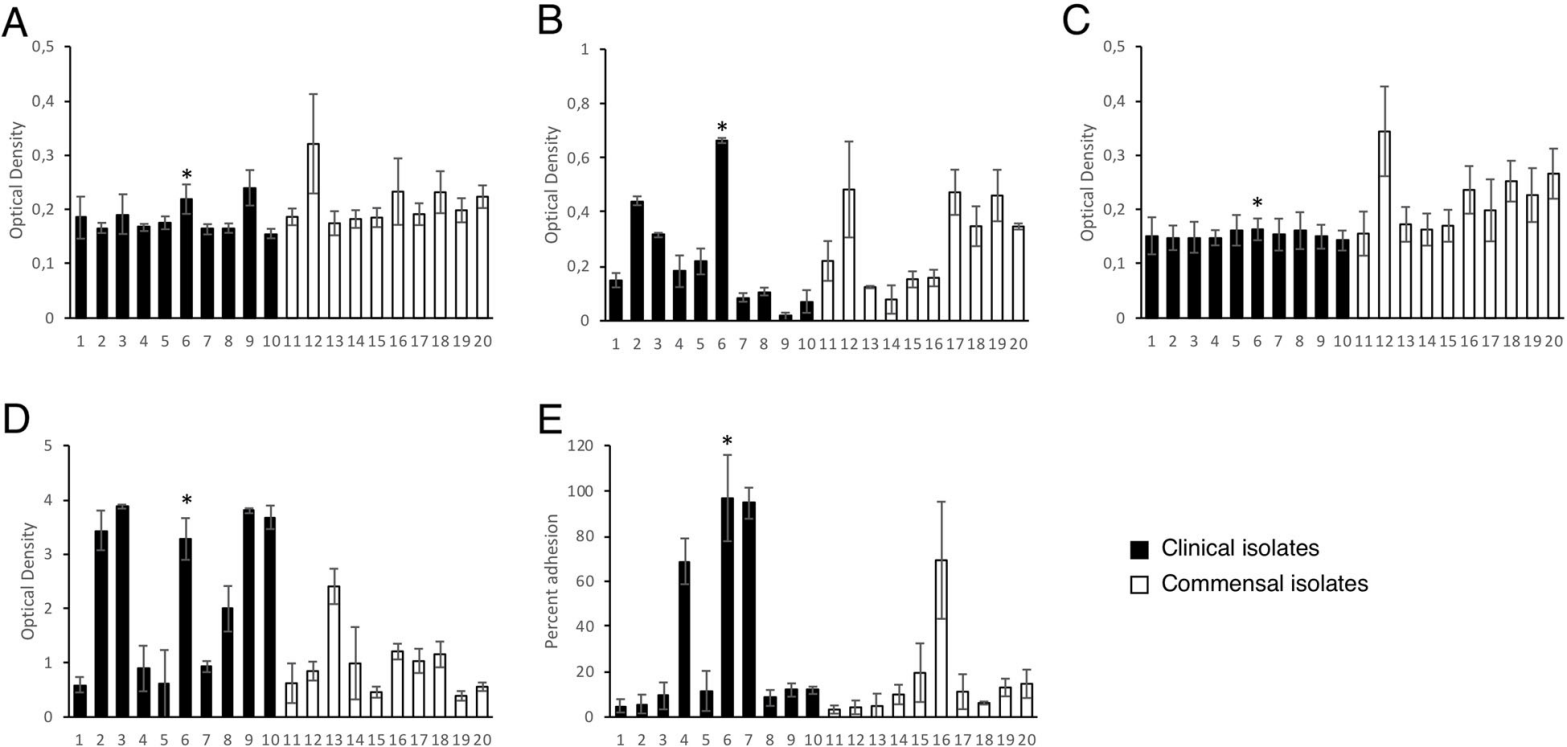
Adhesion and biofilm assays of *S. haemolyticus*. Columns with black bars are clinical isolates and white bars are commensal isolates. Sample no. 6 was chosen for bacterial surface shaving (marked with asterisk). **a-c** Solid phase host matrix binding assay; **a**) Adhesion to fibronectin; **b**) Adhesion to plastic; **c**) Adhesion to collagen; **d**) Semi-quantitative determination of biofilm formation; **e**) Adhesion to human keratinocytes

Both clinical and commensal strains adhered to plastic but no significant difference was observed between the two groups. Fibronectin and collagen binding were low for all strains, but still significantly higher for the commensal strains compared to clinical strains, *p* = 0.041 and *p* = 0.001 respectively (Fig. 2a-c).

### Semi-quantitative determination of biofilm formation

The biofilm-forming ability of the strains was determined using a semi-quantitative assay. All strains formed biofilms and a trend towards higher biofilm formation was observed for the clinical strains (*p* = 0.059) where 5/10 clinical strains formed substantial amounts of biofilm in this assay (OD_570_ > 3) compared to 0/10 commensal strains (Fig. 2d).

### Adhesion to human keratinocytes

The strains were screened for their ability to adhere to human keratinocytes. In three clinical and one commensal strain > 60% of the inoculum adhered to the keratinocytes, while seven strains showed an adhesion of ~ 10–20% of the inoculum, which was in the same range as the *S. aureus* (NCTC 8325–4) control strain (Fig. 2e). On average, the clinical strains adhered better to the keratinocytes compared to the commensal strains, although the findings were not statistically significant (*p* = 0.4). One strain, displaying high adhesion to HaCaT cells in addition to being a strong biofilm producer, was chosen for further analyzes.

### Bacterial surface protein shaving

Expressed surface proteins of a clinical *S. haemolyticus* isolate either colonizing HaCaT cells or grown in cell culture medium supplemented with serum, was examined by surface shaving using a Lipid-based Protein Immobilization flow cell. Relative quantification of protein abundance was performed by labelling proteins with tandem mass tags (protein markers) prior to LC-MS/MS.

#### Protein identification and subcellular localization of *S. haemolyticus* proteins detected by surface shaving

Cell surface shaving of bacteria colonizing HaCaT cells or incubated in cell culture media supplemented with serum resulted in identification of 436 proteins by LC-MS/MS analysis. Only proteins with ≥ #2 peptide-spectrum matches (PSMs) were included for further analysis, resulting in 325 proteins (Supplementary Table 1 and 2).

Subcellular localization analysis of the 325 proteins in silico and functional annotation predicted 249/325 (76.6%) cytoplasmic proteins, 65/325 (20.0%) surface proteins (i.e. proteins predicted to originate from the cytoplasmic membrane, cell wall or extracellular origin), and 11/325 (3.4%) as undefined proteins.

#### Clusters of orthologous groups

**Fig. 3:**
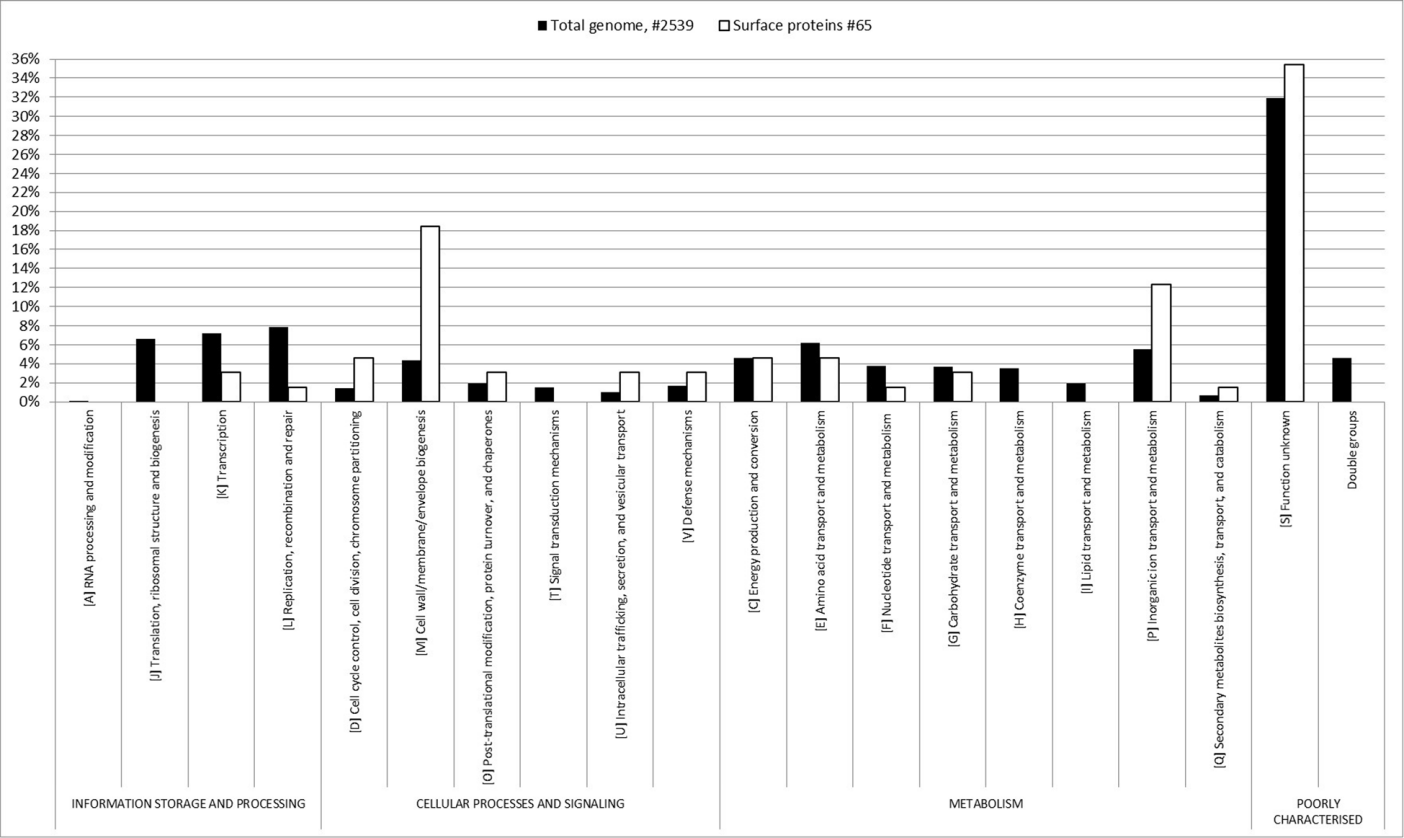
Clusters of Orthologous Groups of proteins (COG). Comparison of Clusters of Orthologous Groups of proteins (COG) between the total proteins of the strain (#2539) and surface proteins (#65) found after HaCaT colonization

The 65 identified surface proteins were distributed in Clusters of Orthologous Groups (COG). A higher percentage of proteins in COG groups M (cell wall/membrane/envelope biogenesis) and P (inorganic ion transport and metabolism) was found when we compared the COG distribution of the identified surface proteins (65) to the COG distribution of the total number of predicted proteins (2539) encoded in the *S. haemolyticus* genome (Fig. 3).

#### *S. haemolyticus* surface proteins

Characteristic motifs of surface proteins such as signal peptides and LPXTG motifs were identified by bioinformatic tools. The covalently anchored cell wall proteins classified as MSCRAMMs are characterized by the C-terminal LPXTG sorting signal. A total of 19 proteins were predicted to have LPXTG motifs based on in silico analysis of the whole genome sequence of *S. haemolyticus* 53–38, of these seven were annotated as adhesion proteins, four were hypothetical proteins and two were DUF 402 and 368.

Of the 325 proteins identified after surface shaving, 65 were annotated as surface proteins (Table 1). Three of the LPXTG proteins identified as adhesins by the in silico analysis were expressed on the *S. haemolyticus* surface. Five LPXTG, one LPXSG and one LPXAG domain containing surface proteins were identified. Three Serine-Aspartate-Repeat (Sdr-like) proteins, the extracellular matrix binding protein (Embp), one Mannosylglucosyl-3-phosphoglycerate phosphatase (SasH-like), and two uncharacterized surface proteins were identified. Other well characterized proteins identified surface proteins were the lytic transglycosylase immunodominant staphylococcal antigen A (IsaA), the Immunodominant staphylococcal antigen B (IsaB) and the elastin binding protein (EbpS).

**Table 1:** Predicted surface proteins after bacterial surface protein shaving of S. haemolyticus

Accession	# PSM	# Unique Peptides	Fold change HaCaT vs Control	***p***-value HaCaT vs Control	LPxTG Cell-wall anchored	Prediction of subcellular localization	Preferred name, EggNOG	Annotation summary
ACAKHAOO_00208	8	7	1.75	**0.046**	–	Extracellular (SPI)	ymaC	DUF867 type protein
ACAKHAOO_02015	7	2	1.60	**0.014**	–	Extracellular (SPI)	sceD	Putative transglycosylase SceD
ACAKHAOO_00540	433	60	1.56	0.123	LPDTG	Cell Wall (SPI)	pelX	Serine-aspartate repeat-containing protein I / YSIRK-type signal peptide-containing protein
ACAKHAOO_01033	54	25	1.46	**0.039**	–	Extracellular (SPI)	atl	Bifunctional autolysin
ACAKHAOO_00522	6	3	1.35	0.054	LPNAG	Cell Wall (SPI)	sasH	Mannosylglucosyl-3-phosphoglycerate phosphatase
ACAKHAOO_00546	188	38	1.34	0.200	LPDTG	Cell Wall (SPI)	–	Serine-aspartate repeat-containing protein I / C protein alpha-antigen
ACAKHAOO_00080	6	5	1.24	0.143	LPKSG	Cell Wall (SPI)	–	Serine-aspartate repeat-containing protein D / YSIRK-type signal peptide-containing protein
ACAKHAOO_02469	10	3	1.24	0.380	–	Extracellular (SPI)	isaA	Putative transglycosylase IsaA
ACAKHAOO_00631	5	2	1.18	0.213	–	Extracellular (SPI)	–	Hypothetical protein
ACAKHAOO_02587	2	2	1.06	0.815	–	Extracellular (SPI)	isaB	Immunodominant staphylococcal antigen B
ACAKHAOO_00744	2	2	1.01	0.916	–	Membrane (SPI)	dtpT	Di-tripepride ABC transporter
ACAKHAOO_02598	8	5	−1.01	0.997	–	Surface^a^(SPII)	proX	ABC transporter substrate-binding protein / Glycine betaine/carnitine transport binding protein GbuC
ACAKHAOO_02593	81	28	−1.01	0.936	LPNTG	Cell Wall (SPI)	–	Cell wall anchor protein / hypothetical protein
ACAKHAOO_01810	3	3	−1.02	0.960	–	Membrane (SPI)	yhaN	Putative protein YhaN
ACAKHAOO_01224	2	2	−1.04	0.907	–	Membrane (SPI)	rseP	Putative zinc metalloprotease
ACAKHAOO_02549	5	3	−1.05	0.766	–	Membrane (SPI)	brpA	Polyisoprenyl-teichoic acid--peptidoglycan teichoic acid transferase TagU / transcriptional regulator
ACAKHAOO_01770	2	2	−1.06	0.763	–	Extracellular (SPI)	lytD	Bifunctional autolysin
ACAKHAOO_01453	7	3	−1.07	0.734	–	Extracellular (SPI)	lapA	Extracellular matrix-binding protein ebh / YSIRK-type signal peptide-containing protein
ACAKHAOO_01492	13	5	−1.07	0.915	–	Surface^a^(Possibly sec)	ebpS	Elastin-binding protein EbpS
ACAKHAOO_01541	2	2	−1.09	0.689	–	Membrane (SPI)	yqhL	Putative protein YibN / sulfurtransferase
ACAKHAOO_00323	5	4	−1.09	0.573	–	Membrane (SPI)	ykuT	Small-conductance mechanosensitive channel
ACAKHAOO_01042	4	2	−1.10	0.582	–	Membrane (SPII)	cyoA	Putative quinol oxidase subunit 2
ACAKHAOO_02168	8	5	−1.10	0.528	–	Membrane (SPI)	cusA	Swarming motility protein SwrC
ACAKHAOO_01077	3	3	−1.10	0.596	–	Extracellular (SPII)	recN	Cell-wall vinding protein / hypothetical protein
ACAKHAOO_01640	2	2	−1.11	0.230	–	Membrane (SPI)	secF	Protein translocase subunit SecDF
ACAKHAOO_01808	9	5	−1.12	0.545	–	Membrane (SPII)	prsA	Foldase protein PrsA
ACAKHAOO_00719	2	1	−1.14	0.487	–	Membrane (SPII)	corC1	UPF0053 protein / HlyC/CorC family transporter
ACAKHAOO_02236	3	1	−1.14	0.633	–	Membrane (SPI)	lyrA	Lysostaphin resistance protein A
ACAKHAOO_01582	14	7	−1.16	0.360	–	Membrane (SPI)	yqfA	UPF0365 protein / hypothetical protein
ACAKHAOO_01462	6	6	−1.17	0.399	–	Membrane (SPI)	ponA	Penicillin-binding protein
ACAKHAOO_01561	3	3	−1.17	0.634	–	Extracellular (No pathway)	sodA	Superoxide dismutase [Mn/Fe]
ACAKHAOO_01806	5	3	−1.18	0.235	–	Extracellular (SPI)	yhaH	Hypothetical protein
ACAKHAOO_00464	28	12	−1.19	0.327	–	Membrane (SPI)	ftsH	ATP-dependent zinc metalloprotease FtsH
ACAKHAOO_01734	49	8	−1.19	0.394	–	Surface^a^(SpI)	ytxG	DUF948 domain-containing protein
ACAKHAOO_02191	7	6	−1.20	0.452	–	Membrane (SPII)	fhuD	Ferrichrome ABC transporter substrate-binding protein
ACAKHAOO_01088	5	1	−1.20	0.591	–	Membrane (SPI)	–	DUF4064 hypothetical protein
ACAKHAOO_01722	15	8	−1.21	0.377	–	Membrane (SPI)	htrA	Serine protease Do-like HtrA/HtrB
ACAKHAOO_02068	2	2	−1.21	0.607	–	Membrane (SPI)	–	Hypothetical protein
ACAKHAOO_01403	2	1	−1.21	0.660	–	Surface^a^(No pathway)	pstB	Phosphate import ATP-binding protein PstB 3
ACAKHAOO_00958	5	4	−1.22	0.357	–	Membrane (SPI)	spsB	Signal peptidase IB
ACAKHAOO_00494	2	2	−1.23	0.243	–	Membrane (SPI)	yacL	Putative PIN and TRAM-domain containing protein YacL
ACAKHAOO_02026	2	1	−1.23	0.314	–	Membrane (SPI)	atpF	ATP synthase subunit b
ACAKHAOO_01924	10	3	−1.24	0.066	–	Membrane (SPI)	–	Hypothetical protein
ACAKHAOO_01062	3	1	−1.25	0.454	–	Extracellular (No pathway)	–	Hypothetical protein
ACAKHAOO_00182	40	16	−1.28	0.265	–	Membrane (SPII)	sitA	Metal ABC transporter substrate-binding protein / Manganese-binding lipoprotein MntA
ACAKHAOO_01347	8	5	−1.29	0.151	–	Surface^a^(SpI)	–	Hypothetical protein
ACAKHAOO_00718	4	3	−1.31	0.223	–	Membrane (SPI)	fruA	PTS system fructose-specific EIIABC component
ACAKHAOO_00753	25	10	−1.32	0.226	–	Surface^a^(SPII)	fatB	Putative ABC transporter solute-binding protein YclQ
ACAKHAOO_01747	12	6	−1.34	0.096	–	Surface^a^(SpI)	–	Hypothetical protein
ACAKHAOO_00561	8	7	−1.34	0.209	–	Membrane (No pathway)	murF	Capsule biosynthesis protein CapA
ACAKHAOO_02597	8	7	−1.35	0.204	–	Membrane (SPI)	ydfJ	Membrane protein YdfJ
ACAKHAOO_01736	2	2	−1.38	0.169	–	Surface^a^(No pathway)	sftA	DNA translocase FtsK/SftA
ACAKHAOO_02099	2	2	−1.38	0.121	–	Membrane (SPII)	fecB	Iron citrate ABC transporter substrate-binding protein YfmC
ACAKHAOO_00362	6	5	−1.39	0.108	–	Membrane (SPII)	penP	Beta-lactamase
ACAKHAOO_00976	9	5	−1.40	0.051	–	Extracellular (SPII)	oppA	Oligopeptide ABC transporter / Dipeptide-binding protein DppE
ACAKHAOO_00003	25	7	−1.41	0.500	LPMTG	Cell Wall (SPI)		Hypothetical protein
ACAKHAOO_01406	17	7	−1.42	0.107	–	Membrane (SPII)	pstS	Phosphate-binding protein PstS
ACAKHAOO_02108	18	7	−1.43	0.261	–	Membrane (SPI)	–	Hypothetical protein
ACAKHAOO_00974	3	3	−1.47	0.139	–	Membrane (No pathway)	oppD	ABC transporter / nickel transport system / Oligopeptide transport ATP-binding protein OppD
ACAKHAOO_00701	11	4	−1.47	0.338	–	Surface^a^(SpI)	–	Hypothetical protein
ACAKHAOO_00229	25	14	−1.49	0.130	–	Membrane (SPI)	pbpC	Beta-lactam-inducible penicillin-binding protein
ACAKHAOO_01885	2	1	−1.53	0.146	–	Membrane (SPI)	yihY	UPF0761 protein
ACAKHAOO_02197	5	2	−1.54	**0.038**	–	Extracellular (SPI)	ssaA	Staphylococcal secretory antigen SsaA / CHAP domain-containing protein
ACAKHAOO_01752	3	2	−1.67	0.102	LPNTG	Cell Wall (SPI)	–	Extracellular matrix-binding protein ebh / Signal peptide protein, YSIRK family / DUF1542
ACAKHAOO_00904	9	4	−1.74	**0.026**	–	Surface^a^(SPII)	metQ	Methionine-binding lipoprotein MetQ

Surface proteins were defined as proteins predicted from cytoplasmic membrane, cell wall or extracellular origin. Positive prediction of subcellular localization was determined by a two out of three or greater concurrent results between the databases

^a^Surface: proteins were predicted as from cytoplasmic membrane, cell wall or extracellular origin, however, concurrent results between two out of three databases were not obtained

#### HaCaT colonisation causes changes in abundance of proteins

We wanted to explore if protein abundance differed when *S. haemolyticus* colonized HaCaT cells compared to when grown in cell culture media supplemented with serum. The large majority of proteins were found similarly abundant when comparing the two conditions, this included EbpS, IsaB and cytoplasmic proteins (Supplementary Table 1).

Only nineteen of 325 proteins (5.8%) showed a significant change in abundance (≥ ± 1.2 fold change) following HaCaT colonization (Table 2). The lytic transglycosylase *Staphylococcus epidermidis* D protein (SceD) (*p* = 0.01) and the autolysin Atl (*p* = 0.04) showed significantly increased abundance with a fold increase of 1.6 and 1.5 respectively when *S. haemolyticus* colonized keratinocytes. The Toll/interleukin-1 like (TIRs) domain protein (*p* = 0.04) also had an increase in abundance (1.4-fold) after HaCaT co-incubation, while the Staphylococcal secretory antigen (SsaA) was significantly (*p* = 0.04) less abundant following keratinocyte colonization, showing a 1.5-fold reduced abundance.

**Table 2:** Proteins with statistically significant altered abundance after surface shaving of S. haemolyticus incubated with human keratinocytes

Accession	# PSM	# Unique Peptides	Fold change HaCaT vs Control	p-value HaCaT vs Control	Prediction of subcellular localization	Preferred name, EggNOG	Annotation summary
ACAKHAOO_01782	3	2	1.90	0.015	Cytoplasmic	metK	S-adenosylmethionine synthase
ACAKHAOO_00208	8	7	1.75	0.046	Extracellular (SPI)	ymaC	DUF867 type protein
ACAKHAOO_02015	7	2	1.60	0.014	Extracellular (SPI)	sceD	Putative transglycosylase SceD
ACAKHAOO_02031	2	2	1.57	0.016	Cytoplasmic	upp	Uracil phosphoribosyltransferase
ACAKHAOO_00454	6	3	1.55	0.027	Cytoplasmic	ctc	50S ribosomal protein L25
ACAKHAOO_01033	54	25	1.46	0.039	Extracellular (SPI)	atl	Bifunctional autolysin
ACAKHAOO_00250	4	3	1.40	0.044	Cytoplasmic	–	TIR domain-containing protein
ACAKHAOO_00947	2	1	1.39	0.032	Cytoplasmic	ppiB	Putative peptidyl-prolyl cis-trans isomerase
ACAKHAOO_02231	2	2	1.35	0.031	Cytoplasmic	–	Putative oxidoreductase YghA
ACAKHAOO_01626	2	1	1.33	0.012	Cytoplasmic	mnmA	tRNA-specific 2-thiouridylase MnmA
ACAKHAOO_01821	4	3	1.31	0.001	Cytoplasmic	nagB	Glucosamine-6-phosphate deaminase
ACAKHAOO_00516	112	20	1.22	0.017	Cytoplasmic	tuf	Elongation factor Tu
ACAKHAOO_00797	45	14	−1.31	0.048	Cytoplasmic	pgk	Phosphoglycerate kinase
ACAKHAOO_01712	7	5	−1.44	0.026	Cytoplasmic	ezrA	Septation ring formation regulator EzrA
ACAKHAOO_01065	2	1	−1.51	0.004	Cytoplasmic	–	DUF697 domain-containing protein
ACAKHAOO_02197	5	2	−1.54	0.038	Extracellular (SPI)	ssaA	Staphylococcal secretory antigen SsaA / CHAP domain-containing protein
ACAKHAOO_01875	14	5	−1.65	0.034	Cytoplasmic	yhbO	Uncharacterized protein SH1084
ACAKHAOO_00904	9	4	−1.74	0.026	Surface (SPII)^a^	metQ	Methionine-binding lipoprotein MetQ
ACAKHAOO_01422	2	2	−1.78	0.000	Cytoplasmic	yaaN	TelA-like protein

Surface proteins were defined as proteins predicted from cytoplasmic membrane, cell wall or extracellular origin. Positive prediction of subcellular localization was determined by a two out of three or greater concurrent results between the databases

^a^ Surface: proteins were predicted as from cytoplasmic membrane, cell wall or extracellular origin, however, concurrent results between two out of three databases were not obtained

#### Moonlighting proteins identified by surface shaving

Several proteins that have previously been shown in other bacteria to have moonlighting functions - proteins dually engaged intracellularly and with important adhesive functions extracellularly - were found among the predicted cytoplasmic proteins. These are the moonlighting proteins glyceraldehyde-3- phosphate dehydrogenase (GAPDH), [24–26], enolase [27], aldolase (ALDA) [26], triose phosphate isomerase (TPI) [28], fructose-bisphosphate aldolase (FBA) [29], ornithine carbamoyl transferase (ARGF) [30], pyruvate kinase (PYK) [31], Inosine 5′-monophosphate dehydrogenase (IMPDH) [32], Clp [33], DNaK [34] and (Atl) [35].

## Discussion

The ability to adhere to and colonize implanted biomaterials in addition to biofilm formation is considered the main virulence factors of *S. haemolyticus* and other coagulase-negative staphylococci.

[1–3]. Despite the clinical relevance of *S. haemolyticus*, published information about virulence factors is scarce compared to literature published on other staphylococcal species. We recently published a comparative analysis of clinical and commensal *S. haemolyticus* isolates [23]. We identified distinct differences in the population structure, where the clinical isolates clustered together separately from the commensal isolates. Clinical isolates were more antibiotic resistant and had different versions of genes encoding surface proteins [23]. In this study, adhesive properties and biofilm formation was compared between clinical and commensal isolates, while the expressed surface proteins were characterized in one clinical isolate after keratinocyte colonization or incubation in cell culture medium supplemented with serum.

### Solid phase host matrix protein binding assay

We found that both fibronectin and collagen binding was low for all *S. haemolyticus* strains. However, fibronectin and collagen binding was significantly higher for commensal compared to the clinical strains. Fibronectin is a glycoprotein found in substantial amounts in blood and loose connective tissue [36] while collagen is an abundant class of proteins in humans, offering structural support to connective tissues and the extracellular matrix [37]. In *S. aureus*, fibronectin binding is described as a crucial step in host cell adhesion. Adhesion mainly involves binding by bacterial fibronectin binding proteins (FNBPs) to fibronectin which forms a bridge between (α_5_)β_1_ integrin on mammalian cells [38]. Low fibronectin binding in *S. haemolyticus* was previously shown when compared to *S. aureus* [39], while a varying capacity of fibronectin binding in clinical *S. haemolyticus* and other CoNS was demonstrated by Switalski et al. [40]. FnBPA and FnBPB involved in *S. aureus* fibronectin binding have not been identified in CoNS so far, but fibronectin binding by the extracellular matrix binding protein (Embp) has been shown in *S. epidermidis*. Expression of Embp in *S. epidermidis* was shown to be induced by supplementation of serum in the growth media [41]. Embp mediates adhesion to fibronectin and biofilm accumulation in *S. epidermidis* [42], and is present in 90% of clinical *S. epidermidis* strains [43]. Cell culture media supplemented with serum was also used in the adhesion assays in this study, where low binding was observed for all strains tested. We identified Embp on the surface of *S. haemolyticus* in the presence of serum. However, if Embp mediates fibronectin binding in *S. haemolyticus*, this did not result in good fibronectin binding in the adhesion assay in this study. Our findings reflect that the role of Embp in fibronectin binding of *S. haemolyticus* needs to be further investigated.

Cooperative binding of collagen in the presence of vitronectin has previously been demonstrated for *S. haemolyticus* [44]*.* Paulsson et al. used different bacterial growth media to induce optimal binding to both collagen and vitronectin. Thus, the type of media used in our experiments might not have been optimal for expression of proteins conferring collagen and fibronectin binding, which also could explain the low binding capacity observed in our experiments.

### Adherence to plastic and semi-quantitative determination of biofilm formation

When we examined the ability to form biofilm we found trends towards more biofilm formation in the clinical strains compared to the commensal strains. However, all strains had the ability to form biofilm. In *S. epidermidis,* similar biofilm forming abilities were observed for both clinical and commensal strains, despite differences in population structure. Rather, different biofilm morphotypes and biofilm encoding genes were found among distinct genetic lineages indicating that biofilm formation is an important property of both commensal and clinical strains [45, 46].

We did not find any correlation between adherence to plastic and the degree of biofilm formation. As adherence is the first step in biofilm formation, one could expect an observed correlation between adhesion to plastic and biofilm formation. The discrepancy in these results can be explained by the use of different media when performing the two assays. It has previously been shown that the amount of biofilm varies depending on the media [47], making comparisons of results from different methods difficult.

### Adhesion to human keratinocytes and bacterial surface protein shaving

We found a trend towards higher adhesion to keratinocytes for the clinical strains compared to the commensal strains. We selected one clinical strain with good adhesive and biofilm forming properties, and performed bacterial surface shaving. To date, most surface protein expression analyses are performed on bacteria incubated in bacterial growth medium [18–22]. As *S. haemolyticus* constitute a significant proportion of the skin microbiota of humans [1, 48, 49], we decided to choose a more biological relevant condition to study protein expression; incubation of *S. haemolyticus* with keratinocytes prior to bacterial surface shaving. Abundance of proteins following keratinocyte colonization was compared to protein abundance following growth in cell culture medium supplemented with bovine serum.

We identified 65 surface proteins in total, of which SceD and Atl were significantly more abundant when *S. haemolyticus* was colonizing keratinocytes. Transglycosylases cleave the β-1,4 glycosidic bond between *N*-acetylmuramic acid and *N*-acetylglucosamine residues of peptidoglycan, accompanied with formation of 1,6-anhydromuramic acid residues [50]. In *S. aureus* the transglycosylases SceD and IsaA are well described virulence factors involved in cell wall remodeling, contributing to resistance to antimicrobial peptides, adhesion and pathogenicity, shown in a murine septic arthritis model [51]. SceD has also been shown to have a pronounced upregulation upon nasal colonization of humans and rats [51, 52].

Biofilm formation is an important virulence factor in *S. haemolyticus*, and in this study we showed a trend towards stronger biofilm formation in clinical *S. haemolyticus* isolates. The bifunctional autolysin Atl was significantly more abundant in *S. haemolyticus* colonizing HaCaT cells. Atl homologs are described in several staphylococcal species [1]. In *S. epidermidis* and *S. aureus*, Atl is important for initial adhesion and biofilm formation [53], and has in *S. epidermidis* been demonstrated to mediate adhesion to vitronectin [54]. In *S. aureus* IsaA is involved in biofilm formation and *isaA* mutants form significantly less biofilm [55]. In this study we identified IsaA when *S. haemolyticus* was grown in the presence of serum. The *S. haemolyticus* biofilm is mainly composed of environmental DNA (eDNA) and proteins [47]. As Atl also mediates adhesion indirectly by hydrolysis of the bacterial cell wall causing the release of proteins and eDNA [1], it is likely that Atl and IsaA expression also in *S. haemolyticus* have similar functions as observed in *S. epidermidis* and *S. aureus* in both adhesion and biofilm formation.

In silico analysis of the genome sequence of the clinical *S. haemolyticus* isolate used for HaCaT colonization identified 19 LPXTG containing genes. Seven of these genes were annotated as genes encoding proteins involved in adhesion, while six had unknown function. These findings resemble what is found in *S. aureus*, where 21 LPXTG genes were predicted in silico, of which eleven had unknown function [56]. In this study, five LPXTG and two LPXSG, LPXAG containing proteins were identified after surface shaving. We identified three Sdr-like proteins which were expressed both when *S. haemolyticus* were co-incubated with HaCaT cells, and when grown in media containing serum. In *S. aureus,* transcription of SdrD and SdrG is increased in the presence of blood and serum [57, 58]. As both tested conditions contained media supplemented with serum, this could explain the expression of the Sdr-like proteins under both conditions.

In *S. epidermidis,* three Sdr proteins have been identified; SdrF, SdrG (Fbe) and SdrH. SdrF has been shown to mediate strong binding to keratins, keratinocytes and nasal epithelial cells [59]. In *S. aureus,* SdrD has been shown to mediate adhesion to keratinocytes through binding to desmoglein1, expressed in human epidermis [60]. The expression of Sdr-like proteins in *S. haemolyticus* after HaCaT colonization and grown in the presence of serum suggests that it might exert similar functions in keratinocyte binding, as found in *S. epidermidis* and *S. aureus*.

HaCaT colonization resulted in the significant upregulation of a TIR protein. TIR domain containing proteins have been shown in several pathogenic bacteria [61], but has not previously been described in *S. haemolyticus*. TirS in *S. aureus* increases survival in the host by blocking the cascade reaction leading to activation of the nuclear factor–ĸB (NF-ĸB), which regulates the expression of a pro-inflammatory immune response [62]. Bacterial circumvention of the host immune defense is an important mechanism in bacterial host colonization.

#### Cytoplasmic proteins

Many of the proteins identified in this experiment were predicted as cytoplasmic proteins. Detection of some cytoplasmic proteins are inevitable when performing surface shaving [10, 63]. The presence of predicted cytoplasmic proteins after bacterial surface shaving can be due to cellular lysis, moonlighting proteins or protein containing membrane-vesicles (MV) [10, 63, 64].

We recently showed that *S. haemolyticus* produces MVs [65]. The *S. haemolyticus* MV cargo mainly contained cytoplasmic proteins, amongst them several moonlighting proteins, which are proteins that express more than one function when transported to a different cellular location [24]. Release of MVs in incubation buffer after culturing and washing of cells might add to the identification of predicted cytoplasmic proteins [10].

#### Strengths and limitations of the study

The main advantage of the developed method is the direct contact between bacteria and mammalian cells before bacterial surface shaving, mimicking a more relevant host-microbe interaction compared to other protein expression systems. *S. haemolyticus* surface shaving subsequent to colonization of human keratinocytes has to our knowledge not been described before. By using the LPI™ approach for bacterial surface shaving, whole cells are immobilized by a passive process (personal communication Nanoxis Consulting AB) within a flow cell prior to digestion, allowing binding of intact cells only. In this study we only used one clinical isolate. In order to find surface proteins that are present only in clinical vs. commensal isolates, several isolates from different commensal and clinical lineages need to be compared.

The separation of bacteria from the mammalian cells by FACS is time consuming, leading to a low throughput of samples. The individual sorting of samples before being concentrated and subsequently subjected to surface shaving in individual LPI flow cell channels, might have led to slight variations in the concentration of cells or even slight differences in expression due to slight differences in handling time. However, we kept all samples on ice and in PBS throughout the experiment in order to minimize potential alteration of gene expression.

## Conclusion

This is to our knowledge the first described study using surface shaving of expressed staphylococcal proteins after direct contact with eukaryotic cells and in cell culture media supplemented with serum. Gaining information about surface exposed proteins is important in order to better understand host-pathogen interactions, biofilm formation and for the discovery and design of novel targets for antimicrobial and anti-biofilm treatment. Thus, this method is transferable to other bacterial species and mammalian cell types. The method has provided novel knowledge about the *S. haemolyticus* surface proteins in a clinical isolate. We have identified surface proteins and immune evasive proteins previously only functionally described in other staphylococcal species. We have also identified hypothetical surface proteins, of which future analysis should be undertaken in order to describe function. Further functional assays should be performed to determine the importance of the different identified proteins in host microbe interactions and biofilm formation.

## Methods

### Bacterial strains and mammalian cell lines

Ten clinical and ten commensal *S. haemolyticus* strains were included in the study (Table 3). The clinical strains are a subset of a larger collection, isolated from blood, catheters and wounds [2]. The commensal strains are a subset of a collection of strains from the skin of healthy adults [49]. HaCaT cells were from a human keratinocyte cell line [66] (Cell Lines Service (CLS), Germany, no. 300493).

**Table 3:** S. haemolyticus strains included in the study

Sample	Country	Isolated from	Year of isolation	ENA ID^**a**^	Lab. ID
1	Norway	Blood	1995	ERS066267	25–12
2	Norway	Blood	2004	ERS066284	51–11
3	Norway	Blood	2002	ERS066281	51–08
4	Switzerland	Blood	2001	ERS066398	53–18
5	Germany	Blood	2008	ERS066335	53–73
6^b^	Switzerland	Wound	2004	ERS066380	53–38
7	Norway	Blood	2004	ERS066295	51–29
8	Switzerland	Blood	2004	ERS066370	53–35
9	Switzerland	Unknown	2006	ERS066381	53–49
10	Switzerland	Blood	2005	ERS066386	53–48
11	Norway	Nasal Swab	2010	ERS066315	54–64
12	Norway	Armpit	2013	ERS3370776	57–01
13	Norway	Groin	2013	ERS3370780	57–12
14	Norway	Armpit	2014	ERS3370802	57–66
15	Norway	Groin	2014	ERS3370809	58–28
16	Norway	Hamstring	2013	ERS3370784	57–22
17	Norway	Groin	2014	ERS3370790	57–33
18	Norway	Groin	2014	ERS3370800	57–61
19	Norway	Groin	2014	ERS3370806	58–08
20	Norway	Unknown	2013	ERS3370815	58–62

Ten clinical and ten commensal S. haemolyticus strains were included in the study. Samples 1–10 are clinical strains and 11–20 are commensal strains

^a^ENA = European Nucleotide Archive.

^b^ Strain no. 6 was chosen for bacterial surface protein shaving

### Solid phase host matrix protein binding assay

The ability of *S. haemolyticus* to adhere to collagen, fibronectin and plastic was determined using a protocol based on Edwards et al. [67]. Bacterial cultures were grown for 10 h (Optical density (OD)_600_ 0.7–1.0) in Dulbecco’s Modified Eagle’s Medium (DMEM) (Merck, Germany) with 10% heat inactivated Fetal Bovine serum (FBS) (Thermo Fisher Scientific, MA, USA), pelleted and re-suspended to a concentration of 10^8^ colony forming units (CFU)/mL. Microtiterplates (96 well) pre-coated with collagen (Thermo Fisher Scientific, MA, USA) or fibronectin, 1 μg/well (R&D Systems, MN, USA) were blocked with 150 μl 3% Bovine Serum Albumin (BSA) (Merck, Germany) for 1 h at room temperature and then washed 2x with Phosphate Buffered Saline **(**PBS) (Merck, Germany). Inoculum was added to plastic (CAT.NO 163320, Thermo Fisher Scientific, MA, USA), collagen and fibronectin plates and incubated for 1 h at 37 °C followed by 1x wash with PBS. The plates were fixed at 55 °C for 1 h and stained with 0.25% crystal violet (Merck, Germany) for five minutes. Biomass of adherent bacteria was determined by solubilization of crystal violet with 150 μL 70% EtOH. Absorbance (Abs) was measured at 590 nm (Versamax, Molecular Devices, CA, USA). Values from bacterial binding to wells coated with BSA only were subtracted.

### Semi-quantitative determination of biofilm formation

We performed semi-quantitative determination of biofilm production as described previously [47, 68]. Biofilm formation was induced in Tryptic Soy Broth (TSB) (BD, NJ, USA / Merck, Germany) with 1% glucose (Merck, Germany) in 96-well microtiter plates (Thermo Fisher Scientific, MA, USA). All strains were tested in eight wells with three parallel runs and controls were included on each plate. After 24 h, wells were washed, fixed and stained with 0.1% crystal violet (Merck, Germany). Crystal violet was dissolved from the biofilm with 70% ethanol for 10 min and Abs_570_ was determined (Versamax, Molecular Devices, CA, USA). We removed the highest and lowest outlier for each parallel and the remaining six values were averaged. Based on the distribution of the tested strains, strains with average OD values over 1 were considered strong biofilm-producers.

### Adhesion to human keratinocytes

*S. haemolyticus* adhesion to human keratinocytes (HaCaT) was determined. HaCaT (2 × 10^5^ cells/ml) were added to 24-well plates (Thermo Fisher Scientific, MA, USA) and allowed to attach for 16 h (37 °C, 5% CO_2_) in DMEM with 10% FBS. Bacterial cultures were grown at 37 °C to late exponential phase (OD_600_ 0.7–1.0) in DMEM with 10% FBS, and then washed twice in Dulbecco’s Phosphate Buffered Saline (DPBS) (Merck, Germany). Approximately 2 × 10^6^ CFU in DMEM with 10% FBS were added to each well of a cell culture plate to achieve a multiplicity of infection dose (MOI) of 10:1. The plates were centrifuged at 900xG (Eppendorf 5430R, Germany) for 10 min at 37 °C and incubated for 30 min. at 37 °C in 5% CO_2_ [69]. After incubation, the plates were thoroughly washed to remove all unbound bacterial cells. To enumerate the number of adhered bacteria, 0.25 mg/mL Trypsin-EDTA (Merck, Germany) and 0.1% mg/mL Triton X-100 (Merck, Germany) were added, and the suspension was pipetted in order to fully lyse the HaCaT cells. CFU/mL was determined by plating on blood agar plates (Thermo Fisher Scientific, MA, USA) and incubated at 37 °C overnight. Three biological replicates were performed.

### Bacterial surface protein shaving

#### Preparation of bacteria for cell surface shaving

To explore the expression of surface proteins in *S. haemolyticus* when colonizing HaCaT cells, one clinical bacterial strain (53–38) with strong adhesive and biofilm-forming properties (Table 3) was co-incubated with HaCaT cells. We wanted to further explore this isolate as adhesion and biofilm formation is regarded as important virulence traits in the coagulase negative staphylococci. A bacterial control sample (same bacterial isolate) grown in cell culture media supplemented with serum but without HaCaT cultivation was included. Three biological replicates were performed for all samples and both conditions. The workflow of the bacterial surface shaving experiment is summarized in Fig. 1 and Supplementary Table 3.

HaCaT cells were seeded in 6-well plates, and bacterial cultures were grown to late exponential phase (OD_600_ 0.6 ± 0.1) in DMEM with 10% FBS, washed twice in DPBS and resuspended in DMEM with 10% FBS and further handled as previously described for the HaCaT adhesion assay. A MOI of 100:1 was used and bacteria were centrifuged with HaCaT cells for 10 min, and further incubated for 50 min. After incubation, tissue culture plates were washed 4 times with DPBS to remove free-floating bacteria. Mechanical detachment of eukaryotic and bacterial cells from the tissue culture plates was performed with a cell scraper (VWR, PA, USA) followed by pipetting in DPBS. Cells were transferred to polystyrene tubes with a cell strainer cap (Thermo Fisher Scientific, MA, USA). Twelve wells from two tissue culture plates were used for each replicate.

The samples were prepared for Fluorescence-activated cell sorting (FACS), in order to separate bacteria from HaCaT cells, by labelling with the Vancomycin BODIPY™ FL Conjugate (Thermo Fisher Scientific, MA, USA) (0.6 μg/mL), targeting the Gram-positive bacterial cell wall [70].

The bacterial control samples that were not co-cultivated with HaCaT cells were grown to late exponential phase in DMEM with 10% FBS (OD_600_ 0.6 ± 0.1) and resuspended in DPBS after centrifugation and washing and further stored on ice. Samples were then prepared for FACS by Vancomycin BODIPY™ labelling, in order to treat the bacterial control samples in a similar manner to the test samples.

#### Fluorescence-activated cell sorting system (FACS)

*S. haemolyticus* was sorted from HaCaT cells by using FACS Aria III (BD, NJ, USA) (Software BD FACSDiva 8.0.1), according to size and fluorescence. Based on the size of single bacteria (1 μm) and the fluorescent signal strength, the gating was set to sort single or doublets of bacteria. Fluorescent beads (Polystyrene Particle, Flow Cytometry grade PPS-6 K and Nano Blank Polystyrene NFPPS-52-4 K (Spherotech, IL, USA)) were used for calibration. Vancomycin BODIPY™ was excited with a 488 nm blue laser. A FITC-detector was used to read the emitted green, fluorescent light. Normal density filter 1.0 was used in front of the FSC detector. After FACS all samples were stored on ice.

#### Surface shaving - sample processing and generation of peptides by LPI™ HexaLane flow cell

**Fig. 4:**
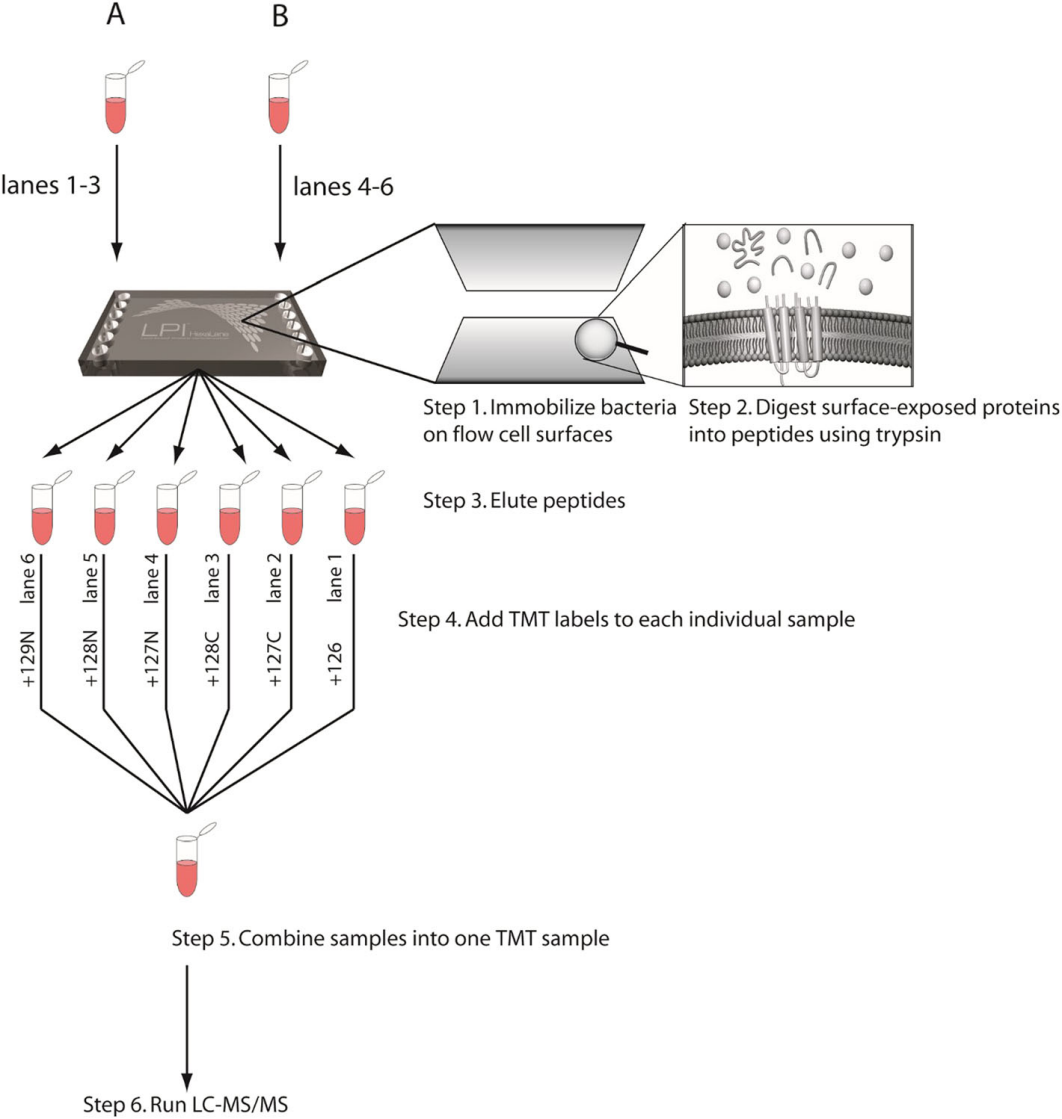
The use of LPI™ methodology together with TMT labelling when performing surface shaving. Three lanes were filled with bacterial cells after exposure to HaCaT cells (**a**) and three lanes were filled with bacterial cells only exposed to media (**b**). After surface shaving, the eluted peptides were tagged with TMT labels, pooled and subsequently analyzed by LC-MS/MS.

In order to concentrate the bacterial samples after FACS (≈230 mL), samples were centrifuged twice, both steps at 10000xG for 30 min at 4 °C in swing bucket rotors (Beckman Coulter, CA, USA), resulting in samples containing approximately 2.8 × 10^7^ CFU/mL. The concentrated samples were resuspended in ice cold PBS, kept on ice and immediately loaded into the LPI™ HexaLane Flow Cell (Nanoxis Consulting AB, Sweden), as seen in Fig. 4, step 1. To allow bacterial attachment, the flow cell was incubated for 35 min at room temperature. The cells attach to the gold coated channels in the Flow Cell by a passive process (personal communication Nanoxis Consulting AB). Unbound bacteria were removed by washing the channels with 200 μL PBS using a syringe pump (Harvard Apparatus, MA, USA) at a flow rate of 50 μL/min. Enzymatic digestion of bacterial surface proteins was performed by injecting 100 μL of trypsin (Promega, WI, USA) (40 μg/mL) into the LPI HexaLane Flow Cell channels and further incubated for 20 min at room temperature. After digestion, peptides were eluted in 200 μL PBS and the digestion was terminated by adding 4 μL formic acid (neat) (Merck, Germany). The peptide samples were centrifuged for 10 min at 10000xG, in order to remove any cell debris and the supernatants were subsequently dried using a SpeedVac (Eppendorf, Germany) and stored at − 20 °C.

#### Protein identification and relative quantitation

The proteomic analysis was performed at The Proteomics Core Facility at Sahlgrenska Academy, Gothenburg University. Digested peptides were dissolved in 100 μL triethylammonium bicarbonate (TEAB) (350 mM, Thermo Fisher Scientific, MA, USA) and labelled using TMT 10-plex isobaric mass tagging reagents (Thermo Fisher Scientific, MA, USA) according to the manufacturer’s instructions. The TMT-set were fractionated into twelve fractions using Pierce High pH Reversed-Phase Peptide Fractionation Kit (Thermo Fisher Scientific, MA, USA) according to the manufacturer’s protocol, but with a modified gradient (Supplementary Table 4).

The fractions were analyzed on a QExactive HF mass spectrometer (MS) interfaced with Easy-nLC1200 liquid chromatography system (LC-MS/MS) (Thermo Fisher Scientific, MA, USA). Peptides were trapped on an Acclaim Pepmap 100 C18 trap column (100 μm × 2 cm, particle size 5 μm, Thermo Fisher Scientific, MA, USA) and separated on an in-house packed analytical column (75 μm × 300 mm, particle size 3 μm, Reprosil-Pur C18, Dr. Maisch, Germany) using a gradient from 7 to 35% B over 70 min followed by an increase to 100% B for 5 min at a flow of 300 nL/min. Solvent A was 0.2% formic acid and solvent B was 80% acetonitrile, 0.2% formic acid. The instrument operated in data-dependent mode where the precursor ion mass spectra were acquired at a resolution of 60,000, the 10 most intense ions were isolated in a 0.8 Da isolation window and fragmented using collision energy HCD settings at either 28 or 50. MS2 spectra were recorded at a resolution of 60,000, charge states 2 to 4 were selected for fragmentation and dynamic exclusion was set to 20 s with 10 ppm tolerance.

MS raw data files for the TMT set were merged for identification and relative quantification using Proteome Discoverer version 1.4 (Thermo Fisher Scientific, MA, USA). *S. haemolyticus* 53–38 with European Nucleotide Archive (ENA) accession number GCA_001226325.1 (Illumina sequence) and ENA accession number PRJEB36042 (PacBio sequence) [2] were aligned using BWA-MEM [71] and further used as reference proteome (2539 coding sequences). Structural and functional annotations were performed using Prokka [72]. Mascot 2.5 (Matrix Science Ltd., UK) was used as a search engine with precursor mass tolerance of 5 ppm and fragment mass tolerance of 200 mmu. Tryptic peptides were accepted with one missed cleavage and variable modifications of methionine oxidation, cysteine alkylation and fixed modifications of N-terminal TMT-label and lysine TMT-label were selected. Fixed Value of 13 was used for identification and the quantified proteins were filtered at 1% False Discovery Rate (FDR) resulting in a mascot score of at least 20. No missing values were present in the data set at Threshold of 2000. Proteins were grouped by sharing the same sequences to minimize redundancy. The resulting ratios were normalized in the Proteome Discoverer 1.4 and the sum of the samples cultivated with HaCaT was used as denominator. Only unique peptides were used for comparison between groups.

The mass spectrometry proteomics data has been deposited to the ProteomeXchange Consortium via the PRIDE partner repository with the dataset identifiers PXD014450.

### Statistical analyses

For the results from biofilm-, solid phase host matrix protein and HaCaT adhesion assays the data were analyzed using IBM SPSS software, version 25.0. The non-parametric Mann-Whitney U-test was used to compare two groups, a *p* value < 0.05 was considered statistically significant.

As the technical variation for the identified proteins was assumed to be 20%, only proteins displaying a higher degree of fold change (FC) than ±1.2 were considered as biologically significant regarding increased or reduced abundance of proteins. The most changed abundance of proteins had a threshold of at least ±1.5. Welch’s t-test was performed (3 parallels vs. 3 parallels) and only proteins passing filter *p* < 0.05 were considered statistically significant.

### Bioinformatic analyses

LPXTG motifs were predicted in silico from the whole genome sequence of *S. haemolyticus* 53–38 using a manual sequence search. Prediction of the subcellular localization of proteins was done using PSORTb v.3.0 algorithms [73], CELLO v.2.5 [74] and LocateP v.2.0 [75]. Positive prediction of subcellular localization was determined by a two out of three or greater concurrent results between the databases. Surface proteins were defined as proteins predicted from cytoplasmic membrane, cell wall or extracellular origin.

Functional annotation of proteins was done with the EggNOG v.5.0 database with HMMER and Diamond mapping mode; i.e. functional description, seed orthologues, predicted name, KEGG KO and categorization of proteins into Clusters of Orthologous Groups of proteins (COG) [76], PHMMER v.3.3 [77, 78] and protein BLAST [79].

Moonlighting proteins were identified by using the MoonProt database and by manual searches based on published literature [80, 81].

## Supplementary information

**Additional file 1: Table S1.** The cell surface shaving and LC-MS/MS analysis results identified 325 proteins with ≥ #2 peptide-spectrum matches (PSMs).

**Additional file 2: Table S2.** FASTA sequences of the proteins from the cell surface shaving and LC-MS/MS analysis.

**Additional file 3: Table S3**. Workflow for bacterial protein surface shaving samples. X = performed, − = not performed

**Additional file 4: Table S4**. Manufacturer’s and modified gradient using the Pierce High pH Reversed-Phase Peptide Fractionation Kit.

## Data Availability

The proteomic raw data supporting the conclusions of this article is available in the ProteomeXchange Consortium via the PRIDE partner repository with the dataset identifier PXD014450 [82]. The whole genome sequence of the strain used for surface shaving is depoasited in the European Nucleotide Archive with the unique identifier ERS066380 [83] and PRJEB36042 [84]. The dataset supporting the conclusions of this article is included within the article (and its Additional files S1–4).

## Abbreviations

Abs: Absorbance
ALDA: Aldolase
ARGF: Ornithine carbamoyl transferase
Atl: Autolysin
BSA: Bovine Serum Albumin
CFU: Colony Forming Units
CLS: Cell Lines Service
COG: Clusters of Orthologous Groups
CoNS: Coagulase Negative Staphylococci
CWA: Cell Wall Anchored
DMEM: Dulbecco’s Modified Eagle’s Medium
DPBS: Dulbecco’s Phosphate Buffered Saline
DUF: Domain of Unknown Function
Ebps: Elastin binding protein
eDNA: Environmental DNA
Embp: Extracellular matrix binding protein
ENA: European Nucleotide Archive
FACS: Fluorescence-activated cell sorting
FC: Fold Change
FBA: Fructose-Bisphosphate Aldolase
FBS: Fetal Bovine Serum
FDR: False Discovery Rate
FNBPs: Bacterial Fibronectin binding proteins
GAPDH: Glyceraldehyde-3- phosphate dehydrogenase
HaCaT: Human Keratinocytes
IgG: Immunoglobulin G
IMPDH: Inosine 5′-monophosphate dehydrogenase
IsaA: Immunodominant staphylococcal antigen A
IsaB: Immunodominant staphylococcal antigen B
LC-MS/MS: Liquid Chromatography tandem Mass Spectrometry
LPI: Lipid-based Protein Immobilization
LPXTG: Leu-Pro-X-Thr-Gly; where X can be any amino acid
MOI: Multiplicity Of Infection
MS: Mass Spectrometry
MSCRAMM: Microbial Surface Component Recognizing Adhesive Matrix Molecule
MV: Membrane Vesicle
NF-ĸB: Nuclear Factor–ĸB
OD: Optical Density
PBS: Phosphate Buffered Saline
PRIDE: PRoteomics IDEntifications database
PSM: Peptide Spectrum match
PYK: PYruvate Kinase
SasH: Mannosylglucosyl-3-phosphoglycerate phosphatase
SceD: Lytic transglycosylase *Staphylococcus epidermidis* D protein
Sdr: Serine Aspartate repeat containing protein
SsaA: Staphylococcal secretory antigen
TEAB: Triethylammonium Bicarbonate
TIR: Toll/interleukin-1 receptor
TMT: Tandem Mass Tags
TPI: Triose Phosphate Isomerase
TSB: Tryptic Soy Broth
