# Supplementary material for: Identification of surface proteins in a clinical Staphylococcus haemolyticus isolate by bacterial surface shaving
Source: BMC Microbiol. 2020 Apr 7;20:80. doi: 10.1186/s12866-020-01778-8 (PMC7137321; doi:10.1186/s12866-020-01778-8)
Supplement: Supplementary file 2 — Additional file 2: Table S2. FASTA sequences of the proteins from the cell surface shaving and LC-MS/MS analysis. [file 12866_2020_1778_MOESM2_ESM.pdf]

>ACAKHAOO_00003 hypothetical protein
MIKRRFKYAFGTTLVTSSLLLMSHGHAFAVTNNIDQAFNHDNNGQPITSQDSNNNVDPGF
NHDVDDNNDTDPGFNHDVIHNNDPAKHQAEDNDMGFNGYAEQNGLVKQTSKSNSQQDPAK
HQAEDNDMGFNGYVEQNGLVKQTSKSNSQQDPTKHQAEDNDMGFNGYAEQNGLVKQTSKS
NSQQDPTKHQAEDNDMGFNGYVEQNGLVKQTSKSNSQQDPTKHQAEDNDMGFNGYAEQNG
LVKQTSKSNSQQDPAKHQAEDNDMGFNGYAEQNGLVKQTSKSNSQQDPAKHQAEDNDMGF
NGYAEQNGLVKQQDQSPKSQMNSTLSKQATRSQETSQTSLHHELPMTGERASTTTFVYSF
IAFILGVGVISLRRKFSK
>ACAKHAOO_00032 Pyrrolidone-carboxylate peptidase
MKILVTAFDPFGGEKINPALEAVKQLENTIGEHTISKLEIPTVFHESKDVIDKELANGNY
DVVLSIGQAGGRYDLTPERVGINIDDARIPDNKGNQPIDVAIQEDGAPAYFSNLPVKTMT
EAIKAAGVPASLSNTAGTFVCNHVLYQLGYLADKSYPGLLFGFIHVPFIPEQVTDKPEKP
SMSIETIAKGLTAAIKAISKEDDAKVALGETH
>ACAKHAOO_00061 Glycine cleavage system H-like protein
MAKLANYLYVEKVGNQYIYRMTPELQDDVGTVGYVEFMGPDDVNVNDEIVSIEASKTVLD
VQSPLAGKIVERNTKAEDEPAILNSEKPEENWLVKLENVDEAAYDALPDPDED
>ACAKHAOO_00068 Carbamate kinase 1
MSKIVVALGGNALGQSPEEQLELVKGTAKSLVSLIQKGYEVVISHGNGPQVGSINLGLNY
AAENGQGPAFPFPECGAMSQAYIGYQLQESLLNELHALNIDKQVVTLVTQVEVAGDDQAF
NNPTKPIGLFYTKEQAEQTMEEKGYKFVEDSGRGYRRVVPSPMPINIVELDSIETLIKHG
TLVIAAGGGGIPVVKEEGNYKGVDAVIDKDKTSALLAAHLKSDQLIILTAVDYVYINYGK
DNQEALGEVTVDEMNQHIADGQFAKGSMLPKVEAALQFIEKNPEGSVLITSLEDLGDALD
GKIGTLIKK
>ACAKHAOO_00069 Ornithine carbamoyltransferase
MQNLRNRSFLTLLDFSQKEVEFLLNLSEDLKRAKYAGIEQQKLKGKNIALIFEKDSTRTR
CAFETAAYDQGAHVTYLGPTGSQMGKKESAKDTARVLGGMYDGIEYRGFSQRTVEELAKY
SGVPVWNGLTDEDHPTQVLADFLTAKEVLKKPYHEINFTYVGDGRNNVANALMQGAAIMG
MTFHLVCPKELNPTDELLNRCKEIAAKNGGEILVTDDIDEGVKGSDVIYTDVWVSMGEPD
EVWEKRIKLLEPYRVTKEMMEKTGNPRAIFEHCLPSFHDTETKIGKEIQEKYGLTEMEVA
DEVFESEQSVVFQEAENRAHTIKAVMVATLGE
>ACAKHAOO_00080 Serine-aspartate repeat-containing protein D
MDNKQLGNKRESFSIRKYSIGTASILVGSLLFLGGGEALAAESNESNTSEKVPMEQTQSA
QEQPSHEASTERVEQPQNKAIATQETKTNEDASSEETQSNEADTESNEVIHNDTQSNETS
EQSTAEPKTQQNKASDDVTTQEVTSTEQPTQEKTQSTSKQEVTTQETSTTEVQPKNEQSQ
TEEQPQHETKETYSQSNTQEAPTKETKKPTQPTTENKQSEKSNTTQQTNQKANSEVSQSN
VTSTEEVTNTNETRTRTQPFSKEEMNKVTATEESDVAVSDVPQTQMKYMSTKQKQVLFNT
LQRDANTQDQQPKAILSTIPEWTSAQRTKNASRTGQDQLNITHKGRYTSGADFGNGGTEI
VKYNPKNGYAYSVNGDKEALDIIDVKHPGKDGAINLVKRIYLQDNGIEAGDLTSVTVHPS
GDYVAVSAPAVDKTKPGHVVFYGSNGDYINNVTVGSLPDMVTFSKDSKYLLVANEGEPSD
DYTVNPPGSVSVIDVTGGPANVTANNVRTAMFTKEHQEGIRALGPNAEDAYLNIEPEYIA
VDSQSKYAYVTLQEVSAIAKVDIVKGQIVQVKGLPYKDHSLAQNAMDVSDEDGKSELRRV
PVLGLLQPDGIDTYDYNGETYLLIANEGDSQDYEGYSEEKRVKKLKDDIQLDARYYQGYT
QAELDDMVDNGLFDDEQLGRLKVTTSHAFKDADGKYNALVSYGGRSFSILRASDLEMIYD
SGSDIEQRVLDLLPERFNANYESADDIKVDDRSDDKGPEAENVVVGKVGSHSYAFVGLER
VGGIMIYDITNPNEPYFVKYLYDLDNKDISPEGITFESAEESPNGKPMLIASFELSGTTS
AWELEDLTGDQESDDGEDSDNPGNTNGESNDDDTTPNPPSENDGSNTDHDVDNGEQPDNG
DDTSSNDEDNHSSDDTNGNDEGLEPPYEVDGDIFEDDSDEVISDKEDNNDTSAQSHEEVE
SKGASHNINHSAHANHQEESRDKLTVNSNTHNLSVVSTKSSSHHAVTTNHGSVVSDKSNH
ANTSSDNVKKELPKSGQTETNTTLWSVLLGGLGLAFIRKRKASKSEK
>ACAKHAOO_00087 Teichoic acid poly(glycerol phosphate) polymerase
MQIIKQDGSILHIYFENPINKIEKLYIKNEFNSLTLEKTNGDLHFKIDLVEVAKQFIQYE
NKKIYIIVEESTPILTTQSNLKVTNSQSEVSGLTEVLIDDSENIKIQPYITRNGYLHLSL
DDVDLNKTYFSRRHIDTLKINNSEAIIEGQFATLNSTLKDVQLLIKTRLTDREKFVNINL
EEIGKTKNASTYNFSVDIYKDLIEFMKYEYKNEDIIDIYLSIKVLESSENLRIKIGNPRI

MAEKLLKGEIITEFNGNIVSLTPYFTMKGRNLSFRINTYDIDSYLTYVDELKRPKRPNFL
KKENQKIWVIGEKSYKAQDNGYHFFKYMRTYHPDKPVYYIIEKDSKEAENVLPFGNVIFY
NSAEHFKIMIKADYICTTHHPELIYPTTSDIYTRKIKAVKVFLQHGVLGTKNLSNINGNQ
LKDFNVDLFITSSSREKEIVVRDLNFFSNQVAVTGLARFDELFNPNVEVKNQILIIPTWR
DWLTSIDRLQTSEYLKRMNDLLHSQKLKEIANKGTDIIFCLHPNMQPFIDYFDVPSYVTS
IKQGDIDVQKLIKESKLMITDYSSVAFDFSFLNKPVIYYQFDRNEFLGKEASHIDIEKEL
PGIIVNAQDKLERELDNIQKKQFKIDEALEKRIDRFIAYKDRNNCERIFDAITNFRETSK
VKSKIKYDILSQHVVNRFRKDKKYFKVMEKFNTGLTRLLPVKKDLIVFESNVGKLVGDSP
KFIYDELKKYNKKYQIVWATNTLYPFNDPNVKTVKRLSPEYFYYLSRAKYWINNQNFPHY
LRKHKDTIYIQTWHGTPLKKMLNDVETFQGRDANYKNRVNQAIKNWDYLISPSPYASACF
KTAFDFKNEILEIGYPRNDVFYNVENTDMTEKKKLIKQKLGIESNKKIILYAPTFRDDEI
NKAKKHIINLKLDLPRLKESIGDEYILILRPHIIISNALNLSESLNDFVVDGTKYPEISD
LYLITDICITDYSSVMFDFANTKKPLLFFTYDLEFYKNKLRGFYFDFEKEAPGPLIKTND
GLIEAIKNITNVQEEYDDKYQVFYNRFCTFEKGIASKTIVDKFFKQDC
>ACAKHAOO_00098 Dihydrolipoyl dehydrogenase
MKTTSKVKGVQMMSKSYDLIVIGAGPGGYVAAIRAAQLGKNVAIVEKQHVGGVCLNVGCI
PSKIFLEYGAKVRDINSANNWGIKTNHIDIDVTSLVQRKDQVVKTVTDDVRDALRQHNVD
FIEGEAEVLEGLKVQVDQTIYTAKDIILATGTKPFVPPIEGLDKAHFETADTFFDMEQLP
KQLVIIGGGVIASEIASSMADLDVDVTILEKGDSILSSEIKEIRDHLSTYLKQQGVNIIT
NSETKKVNAKTLEIDGKDGPKEIPYETLLFATGRQPNVHVAKALNLEQNGKCLQVNEHYE
TSYAHVYAIGDLVPGYQLAHTASAHGKYVAEYIAGKQLETINQEDIPRCIYTRLESASVG
LSELQAQEAGYEVEVTTAPFQKNPKAILKGETQGMVKIVANKQDGKILGGFVVGPHATDL
ISEILGIKVSGGTLNDISQIIQPHPSLSEVIGESADASFGKAIYQ
>ACAKHAOO_00099 Dihydrolipoyllysine-residue acetyltransferase
component of pyruvate dehydrogenase complex
MSENIIMPKLGMTMKEGTVEEWFKAEGDVVEQGESICTISSEKLTQDVEAPASGTLLEIK
VQAGEETEVKSVLGIIGDEGESTESSSTTESENEEQNNSHSENEEQQTSDTTSVNEDDSN
HDEDTNKSNNVEPQNGKRIFISPLARKMAEDKNFDITRIQGTGGNNRITKLDIQRVEEQG
YDIEAKNEDMQSKEDKNTKFNSANVGEGLNPMRKRIAQNMRQSLAETAQLTLHRKVDADR
LLDFKNKLSAELENANQDTKLTVTALLAKAIVLALKDYGAMNARYENGQLTEYDDVHLGV
ATSLDEGLMVPVINNADTKSIGALAKEIKSSAEAVRDGNTNDVKLAGATFTITNMGTSGI
EYFTPILNLGETGILGVGALSKEVVLEGDSVKQVSRIPLSLTFDHQILDGASAADFLKVL
AKYIENPYLLIL
>ACAKHAOO_00106 Protein/nucleic acid deglycase 1
MNTKRALVVVTSVTKYPDMNRPTGLWFGEVVHFADVLYKNGYKVHYISPEGGYTAIDPQS
LQEDMMTDLDWEYYQNKDFMTRLGHTVTPDAVKAENYDVIYFAGGHGTIWDFKDNEDLQK
LTQEIYENNDVVASVCHGAIGLLNVKDAEGKHIIDGKTVTGFSNSEEQAVGLADVVPYLT
EDELKNRGANYEQGDNWTEFAVADGRIITGQNPQSGKAVAEKFLESQK
>ACAKHAOO_00118 hypothetical protein
MKEQVLVTGGTGFLGLRIVAELLKQDYSVRATIRSLSKKDTILETLKAQNIDT
>ACAKHAOO_00128 hypothetical protein
MKKLLFMCGVFLLLLAACGSNDGSKEDSAKTKSNAEQTKSKKSKKEQKLEKEVAKLSEKQ
KLALAFCVEDVDRYTLTKNEILTGIYEYKLATGNKNFKLVDFKLVKYDDSIKNAPKGMNF
YNVSPNKGNFEALIGVSNDKIFIGRMQSGSLDYKDLLGKGKEVKLIDVYKANKDNKALPE
LTDKINIVDSLSKKDKENNNPLSAEYLEKSGTVNTHFRNQVYQMISDFEGIAVGKTNYLW
DDVKMVGHSGDWIVNYRNKDGEILGTYKTKNNKIIKLDANGKVIKQEN
>ACAKHAOO_00159 putative ABC transporter permease
MFLAWNEIKRNKLKFGLIIGILVLISYLLFLLSGLASGLINMNTEGIKKWKADAIVLNKD
ANQTVQQSVFKSSDVEGKFKEEAPLKQIGVIASNGESEENALLFGVTSNSFLIPKIEEGK
KFNKDNDVVIDQSLKDKGFKVGDTITLSQSDEKLHIVGVSESAKYNASPVIFANDKTIEK
INPALSSDKTNAVVVKDSNWKDKNVDKDLEVIGIDDFVENLPGYKPQNLTMNFMITFLFV
ISATVIGVFLYVITLQKKNLFGVLKAQGFTNGFLMKMVLAQTFILALIGTLIGLILTLLT
SLVLPEAVPVQFNIGTLIIFGIVLILTSLVGSLFSVLSIRKIDPLKAIG
>ACAKHAOO_00180 High-affinity zinc uptake system ATP-binding protein

ZnuC
MIDISNMNLHLNHKHVLKDITLKIPISGEIIGIMGPNGAGKSSLLKSLIGSFNASGEMNL
YGKSIHKQLQYITYIPQKAQLDLDFPINVEKVILSGCYQTIGWFKRVDHASKMKFHKLLR
DLDLESLQYKQISELSGGQLQRVLVARALMTDSTIYLLDEPFVGIDFNSEQLIMEKLQHL
KNQGKLILIVHHDLSKAEQYFDRILLLNRTVRFFGPSHQAMQPQNLNRTFLFNVTSTLKE
GSDISND
>ACAKHAOO_00182 Manganese-binding lipoprotein MntA
MKKFIPLLIALILVLAACGSNSSEQHSKGHGKLKVVTTNSIIYDMVKQVGGDKVDVHSIV
PVGQDPHEYEVKPKDIKQLTDADLIFYNGFNLESGNGWFEKALEQAGKSKDDKSVFAVSK
GVKPIYLNGETGNKDKIDPHAWLSLDNGIQYVKNIQEQLVKADASHKADYHKQGDAYLAQ
LEKLNKDSKDKFNDIPKEERAMITSEGAFKYFSKAFDIKPGYIWEINTEKQGTPSQMKQA
IQFVKDNHIKHLLVETSVDKKSMNSLSEETGKSIYGEVFTDSIGEKGSKGDSYYKMMKHN
IKTIHGSME
>ACAKHAOO_00183 Putative glycosyltransferase EpsH
MAKLVSIIVSVYNKEQFLEKCIESLIDLKMDKNNIEAIFVDDCSTDESVNIIKHYEKDYD
FIKLIQLPENTGSPSEPRNIGMREAQGKYITLLDADDWLDKEGFPQVIEKVNKDDADLGF
GQSFKHKSKNVKYHARFTSYKEASHLKPQDISKIFRAVGPPGKVFKRSLVMDHHIEFEHM
KYGEDKLFFFQLFGKVDDITMSTIPMYHVNRYDENKSLVQQTSMLDKANSNLEVLDRTCH
MDMSSELKHMALARMVEVDFISRFLRTKTFIKSADKEKFYAVIEKVERKIKEQGIDINTL
ITNPVFKQIYTLYHHADESVFVNFTKDVVNDQWRYIIKDGIVFRDFVHQYDMIKPTVVDC
YPVYEGTQMMGENKYEVIRVMKPDDISIQSVSLIEINNAANEYEVTYKYSDDKIYVPHEE
FEKLNKDININFNVNYSESGRSLVYASYPSFNDVFKMKRQNFKVEFVHKKNENKTQQVTN
RKSEYFTRIMNPMMTLKKIKIYKDVSFKEEVGSLEAGTRVEASDIQYTTKGTPRLVLDDG
SIITANKDFITLINTSGLNKYITEVPKKVKVIKACKLYDSRDFKDNTVRKLKKGDVLPIR
DIIYTTNSTPRLVTQEGLFLTANKDFIKVIK
>ACAKHAOO_00208 hypothetical protein
MKTNIRTFSSKKLVLGIVTTVIGISTIALVSENAYASTSTVKSQIVKNTTKPTTTVKTVK
PVAVKNTTTKVATNVTIKKSKATVKPIVQKAPTTTKKPSTTTKAPTTTKKPSTTTKAPTT
ATKKPSTTTKTPTIATKKPSATTKAPVTTAKKPSTTTKAPTITAKKPSTTTKAPTITTPK
PSTTTKASATTAKKPSITTKAPTTTAKKPSMTTKAPTTTTSPTTKPNASSTGNATRKQDY
YKSMTELYNDTTEGIDWKKDTRNVGKSVLIVAPHGGNLEQGTSELTKLVANNGDFDYFSF
EAIRPSNNTQLHVTSTNYDDATLHDMIQDRTATISIHGARGEEQLVYLGGYQSSLRDAIQ
SQLELKGFIVKIPPKYLGGLSNNNFINKVEESTGVQLELTTALRKAFFKNEDTSTVSRKK
>ACAKHAOO_00228 Bifunctional protein PaaZ
MKYDDFIVGETFKTKSLHITEEEIIQFATTFDPQYMHIDKEKAEQSRFKGIIASGMHTLS
ISFKLWVEEGKYGEEVVAGTQMNNVKFIKPVYPGNTLYVIAEITNKKSIKKENGLVTVSL
STYNENEEIVFKGEVTALINNS
>ACAKHAOO_00229 Beta-lactam-inducible penicillin-binding protein
MKKIKIVPLILIVVVVGFGIYFYASKDKEINNTIDAIEDKNFKQVYKDSSYISKSDNGEV
EMTERPIKIYNSLGVKDINIQDRKIKKVSKNKKRVDAQYKIKTNYGNIDRNVQFNFVKED
GMWKLDWDHSVIIPGMQKDQSIHIENLKSERGKILDRNNVELANTGTAYEIGIVPKNVSK
KDYKAIAKELSISEDYIKQQMDQNWVQDDTFVPLKTVKKMDEYLSDFAKKFHLTTNETES
RNYPLGKATSHLLGYVGPINSEELKQKEYKGYKDDAVIGKKGLEKLYDKKLQHEDGYRVT
IVDDNSNTIAHTLIEKKKKDGKDIQLTIDAKVQKSIYNNMKNDYGSGTAIHPQTGELLAL
VSTPSYDVYPFMYGMSNEEYNKLTEDKKEPLLNKFQITTSPGSTQKILTAMIGLNNKTLD
DKTSYKIDGKGWQKDKSWGGYNVTRYEVVNGNIDLKQAIESSDNIFFARVALELGSKKFE
KGMKKLGVGEDIPSDYPFYNAQISNKNLDNEILLADSGYGQGEILINPVQILSIYSALEN
NGNINAPHLLKDTKNKVWKKNIISKENINLLTDGMQQVVNKTHKEDIYRSYANLIGKSGT
AELKMKQGETGRQIGWFISYDKDNPNMMMAINVKDVQDKGMASYNAKISGKVYDELYENG
NKKYDIDE
>ACAKHAOO_00248 hypothetical protein
MNKIEVYKFVKVKQLVYQLIKLYRTNDMNSHKTQKDFLLNEINDIFKEKDIDISDFITSI
DDVKLTKKKAEHLLNELKVYIQDFEIPSSSQLEKIFRKVKKLKRPDINLIDTKEISYLGW
NDNSSNRKYIVYKNLDDKFEGIYGEISPNKVKGFCKICNQESDTSLFLNKTKHNKSSGTY

TKKGDYICYDSFKCNQNLDDINNLYEFIVKIK
>ACAKHAOO_00250 hypothetical protein
MSVLETKLKSQMSKSAKIARNMNKLPDEIDRLRKRIERINKKRKPTSSNIRDLEKSNKQL
VTKQQKLADLQVEYTKIEKKINETKINLQKEQSRNQKKLSSMLDKNTKGNEEIMEKLLTN
SDQINEISNQIKKAVNQKEIIEYDVFLSHSSLDKEDYVSKISEKLIEKGLKVFEDVKVFE
IGKSQTETMNMGILNSRFVVVFLSPNFIESGWSRYEFLSFLNREINEEHVIILPIWHKVS
VEDVRAYNPYLVDKYALNTSDFSIEEIVEKIYQVIVNSKN
>ACAKHAOO_00263 hypothetical protein
MKTAAIYLNEGNESQFGVPLEEQLEILTKIAKEKNYHIEKVYTDNEEDFIVGYYPKLNEL
NGKIYNTDYDLVLFYKSFNMFHEIYDYLKQIEFCINSHVDVYSHMEFIDSTAGSEGLKFI
HGLIATDDCL
>ACAKHAOO_00266 putative type I restriction enzymeP M protein
MSTTEKQRQQQAELQKKLWSIANDLRGNMDASEFRNYILGLIFYRFLSEKTEEEVAELLK
EDNISYAEAWEDEEYREALQQELINLIGFVIEPQDLFSHLIQKIETQTFEIEDLHKAINK
IEESTRGEDSEEDFDHLFADMDLNATRLGNTNAARTKLISKVMVNLATLPFVHSDIEIDM
LGDAYEYLIGQFAANAGKKAGEFYTPQQVSKILAKIVTTNKPNLKNVYDPTCGSGSLLLR
VGREADVRFYYGQEYNNTTFNLARMNMLLHDVNYTRFKIDNDDTLENPAFRGEKFDAVVA
NPPYSAKWSADPSFLDDERFSGYGKLAPKSKADFAFIQHMIHYLDDNGTMAVVLPHGVLF
RGAAEGTIRKYLIEEKNYLDAVIGLPANLFFGTSIPTSILVFKKCREDSDNVLFIDASQS
FEKGKNQNLLTDEDVDKIVETYRNRETIDKFSYVATLDEIKDNDYNLNIPRYVDTFEEEE
PIDLDLVQQQLTDIDKEITDVESEINDYLKELGVLKND
>ACAKHAOO_00273 Sensor protein kinase WalK
MKWLKQLQSLHTKLVIVYVLLIIIGMQIIGLYFTNSLEKELTNNFMKNIKQYATQLEVNI
ERIYRDDPSNAQKEVQSLLNEYANRQEIEEIRFIDKDQIIMATAKISSHNMINQKVNDNS
VQKALSLGESNSHNVLKDYGSGKERIWIYNLPVKNGNETIGNIYIESNINDVYNQLNNIN
QIFIIGTAISLFITVILGFFIARTITRPITDMRNQTVEMSKGNYTQRVKIYGNDEIGELA
LAFNNLSKRVQEAQANTESEKRRLDSVITHMSDGIIATDRRGRVRIVNDMAIKMLGMSKE
DLIGYYMLSVLNLEDEFSLDEIQENNDSFLLDINEDEGIIARVNFSTIVQETGFVTGYIA
VLHDVTEQQQVERERREFVANVSHELRTPLTSMNSYIEALEEGVWKDDNLAPSFLSVTRE
ETERMIRLVNDLLQLSKMDNESEQITKEIVDFNMFINKIINRHEMAAKDTTFVREIPSET
IFTEIDPDKMTQVFDNVITNAMKYSRGEKRVEFHVKQNALYNRMTIRIKDNGIGIPINKV
DKIFDRFYRVDKARTRKMGGTGLGLAISKEIVEAHNGRIWANSVEGQGTSIFITLPCEVI
DDGDWDEE
>ACAKHAOO_00274 Transcriptional regulatory protein WalR
MARKVVVVDDEKPIADILEFNLKKEGYDVYCAYDGNDAVDLIYEEEPDIVLLDIMLPGRD
GMEVCREVRKKFEMPIIMLTAKDSEIDKVLGLELGADDYVTKPFSTRELIARVKANLRRH
YSQPAQEVSGTTNEITIKDIVIYPDAYSIKKRGEDIELTHREFELFHYLSKHMGQVMTRE
HLLQTVWGYDYFGDVRTVDVTIRRLREKIEDDPSHPEYIVTRRGVGYFLQQHD
>ACAKHAOO_00279 50S ribosomal protein L9
MKVIFTQDVKGKGKKGEIKDVPVGYANNFLIKNNYAVEATPGNLKQLEQQNKRAEADRQK
EIDDAKALKAKLEEIEVEVSAKTGEGGKLFGSVSTKQIAEALKTQHDIKIDKRKMDLPQG
IHALGYTNVPVKLDKEVEGTIRVHTVEK
>ACAKHAOO_00280 Cyclic-di-AMP phosphodiesterase GdpP
MNRQSTKKALLIPFILMVLTSIALVVVWFIFKPLVATIAAAILVVMIIISIVLVRQALLK
MDNYVDNLSGHISAGSNRAIKRLPIGMVVLDADDYIEWINQYMSEHLETNVISEPVNEVF
PNILKQLEKVQEVEIEHGQYHYHVRHSEEESCLYFFDITDEVQTNELYEESKPIIATLFL
DNYDEITQNMNDTQRSEINSMVTRVISRWASEYNIYFKRYNSDQFVAYLNQKILAEIEES
NFEILSQLREKSVGYRAQLTLSIGVGEGTENLIDLGELSQSGLDLALGRGGDQVAIKNMN
GNVRFYGGKTDPMEKRTRVRARVISHALKDILTEGDKVIIMGHKRPDLDAIGAAIGVSRF
ALMNNLEAYVVLNEEDIDPTLRRVMDEIDKKPELKERFVTSDEAWDMMTSKSTVVVVDTH
KPEMVLDENILNKANRKVVIDHHRRGESFISNPLLVYMEPYASSTAELVTELLEYQPTEQ
RLTRLESTVMYAGIIVDTRNFTLRTGSRTFDAASYLRAHGADTILTQHFLKDDVDTYINR
SELIRTVEVQDNGIAIAHGSNEKIYHPVTVAQAADELLSLEGIEASYVVAKREDNLIGIS
ARSLGSINVQLTMEALGGGGHLTNAATQLKGLSIEEAIEQLQQAITEQMSRSEDA

```
>ACAKHAOO_00285 Serine--tRNA ligase
MLDIRLFREQADTVKSKIELRGDDPKVVDEVVELDNERRQLIGKTEEMKARRNKVSEEIA
EKKRNKENADDVIKEMRELGDEIKENDAKLNEVDNKVRDILIRIPNLIAEDVPQGDSDEE
NVEVKKWGTPREFDFEPKAHWDLVEELKMADFERAAKISGARFVYLTKDGALLERALMNY
MLTKHTTQHGYTEMMTPQLVNADTMFGTGQLPKFEEDLFKVEKEGLYTIPTAEVPLTNFY
RDEIIQPGVLPEKFTAQTACFRSEAGSAGRDTRGLIRLHQFDKVEMVRIEKPEDSWDALE
DMTQNAEAILEELGLPYRRVILCTGDIGFSASKTYDLEVWLPSYDNYKEISSCSNCTDFQ
ARRANIRFKRDAASKPELAHTLNGSGLAVGRTFAAIVENYQNADGSITIPEALVPFMGGK
TEIRPVNG
>ACAKHAOO_00287 DNA gyrase subunit A
MADLPQSRINERNITSEMRESFLDYAMSVIVSRALPDVRDGLKPVHRRILYGLNEQGMTP
DKPYKKSARIVGDVMGKYHPHGDLSIYDAMVRMAQTFSYRYPLVDGQGNFGSMDGDGAAA
MRYTEAKMTKITLELLRDINKDTIDFLDNYDGTEREPEVLPSRFPNLLVNGASGIAVGMA
TNIPPHNLTEVINGVLHLSKNPDVTIAELMEDIQGPDFPTAGLILGKSGIRRAYETGRGS
IQMRARAEIEERGGGRQRIVVTQIPYQVNKARMIEKIAELVREKKIEGITDLRDETSLRT
GVRVVIDVRKDANANVILNNLYKQTPLQTSFGVNMIALVNGRPKLINLKQALVEYLEHQK
TVVRRRTEYNLKKAKDRAHILEGLRIALDHIDEIITTIRESETDKVAMETLQERFKLSER
QAQAILDMRLRRLTGLERDKIENEYNELLGYISELEEILADDEVLLQLVRDELTDIKERF
GDERRTEIQLGGLDDLEDEDLIPEEQIVITLSHNNYIKRLPVSTYRSQHRGGRGVQGMNT
LEEDFVSQLVTLSTHDHVLFFTNKGRVYKLKGYEVPELSRQSKGIPVINAIELDNDETIS
TMIAVRNLEDEDSYLVFATKNGIVKRSALSNFSHINKNGKIAIGFKEDDELIAVRLTDGN
QDILIGTSHASLIRFAETSLRPLGRTAAGVRGISLREDDVVVGLDVAHADSEDEVLVVTE
NGYGKRTPVSEYRLSNRGGKGIKTAKITERNGNIVCITTVNGEEDLMIVTNSGVIIRLEV
EDISQNGRATQGVRLMKLGDDQFVSTVAKVIDKTEDEDATEAAQTENGESNAESTESPSE
EVIDDDTPGNALHTEADEETTVSSEDDSTDERIEVRQDFMDRVNEDIENASNNDEDDEN
>ACAKHAOO_00288 DNA gyrase subunit B
MVNTLSDVNNTDNYGAGQIQVLEGLEAVRKRPGMYIGSTSERGLHHLVWEIVDNSIDEAL
AGYANQIEVIIEKDNWIKVTDNGRGIPVDIQEKMGRPAVEVILTVLHAGGKFGGGGYKVS
GGLHGVGSSVVNALSEDLEVYVHRNDTIYHQAYKKGVPQFDLKEVGSTDKTGTVIRFKAD
GEIFTETTVYNYETLQKRIRELAFLNKGISITLRDEREDVEQREDTYHYEGGIKSYVELL
NENKEPIHEEPIYIHQTKDDVEIEIAMQYNSGYATNLLSYANNIHTYEGGTHEDGFKRAL
TRILNSYGTQSKIIKDDKERLSGEDTREGLTAVVSIKHGDPQFEGQTKTKLGNSEVRQIV
DRLFAEHFERFLYEHPNVARIVVEKGIMASRARVAAKKAREVTRRKSALEISSLPGKLAD
CSSKKPEESEIFLVEGDSAGGSTKEGRDSKTQAILPLRGKILNVEKARLDRILNNNEIRQ
MITAFGTGIGGEFDISKARYHKIVIMTDADVDGAHIRTLLLTFFYRFMRPLIEAGYVYIA
QPPLYKLAQGKQKYYVFNDRELDKLKSELSPTPKWQISRYKGLGEMNADQLWETTMNPEN
RMMLQVKLEDAIEADQTFEMLMGDVVENRRQFIEDNAVYANLDF
>ACAKHAOO_00291 Beta sliding clamp
MMEFTIRRDYFINQLNDTLKAISPRTTLPILTGIKIDAKDNEVILTGSDSEISIEITIPK
QVDGEDIVTISETGSVVLPGRFFVDIIKKLPGKDVKLSTNEQFQTLITSGHSEFNLSGLD
PDQYPLLPQVSRDDAIQLSVKVLKNIIAQTNFAVSTSETRPVLTGVNWLIQDNELICTAT
DSHRLAVRKLKLEDDSENKNVIIPGKALSELNKIMSDSDEEIDIFFASNQVLFKVGNVNF
ISRLLEGHYPDTTRLFPENYEIKLGLDNGEFYHAIDRASLLAREGGNNVIKLSTGNDVVE
LSSTSPEIGTVKEEVTATDVEGGNLKISFNSRYMMDALKAIDNDEVEVEFFGTMKPFILK
PKEDDSVTQLILPIRTY
>ACAKHAOO_00292 Chromosomal replication initiator protein DnaA
MSEQEIWKKVLEVAESEISKSTFNTFLKDTELKEIRDNVAIIFVIHEFYAEWLNSNYKEV
IQTIMKDVIGYEVEPKFFTAEQLAELDETSRKSNTPSEPQRQIIEYGHEGTDQFNTHNTF
DTFVIGPGNRFPHAASLAVAEAPAQAYNPLFIYGGVGLGKTHLMHAIGHHVLSNQPNAKV
LYTSSEKFTNDFIKSIRNNEPEAFREKYRNIDVLLIDDIQFIQNKEQTQEEFFHTFNELH
QNKKQIVISSDRPPKEIAKLEDRLRSRFEWGLIVDITPPDYETRMAILQKKIEEENLEIP
AEALNYIANQIQSNIRELEGALTRLLAYSKLQGRPITTELAAEALKDIIQVPKSKKITIQ
DIQKVVGHYYNVRIEDFSAKKRTKSIAYPRQIAMYLSRELTDFSLPKIGEEFGGRDHTTV
IHAHEKIAKDIKADTIFKQEVEDLEKEIRNQ
```

>ACAKHAOO_00295 tRNA modification GTPase MnmE
MDFDTITSISTPMGEGAIGIVRLSGPQAVEIGDKLYKGKKKLEDVDSHTINYGHIVDPET
NEVVEEVMISVLRAPRTFTREDIIEINCHGGILTINRILELTMTHGARMAEPGEYTKRAF
LNGRIDLSQAEAVMDFIRSKTDRASKVAMNQIEGRLSDLIKRQRQSILEILAQVEVNIDY
PEYDDVEDATTEFLLEQSKKIKNEINLLLETGAQGKIMREGLSTVIVGKPNVGKSSMLNN
LIQDNKAIVTEVAGTTRDVLEEYVNVRGVPLRLVDTAGIRDTEDIVEKIGVERSRKALSE
ADLILFVLNNNEPLTQEDRTLYEVIKNEDAIVIVNKTDLEQNLDINEVKEMIGDTPLIQT
SMLKQEGIDQLELQIRDLFFGGDVQNQDMTYVSNSRHISLLKQARNAIQDAIDAAESGIP
MDMVQIDLTRTWELLGEIIGESASDELIDQLFSQFCLGK
>ACAKHAOO_00323 Small-conductance mechanosensitive channel
MNQVKNVLSSLFEPLTKIETYENLSIKIALIIIYIIVALIVIAILNKVIEQAFKIQNRSS
KGNKKRSRTLISLVQNVVSYIVWFIVITTILSKFGISVEGIIASAGVVGLAVGFGAQTIV
KDIITGFFIIFENQFDVGDYVKINSGGTTVAEGTVKSIGLRSTRINTITGELTILPNGSM
GEITNFSITNGFSIVEIPVSVEENLDKVEKRLNKLFTAMRSKYYLFITDPVVEGIDSLDE
TKVTFRISAETIPGEGVSGSRILRKEIQRVFVQEGIKLPQPIYIKNNNQQGKS
>ACAKHAOO_00325 Ribosome-binding ATPase YchF
MALTAGIVGLPNVGKSTLFNAITKAGALAANYPFATIDPNVGIVEVPDSRLNKLTEMVKP
KKTIPTTFEFTDIAGIVKGASKGEGLGNKFLSHIREVDAICQVVRAFDDENVTHVSGRVN
PLDDIEVINMELVLADLESVEKRLPRIEKMARQKDKTAEMEMRILSRIKEALENGDPVRS
LDFNEEDQRYVNQAQLLTSKKMLYIANVGEDEIGDEDNDKVKAIREYAAKEDSEVIVISA
KIEEEIATLDDEDREMFLEDLGIEEPGLDVLIRTTYDLLGLSTYFTAGVQEVRAWTFRQG
MTAPQCAGIIHTDFERGFIRAEVTSYDDYVEHGGEQGAKEAGKQRLEGKEYIMQDGDIVH
FRFNV
>ACAKHAOO_00328 30S ribosomal protein S6
MRTYEIMYVVRPNIEEDAKKAVVERFNGILASEGSEVLEEKDWGKRRLAYEIEDFKEGFY
NIVRIKTDNNRATDEFQRLAKISDDIIRYIVIREDQDK
>ACAKHAOO_00329 Single-stranded DNA-binding protein A
MINRVVLVGRLTKDPEYRTTPSGVSVATFTLAVNRTFTNAQGEREADFINVVVFRRQAEN
VNNYLFKGSLAGVDGRIQSRSYENQEGRRIFVTEVVADSVQFLEPKNAQGGQRNQSNNND
FQDYGQGFGGQQSGQNTSYNNNNSSNNKQSDNPFANANGPIDISDDDLPF
>ACAKHAOO_00347 Aminoglycoside 3'-phosphotransferase
MAKMRISPELKKLIEKYRCVKDTEGMSPAKVYKLVGENENLYLKMTDSRYKGTTYDVERE
KDMMLWLEGKLPVPKVLHFERHDGWSNLLMSEADGVLCSEEYEDEQSPEKIIELYAECIR
LFHSIDISDCPYTNSLDSRLAELDYLLNNDLADVDCENWEEDTPFKDPRELYDFLKTEKP
EEELVFSHGDLGDSNIFVKDGKVSGFIDLGRSGRADKWYDIAFCVRSIREDIGEEQYVEL
FFDLLGIKPDWEKIKYYILLDELF
>ACAKHAOO_00362 Beta-lactamase
MKKLIFLIVIALVLSACNSNSSHAKELNDLEKKYNAHIGVYALDTKSGKEVKFNSDKRFA
YASTSKAINSAILLEQVPYNKLNKKVHINKDDIVAYSPILEKYVGKDITLKALIEASMTY
SDNTANNKIIKEIGGIKKVKQRLKELGDKVTNPVRYEIELNYYSPKSKKDTSTPAAFGKT
LNKLIANGKLSKENKKFLLDLMLNNKSGDTLIKDGVPKDYKVADKSGQAITYASRNDVAF
VYPKGQSEPIVLVIFTNKDNKSDKPNDKLISETAKSVMKEF
>ACAKHAOO_00375 Alkyl hydroperoxide reductase subunit F
MLNADLKQQLQQLLGLMEGDVEFRASIGSDDKSKELKELLDEIAEMSEHITIVEKELKRT
PSFSVSKPDEEAGVTFAGIPLGHEFNSLVLAILQVSGRAPKEKQSIIDQIKGLEGKYNFE
TYVSLTCQKCPDVVQALNLMSVVNPNITHTMIDGSVFREESEDIMAVPAVFLDGEEFGNG
RMTISDILTKLGSTQDASEFEGKDPYDVLIIGGGPASGSAAIYTARKGLRTGIIADRIGG
QVNDTAGIENFITVKETTGSQFSANLAAHIEEYDIDTMTGIRATNIEKTDQAIRVTLENN
AVLETKTAIISTGASWRKLNIPGEDRLINKGVAFCPHCDGPLFENKDVAVIGGGNSGVEA
AIDLAGIVKHVTLFEYSSELKADTVLQDRLRSLSNVDIHTNARTAEVLGEDHVTGISYED
LNSGEMKELSLDGIFVQIGLVPNTSWIGDAVELNNRGEVVIDRNNNTNVPGIFAAGDVTD
QKNKQIIISMGAGANAALNAFDYIIRN
>ACAKHAOO_00376 Alkyl hydroperoxide reductase C
MSLINKEILPFIAQAYDPKKDEFKEVSQDDLKGSWSVVCFYPADFSFVCPTELEDLQNQY

DKLQDLGVNVFSVSTDTHFVHKAWHDHSDAISKIQYQMIGDPSQTITRNFDVLDEEAGLA
QRGTFIIDPDGVVQAAEINADGIGRDASTLVNKIKAAQYVRQHPGEVCPAKWEEGSESLQ
PGLDLVGKI
>ACAKHAOO_00383 Xanthine phosphoribosyltransferase
MYTNLGGFDVESLRQKVKEDGVVIDEKILKVDGFLNHQIDAKLMHEVGKTFYEQFKDKGV
TKILTIEASGIAPAIMASLHFDVPCLFAKKAKPSTLKDGFYSTDIHSFTKNKTSTVIVSE
EFLNENDTVLIIDDFLANGDASLGLYDIAQQAKAKTVGIGIVVEKSFQDGRQRLEEAGLN
VSSLCKVASLKGNQVTLLGENE
>ACAKHAOO_00385 Inosine-5'-monophosphate dehydrogenase
MWENKFAKESLTFDDVLLIPAASDVLPNDADLSVELSERIKLNIPVISAGMDTVTESKMA
IAMARQGGLGVIHKNMGIEEQAEEVQKVKRSENGVITNPFYLTPDESVYEAEALMGKYRI
SGVPIVSDKESRELVGILTNRDLRFIEDFSIKISDVMTKENLITAPVGTTLDEAETILQE
HKIEKLPLVENGRLEGLITIKDIEKVLEFPHAAKDAHGRLLAAAAIGTSKDTEIRAQKLV
EAGVDALIIDTAHGHSSGVIQEVKKMKEKYPEITIVAGNVATAEATRALFEAGADVVKVG
IGPGSICTTRVVAGVGVPQITAIYDCATEARKFGKAIIADGGIKFSGDIIKALAAGGHAV
MLGSLLAGTEESPGATEVFQGRQYKVYRGMGSLGAMEKGSNDRYFQEDKTPRKFVPEGIE
GRTAYKGPLQDTIYQLMGGVRAGMGYTGSPDLKTLRDEAQFTRMGPAGLAESHPHNVQIT
KESPNYSF
>ACAKHAOO_00386 GMP synthase [glutamine-hydrolyzing]
MEMAQEQELILVLDFGSQYNQLITRRIREMGVYSELHDHEISVDEIKRMNPKGIILSGGP
NSVYEEGSFTIDPEIYNLGIPVLGICYGMQLTTKLLGGKVERANEREYGKAIINAKSDEL
FFGLPAEQTVWMSHSDKVIEIPDGFEVIADSPSTNYAAIEDKKRRIYGVQFHPEVRHTEY
GNDLLRNFVRRVCECTGEWTMENFIDIEVEKIRKRVGDRKVLCAMSGGVDSSVVAVLLHK
AIGDQLTCIFVDHGLLRKGEGDMVMEQFGEGFDMNIIRVNAQERFMDKLKGVSDPEQKRK
IIGNEFVYVFDDEAAKLTDVDFLAQGTLYTDVIESGTKTAQTIKSHHNVGGLPEDMEFEL
IEPINTLFKDEVRALGIELGIPEHLVWRQPFPGPGLGIRVLGEITEDKLEIVRESDAILR
EVVREEGLERDIWQYFTVLPGIQSVGVMGDYRTYDHTVGIRAVTSIDGMTSDFARIDWEV
LQKISSRIVNEVDHVNRVVYDITSKPPSTIEWE
>ACAKHAOO_00412 Cystathionine gamma-lyase
MKKKTQMIHGGHTTDDFTGAVTTPIYQTSTYLQDEIGDLRQGYEYSRTANPTRTAIESVI
ANLENGKHGFAFGSGMAAISAVIMLLDKGDHLILNSDVYGGTYRALTKVFTRYGIDVDFV
DTTHIENVKDYIKPETKMLYVETPSNPLLRVTDIKRTVEIAKENNLISVVDNTFMTPYYQ
NPLDFGIDIVLHSATKYIGGHSDVVAGLVATADDELGERLAFISNSTGGVLGPQDSYLLV
RGIKTLGLRMEQINRNVEAIVEMLQNHPSVQQVFHPSIKDHLNHDIHVAQASGHTGVVAF
EVKDTEAAKQLIRETNYFTLAESLGAVESLISVPALMTHASIPADVRAKEGITDGLVRLS
IGIEDTDDLVEDLKQAIDTLN
>ACAKHAOO_00420 Glutamate synthase [NADPH] large chain
MFDERLKRGLYDYREEHDACGIGFYANMNNLRSHDIIEKSLEMLRRLDHRGGVGADGITG
DGAGIMTEIPFQFFKAHVNLDLPNEGDYAVGLFFSNEKIQGTKHETQFNDYFQSEGLKVI
GYRDVPVDVNAVAPHVASTMPHIQQVFIDIRLIETPAKPLYIARKQIEQYAERQDLGLYF
TSLSHKTIVYKGWLRSDQIKKLYLDLNHEDYQSKLGLVHSRFSTNTFPSWKRAHPNRLLM
HNGEINTIKGNVNWMRARQRQLIQTLFGEEHTKIQNIVDEDGSDSAIVDNALEFLSLAME
PEEAAMLLIPEPWLYNKENDDNVRAFYEFYSYLMEPWDGPTMISFCNGDKIGALTDRNGL
RPGRYTITKDNFIVFSSEVGVVDVPEVNVAYKGQLNPGKLLLVDFDSHKVVENNELKARI
ANQYPYKEWLDNHKVDLNLEGQTYHQPLLNSETLFELQRQFGYTKEDIYKYMAELVKGKK
DPIGAMGYDAPLAVLNERPESLFNYFKQLFAQVTNPPIDAYREKIVTSELSYLGGEGNLF
HPDETVLDRIQLAKPVLTLTQLDKIKQSKFNVKHLSTLYQDNLKEALERLGEAAIQATRD
GHTILVLDDSELIDNDDESKLKEQRYAMPILLAISHIHQLLIREGLRMDTSLVALSGETR
EVHHVACLLGYGANAVIPYLAQHTIAHLTETHRLEGTVSDNVETYTRVLSEGVIKVMAKM
GISTVQSYQGAQIFEAVGLSQEVIDDYFTGTQSKLSGLSIQQIDTENKMRQQRHNEYLDS
GSVFQWRQQGQHHVFNPTTIHLLQHACRENDYTQFKTFSNAIHEQRNDHLRDLLEFKAQQ
SIHISEVESVESIVKRFNTGAMSYGSISEEAHQTLAQAMNKIGGKSNSGEGGENPKRYEI
QEDGTVLSSAIKQVASGRFGVTSNYLQHAKELQIKVAQGAKPGEGGQLPGTKVYPWIAET
RGSTPGIGLISPPPHHDIYSIEDLGQLIHDLKNANRSADVAVKLVSKTGIGTIASGVAKA

FADKIVVSGYDGGTGASPKTSIQHAGLPWELGLAETHQTLKLNHLRSRVRLETDGKLMTG
RDVALACALGAEEFGFATAPLVVLGCIMMRVCHNDTCPVGIATQNKDLRALFRGKADHVV
NFMHFIAEELREVLASLGIRTVEELVGRTDLLQRSSRIDEQSKAATLDIEKLLDSDDGPN
SKEINQNHHLDIGFDLNYLYKDAKQSIEDGKLFKGNYVINNEQRDVGVITGSYITQCHGI
KGLPEGTIVAKTHGHAGQSLAAFAPSGLLVQHTGDANDYVGKGLSGGTVVIKAPNKQRER
EIIAGNVCFYGASKGKAFINGSAGERFCIRNSGVDVVVEGIGDHGLEYMTGGHVVNLGDV
GKNFGQGMSGGVCYVFPSNLELFKRKNQLPTLAFERVTHKEEKALLKTMLEEHYRYTQST
KAASILNDFEHVADKVIKVIPKDYELMMQKIELQSRHLPQLDDAKLAAFYDERTTIEQEL
QPAVIY
>ACAKHAOO_00443 Methionine--tRNA ligase
MAKETFYITTPIYYPSGNLHIGHAYSTVAGDVISRYKRMQGYDVRYLTGTDEHGQKIQEK
AQKAGKTELEYLDEMISGIKSLWSKLEISNDDFIRTTEDRHKQVVEKVFERLLKQGDIYL
GEYEGWYSVPDETYYTESQLVDPIYENGKIVGGKSPDSGHEVELVKEESYFFNINKYTDR
LLEFYDANPDFIQPPSRKNEMINNFIKPGLEDLAVSRTSFDWGVRVPSNPKHVVYVWIDA
LVNYISALGYLSDDDELFQKYWPADVHLMAKEIVRFHSIIWPILLMALDLPLPKKVFAHG
WILMKDGKMSKSKGNVVDPNVLIDRYGLDATRYYLMRELPFGSDGVFTPEAFVERTNYDL
ANDLGNLVNRTISMINKYFQGELPAYEGPKHELDEDMEALAHETVKHFNESMESFQFSVA
LSTVWKFISRTNKYIDETTPWVLAKDDSQKDMLGNVMAHLVENIRFAAVLLRPFLTHAPK
EIFKQLNINEPELFELESLEQYGALKQPIMVTEKPTPIFPRLDTEAEIAYIKESMQPPKS
EESKDEVEEPSKAQIDIKDFDKVEIKAATITDAENVPKSDKLLKIQIDLGLEQRQIVSGI
AKFYRPEDIIGKKVAVVTNLKPAKLMGQKSEGMILSAEKDGVLTLVSLPSAIPNGAVIK
>ACAKHAOO_00450 2-iminobutanoate/2-iminopropanoate deaminase
MKTINTNKAPEALGPYSHAMVVNNLVFTSGQIPLDTEGNIVSDDVKEQTKQVLDNLSVVL
EEAGSDLNSVVKATIFISDMNEFQQINEVYGSYFSEHQPARSCVEVARLPKDVKVEIEVV
GKVKGI
>ACAKHAOO_00451 Putative septation protein SpoVG
MKVTDVRLRKIQTDGRMKALVSITLDEAFVIHDLRVIEGNSGLFVAMPSKRTPDGEFRDI
AHPINSDMRQEIQDAVMKVYDETDEVIPDKNASSEDSDEA
>ACAKHAOO_00453 Ribose-phosphate pyrophosphokinase
MLNNEYKNSSLKIFSLKGNEPLAQEVADRVGIELGKCSVKRFSDGEIQINIEESIRGCDV
FIIQPTSNPVNLHLMELLIMIDACKRASAANINIVVPYYGYARQDRKARSREPITAKLVA
NLIETAGANRMIALDLHAPQIQGFFDIPIDHLMGVPIIGQHFENDPNIDPEECVVVSPDH
GGVTRARKLADILKTPIAIIDKRRPKPNVAEVMNIVGEIEGRTAIIIDDIIDTAGTITLA
AQALKDKGAKDVYACCTHPVLSGPAKERIENSAIKELIVTNSILLDDTRKPSNTKELSVA
GLLAQAIIRVYERESVSVLFD
>ACAKHAOO_00454 50S ribosomal protein L25
MASLKSIIRQGKQTRSDLKKLRNTGKVPAVVYGYGTKNTSVKVDEVEFIKVIREVGRNGV
IDLGVGSKSIKVMVSDYQFDPLKNQITHIDFLAINMTEERTVDVPVHLVGEAAGAKEGGV
VEQPLFDLQVTATPENIPESIEVDITELEVNDSYSVSDIKVSGDFTIENDPEESVVTVVP
PTDEPTEEEVEAMEGEAATEEPEVVGEEKEEDSEEENKDEE
>ACAKHAOO_00459 putative protein
MRLDKYLKVSRLVKRRTLAKEISDQGRITINGNVAKAGTDVKVEDELVIRFGQKLVTVKV
TGLSEHASKENAKGMYDLIKEERINQNQFEEE
>ACAKHAOO_00463 Hypoxanthine-guanine phosphoribosyltransferase
MKDDLKEILLTEEDIQNICKDLGEQLTKDYKDKPLVCVGILKGSVMFMADLIKRIDTHLA
IDFMDVSSYHGGTESTGEVQILKDLGSSIENKDVLIIEDILETGTTLKSITELLQSRKVN
SLEIVTLLDKPNRRKADIEAKYVGKKIPDEFVVGYGLDYAEHYRNLPYIGTLKPEIYNK
>ACAKHAOO_00464 ATP-dependent zinc metalloprotease FtsH
MQKAFRNVLVIAIIGVIIFGVFSYINGNGNTPKQLSYSQFVEKLDKGDLKTLEIQPEQNV
YLVSGKTKNDEDYSSTILYNNEKDLQKITNEAKSQKGLDFKVKEEEKQSVFVSMLTTLIP
VLIIAFLFIFFLSQAQGGGGGGRMMNFGKSKAKMYDNQKRRVRFSDVAGADEEKQELIEI
VDFLKDNKKFKQMGSRIPKGVLLVGPPGTGKTLLARAVAGEAGAPFFSISGSDFVEMFVG
VGASRVRDLFENAKKNAPCIIFIDEIDAVGRQRGAGVGGGHDEREQTLNQLLVEMDGFGE
NEGIIMIAATNRPDILDPALLRPGRFDRQIQVGRPDVKGREAILHVHSKNKPLDETVDLK

AISQRTPGFSGADLENLLNEASLIAAREGKNKIDMRDIEEATDRVIAGPAKKSRVISDKE
RNIVAHHEAGHTVIGMVLDEAEVVHKVTIVPRGQAGGYAMMLPKQDRFLMTEPELLDKIC
GLLGGRVSEDINFHEVSTGASNDFERATQIARSMVTEYGMSKKLGPLQFSSSGGGQVFLG
KDMQGEPNYSGQIAYEIDKEVQRIIKEQYERCKQILLDHEKELKLIAKTLLTEETLVAEQ
IRSLFYDGVLPEVDYDAARVVKDEDSDYSEGKYGKSYDDIREEQLEEGKEDMREDRKEDN
DMNRERRHRQRDDRDNQTGHDQLRDGSNGDNDQHRGHSNNEEDTGHEQSPNIDKPYNPND
PNNPSNR
>ACAKHAOO_00465 33 kDa chaperonin
MTHDYIVKSLAFDGEIRAYAALTTESVQEAQTRHYTWPTASAAMGRTMTATLLMGSMLKG
EQKLTVTVDGKGPIGRIIADADAEGNVRAYVDKPQTHFPLNEQGKLDVRRAVGTDGSIQV
VKDVGMKDYFSGASPIVSGELGEDFTYYFATSEQTPSSVGLGVLVNPDNSIKAAGGFIIQ
VMPGAKDETVTKLENAINNMQPVSKLIEQGLTPEGILNEILGEDNVQILETMQAQFECNC
SHEKFLNAIKGLGEAEIQDMIKEDHGAEAICHFCGNKYNYSEAELEDLLASMNA
>ACAKHAOO_00466 Cysteine synthase
MAQKPVENITQIIGNTPVVKLRNVVDEDAADVYVKLEYQNPGGSVKDRIALAMIEKAEKE
GKIKPGDTIVEPTSGNTGIGLAFVCAAKGYKAVFTMPETMSSERRNLLKAYGAELVLTPG
SEAMKGAIKKAKELKEEHGYFEPQQFENPANPEVHALTTGPELVEQFEGKQIDAFLAGVG
TGGTLSGVGKVLKEKYPNVEIVAIEPEGSPVLSGGEPGPHKLQGLGAGFVPDTLNTNIYD
SIIKVGNETAMETSRRVAKEEGILAGISSGAAIYAAIEKAKELGKGKTVVTVLPSNGERY
LSTPLYSFDD
>ACAKHAOO_00470 Lysine--tRNA ligase
MSEEMNDQMQVRRQKLQELIDLGIDPFGKRFERSATASELKSQWDEFSKEELHDKEDESH
VSIAGRLMTKRGKGKAGFAHVQDLTGQIQIYVRKDQIGEEDFDNLWKGADLGDIVGIEGV
MFKTNTGELSVKAKTFTLLSKALRPLPDKFHGLQDIEQRYRQRYLDLITNQDSTQTFINR
SKIIQEMRNYLNKQGFLEVETPMMHQIAGGAAARPFVTHHNALDATLYMRIAIELHLKRL
IVGGLEKVYEIGRVFRNEGVSTRHNPEFTMIELYEAYADYHDIMDLTESMVRHIAQEVFG
SAKVQYNEEEIDLESAWTRLHIVDAVKEATGVDFYQIKSDEEAIAAAKEHGIEITNNMKY
GHILNEFFEQKVEETLIQPTFIYGHPIEISPLAKKNPEDPRFTDRFELFIVGREHANAFT
ELNDPIDQRERFEAQLVEKAQGNDEAHEMDEDYIEALEYGMPPTGGLGIGIDRLVMLLTD
SPSIRDVLLFPYMRQK
>ACAKHAOO_00492 ATP-dependent Clp protease ATP-binding subunit ClpC
MLFGRLTERAQRVLAHAQEEAIRLNHSNIGTEHLLLGLMKEPEGIAAKVLESFNITEDKV
IEEVEKLIGHGQDQTGTLHYTPRAKKVIELSMDEARKLHHNFVGTEHILLGLIRENEGVA
ARVFANLDLNITKARAQVVKALGSPEMSNKNATANKSNNTPTLDGLARDLTVIAKDGTLD
PVVGRDKEITRVIEVLSRRTKNNPVLIGEPGVGKTAIAEGLAQAIVNNEVPETLKGKRVM
SLDMGTVVAGTKYRGEFEERLKKVMEEIHQAGNVILFIDELHTLVGAGGAEGAIDASNIL
KPALARGELQCIGATTLDEYRKNIEKDAALERRFQPIQVDEPTVEDTIAILKGLRDRYEA
HHRINISDEALEAAAKLSDRYVSDRFLPDKAIDLIDEASSKVRLKSHTTPNNLKEIEQEI
EKVKNEKDAAVHAQEFENAANLRDKQTKLEKQYEDAKNEWKTTQGDQSTSLSKEDIGEVI
AGWTGIPLTKINETESDRLLNLEQTLHDRVIGQNDAVTSISKAVRRARAGLKDPKRPIGS
FIFLGPTGVGKTELARALAESMFGEEDAMIRVDMSEFMEKHAVSRLVGAPPGYVGHDEGG
QLTEKVRRKPYSVILFDEIEKAHPDVFNILLQVLDDGHLTDTKGRQVDFRNTVIIMTSNV
GAQELQDQRFAGFGGGSEGHDYETIRKTMMKELKNSFRPEFLNRVDDIIVFHKLSKDELK
EIVTMMVNKLTQRLSEQDINIVVTDKAKEKIAEEGYDPEYGARPLIRAIQKTVEDNLSEL
ILDGNQIEGKEVVIDHDGDEFKYNINEKNNGISDEGNEVTETEA
>ACAKHAOO_00494 putative PIN and TRAM-domain containing protein YacL
MNVLKGIVIIIYIILGAALGILLIPEILKDFNINGYPFLHNYYIDGLIGIVVFFLIFGFF
INKVTKTFKAIEHWIMQRSAVEILFATLGLIIGLFISVMASFILEMIGNTILNHLVPLII
TLLLCYLGFQFGLKKRDEMLMFLPENMARSMSKSNISAVPKLLDTSAIIDGRILDVIRCG
FIDGEILIPQGVINELQVVADANDSVKREKGQRGLDILNELYDLEYPTRVIHPQKSHSDI
DELLVKLAQTYHAHVITTDFNLNKVCHVQGIKALNVNDLSEAIKPNVHQGDRINILLTKM
GKEPGQGVGYLDDGTMVVVDNAKSLVGKHVDLEVISLLQTSSGRIIFAKKIDDSE
>ACAKHAOO_00497 Cysteine--tRNA ligase
MITLYNTLTRQKETFEPIEPGKVKMYVCGPTVYNYIHIGNARPAINYDVVRRYFEYKGYE

VIYVSNFTDVDDKLINRSKELNESVPDIADRYIKAFYEDVGALNVKKATSNPRVMNHMDE
IIDFIKELVDEGYAYESDGDVYFRTRKFEGYGKLSHQSLDDLKVGARIEAGEQKEDALDF
TLWKKAKPGEISWESPFGEGRPGWHIECSVMAFHELGKTIDIHAGGSDLQFPHHENEIAQ
SEAHNHAPFANYWMHNGFINIDNEKMSKSLGNFILVHDIIKEVDPDVLRFFMISVHYRSP
INYNLELVNAAKSGLERIRNSYQLIEERETIATDIVDQSKYIEQIDAIIEQFETVMDDDF
NTANAVTAWYDLSKLANKYVLENNTSTQVISRFKEVYQIFSDVLGVPLKGNKSDMLLDED
IEKLIEERNEARKNKDFARADEIRDMLKEQNIILEDTPQGVRFKRG
>ACAKHAOO_00499 Putative TrmH family tRNA/rRNA methyltransferase
MKTVEDIVIVGRHAVKEAIVSGHTINKIWIQEGIRKQQINDILQNAKEQKLIVQTVPKSK
LDNLANAPHQGVAALIAPYEYADFDQFIQSQKDKEGLSTVVILDGLEDPHNLGSILRTAD
ASGVDGVIIPKRRSVALTQTVAKASTGAIQHVPVMRVTNLAKTIDELKEHGYWVAGAEAD
NATDYRDMAADMPLAIVIGSEGQGMSRLVKDKCDFYIKIPMVGHVNSLNASVAASLMMYE
VYRKRHQVGDNA
>ACAKHAOO_00504 50S ribosomal protein L11
MAKKVEKVVKLQIPAGKANPAPPVGPALGQAGVNIMGFCKEFNARTQEQAGLIIPVEISV
YEDRSFTFITKTPPAPVLLKKAAGVEKGSGEPNKTKVATVTKDQVREIAQTKMQDLNAAD
EEAAMRIIEGTARSMGITVQ
>ACAKHAOO_00505 50S ribosomal protein L1
MAKKGKKYQEAANKVDRTKHYSVEEAISLAKETSIANFDASVEVAFRLGIDTRKNDQQIR
GAVVLPNGTGKSQRVLVFAKGDKITEAEEAGADYVGESEYVQKIQQGWFDFDVVVATPDM
MGEVGKLGRVLGPKGLMPNPKTGTVTMDVKKAVEEIKAGKVEYRAEKAGIVHASIGKVSF
SDEKLVENFKTLQDVLAKAKPSSAKGTYFKSVAVTTTMGPGVKIDTSSFKL
>ACAKHAOO_00507 50S ribosomal protein L10
MSAIIEAKKQQVDVIAEQLKNSVSTVIVDYRGLTVAEVTELRSQLREAGVEYKVYKNTMV
RRAAEQAGIEGLDEFLTGPTAVATSTEDVVAPAKVIAGFAKEHEALEIKTGVMEGSVISA
EEVKTVGSLPSHDGLVSMLLSVLQAPVRNFAYAVKAVGEQKEESAE
>ACAKHAOO_00508 50S ribosomal protein L7/L12
MANQEQIIEAIKEMSVLELNDLVKAIEEEFGVTAAAPVAAAGAAGGGDAAAEKTEFDVEL
TSAGSSKIKVVKAVKEATGLGLKDAKELVDNAPKVIKEGVAKEEAEKLKEQLEEVGATVE
LK
>ACAKHAOO_00510 DNA-directed RNA polymerase subunit beta
MAGQVVQYGRHRKRRNYARISEVLELPNLIEIQTKSYDWFLKEGLLEMFRDISPIEDFTG
NLSLEFVDYRLGEPKYDLEESKNRDATYAAPLRVKVRLIIKETGEVKEQEVFMGDFPLMT
DTGTFVINGAERVIVSQLVRSPSVYFNEKIDKNGRENYDATIIPNRGAWLEYETDAKDVV
YVRIDRTRKLPLTVLLRALGFSNDQEIIDLLGDSEYLRNTLEKDGTENTEQALLEIYERL
RPGEPPTVENAKSLLYSRFFDPKRYDLASVGRYKANKKLHLKHRLFNQRLAEPIVNSETG
EIVAEEGTVLDRRKLDEIMEVLETNANSEVFELEGTVIDEPVEIQSIKVYVPNDEEGRTT
TVIGNALPDSEVKCITPADIIASMSYFFNLLNGIGYTDDIDHLGNRRLRSVGELLQNQFR
IGLSRMERVVRERMSIQDTDSITPQQLINIRPVIASIKEFFGSSQLSQFMDQANPLAELT
NKRRLSALGPGGLTRERAQMEVRDVHYSHYGRMCPIETPEGPNIGLINSLSSYARVNEFG
FIETPYRKVDLDTNSITDQIDYLTADEEDSYVVAQANSRLDENGRFLDDEVVCRFRGNNT
VMAKEKMDYMDVSPKQVVSAATACIPFLENDDSNRALMGANMQRQAVPLMNTEAPFVGTG
MEHVAARDSGAAITAKYRGRVEHVESKEILVRRLVEENGTEHEGELDRYPLAKFKRSNTG
TCYNQRPIVSVGDVVEYNEILADGPSMELGEMALGRNVVVGFMTWDGYNYEDAVIMSERL
VKDDVYTSIHIEEYESEARDTKLGPEEITRDIPNVSENALKNLDDRGIVYVGAEVKDGDI
LVGKVTPKGVTELTAEERLLHAIFGEKAREVRDTSLRVPHGAGGIVLDVKVFNREEGDDT
LSPGVNQLVRVYIVQKRKIHVGDKMCGRHGNKGVISKIVPEEDMPYLPDGRPIDIMLNPL
GVPSRMNIGQVLELHLGMAAKNLGIHVASPVFDGANDDDVWSTIEEAGMARDGKTVLYDG
RTGEPFDNRISVGVMYMLKLAHMVDDKLHARSTGPYSLVTQQPLGGKAQFGGQRFGEMEV
WALEAYGAAYTLQEILTYKSDDTVGRVKTYEAIVKGENISRPSVPESFRVLMKELQSLGL
DVKVMDEQDNEIEMADVDDEDATERKVDLQQKDVPETQKETTD
>ACAKHAOO_00511 DNA-directed RNA polymerase subunit beta'
MIDVNNFHYMKIGLASPEKIRSWSYGEVKKPETINYRTLKPEKDGLFCERIFGPTKDWEC
SCGKYKRVRYKGMVCDRCGVEVTKSKVRRERMGHIELAAPVSHIWYFKGIPSRMGLLLDM

SPRALEEVIYFASYVVVDPGPTGLEKKSLLSEAEFREYYDKYPGQFVAKMGAEGIKDLLE
EINLDEELKSLRDELESATGQRLTRAIKRLEVVESFRNSGNNPAWMILDVLPIIPPEIRP
MVQLDGGRFATSDLNDLYRRVINRNNRLKRLLDLGAPGIIVQNEKRMLQEAVDALIDNGR
RGRPVTGPGNRPLKSLSHMLKGKQGRFRQNLLGKRVDYSGRSVIAVGPSLKMYQCGLPKE
MALELFKPFVMKELVQREIATNIKNAKSKIERMDDEVWDVLEDVIREHPVLLNRAPTLHR
LGIQAFEPTLVEGRAIRLHPLVTTAYNADFDGDQMAVHVPLSKEAQAEARMLMLAAQNIL
NPKDGKPVVTPSQDMVLGNYYLTLERKDAMNTGTIFNDTNEVLKAYANGYVHLHTRIGVH
AKSFNNPTFTEAQNNKILATSVGKVIFNEIIPDSFAFINEPSQTNLEGKTPDKYFIDSTQ
LGEGGLKAYFEEQELIEPFNKKFLGNIIAEVFNRFSITDTSMMLDRMKDLGFKFSSKAGI
TVGVADIVVLPDKQDILDEHEKLVERVSKQFNRGLITEDERYNAVVEIWTDAKDQIQGEL
MQSLEKTNPIFMMSDSGARGNASNFTQLAGMRGLMAAPSGKIIELPITSSFREGLTVLEY
FISTHGARKGLADTALKTADSGYLTRRLVDVAQDVIVREEDCGTDRGLLVSDIKEGTEMI
EPFIERIEGRYSKETIRHPETDEIIIRPDELITAEIAKKITDAGIEQMYIRSAFTCNTRH
GVCEKCYGKNLATGEKVEVGEAVGTIAAQSIGEPGTQLTMRTFHTGGVAGSDITQGLPRI
QEIFEARNPKGQAVITEIEGVVEDIKLAKDRQQEIVVKGANETRSYLASGTSRLKVEVGQ
SVERGEVLTEGSIEPKNFLAVAGLNATESYLLKEVQKVYRMQGVEIDDKHVEVMVRQMLR
KVRIIEAGDTKLLPGSLVDIHSFTDANREAFKERKRPATAKPVLLGITKASLETESFLSA
ASFQETTRVLTDAAIKGKRDDLLGLKENVIIGKLIPAGTGMRRYSDVKYDKAETPVTETE
EAEIIE
>ACAKHAOO_00513 30S ribosomal protein S12
MPTINQLVRKPRQSKSKKSDSPVLNRGFNSKKKQFTNLNSPQKRGVCTRVGTMTPRKPNS
ALRKYARVRLSNNIEINAYIPGIGHNLQEHSVVLVRGGRVKDLPGVRYHIVRGALDTSGV
DGRRQGRSLYGTKKPKN
>ACAKHAOO_00514 30S ribosomal protein S7
MPRKGSVPKRDVLPDPIHNSKLVTKLINKIMLDGKRGTAQRILYSAFDLVEQRSGRDALE
VFEEAINNIMPVLEVKARRVGGSNYQVPVEVRPERRTTLGLRWLVNYARLRGEKTMEDRL
ANEILDAANNTGGAVKKREDTHKMAEANKAFAHYRW
>ACAKHAOO_00515 Elongation factor G
MARDFSLKNTRNIGIMAHIDAGKTTTTERILYYTGRIHKIGETHEGASQMDWMEQEQDRG
ITITSAATTAQWDGHRVNIIDTPGHVDFTVEVERSLRVLDGAVTVLDAQSGVEPQTETVW
RQATTYGVPRIVFVNKMDKLGANFDYSVSTLHDRLDANAAPIQLPIGAEDEFEAIIDLVE
MKCFKYTNDLGTEIDEIEIPEDHKERAEEARSNLIEAVAETNDELMEKYLGDEEISVAEL
KNAIRQATTDVEFYPVLCGTAFKNKGVQLMLNAVIDYLPSPLDVKPIVGHRAENPEEEVI
AKPDDSAEFAALAFKVMTDPYVGKLTFFRVYSGTLTSGSYIKNSTKDKRERVGRLLQMHA
NSRQEIDTVYSGDIAAAVGLKDTGTGDTLCGEKNDIILESMEFPEPVIHLSVEPKSKADQ
DKMTQALVKLQEEDPTFHAHTDEETGQVIIGGMGELHLDILVDRMKKEFNVECNVGAPMV
SYRETFKQSAQVQGKFSRQSGGRGQYGDVHIEFTPNETGAGFEFENSIVGGVVPREYIPS
VEAGLKDAMENGVLAGYPLIDVKAKLFDGSYHDVDSSEMAFKIAASLALKEAAKKCDPVI
LEPMMKVTIEMPEEYMGDIMGDVTARRGRVDGMEPRGNAQVVNAYVPLSEMFGYATSLRS
NTQGRGTYTMYFDHYAEVPKSIADDIIKKNKGE
>ACAKHAOO_00516 Elongation factor Tu
MAKEKFDRSKEHANIGTIGHVDHGKTTLTAAIATVLAKNGDTVAQSYDMIDNAPEEKERG
ITINTAHIEYQTDKRHYAHVDCPGHADYVKNMITGAAQMDGGILVVSAADGPMPQTREHI
LLSRNVGVPALVVFLNKVDMVDDEELLELVEMEVRDLLSEYDFPGDDVPVIAGSALKALE
GDAQYEEKILELMQAVDDYIPTPERDSDKPFMMPVEDVFSITGRGTVATGRVERGQIKVG
EEVEIIGIHDTSKTTVTGVEMFRKLLDYAEAGDNIGALLRGVAREDVQRGQVLAAPGSIT
PHTKFKADVYVLSKDEGGRHTPFFTNYRPQFYFRTTDVTGVVNLPEGTEMVMPGDNVEMT
VELIAPIAIEDGTRFSIREGGRTVGSGVVTEIIE
>ACAKHAOO_00518 Putative pyridoxal phosphate-dependent
acyltransferase
MVQSLHNFLEENIQYLKDNGLYNEIDSIEGANGPEITINGKQYINLSSNNYLGLATNEDL
KSAAKHAIDSHGVGAGAVRSINGTLDLHDELEETLAKFKGTEAAIAYQSGFNCNMAAISA
VMNKNDAILSDELNHASIIDGCRLSKAKIIRVNHSDMDDLRAKAKEAVESGQYNKVMYIT
DGVFSMDGDVAKLPEIVEIAEEFGLITYVDDAHGSGVMGKGAGTVKHFGLQDKIDFQIGT

LSKAIGVVGGYVAGTKELIDWLKAQSRPFLFSTSLAPGDTKAITEAVKKLMTSTELHDQL
WDNAKYLKDSLEKLGFNTGESETPITPVIIGDEKETQEFSKRLKDEGVYVKSIVFPTVPR
GTGRVRNMPTAAHTKEMLDKAIAAYEKVGKELGTIK
>ACAKHAOO_00522 Mannosylglucosyl-3-phosphoglycerate phosphatase
MKRYLIPLLVLCFIVFGITTGTSYAAESQATQSTNITATEEASQTASKEASSANIESTNK
PLSLESSNVSHNEQATPSSETTTHPSVETSTTQNTTSEKHNNSSQGSAFSQEKATPEPAT
NDDNTSSQEKASSESTSNENNTSQFHGTEVKPSPSTSEQSKSTQVEPTKESTSTKVKQTP
QEPTQQQQKRNVPESQQSTPASKQVSKNSNTTSEQAAPKSQHVTVNKRMAATSDNTNEHT
ILHTNDIHGRFVEDDGRVIGMAKVKGLKDKYNPDLMVDSGDAFQGLPVSNNSKGEEMAKA
MNGVGYDAMTVGNHEFDFGYDQLLKLQKQLNFPIVSSNIYKNGKRVFDPSTTVTKNNVRY
GIVGVTTPETKTKTSPTAVEGVEFKDPLTSVKQAMNEIKNNVDVFVILSHLGVDKSTQKT
WRGDYLIDQLTKDGSFKQPIFVLDGHSHTVIDKGEHYGTNNVLAQTGTALANVGRVDFTF
QNQKASDINASLINVADAKDITPDPQIDAQTKKANDDFLKETSTVVIPNNTVTLNGERAQ
ARTQETNLGNLITDAMEAYASKNFSHQPDFAITNGGGIRASIEKGEVTKNDIITVLPFGN
LISQIQVKGSDVEKAFEHSLSADTETQDGKKVLGANGGFLQVSDSLRVYYDINKASGQRI
NAIKVLNKENGEYEDLDPNRTYYVTTNDFTANQGDGYDMFGGQREEGISLDAVVAQYIKN
TDLNQYNTEEPVRIINGLPESNEQPDTSKEDNDNNNNHSTNGHDGQQQTPDSNNDDKDNT
GHINDNDDNIKDDNEADSSNKDNVIEFPNKDHQKDNDNVIEIPANNSQTHNSTNDNIVIT
STTDKMHTNNIKGNAIEHHPTSNLVSISNNNKGTRDQLTGNASHSQHCKDVIKFPVQINP
KDNFYNETLITTNSNASKNSNLMFTQQHGAITSLPNAGLKDGSLEHGIAIILVVAGTGLI
YIRTRKKSA
>ACAKHAOO_00524 putative branched-chain-amino-acid aminotransferase
MSEKVKFEQRDSLKEKPEGNLGFGQYFTDYMLSFDYDVDKGWHDLKIIPYAPFEVSPAAQ
GLHYGQAVFEGLKAYKHEGEVVLFRPDQNFKRINDSLARLEMPKVDEELLLEGLKQLVDV
ERDWVPEGEGQSLYIRPFVFATEGILGVRASHQYKLLIILSPSGAYYGGDTLKTTKIYVE
DEYVRAVRGGVGFAKVAGNYAASLLAQTNANNLGYDQVLWLDGVEQKYVEEVGSMNIFFV
ENGKLVTPALNGSILPGITRKSIIELAKELGYEVEERKVSIEELFEAYDKGELTEVFGSG
TAAVISPVGTLKYEDREIVINNNEPGEITKKLYDIYTGIQSGKLEDTHGWRVVVPEYK
>ACAKHAOO_00540 Serine-aspartate repeat-containing protein I
MSKRRQGPINKRLDFINNKLNKYSIRKFTVGTASILVGATLIFGHADDEAKAAEENHMEN
TSLNSIENDEDKNNQKVDSSNETATTLTEKTTTKEAPSSEENTEDVTTKEAPSSEENTED
VTTKEAPSSEENTEDVTTKEAPSSEENTEDVTTKEAPSSEENTEDVTTKEAPSSEENTED
VTTKEAPSSEENTEDVTTKEAPSSEENTEDVTTKEAPSSEENTEDVTTKEDNTKEVATKE
DNTEEVATKEDNTEEAATKEEAVTTEEAATKEEAVTTEEAATKEEVATAEEAQEVNNKSK
QLLDMDKNSTIDEKIDYAKQTINELNINQNDISNIETSIKNNSDLKNLSKEELNNEILRA
ALVNESNNNDYGLQTLSAIEPLTTNVRNKNNSLSPVSKLRMLATATSGQNVNDKINITNA
SLTLNKKNNQHDDNTVWPTSNEQLRLSADYELDNSIKEGDTFTIKYGDYIRPGALELPAK
NTQLRSKEGSIVANGVYDETTNTTTYTFTNYVDQYQNITGSFNLLATPKRETATKDKQSY
PMDVTIANQEVRENFVVDYGNHKDHLTSAAVANVDNVNNKHNEVVYLNQSGNRIYDAKYF
STVQNGTFIPNEVKVYEVLDDSVLVDSFNPDLNGPAVKDVTSEFTPKYSLNNTRVDIDLN
RSNMNKGKRYIITQAVKPSGTGNVTTNYELTRYGNQESRYPTGTKSTTVSYINGSSTAQG
DNPTYSLGDYVWLDKNKDGIQDDDEKGIPGVYVILKDSNNKELQRATTDDTGHYQFNNLQ
NGTYNVEFVIPNNYTPSPSNTIDNDTIDSDGQKDGDSNVVVAKGTINNADNMTVDTGFYE
TPKYSLGDYVWKDTNKDGVQDSDEKGIQGVTVTLKDKNGNVLKTTTTDENGNYRFDDLDS
GDYIVHFEKPEGLTQTTTNSGSDDEKDADGEEVHVTITDHDDFSIDNGYFDEDSDSDADA
DSDADADADADADSDADADSDADADSDADADADADADADADADADADADSDADADADADA
DSDADADADADADSDADADSDSDADADADADSDADADADADADADADADSDADADADADA
DSDSDADADSDADADADADADADADSDSDADSDADADADADADSDADADADADADADADA
DADSDADADADADADSDADADSDADADSDADADADADADADADSDADADADADADADADA
DADADADADADSDADADSDADADADSDADADADADADADADADADADADADADADADADA
DADADADADADADADADSDADADSDADADADADADSDADADSDADADSDADADADADADS
DADADSDADADADADADADADADADADADADSDADADADADADSDADADSDADADADADA
DSDSDADADADADADADADSDADADSDADADADADADSDADADSDADADADADADADADA
DSDADADSDADADSDADADSDADADADADADADADADADADADADADADSDADADADADA

DADADADADADADADADADADADADADADADADADADADADADADADADADADADADADSDA
DADSDADADADADADSDADADSDADADSDADADADADADSDADADADADADSDADADADA
DADSDADADADADADSDADADSDADADSDADADSDSDADSDADADSDADADADADSDADA
DADADADADSDADADADADSDADADADADADSDADADSDADADADADADSDADADADADA
DADADADSDADADADADADSDSDADADSDADADADADADADSDADADADADADSDADADA
DADADSDADADSDADADADADADADSDSDADSDADADADADADSDADADADADADADADA
DADSDADADADADADSDADADSDADADSDADADADADADSDADADADADADADADADADA
DADADADSDADADSDADADADSDADADADADADADADADADADADADADADADADADADA
DADADADADADADADSDADADSDADADADADADSDADADSDADADADADADSDADADADA
DADSDADADSDADADADADADSDADADADADADADADSDADADSDADADADADADADSDA
DADSDADADADADADSDADADSDADADSDADADADADADSDADADSDADADADADADADA
DADADADADADADADSDADADADADADADADADSDADADADADSDADADADADADADADA
DSDADADSDADADADADADADADADADADADADSDADADADADADADSDADADADADADA
DADADADADADADKDHNDKVNSKELPDTGNENNGTLFGSLFAALGGLFLVGRRRKNKNNE
EK
>ACAKHAOO_00546 C protein alpha-antigen
MLCSINLKNRGVSYLKKKSNKRLDFLPNINNKYSIRKFTVGTASILVGATLIFGVAHDEA
KAAEDTKTEEGVTSNSNESAQSSSETANTGVETTEQATAEQPTTEEKAIEESTTEQPSTE
EKATEESTTEQPSTEEKASKESTTEQPSTEEKASKESTTEQPSTEEKASKESTTEQPSTE
EKASKESTTEQPSTEEKASKESTTEQPSTEEKASKESTTEQSSTEEKASKESTTEQSSTE
EKASKESTTEQPSAEEKASKESTTEQPSTEEKTSEESTTEGSTVIDKDSNQSVQNISQNL
GVSNEETISALSDAGVNTSNASEVDAIAALIQKDYQNNKNENPVATFSNTSSQATTNNNT
RTRTLANKIRIAAAAVAEDNSKIIDADALANGYIKSQTDATNAANTLSGRAWVVDQGTPA
TMSNGLTSVPEGTPVYMQWIDKDGVVSPIYRAHTNNSLSTSGGSQVGPGAYAFDLRQAYT
DANGKEHKYSATSGQYYKLWIEDYQTKNGNTVTMLRQAGGFFPGSYVNSVTSNNIGQFPL
IGTNMQRTGIYMGVKPSSDYMTTSKDQWIHDTQGPISNPAVNTKAKNTVSGQVWLETGTG
VDLANSATGPNLSGNDKVAPGYTVVMSSLTSEGIQAYKAAVDSLPESERADAARVLLSDH
PEYISATVYGETDANGRYTLRFPDGTLNDKYMYGYVVNPNGEVQNAYSSFTSPQFRSPMA
NSSFVPQSRPGQNLIVNPMWYNLNFALVPQNDVKIDITNYNNTTKPARPGDVAQIDLSGT
QLSPLPTHIEWRDKNGNVVSKTDDITSFTDGEQKGQFTVPASAQNGDIYTVVLVTGGKDV
GADSFVVGTPKDNNVYEPTTEGVDKQYGEPTTEDDVTGAVTIPDYPADKEQPTITVDDPT
QLPDGNTPGKTEVDVTVKYPDGTEDHIKVPVTVGEQADNDAYEPTTDGVTKDHGTPTTAD
DVTGSVTIPDYPADKEQPTITVDDETQLPDGNTPGKTEVDVTVKYPDGTEDHIKVPVTVG
EQADNDAYEPTTDGVTKDHGTPTTADDVTGSVTIPDYPADKEQPTITVDDETQLPDGNTP
GKTEVDVTVKYPDGTEDHIKVPVTVGEQADNDAYEPTTDGVTKDHGTPTTADDVTGSVTI
PDYPADKEQPTITVDDETQLPDGNTPGKTEVDVTVKYPDGTEDHIKVPVTVGEQADNDAY
EPTTEEVTKDYGTPTTEDDVTGAVTIPNYPAEKGKPTITVDDPSKLPDGNTPGKTEVDVT
VKYPDGTEDHIKVPVTVGEQADNDAYEPTTDGVTKDHGTPTTADDVTGSVTIPDYPAEKG
KPTITVDDPSKLPDGNTPGKTEVDVTVKYPDGTEDHIKVPVTVGEQADNDAYEPTTDGVT
KDHGTPTTADDVTGSVTIPDYPADKEQPTITVDDETQLPDGNTPGKTEVDVTVKYPDGTE
DHIKVPVTVGEQADNDAYEPTTDGVTKDHGTPTTADDVTGSVTIPDYPADKEQPTITVDD
ETQLPDGNTPGKTEVDVTVKYPDGTEDHIKVPVTVGEQADNDAYEPTTDGVTKDHGTPTT
ADDVTGSVTIPDYPADKEQPTITVDDETQLPDGNTPGKTEVDVTVKYPDGTEDHIKVPVT
VGEQADNDAYEPTTDGVTKDHGTPTTADDVTGSVTIPDYPADKEQPTITVDDETQLPDGN
TPGKTEVDVTVKYPDGTEDHIKVPVTVGTDTVAPDAPTVDVPEAGADTVTGTGSEPGNTI
TVTFPDGTTGTGTVGEDGSWSVEVPGGTELNNGDKITAVETDDAGNVSDEGSAIVVDDSD
ADADSDADADSDADADSDADADSDADADSDADADSDADADSDADADSDADADSDADADSD
ADADSDADADSDADADSDADADSDADADSDADADSDADADSDADADSDADADSDADADSD
ADADSDADADSDADADSDADADSDADADSDADADSDADADSDADSDADADSDADADSDAD
ADSDADADSDADADSDADADSDADADSDADADSDADADSDADADSDADADSDADADSDAD
ADSDADADSDADADSDADADSDADADSDADADSDADADSDADADSDADADSDADADSDAD
ADSDADADSDADSDADADSDADADSDADADSDADADSDADSDADSDADADSDADADSDAD
ADSDADADSDADADSDADADSDADADSDADADSDADADSDADADSDADADSDADADSDAD
ADSDADADSDADADSDADADADSDADADSDADADSDADADSDADADSDADADADSDADAD

SDADADSDADADSDADADSDADADSDADADSDADADSDADADSDADADSDADADSDADAD
ADSDADADSDADADSDADADSDADADSDADADSDADADSDADADSDADADSDADADSDAD
ADSDADADSDADADSDADADSDADADKDHNDKADKPNDKELPDTGNDAQNNATLFGSLFA
ALGGLFLVGKRRKNKNNEEK
>ACAKHAOO_00548 GTP cyclohydrolase FolE2
MTEFDLSTREGRWKHFGSVDPIEGTKPTTKNEMTDLQSTHKNFLFEIEEVGIKNLIYPVN
IDRFQTAGRFSFSTSLNKDEKGINMSRILESVEKHYSNGLELNFDTLYQVLRTLQTNMKQ
NSAGVDVSGKWFFDRFSPVTNIKAVGNADVTYGLAIEQDQITRKEITIEATVTTLCPCSK
EISEYSAHNQRGVVTVKVYLDKNNQVVDDYKDKILDAMEANASSILYPILKRPDEKRVTE
RAYENPRFVEDLIRLIAADLVEFDWIDGFDIECRNEESIHQHDAFAKLKYRK
>ACAKHAOO_00551 3-hexulose-6-phosphate synthase
MELQLAIDLLNKEEAAELAKKVEEYVDIVEIGTPIVINEGLPAVQHLDENINNAKVLADL
KIMDAADYEVSQAIKFGADVVTILGVAEDASIKAAVEEAHKHDKQLLVDMIAVQDLEKRA
KELDEMGADYIAVHTGYDLQAEGQSPLDSLRTVKSVIKNSKVAVAGGIKPDTIKDIVAEQ
PDLVIVGGGIANADDPVEAAKQCRDAIEGK
>ACAKHAOO_00561 Capsule biosynthesis protein CapA
MIYDKERIEQLLDGYWYREPKEEWYVDNIDINKQQMMKYCQKGYKTLFIAIDSETWHKGS
GNTGIYAGWEDTHKNLEEYKYFMSGVIASRPIDYLDEDIPQFIMKDTYSAIKKLGEFSFS
LFKGKMIGITGTAGKSTCKTLLNELLEVNHTVNSTRGNHNTRTGVPLTVANAINNPDYLV
LEMAISSLWMKSGGIAKTYIPDLALITSIDGGQNKTPYETAILKSKIAEGMHHNGKVILN
RDMNEYFTVKSTIEKYNKNIVTYGFNNESDSIIERFEEYKDYTHVEASILGEPVSFNTFL
SGKAMIENIIGVLTIIKLLDIPLESIMYNLENYQPNNGVQNFEHYKKNNGVTYTLINDSW
NAMGISMLEGIKVLKTKSRFYKGKTIAILGRIIGLDKNEKEAERQHELIAEELINSNIDL
VYGHGKEMKYTMKKLPKHMIGGYYERAELLAYEVANIIEDDDLILIKGSVRNSNFKNVKK
HLILNANSNTTHKVNTHKGSSKGYGVATFSVKTNEKVSYIGNQDVIQNQGLGGILIIHHI
LDLIFSKRLSLSDIYKPDKQAIRESKNPRSIPLNEKDEITLNQLLTSAIVTASPNAILML
ANTVIKSNSDSLKYIKETIKEIGANPRSALNITGRRISNKIQELSLNDLYLASKLLFNKY
PFIKDMLTKNNYVFKDKCYKSESNLFNYGMITHGFFYGQDHSIGTVLSKINGEEYITVVL
GAKDAFHRDELIYNSIMQVTQGKPKHTKCDSIRKKRKLPFEMNIIGDTYFGEYYTKKRQA
KDIDDALTSKGRHYSFDGIRDFLKTGDFNICNFEAAISDDDNAYLKQRKPYVLHASEEET
ARALKKEYIHLATLANNHLMDCNIEGLNRTIKQFEAENIYTIGAGNTQEEAEKPFVLNYN
GQKYTIFNAYWYRRPMYREYDFYAIGNKPGVACINPSLYKQISKVKEEGAKVIVIAHWGV
DFGKVQIKQREYAQLLEEAGADLIIGHGAHMMQSIEKINRTTVVYSIGNGIFNSNGEYDQ
RFVPPYSFIARLTITPENDLSLKLYPIYSNNKETFWQPRFLTEDEFKHCSQMLKQYGSIE
TIKKGYDQHYYYDIPL
>ACAKHAOO_00569 Pyridoxine kinase
MALKKVLTIAGSDTSAGAGMQADLKTFQELDTYGMVALTAVVTMDKETWSHDVTPLPMDL
FEKQLETAISIGPDAVKTGMLGTQEIIKRAGEAFEESGAQYFVVDPVMVCKGEDEVLNPG
NTDAMIEYLLPKATVVTPNLFEAGQLSGLGKLTSIENMKKAADIIFNQGAQNVIIKGGKA
LDQDKSYDLYYDGNTFYQLTTDMFQQSYNHGAGCTFAAATTAYLANGKSPKEAVISAKAF
VASAIKNGWKMNDFVGPVDHGAYNRVEHIDVDVTEV
>ACAKHAOO_00577 Phosphate acetyltransferase
MSSLLDVLQEKLSGKNVKIVLPEGEDERVLTAATQLQATDYVTPVVLGDYDKINLLAKDK
GLDVSDIEIINPSTSELKPELVKSFVERRKGKATEEQADELLNNVNYFGTMLVYAGHADG
LVSGAAHSTGDTVRPALQIIKTKPGVSKTSGIFFMIKDDQQYVFGDCAINPTLESSDLAE
IAVESAKSAKSFGMDPRVAMLSFSTKGSAKSDDVDKVANAVKLAQEKVESEGLTDVVIDG
EFQFDAAIVPSVAEKKAPGAKIQGNANVFVFPSLEAGNIGYKIAQRLGGFDAVGPVLQGL
NSPVNDLSRGCSTEDVYKLSIITAAQTLQ
>ACAKHAOO_00584 Cold shock-like protein CspLA
MNNGTVKWFNAEKGFGFIEREDGSDVFVHFSGIAGEGYKTLEEGQKVDFDITEGQRGEQA
TNVVVLS
>ACAKHAOO_00587 Aldo-keto reductase IolS
MADLVQLGKSDVHVFPIALGTNAVGGHNLYPNLDEEQGKDVVRKAIDNGITLLDTAYIYG
PERSEELVGQVVQEYPREQVQIATKGSHVIKENDEVVQNNDPDYLKQQVENSLKRLQVDY

IDLYYIHFPDENTPKDKAVAALKELKDEGKIKAIGVSNFSLEQLKEANKDGYVDVVQLEY
NLLHRENEEVMKYAAENNITFIPYFPLASGILAGKYEENQTFDDHRAPRRDFQPEVFNDN
VRRVKQLQGIAEAHNTSIANVVLAFYLTRPALDVVIPGAKRAEQVVQNIEAANIQLTDDE
LNKIDELFPIE
>ACAKHAOO_00623 Arginine--tRNA ligase
MNIIEKVKSTLIEEIKASIEKANLAEDIPEIKVEIPKDTKNGDYSTNIAMVLTKVAKRNP
REIAQAIVDNLDTSKANVKQVDIAGPGFINFYLDNQYLTAVIPEAINKGDKFGCAEESKN
TNILLEYVSANPTGDLHIGHARNAAVGDSLANILIAAGYNVTREYYINDAGNQITNLARS
IETRFFEALGDTSHEMPADGYNGKDIIEIGKDLADKHPEMKDYSDEERLKTFRQLGVDYE
MDKLKKDLADFNVHFDNWFSETSLYENGAIDNTLAKMNELGYTYEADGATWLRTSDFKDD
KDRVLIKKDGTYTYFTPDTAYHYNKINRGNDILIDLMGADHHGYINRLKASLETFGVDSN
RLEIQIMQMVRLMQDGVEVKMSKRTGNAITLREIMDEVGIDAARYFLTMRSPDSHFDFDL
ELAKEKSQDNPIYYAQYAHARICSILKQAKEQGVEVTADADFSTITNEKAIDLLKKVAEF
EPTIESAAENRAPHRLTNYIQDLASAFHKFYNAEKVLTDDAEKTKAHIALVDAVRITLHN
ALALVGVSAPESM
>ACAKHAOO_00630 Arylesterase
MDLFTRKGGLSLHYNTMGEGYPVVLVHTAYENASIFQNLAKELAKSFQVVLLDLRGHGYS
DKPRQIDFKEFADDIIQLLDYLYIDESALIGHEMGAAIIADLAERYPNYVSSLIMVTPTS
IEGELPEERLFRKYSHKIRNWDDEKQNKFLEKHRYHKPRKVNKFLKHVEDTNAISTKEET
QAIEDVFKETAISSVFEHVTKPTLIIAGEHGERITTLESKEVADLVKESQFDVYAQSSLY
PFEEEKDKFIEDVTPFIKKYMPEH
>ACAKHAOO_00631 hypothetical protein
MKKLVAAFLVSGLVMTGVGVNHAEAASGNTIQTVQQLTQGEKSLENITLGESIKNVSNKY
GTPIYSKNPSNNEGYYEYRTNKGLLVVTAVGKKNQGYVTRISMTYNEANGPTYNQVKKNL
GQNAVARVQYNKVSGNFGYIQKGKTSYQFGSNSPQDKNLKLYRIDLAK
>ACAKHAOO_00634 Transcriptional regulator SarA
MAISKINDCFELLAMITYADKLKNIIKKEFSISFEEFAVLTYISQSKEDEYYLKDIINHL
NYKQPQVVKAVKNLSQEDYFDKKRNEHDERTVLILVNSTQRKKIDSLLNKVNTRIEEANK
ETEL
>ACAKHAOO_00646 Na(+)/H(+) antiporter subunit G
MAQINEIIELIAALLIFLGSIIAVISAIGIVKFQDVFLRSHASTKSSTLSVLLTLVGVLI
YFTNEQSFFSVRLLLSIVFINLTSPVGMHLVARAAYRTGAYMYRKDDAPSRSSILLSSKE
YNSTEELKNRARIREERREKIYYDVQKQRQKEKQQEENIESLSEARRETKD
>ACAKHAOO_00657 Putative glycosyltransferase TagX
MKIAIIIPVYNAEKTIRRAIKSIDTTHEVEIICVNDGSTDHSRHELTQLQKEVKNIMIYN
QDNQGAAASRNFGLAAMSDDVEAFMFLDADDQFLPGRLDKMIDYYERNQEVDIVIGQIGR
GVQGDWKIIPTHEEINRESLVSLAEAPEILQSIGPGGKLFNSKFSALRFDEDVVFCEEHT
FVTRAYLTARDIQLLPLIIYGYNEQEGSITDKRADTFISYIEDARRVRQRVMEMLLLTNE
KAYYSYRMDDLIVSYLIQAYLLKCSKVTPKFIDKVTHYIKDMQHTNYSGDALFRIIQAVE
QGATNWTRDTYEKWRQTLIDVGIGRPGYFRFQAQVMPKKFAFSSKQLVKRTLKR
>ACAKHAOO_00663 Teichoic acid poly(glycerol phosphate) polymerase
MNKLSIIITYFNNEDYISECIHSLKSQRNQDFDIIVVDDGSTDRSTEILQEALKSYDKDV
HFIQLQENTGHAHARNVALEQAHGQYVLFLDADDQLASYAVDYYLQHVNGLDTLIAPIHK
FRSQRPQYVDKDKVRLQYASHQKSPNAILRKESACNILFKNAIIKAHQIQFNESLDIYAD
TSFVLEYLKYAERFVRITNFPFYYRGEVYDPFYGNTLSDQDFTNKFEDHVNSFFDAMQRT
NDKRIKNFLIQRMKNEIKSGFDPALRDIKSRYVSLEDALIKIVKALKWNILKDGKVLYNL
EILFLSMDDVENAKYVNDLRNKTRLVKNIVLNSKMKERSKYQLSDNEDLVDPNTIVFEAF
GGKNYSDSPKYIYEYMQKHYPHLNYIWVFSKPENNNIPGNATKVKKGSQAYYDAYAKAKY
WVTNARLPLYLNKKENQMYIQTWHGTPLKRLANDMKVVRMPGTTTANYKKNFYAEASRWD
RLVSPNRYSSDIFKSAFWMDEDRIWEIGYPRNDVLVNRQNDTEYIEQIKRDLNLPADKKV
IMYAPTWRDDEFVKKGQYLFDLKINLANLQKEIGDEYVILLRMHYLIANALDLHGYDDFA
IDVSNYNDISELYLISDALVTDYSSVMFDFGILKRPQFFFAYDIEKYDKGLRGFYMDYMN
DLPGEIITDEFKLAEELKQIDQHKVKYQDKIESFYNDFCSIENGKASQYIGDYIYKDIEK
KS

>ACAKHAOO_00674 Acetyl esterase
MNTQKKWTIITGIIIVIVSVVVAWGLYHHQQESEKKKQQREKVQINNPNVKLFQNITYNN
HLPGSQLDIMMPDDVDKDTKLPVIFWMHGGGYVAGDKQYKNPLLSQIVEQGYVVVNVNYA
LAPNYKYPTPLIQFDDAIQFIKHNKDRFPIDLDQVVIGGDSAGAQLTSQYVAMQTNSSLR
SEMGFEQRFTPSQIKGAIFFGGFYDMKTVRETEFPRIQMFMHSYTGATKWESEFKNISQM
STINQVTKDYPSTYLSVGDADPFYSQNIEFYRKLKEEGIPVSTLFYDGSHHLHHQYQFHL
EKPESKENMKRVLAFLSRNTSSSGVEQNQQSPFDGSTSSDKSFSLSPY
>ACAKHAOO_00676 hypothetical protein
MKPNVLLAGGSGYIGKYISSVIEKDANIYALSKYPNTKKEDNDRIIWLKRDIYNYTDVVK
AMEGMDIAVFYLDPNKNSAKLTQATARDLNLIAADNFARAAAQQGVSKIVYISGSRFDIE
TVQRLENYGVPVEKTNTQIKRPHINAELQMSKYDDIRTAMRMILPRKWTLSYLVDYFMKW
LNDTRGTFMHTYQDNDRYIVYARKKSKPLLIMEKVEDDSGLITLHLISGSMIKSNQKKQG
KLEFRQIKGTRLVIVHLYDYIPKLLWPIYYFVQAPLQGLIMRGFEIDCRIKHFNGRVQSG
EKMKYTK
>ACAKHAOO_00697 hypothetical protein
MHEQDFHILEGREITLPELGREIENITGRTIVDSTGEIKRVVAHLPNFESDTDTFVATFK
LNHRNDFVDATFVAPKDQRDRLKEIPVHIELVSYISRG
>ACAKHAOO_00701 hypothetical protein
MDKKKIIKTAINVLPIIIVPLVVERKRIKSHPEVQKATHATSNAGQAIANKATGAKEYLG
DKKQEFDNKRELKKIAKENDPEYIQKKGEKLAKKNYKEADKMNKKLQKNIEQRHKEEEKE
REKNEKQRIKDMKKTQKYQEKVGLTPGKLDDETEKKGEKLEKDNKKDVNKLDKKLQKNID
KRHKEEEKLQKQEEKARIKEMKKFKDHEAESVVTQNKNEDNNKA
>ACAKHAOO_00718 PTS system fructose-specific EIIABC component
MRITELLTKDTIAMDLSASDKNGVIDELVNQLDKAGKLSDIAQFKEAIHNRESQSTTGIG
EGIAIPHAKVAAVKSPAIAFGKSKEGVDYQSLDGQPAHLFFMIAAPEGGAQTHLDALAKL
SGILMDDKVRESLLHANSPEEVLQIIDNADDEATKEEEKEAQEQEAAAAGATSGSANTTD
ANEPYVLAVTACPTGIAHTYMARDALKKQADKMGVKMKVETNGSGGIKNHLTQQDIERAT
GVIVAADVHVETDRFDGKNVVEVPVADGIKRPEELINTALDTSRKPFVARGGNSKSTDSE
SNEKQSFGKAFYKHLMNGVSNMLPLVIAGGILMAIVFLFGANSFDPKSSEHNAFAEQLWN
IGNKSAFALIIPILSGFIARSIADKPGFAAGLVGGMLAISGGSGFIGGIIAGFLAGYLTQ
GIKYITRGLPQAVEGLKPTLIYPLLSVTITGLLMIYAFNPPAAWLNNLLLNGLNSLSGSN
IVLLGLVIGAMMAIDMGGPFNKAAYVFATAALTEGNAAPITAAMVGGMIPPLAIATAMIF
FKRKFTKEQRGSIVPNYVMGLSFITEGAIPFAAADPLRVIPSMMVGSGVGGAIALALGSR
INAPHGGIIVIAATDFSHILQTLIALIVGTLVSAIMYGLLKPKVTKNEIEASKAMDE
>ACAKHAOO_00719 hypothetical protein
MDTVTIINLVIFFLLIALTTVFVGSEFALVKVRSTRIEQLAEEGNRSAKIVKKMIANLDY
YLSACQLGITVTSLGLGWLGEPTFEKLLHPLFNLLQLPDALTTTISFVISFIVVTYLHVV
LGELAPKSIAIQHTEKLALTYARPLYYFGNIMKPLIWLMNGSARVIIRMLGVDPDAQTDA
MSEEEIKIIINNSYNGGEINQTELNYMQNIFSFDERHAKDIMVPRTQMVTLNEPFNVDEL
LETIKEHQFTRYPITEDGDKDHIKGFINVKEFLTEYASGKPIKINNYIHELPMISETTRI
SDALVRMQREHVHISLIIDEYGGTAGILTMEDILEEIVGEIRDEFDDDEVNDIVKLGEDT
YQINGRVLLDDLNEKFNIEFEDSEDIDTIGGWLQAHNTNLQVEDHIDTQYDRWIVSEMDN
HQLVWVVLKYEFIDNRPTFGEDQDENEDKKDNNDESRE
>ACAKHAOO_00741 Histidinol-phosphate aminotransferase
MKQQLNQLAAYQPGLSPQALKEKHGIEGELYKLASNENLYGPSPKAKQAVQAHLDELFYY
PETGSPSLRKAISEHLNVDSSRILFGAGLDEVILMISRAVLTPGDKIVTSEGTFGQYYHN
AIVESAEVVQVPLLNGGFDLENIIKEVDEETALVWLCNPNNPTGTYFNHDELESFLERVP
SHVPVLIDEAYFEFVTAEDYPDTLKLQERFDNAFLLRTFSKAYGLAGLRVGYVVATNEAI
EKWNIIRPPFNVTRISEYAAIAALEDQAYLKDVTAKNAKEREKFFEIPQSKHFLPSQTNF
VFVVTEKAQELYEALLKVGCITRPFPTGVRITIGFPEQNDRMIEVLKHFDY
>ACAKHAOO_00744 Di-/tripeptide transporter
MATNNSHEQAVQTIPQRGFFGHPSGLGVLFFVEFWERFSYYGMRAMLIFYMYDQIKNGGL
GIDQTTAMSIMSVYGALIYMSSIPGAWVADRLFGTRGATLIGAVLIIIGHVCLSLPFAMF
GLFASMFFIIIGSGLMKPNISNIVGRLYPENDTRMDAGFVIFYMSVNLGALISPIILQHF

LDVKNYHGGFLIAAIGMALGLVWYMIFNKKNLGSVGMNPTNPLSSEEKKKYGLIVGIIVA
VIAIVLLVTYFTNTLSFNLISNTVLVLGIALPIIYFTTMIRSKDVTDVERSRVKAFIPLF
ILGMLFWAIQEQGSNVLNIYGLERSDMKMNLFGWTTDFGEAWFQSINPLFILLFAPIVSA
IWLKMGKKQPSLPVKFGLGTLLAGASYILMGLIGMSYGDTHFSVNWVILSYVVCVVGELC
LSPTGNSAAVKLAPKAFNAQMMSVWLLTNASAQALNGTLVKLINPLGQTNYFIFLGSVAI
VITVIILAFTPKISKAMKGIH
>ACAKHAOO_00748 Ribonucleoside-diphosphate reductase subunit alpha 1
MKIMNEKKYNHIELNNEVTKRKDNGFFNLEKDQEALAVYLEEIHDKTIYFDSEIERLHYL
VDNNFYFNVFDKYSEEDLVEITEFAKSINFEFASYMSASKFYKDYALKTNDKSQYLEDYN
QHVAIVALYLADGNKQQAKQFILAMVEQRYQPATPTFLNAGRARRGELVSCFLLEVDDSL
NSINFIDSTAKQLSKIGGGVAINLSKLRARGEAIKGIKGVAKGVLPVAKALEGGFSYADQ
LGQRPGAGAVYLNIFHYDVEEFLDTKKVNADEDLRLSTISTGLIVPSKFFDLAKEGKDFY
MFAPHTVKQEYGVTLDDIDLEKYYDDMVANPNIVKKKKDAREMLNMIAQTQLQSGYPYLM
FKDNANKVHANSNIGQIKMSNLCTEIFQLQETSVINDYGIEDEIKRDISCNLGSLNIVNV
METGKIKDSVHTGMDALTVVSDEANIQNAPGVRKANSELHSVGLGVMNLHGYLAKNKIGY
ESEEAKDFANTFFMMMNYYSIERSMEIAKERGEVYQDFEQSDYASGKYFEFYTSQEFEPK
FEKVKRLFDHIDIPTAKDWKALQEQVQQHGLYHAYRLAIAPTQSISYVQNATSSVMPIVD
QIERRTYGNAETFYPMPFLSPETMWYYKSAFNTDQMKLIDLIATIQTHVDQGISTILYVN
SEISTRELSRLYVYAHHKGLKSLYYTRNKLLSVEECTSCAI
>ACAKHAOO_00749 Ribonucleoside-diphosphate reductase subunit beta
MKAVNWNTQEDMTNMFWRQNISQMWVETEFKVSKDIASWKTLTEDEKNTFKRALAGLTGL
DTHQADDGMPLIMLHTTDLRKKAVYSFMAMMEQIHAKSYSHIFTTLLPSSETNYLLDTWV
IEEPHLKYKSDKIIENYHKLWGKEASIYDQYIARVSSVFLETFLFYSGFYYPLYLAGQGK
MTTSGEIIRKILLDESIHGVFTGLDAQSLRNELSENEKQKADKEMYKLLEDLYANEVSYT
HMLYDDIGLSEDVLNYVQYNGNKALSNLGFEPYFEEKEFNPIIENALDTTTKNHDFFSVK
GDGYTLALNVEALQDEDFIFDN
>ACAKHAOO_00753 putative ABC transporter solute-binding protein YclQ
MKKTVLFLVLSLVLVLSACSNGSSSNDSGSKSDSKDSKETVKIKNNFEASGKERDGSDAK
KVSNTVEVPKNPKNAVVLDYGALDVLKELGVADKVKGLPKGENNESLPDFLKEFKDDKYV
NTGNLKEVNFDKVAAAKPEVIFISGRTANQKNLDEFKKAAPDAKIVYVGADEKNLVKDMK
KNTENLGKIYDKEDKAKDINEDLDEKISEMKDETKKFNKTVMYLLVNEGELSTFGPKGRF
GGLVFDTLGFKPADENVSNSPHGQNVNNEYISKHNPDVILAMDRGEVVGGESTAKKVLSN
NVIKDVKAVKNDKVYQLDPKLWYFASGSTTTTMKQIDELEEVVDK
>ACAKHAOO_00761 Glycerate 2-kinase
MKILVAMDEFNGIISSYQANRFVEEAVASQIEDADIVQVPLFNGRHELMDSVFLWQSGTK
YRVNVHDADMKEVETVYGQTEQGLTIIEGNLFIKGQKPVEQRSSYGLGEVIIDALNNGAD
NIVISLGGIDSFDGGIGMMQALGAQFYNDEGELLDAREGAQVIKFIRRIDFSAIPQNFKD
AHIQLMSDFSSKLYGKHSEIMKTYETHQLTREKAAEIDNLIWYFSELVKNEMKIAMGPIE
RGGAGGGIAALLNGLYDAEILTSHELVNQITHLDDLVSQADLIIFGEGVKEEDHLLETTT
LTIAELAQKHDKPAIAICATDDKFDVFDQYHVTAMFNTFVEMPERYTDFKMGIQIRHFTV
QALRLLKTTLNNA
>ACAKHAOO_00771 Ribosome hibernation promotion factor
MIRFEIHGDNLTITDAIRNYIEDKIGKLERYFNDVPNAVAHVKVKTYQNSTTKIEVTIPL
KNVTLRAEERHDDLYAGIDLVTSKLERQVRKYKTRVNRKHKTHGEPEAFVAEVQEAPPET
VDDVNAEPTNDSEIEIIRSKQFSLKPMDSEEAVLQMELLGHDFYIFTDRETDGTSIVYKR
KDGKYGLIETTE
>ACAKHAOO_00772 Protein translocase subunit SecA 1
MGFLSKIVDGNKKETKRLSKIADEVLSLEEDMAILTDEEIKNKTKQFQQEVQEIEDVKKQ
NDYLDKILPQAYALVREGSKRVFNMTPYKVQVMGGIAIHKGDIAEMRTGEGKTLTATMPT
YLNALAGRGVHVITVNEYLSSVQSEEMAELYEFLGLTVGLNLNSKSTNEKREAYAQDITY
STNNELGFDYLRDNMVNYAEERVMRPLHFAIIDEVDSILIDEARTPLIISGEAEKSTSLY
TQANVFAKMLKAEDDYKYDEKTKAVHLTEQGADKAERMFKIENLYDVQNVEVISHINTAL
KAHVTLQRDVDYMVVDGEVLIVDQFTGRTMPGRRFSEGLHQAIEAKEGVKIQNESKTMAS
ITFQNYFRMYNKLAGMTGTAKTEEEEFRNIYNMTVTQIPTNKPVQRDDKSDLIYISQKGK

FDAVVEDVVEKHKQGQPVLLGTVAVETSEYISNLLKKRGIRHDVLNAKNHEREAEIVANA
GQKGAVTIATNMAGRGTDIKLGEGVEEIGGLAVIGTERHESRRIDDQLRGRSGRQGDRGD
SRFYLSLQDELMVRFGSERLQKMMNRLGMDDSTPIESKMVSRAVESAQKRVEGNNFDARK
RILEYDEVLRKQREIIYNERNNIIDSEDSSQLVNAMLRSTLQRGVTYHISEEDDNPDYAP
FINYVNDVFLQEGDLKEEEINGKDSEDIFEVVWSKIEKVYESQKAKIGDQMAEFERMILL
RSIDTHWTDHIDTMDQLRQGIHLRSYAQQNPLRDYQNEGHELFDMMMQNIEEDTSKFILK
SVIQVDEDIEREKTTDFGTAQHVSAEDGKEKAKKQPIVKGDKVGRNDPCPCGSGKKYKNC
HGKEE
>ACAKHAOO_00779 UvrABC system protein A
MKGPSIIVKGARAHNLKDVDIELPKNKLIVMTGLSGSGKSSLAFDTIYAEGQRRYVESLS
AYARQFLGQMDKPDVDTIEGLSPAISIDQKTTSKNPRSTVATVTEIYDYIRLLYARIGKP
YCPNHNIEIESQTVQQMVDRILELEERTKIQILAPVVSHRKGSHEKLIADIGKKGYVRLR
VDGDIVDVNEVPELDKNKNHTIEVVVDRLVVKDGIETRLADSIETGLELADGNITVDVID
GEDLKFSENHACPICGFSIGELEPRMFSFNSPFGACPTCDGLGQRLTVDLDLVVPDPNKT
LNEGAIEPWEPTSSDFYPTLLKRVCEVYKINMDKPYKKLTDRQKNILMNGSGDKEIDFSF
HSRNGGTRHRRMKFEGVVANIDRRYHESPSEYTREVMSKYMTELPCETCHGKRLSKEALS
VYVGGYNIGEVVEYSIKNALNYYENIELSEQDRAIANQILKEIISRLSFLNNVGLEYLTL
NRASGTLSGGEAQRIRLATQIGSRLTGVLYVLDEPSIGLHQRDNDRLISTLKEMRDLGNT
LIVVEHDDDTMRAADYLVDVGPGAGNHGGEIVSSGTPSKVMKDKKSLTGQYLSGKKRIDV
PEHRREVTDRKISIKGARSNNLKGVDVDIPLSVMTVVTGVSGSGKSSLVNEVLYKSLAQK
INKSKVKPGAFDSIEGIDQLDKIIEIDQSPIGRTPRSNPATYTGVFDDIRDVFAQTNEAK
IRGYQKGRFSFNVKGGRCEACKGDGIIKIEMHFLPDVYVPCEVCDGKRYNRETLEVTYKG
KNIAEVLEMTVEEATYFFENIPKIHRKLQTLVDVGLGYITLGQQATTLSGGEAQRVKLAS
ELHKRSTGRSIYILDEPTTGLHVDDISRLLKVLNRLVENGDTVVIIEHNLDVIKTADHII
DLGPEGGDGGGTLVATGTPEEIANVESSYTGKYLKTVLERDKVE
>ACAKHAOO_00784 Thioredoxin reductase
MTEINYDVAIIGAGPAGMTAAVYASRANLSTVMIERGMPGGQMANTEEVENFPGFEMITG
PDLSTKMFEHAKKFGAEYQYGDIKSIEDKGDYKEINLGNKEITARAVIISTGAEYKKIGV
PGEQELGGRGVSYCAVCDGAFFKNKNLFVIGGGDSAVEEGAFLTKFADKVTIVHRRDELR
AQKILQDRAFKNDKIDFIWSHTLKSINEKDGKVGSVTLVSTKDASEQTLDADGVFIYIGM
KPLTAPFVNLGITNDMGYIVTEDNMSTKVPGIFAAGDVRDKGLRQIVTATGDGSIAAQSA
IDYIEELKDKQEA
>ACAKHAOO_00789 ATP-dependent Clp protease proteolytic subunit
MNLIPTVIETTNRGERAYDIYSRLLKDRIIMLGSAIDDNVANSIVSQLLFLQAQDSEKDI
YLYINSPGGSVTAGFAIYDTIQHIKPDVQTICIGMAASMGSFLLAAGAKGKRFALPNAEV
MIHQPLGGAQGQATEIEIAANHILKTREKLNKILAERTGQSIEKIQKDTDRDNFLTADEA
KEYGLIDNVMQPEEK
>ACAKHAOO_00793 hypothetical protein
MSKNNRNRDDFGEPEKKKMSGISKLISTIIVLLLLSGLAFAIFAFVDHSNRSSERLNNQS
TEEHKDKNDKKDKEDKDKKDKSDKDKKTSEDSSNVTQENVTQQTAQTQQRQTQQATPSTQ
QRTKQQPSMEEKQRTKEAQTKEEKTQEKTTKEEATTEETRTKEQATQESNSNNSSDNNSS
ESNTDKSNSNQTSNQTRTQQSPQTQQRSTTQSSTQQSTSRQTSQRSATSSQSQSSQESSN
SNE
>ACAKHAOO_00795 Central glycolytic genes regulator
MKDLIKIQQKLIPDLIDKMYRRFSILTTISNNQPVGRRSLSEHMDITERVLRSETDMLKK
QDLIQVKSTGMEITEEGKSVLNQLNEYFNIYTDDNQLADQIKSTFGIKEVHVIPGDADAQ
ENVKSEMGRQAGQLLESILYEDAIVSVTGGSTMANVSRAMHLLPFNVFFVPARGGLGENV
IYQANTISASMAQQTGGYYTTLYVPDNVSESTYNTLLMEPSVVHTLDKIKQANITIHGIG
DALKMAYRRQSSEQVIESLQHHQAVAEAFGYYFDAQGNVVYKVKTIGLQLEDLETKDFIF
AVAGGQSKGEAIKAYLSIAPKNTVLITDEAAAKVILN
>ACAKHAOO_00796 Glyceraldehyde-3-phosphate dehydrogenase 1
MAVKVAINGFGRIGRLAFRRIQDVEGIEVVAVNDLTDDEMLAHLLKYDTMQGRFTGEVEV
VDGGFRVNGKEVKSYEEPDASKLPWGDLDIDVVLECTGFYTDKEKAEAHINAGAKKVLIS
APAKGDVKTIVFNTNHNDLDGSETVVSGASCTTNSLAPVAKVLSDEFGLVEGLMTTIHAY

TGDQMTQDGPHKKGDKRRARAAAQNIVPNSTGAAKAIGKVIPEIDGKLDGGAQRVPVATG
SLTEVTVVLEKDVTVEDVNKAMKNASNESFGYTEDEIVSSDVVGMTYGSLFDATQTRVMS
VGDRQLVKVAAWYDNEMSYTAQLVRTLEYLAELSK
>ACAKHAOO_00797 Phosphoglycerate kinase
MAKKIVSDLDLKGKVVLERADFNVPLKDGKITNDNRIVQALPTIKYIIEQGGKLVLFSHL
GKVKQESDKEGLTLKPVADALSEKLGKEVTFVPETRGEKLESAIKNLSEGDVLLVENTRF
EDLDGKKESKNDSKLGKYWASLGDVFVNDAFGTAHREHASNVGISTHLETAAGYLMEKEI
KFIGGVVNDPHKPVVAILGGAKVSDKIGVIKNLVNIADKILIGGGMAYTFLKAQGKEIGL
SLLEEDKIDFAKELLESNGDQIVLPVDAKVAKEFSNDAEITEVSIDNIPSDQEAMDVGPK
TVELFSKELEGAHTVVWNGPMGVFEFSNFAQGTIGVCKAIANLKDATTIIGGGDSAAAAI
SLGFEDDFTHISTGGGASLEYLEGIELPGIKAINDK
>ACAKHAOO_00798 Triosephosphate isomerase
MRKPIIAGNWKMNKTVKEAKDFVNNLPTLPDEKEVESVICAPTIQLDALISLVNDGKAKG
LKIGAQNTYFEDNGAFTGETSPVALADLGVKYVVIGHSERRELFHETDEDVNKKAHAVFN
HGMTPIICVGETDEERENGKANEIVSNQVEKALEGLSEEQLKEVVIAYEPIWAIGTGKSA
TSDDANEMCAHVRKTVAKVASQDVADATRIQYGGSVKPNNVKEYMAQSDIDGALVGGASL
KVEDFVQLLEGAK
>ACAKHAOO_00799 2,3-bisphosphoglycerate-independent phosphoglycerate
mutase
MAKQPTALIILDGFANRESEHGNAVKQAHKPNFDRYYSKYPTTQIEASGLDVGLPEGQMG
NSEVGHMNIGAGRIVYQSLTRINKSIADGDFFENDVLNNAVQHVNEHDSALHVFGLLSDG
GVHSHYQHLFALLDLAKQKGLDKVYVHAFLDGRDVDQKSALKYIEDTEAKFKSLGVGQFA
SISGRYYAMDRDKRWDREQKAYNAIRNFEGPTYASAKEGVEANYANDLTDEFVEPFIVEG
QNNGINDGDAVIFFNFRPDRAAQLSEVFTNKAFDGFKVEQVNDLFYATFTKYNDNVDAEV
VFEKVDLTNTIGEVAQNNNLKQLRIAETEKYPHVTYFMSGGRNEEFEGERRRLIDSPKVA
TYDLKPEMSAYEVKDALLEELDKGDLDLIILNFANPDMVGHSGMLEPTIKAIEAVDECLG
EVVDKILDMNGYAIITADHGNSDQVLTDDDQPMTTHTTNPVPVIVTKEGVTLRETGRLGD
LAPTLLDLLNVDQPSDMTGESLIKH
>ACAKHAOO_00800 Enolase
MPIITDVYAREVLDSRGNPTVEVEVLTESGAFGRALVPSGASTGEHEAVELRDGDKSRYS
GKGVTKAVENVNEIIAPEIVEGEFSVLDQVSIDKMMIQLDGTPNKGKLGANAILGVSIAV
ARAAADLLGQPLYKYLGGFNGKQLPVPMMNIVNGGSHSDAPIAFQEFMILPTGAESFKEA
LRWGAEIFHNLKSILSERGLETAVGDEGGFAPKFEGTEDAVETIIKAIEKAGYKPGEDVF
LGFDCASSEFYENGVYDYTKFEGEHGAKRSAAEQVDYLEELIGKYPIITIEDGMDENDWD
GWKQLTDRIGDKVQLVGDDLFVTNTEILSRGIEQGIGNSILIKVNQIGTLTETFEAIEMA
QKAGYTAVVSHRSGETEDTTISDIAVATNAGQIKTGSLSRTDRIAKYNQLLRIEDELFET
AKFDGIKSFYNLDK
>ACAKHAOO_00804 Ribonuclease R
MNLKQSIEEIIKQPDYEPMSVSDFQDALGLNSADSFRDLIKVLVELEQAGLVERTKTDRY
QRKQSHKSSNSKLIKGTLSQNKKGFAFLRPEQEDMDDIFIPPTKINRALDGDTVIVEIQK
SKGEHKGKIEGEVKSIEKHSVTQVVGTYSEAKHFGFVIPDDKRIMQDIFIPKGQSLGAVD
GHKVLVQITKYADGTDNPEGHISAILGHKNDPGVDILSIIYQHGIEIEFPDNVLKEAEAV
PEEIKPDEIKGRTDLRDELTITIDGADAKDLDDAISVKKLKNGHTELTVSIADVSYYVKE
DSALDKEAYDRATSVYLVDRVIPMIPHRLSNGICSLNPEVDRLTLSCRMEINERGEVVKH
DIFDSVIHSNYRMTYDAVNQIITEKDHDVRKQYQEITPMLDLAQDLSNRLIQMRKRRGEI
DFDISEAKVLVNNEGLPTDVVLRERGEGERLIESFMLAANETVAEHFNKLEVPFIYRVHE
QPKSDRLRQFFDFITNFGIMIKGTGEDIHPSTLQSIQEEVEGRSEQMVISTMMLRSMQQA
HYDDTNLGHFGLSAEYYTHFTSPIRRYPDLTVHRLIRKYLIDNSMNNKELHKWEDKLPEL
AEHTSQRERRAIEAERDTDELKKAEYMIQHIGDEFEGIVSSVANFGMFIELPNTIEGMVH
ISNMTDDYYQFDERQMALIGERQAKVFRIGDQVTVKVTHVDVDERMIDFQIVGMPLPKND
RSQRPSRGKTIQAKTRGKSLDKSKDDDHKGKKKKRKQRKGKNQRSRDKQGKTNHKPFYKD
KSVKKKSRRKKK
>ACAKHAOO_00894 Organic hydroperoxide resistance protein-like
protein

MAVQYETKATNVGGRKGHVNTDDNAINVDVLPPQQADGNATNPEQLFAAGYASCFNGAFD
LILKQNKVRDAEPEVTLTVRLEDDPDAESPKLSVAIDAKVKNVLSQEDAEKYLQDAHEFC
PYSKATRGNIDVDLNVQVVD
>ACAKHAOO_00899 Glycine cleavage system H protein
MAVPSELKYSKEHEWVKVEGNTVTIGITEYAQGELGDIVFVELPEVDDEINEGDTFGSVE
SVKTVSELYAPVSGKVVESNEELEDSPEFVNESPYEKAWMVKVELSDESQLDDLLSADQY
KEMIGE
>ACAKHAOO_00902 Methionine import ATP-binding protein MetN 2
MIELNQVVKRYHTKDKDVLAVDNVNLNIETGSIFGVIGFSGAGKSTLIRMFNNLEEPTSG
DIIIDGDNINKLSKAELRRKRQKVSMVFQHFNLLWSRTVLRNITFPLEIAGYSKTKANER
AKELIDLVGLNGRENAYPSELSGGQKQRVGIARALVNEPDVLLCDEATSALDPQTTDEIL
DLLLKIKERENLTIVIITHEMHVIRRVCDEVAVMESGRVIEQGKVTQVFENPQHEVTRRF
VKDDLDDDFEESIKHLEPLDSDAYIVRLNFNGGNTTEPVVSYISKTHNIDINILEANIKN
TRGGSVGFLVVHIPHIAETEFETFKEDLHQQHVNVEVVKHG
>ACAKHAOO_00904 Methionine-binding lipoprotein MetQ
MKKLLSLFLVLTLTVVLAACGNGSGDKDKKIVVGASPAPHAEILEKAKPLLKKKGYDLEI
KTINDYTTPNKLLDKGELDANYFQHVPYLKTESKEKGYKIEDAGKVHLEPMAVYSKKYKS
LKDLPKGATVYVSNNPAEQGRFLKFFVDEGLIKIKKGVKIEDAKFEDITENKKDIKFNSK
QSAEYLPKIYQNEDVDAAIINSNYAIDQGLSPQKDSIAIEKPNNNPYANLIAVQKGHKDD
EKIKALIEVLQSKEIQDFIKDKYKGAVVPAK
>ACAKHAOO_00906 hypothetical protein
MAEDKFEQAKGNLKETVGNVTDNKDLEKEGQNDKASGKAKEAVENVKNKANDLIDKVKGN
NDNK
>ACAKHAOO_00909 Vegetative protein 296
MSSTLEIKDLHVSIEDKEILKGVNLTINTGEIHAIMGPNGTGKSTLSSAIMGHPSYEVTQ
GEVLLDGVNILELEVDERAKAGLFLAMQYPSEITGVTNADFMRSAINAKREEGQEINLMQ
FIKKLDKEMDFLDIDKDMAQRYLNEGFSGGEKKRNEILQLMMLEPKFAILDEIDSGLDID
ALKVVSKGINEMRGEEFGALMITHYQRLLNYITPDKVHVMYGGKVVKSGGPELAKRLEEE
GYEWVKEEFGAKE
>ACAKHAOO_00910 hypothetical protein
MTTETLNISEEQLVDYSKAHNEPSWMTELRQKALKLTETLEMPKPDKTKLRKWDFDSFKQ
HEVKGQSFSNLSELPEAIKKIIDVENTKNLVVQHNNALAYTQVSDQAQKNGVIIEGLSEA
LINHGELVQKYLMTDAVSVDEHRLTALHTALINGGVFVYVPKNVVVEDPIQYVVLHDDDN
ASFFNHVIIVTEESAEVTYVENYLSTASGEGNQLNIVSEVIAGANSNITYGSVDYLDKGF
TGHIIRRGTTAADASINWALGLMNEGSQIIDNTTNLIGDRSTSELKSVVVGTGDQKINLT
SKIVQYGKETNGYILKHGVMRENASSVFNGIGYIKHGGTKSIANQESRVLMLSENARGDA
NPILLIDEDDVEAGHAASVGRVDPEQLYYLMSRGISQKEAERLVIHGFLDPVVRELPIED
VKRQLREVIELKVNK
>ACAKHAOO_00911 putative cysteine desulfurase
MAETSLNVEEIIKDFPILDQKVNGKRLAYLDSTATSQTPVQVLNVLDDYYKRYNSNVHRG
VHTLGSLATDGYEGARETVRRFINAKYFEEVIFTRGTTAAINIVARSYGDANVEEGDEIV
VTEMEHHANIVPWQQLAKRKNASLKFIPMTDEGELRLEDVKATINDNTKIVAIAHVSNVL
GTINDIKEIAKVAHEHGAIISVDGAQSAPHMKIDVQDLDADFFSFSGHKMLGPTGIGVLY
GKRDLLKEMEPAEFGGDMIDFVSKYDATWADLPTKFEAGTPIIAQAIGLGEAIRYIEKLG
FDAIHEHEKALTEYAYEQMSSIEGLEIYGPPKERRAGVITFNLADIHPHDVATAVDTEGV
AVRAGHHCAQPLMKWLNVSSTARASFYVYNTKEDVDQLVEALKQTKEFFSYEF
>ACAKHAOO_00913 hypothetical protein
MAKKAPDVGDYKYGFHDEDVSIFRSERGLTENIVREISKMKEEPEWMLDFRLKALKLFYK
MPMPQWGGDLSELNFDDITYYVKPSEHTQRSWDEVPEEIKRTFDKLGIPEAEQKYLAGVS
AQYESEVVYHNMEKELEEKGIIFKDTDSALRENEELFKEYFASVVPAADNKFAALNSAVW
SGGSFIYVPKNVKLDTPLQAYFRINSENMGQFERTLIIADEGASVNYVEGCTAPVYTTSS
LHSAVVEIIVHKDAHVRYTTIQNWANNVYNLVTKRTFVHENGNMEWVDGNLGSKLTMKYP
NCVLLGEGAKGSTLSIAFAGKGQVQDAGAKMIHKAPNTSSTIVSKSISKNGGKVIYRGIV
HFGRKAKGARSNIECDTLILDNESTSDTIPYNEVFNDNISLEHEAKVSKISEEQLFYLMS

RGISEEEATEMIVMGFIEPFTKELPMEYAVEMNRLIKFEMEGSIG
>ACAKHAOO_00923 Acid sugar phosphatase
MKNYKGYLIDLDGTMYIGTDEIDGAAQFIDYLNNHQIPHLYVTNNSTKTPEEVTQKLKEM
NIDAKPEEVVTSALATANYIADEKSDATVYMLGGSGLRTALTEAGLTVKDDENVDYVAIG
LDENVTYEKLAVATLAVRKGARFISTNPDVSIPKERGFLPGNGAITSVVSVSTGQAPQFI
GKPEPVIMDIALDILKLDKSDVAMVGDLYDTDIMSGINVGVDTIHVQTGVTTYEELKEKD
QQPTYSFKDLNVAISELERNAQK
>ACAKHAOO_00934 NADH dehydrogenase-like protein
MAQDRKKVLVLGAGYAGLQTVTKLQKELSADEADITLINKNKYHYEATWLHEASAGTLNY
EDLIYPIESVIKEDKVKFINAEVTKIDRNAKKVETNHGIYDYDILVVALGFESETFGING
MKDYAFQIENIETARKLSRHIEDKFANYAASKEKDDKDLAILVGGAGFTGIEFLGELTER
IPELCNKYGVNQNKVRVTCVEAAPKMLPMFSDELVNYAVNYLEDRGVEFKIATPIVACNE
KGFVVKINDQEQQLEAGTAIWAAGVRGSKLMEESFEGVKRGRIVTKQDLTIEGHDDIFVI
GDVSAFIPAGEERPLPTTAQIAMQQGEHVAKSIKNILNGQAATDFEYVDRGTVCSLGAHD
GVGIVYGRDITGKKAAFMKKVIDTRAVFKIGGVGLAFKKGKF
>ACAKHAOO_00947 Putative peptidyl-prolyl cis-trans isomerase
MTNYPQLNKEIQDNEIKVAMHTNKGDMTFKLFPDIAPKTVENFVTHAKNGYYDGITFHRV
INDFMIQGGDPTATGMGGESIYGGSFEDEFSLEAFNLYGALSMANAGPNTNGSQFFVVQM
KEVPESMVNQLVDGGWPEPIAKAYADNGGTPWLDQKHTVFGQLIEGEATLEDIANTKVGA
QDKPVHDVVIESIDVEDK
>ACAKHAOO_00951 NAD-specific glutamate dehydrogenase
MTENNNLVTSTQSIIKEALHKLGFDDGMYDLIKEPLRFLQVRIPVRMDDGTVKTFTGYRA
QHNDAVGPTKGGVRFHPEVDEEEVKALSMWMTLKCGIVNLPYGGGKGGIVCDPRQMSIHE
VERLSRGYVRAISQFVGPTKDIPAPDVFTNSQIMAWMMDEYSALDKFNSPGFITGKPIVL
GGSQGRDRSTALGVVIAIEQAAKRRGKEIKGSRVVIQGFGNAGSFLAKFLYDMGAKVVGI
SDAYGALHDPEGLDINYLLDRRDSFGTVTNLFDNTISNKELFELDCDILVPAAISNQITE
DNANDIKADIIVEAANGPTTPEATRILTERGILLVPDVLASAGGVTVSYFEWVQNNQGYY
WSEEEVNDKLREKLVDAFDTIYELAQNRKIDMRLAAYIVGIKRTAEAARYRGWA
>ACAKHAOO_00955 Glucose-6-phosphate isomerase
MTHIQLDYGKALEFFGQHEIDQQQDIVKTIHKTIHEGTGAGSDFLGWVNLPEDYDKKEFS
RIVEASKRIKSNSDVLVVIGIGGSYLGARAAIEMLTSSFRNSNEYPEIVFVGNHLSSTYT
KELVDYLSDKDFSVNVISKSGTTTEPAVAFRLFKQLVEDKYGKAEAKKRIFATTDKAKGA
LKQLADNEGYETFVVPDDVGGRYSVLTAVGLLPIATAGINIESIMIGANKARKELSSDKL
DENIAYQYATIRNILYSKGYTTEMLINYEPSMQYFNEWWKQLYGESEGKDFKGIYPSSAN
YTTDLHSLGQYVQEGRRFLFETVVKVNNPKHDITIEEDSDDLDGLNYLAGKTIDEVNTKA
FEGTLLAHTDGGVPNVVVNIPRLDEETFGYVVYFFELACAMSGYQLGVNPFNQPGVEAYK
QNMFALLGKPGFEDKKKELEERL
>ACAKHAOO_00958 Signal peptidase IB
MKKEIVEWIVAIAVGLLLVWVMVNFVAKSYTIKGDSMDPTLKDGEHVMVNILGYKVGDIK
KGNVIVFHANQQDDYVKRVIGVPGDNVIYKNDKLYVNGKKINEPYLDYNEKRKQGEYITG
SFETKDLLNANPKSNIIPKGKYLVLGDNREVSKDSRAFGLIDRDQIVGKVSFRFWPFSEF
KFNFNPDNEK
>ACAKHAOO_00961 putative protein
MKFLSFNYKDAESYGVKVKREDAVWDLKKVFAEFGEGDFHPQTLLEGLQQNQTLDFQEQV
RKAVVAAEDSGKADEFKIAFNDIEFLPPVTPPNNVIAFGRNYQDHASELNHEVQRLYVFT
KAASSLTGDESTIPNHKDITDQLDYEGELGIVIGKSGEKIPKALALDYIYGYTIINDITD
RNAQNEQDQAFLSKSLTGGCPMGPYIVTKDELPTPENVNIVTKVNNDIRQDGNTSQMINK
IDDLIEEISKYVALHPGDIIATGTPAGVGAGLQPPQFLQPGDEVKVTIDNIGTLTTYIAK
D
>ACAKHAOO_00967 Chaperone protein ClpB
MTYAIQGALQKAVELSKENELQNIEIEAILKGTLEETDSLFKSILERANIDTDELNQAYT
NKLKNYPSVQGDNIQYGQYIGAKANELLNKAESYMKEYEDEYISMEHVLRAAMDIDDTTK
QFVGNKEEVVKEIITKVRGGNHVTSQNPEVNYEALEKYGRDLVEEVRQGKMDPVIGRDEE
IRNTIRILSRKTKNNPVLIGEPGVGKTAIVEGLAQRIVRKDVPESLLDKTIFELDLSALV

AGAKFRGEFEERLKAVLKEVKESDGRIILFIDEIHMLVGAGKTDGAMDAGNMLKPMLARG
ELHCIGATTLNEYREYIEKDSALERRFQKVGVSEPDVEDTISILRGLKERYEVYHGVRIQ
DRALVAAAELSDRYITDRFLPDKAIDLVDQACATIRTEMGSNPTELDQVNRRVMQLEIEE
SALKNESDNASKHRLEELQEELSNEKEKQASLQSRVEQEKEKIAKVQEKRAELDRSRQAL
EDAQTEGNLEKAAELQYGTIPQLEKELKEFEEAYQDEQGDSERMIREVVSDEEIGDIVSQ
WTGIPVSKLVETEREKLLNLSDILHERVVGQDKAVDLVSDAVVRARAGIKDPNRPIGSFL
FLGPTGVGKTELAKSLAASLFDSEKHMIRIDMSEYMEKHSVSRLIGAPPGYVGHDEGGQL
TEAVRRNPYSVILLDEVEKAHSDVFNVLLQILDEGRLTDSKGRSVDFKNTIIIMTSNIGS
QVLLENVKDTGDITEETEKAVMDSLHAFFKPEILNRMDDIVLFKPLSINDMSMIVDKILT
QLNIRLMDQRISIEVSDDAKKWLGEEAYEPQFGARPLKRFVQRQIETPLARMMIKEGMPE
GTVVNVDLDDNHELTFDVQKPENE
>ACAKHAOO_00970 3-oxoacyl-[acyl-carrier-protein] synthase 2
MTENKRVVITGMGALSPIGNDAQTSWENALKGVNGIDTITRIDTEDYNVHLAGELKDFNI
EDHIEKKEARRMDRFTQYAVVAARQAVEDAKLEINDETANRIGVWIGSGIGGMETFETAH
TTLQNKGPRRVSPFFVPMLIPDMATGQVSIDLGAKGPNGSTVTACATGTNSIGEAFKIIQ
RGDADAMITGGTEAPITHMALAGFSASRALSTNDDKETACRPFQEGRDGFVMGEGAGILV
IESLESAQARGAQIYAEIVGYGSTGDAYHITAPAPEGEGGSRAMQAALDDAGIEAKDVQY
LNAHGTSTPVGDMYEIRAIKNTFGDAANQLKVSSTKSMTGHLLGATGGLEAIFSALSIRD
SKVAPTIHANTPDPECDLDIVPNEAQDLEITYAMSNSLGFGGHNAVLVFKKFED
>ACAKHAOO_00974 Oligopeptide transport ATP-binding protein OppD
MSERVLEVNDLHVSFDITAGEVQAVRGVDFYLNKGETLAIVGESGSGKSVTTKAITKLFQ
GDVGRIKKGKIDFLGEDLATKSESELIKLRGKDISMIFQDPMTSLNPTMQIGKQVMEPLI
KHKNYSKSEAKKRALEILNLVGLPNAEKRFKAFPHQFSGGQRQRIVIATALACEPKVLIA
DEPTTALDVTMQAQILDLMKDLQKKIDTSIIFITHDLGVVANIADRVAVMYGGQMIETGD
VDEIFYDPKHPYTWGLLSSMPDLTTSNDTELIAIPGTPPDLLHPPKGDAFARRSQYALDI
DFKQEPPWFKVSPTHFVKSWLLDERAPKVKPPEMVVKRQRQMPNNYDKPRLVERVSFDG
>ACAKHAOO_00976 Dipeptide-binding protein DppE
MKNINFKVILGLIVTTLLLSACGKGGGLYDDSGQTYRVATSSDINTLDSTLATDTVSFTI
YNQVYEGLYSLNGKDEAVPGVAKGKPKKSNGDKTWTIKLRKDAKWSNGDPVTANDFVFAW
RKAVDPVNASEYAYIMYDIKNAEDINLNKNGKKPKDLGVKALDDYTLQVELTKPIPYFQQ
MLAFGTFMPQNEKVVKKYGKSYGTTADKTVYNGPFKIDKWAVEDKIMLTKNKGYWDKNNV
KLDRVNYKVLKDTQAGASLYDTGSVDVAGITSEQVDKYKDSPALFKRLLASTAFLKMNQK
QQPEFKNKDMRYAIAQSVNKEDYVNDVLGTGAKPFDGFTAKGTAKTPDGKDYAETVKSPL
TYNPKEAKKHLEKAKKALGKDKFTFTLNTEDTPDSKISAEFIKSQIENNLPGVTVKVKQL
PFKQRLQAELTMNYSMSLSVWGPDYPDPMTFLDTMTTGNAQNNTNWSSKEYDNLLKKANS
SLLQKPDERYAAMRQAEEIFLNDAPVAPIYQKGGASLRNPQLKGIEYHQIGGDYSLKHAY
MDKSIDRETGKKK
>ACAKHAOO_00986 Oligoendopeptidase F, plasmid
MSQQLTREEQERKYPNDTWDLTTIFKNDEAFEEALKEVEGYLGKEEQFKGHLADSADTLC
DALALEDEIGTKLEKVYVYAHLKQDQDTSNDKYTGFESRAHQLIIKISSAWSFLVPEILQ
IDEDKLQSFIETNDNLKRYEFDLKLINEKRPHILDAEQERLLTEAQDALSTPSNVYGMFS
NADLEFEDAVDKDGEKHPLTQGTFIKYLESDDRELRQSAYNNLYKAYGAYNNTLGSTLAG
EVKKNVFNARTHNYKTARERALSNNHIPEEVYDNLVKTVHKYLPLLHRYTKLRKELLAVD
ELKMYDLYTPMVKDVKFEMPYEEAKEWMLKALEPMGEEYLDVVKEGLNNRWVDVYENKGK
RSGGYSSGAHLTNPFILLNWSDTVSDLYTLIHEFGHSAHSYFSRKHQPSNSSDYSIFVAE
VASTCNEALLSDYMDKHLDDERRLLLLNQELERFRATLFRQTMFAEFEHKIHQIEEAGEP
LTATRMNDEYAKLNKQYFGDVVETDDNISKEWSRIPHFYMNYYVYQYATGYSAAQSLSHQ
ILTEGKPAVERYINEFLKKGSSNYPIEILKNAGVDMTSPEPIEQACEVFEQKLDTFEKLM
KA
>ACAKHAOO_00994 Magnesium transporter MgtE
MSLEIENNDYNKDEVYNQNLLDDLLKQDDIDRFREEFLAMHEYEQSEYFEDTSNENRQKI
FEFLSPQEVGEFFEQLEIDDEAYEDLFDTMNAHYASKVLEEMSSDNAVDILNELSKPKVA
SLLTLMNRDDANEIKHLLHYEEDTAGGIMTTEYISLDVNTPVKEALMLVKEQAPDAETIY
VIFAVNTDGQLVGVLSLRDLIVAENDAYIEDIMSERVISVNVADDQEDVAQKMRDYDFIA

MPVIDYQQHLLGIITIDDILDVMDEEASEDYSRLAGVSDIDSTNDSVVKTATKRLPWLII
LTFLGMITATILGSFEATLSQVALLAAFIPIISGMSGNSGTQSLAVSVRNISTGEIDEQS
KFKVALREAGSGLLSGLVCAIVLFIIIVVIYRQPFLALIVGGSLTIAMTVGTLVGSMIPL
FMNKLNIDPAVASGPFITTINDIVSMLIYFGLATTFMSYLT
>ACAKHAOO_00996 Enoyl-[acyl-carrier-protein] reductase [NADPH] FabI
MINLENKTFVIMGIANKRSIGFGVAKVLDQLGAKLVFTYRKDRSRKELEKLLEQLNQEEP
KLYQIDVQKDEDVVNGFAKIGEEVGNIDGVYHSIAFANIEDLRGRFSETSREGFLLAQDI
SSYSLTIVAHEAKKIMPNGGSIVATTYLGGEFAVQNYNVMGVAKASLEANVKYLAYDLGP
DNIRVNAISAGPIRTLSAKGVGGFNTILKEIEERAPLRRNVDQEEVGKTAAYLLSDFSSG
VTGENIHVDSGFHAIK
>ACAKHAOO_01004 Peptide chain release factor 3
MSLKEEVESRKTFAIISHPDAGKTTLTEKLLYFSGAIREAGTVKGKKTGKFATSDWMKVE
QERGISVTSSVMQFDYDDYKINILDTPGHEDFSEDTYRTLMAVDSAVMVIDCAKGIEPQT
LKLFKVCKMRGIPIFTFINKLDRVGKEPFELLDEIEETLNIDTYPMNWPIGMGQNFFGII
DRESKSIEPFRDEENVLHLDDDYELQEEHAIRNDSAFAQAIEEFMLVEEAGEEFDNDALL
NGELTPVFFGSALANFGVQNFLNAYVDHAPMPNARQTNEEVEVSPFDDEFSGFIFKIQAN
MDPKHRDRIAFMRVVSGAFERGMDVTLQRTSKKQKITRSTSFMADDKETVNHAVAGDIIG
LYDTGNYQIGDTLVGGKQKYSFQDLPQFTPEIFMKVSAKNVMKQKHFHKGIEQLVQEGAI
QYYKTLHTNQIILGAVGQLQFEVFEHRMKNEYNVDVVMEPVGRKIARWIENEDDIQDKMN
TSRSILVKDRYDNFVFLFENEFATRWFEEKFPEIKLYSLL
>ACAKHAOO_01006 Serine protease HtrA-like protein
MDNDKKHVIPREQYRRKRHEYFHNEEREERLEREREQRERLAKKEQEQAKVNEERVKDNM
RKARIEKLTQEEIHQQQHLAKLRSDNESDQELNDTNTHHLTLPEEQQLKNEHKENNDKVT
KPTDEVEKQEKEDNNFTPSKHAEIEPKYSRVEKNKGKQQANINKSEVNHLDKSEQTKKQK
ETKALSDDVLETNESQKIEQKEQKASSNETSNKELNSHTKDKSNEVEDNQDIKKASSQNL
AHSNKSEENGHSEDKPKSNDTMDKVKDFLKLHWLKIVIVVAIILIVILISAIISTINQNS
SIEQSSNNDTKYTTTMKNAETAVKSVVTIENDTPKNITTQTIDKTNTNSNNEVGSGVVYK
AVDDTFFILTNTHIVGSNKRVNITYDDDKTATATVVGRDMWSDIAVLKATIKNKNVLPIK
IGHSKHLKLGESILVVGNPLGNDFKNTVTKGIISGLNRAVPVDFDKDNNNDELVNTFQID
ASVNPGNSGGAVVNRVGELVGLVSLKINMPNIEGMGFAIPIDNAREIAEELEKNGEIQYP
NTGIGIKNVSDLLPYERNLLKVPEDVQNGIVVEILKENGLGKKSGLKIGDVVVELDSKSI
QNNLQYRQIIFNHRQDLKTLSAEIYRKGKSQDIKIKLK
>ACAKHAOO_01028 1,4-dihydroxy-2-naphthoyl-CoA synthase
MTRQWETLREYDEIKYEFYEGIAKVTINRPEVRNAFTPKTVAEMIDAFSRARDDQNISVI
ILTGEGDKAFCSGGDQKKRGHGGYVGEDQIPRLNVLDLQRLIRVIPKPVIAMVRGYAIGG
GNVLNVVCDLTIAADNAIFGQTGPKVGSFDAGYGSGYLARIVGHKKAREIWYLCRQYNAQ
EALDMGLVNTVVPLDQIEDETVQWCKEIMKHSPTALRFLKAAMNADTDGLAGLQQMAGDA
TLLYYTTDEAKEGRDAFKEKRDPDFDQFPKFP
>ACAKHAOO_01033 Bifunctional autolysin
MKKKFNLKFPSMIAITLFGAAFTSQHAHAAEANQNSNNTNNVIDDQQNIENAEQAKKEVT
NSAQNVSGVQTYQNPSDVKASVATVSKTYDAKLDNLATTQQTSQDQQSTQNQSTANTSAT
NSNSELAKVQDNEQEVSTSNKSDQTTNINNTHSNTDANKQTLQASKNALSSSQVQSNDDT
SANSNQNTTQYVNNQIDESSSNSQQENSLTSSNKNQLNSSSVTPSQSNQVAQSNNQIESK
QSSDKKVSTYSNNDNNATQVNVDQAKTSQEANTKNASASKTTQTSTSNSTQSGYSGFRSV
GGKGGPATSVKTVKRYAAKATTSSLPKYKPQVSSSINNYIRSNNLQAPKIEENYSSYFPK
YGYRNGVGKPEGIVVHDTANDNSTIDGEINYMKNNYNSAFVHAFVDGNRIIETAPTDYLS
WGAGPNANGRFINVEIVHTHDYASFAKSMNNYADYAATQLQYYGLTPNSAENDGQGTVWT
HYAISRFLGGTDHSDPHAYLQSHNYTYNELYDLINEKYLIKTGQVAPWGTTSSSSSSSNT
NKGGSSNSGSTTTSPKLTVKSNSGLAQIKTDNSGVYTTVYDDKGKATNQVHNTLSVTKSA
TLGKDKYYLVSDYNSGKVYGWVKQGETVYNTVKSPVKVNQSYNIKSGATLYTVPWGNYNQ
VAGTVSKSNTAPFKATKSQQVGKATYLYGSLNGKNGWVSQAFLSNITKPSSGSKTPTVDN
TLKVSNLKNTLGQVSTKNKGTYTTVYDKNVKANATIGGKTYNITKKATLNNKDYYLISDY
NGNTSRGWVPASEISVKTTTPNKTNATYYDINGNANIYDTPWGTAKQVKATVPSSGKQLK
SIDNLKVGNETYLHGVINNIWGWIKSTDVKQTPKVNAKFAVNTKATKPSTTSTQTVSKVA

QVNANNSGARATVYDKTGKNATKYANRTFNVSKEKTIDGNTYVLLQNTTTNTPLGWFNVK
DLKVQNSSSEQKTSGTYKINNKNNGLYSIAWGTNQQRLDNKDLANKSFKVSKSVVIGNTT
YLYGTVNGKTGWIAKNDLTSSNISSDNGEKYQYEFIINTTNGYYYDDPSSAKATSLKAFN
EQIFQVTNRKVVNGKTWYYGKLSNGKYVWIKDTDLKKELVKYSKSYRTLNQVVSLQQNAY
GAPPQVQRNGYGWSNATYSEIKNAMNTEKLAKDDTLKYQFLRLDRPQNLSVASLNQLLKG
KGVLENQGQAFSQAAATSGINEIYLIAHALIETGNGQSQLAKGANIVNNYVTTKSATKYH
NVFGIAAFDANPLYNGINYAKQAGWNSVSKAIIGGAKFIGKDYIKAGQNTLYKMRWNPDH
PATHQYATDINWANANAQYLKQLYNEIKAVGKYFDISSYSK
>ACAKHAOO_01041 putative quinol oxidase subunit 1
MNFPWDQLLVKGNWMITMAQIGAPFLVIGLIAVITYFKLWKYLYKEWFTSVDHKKIGLMY
LICAVLMFVRGGIDALLLRTQLTIPDNTFLESNHYNEIFSTHGVIMIIFMAMPFVFGLWN
VVVPLQIGARDVAFPVMNNISFWLFFVGMILFNLSFIIGGSPAAGWTNYAPLAGEFSPGP
GVNYYLVAIQISGIGTLMTGINFFVTILRCKTPTMKFMQMPMFTVTTFITTLIVILAFPV
FTVVLALMTFDRVFGTAFFTVADGGMPMLWANFFWVWGHPEVYIVILPAFGIYSEIIPTF
ARKRLFGHQSMVWATAGIAFLSFLVWVHHFFTMGNGALINSFFSISTMLIGVPTGVKLFN
WLLTLYKGRITFESPMLFSLAFIPNFLLGGVTGVMLAMASADYQYHNTYFLVAHFHYTLV
TGVVFACLAGLIFWYPKMMGYKLNEKLNAWCFWLFMIGFNVCFLPQFILGLDGMPRRLYT
YMPSDGWWLLNVISTIGALLMAVGFLFLVVSIVYSHIKAPREATGDNWDGLGRTLEWSTA
SAIPPKYNFAITPDWNDYDTFVDMKEHGRHYLDNHNYKDIHMPNNTHTGVIMGIFMLLGG
FFLIFETIIPAAICLVGILGSLVYQSFVQDHGYHIPASEVAENEARLREARIKEREAVGH
ES
>ACAKHAOO_01042 putative quinol oxidase subunit 2
MSKFKSLLLMFGTLILLSGCSNVEVFNAKGPVASSQKFLIIYSIIFMLVIVAVVLTMFAI
FIFKYSYNKNSETGKMHHNSLIETIWFVVPIIIVIALSIPTVKTLYDYEKPPESKEDPMV
VYAVSAGYKWFFAYPEQKVETVNTLTIPKNRPVVFKLQAMDTMTSFWIPQLGGQKYAMTG
MTMNWTLQADETGTFRGRNSNFNGEGFSRQTFKVHSVDQSEFDSWVKDAKSKKTLSQDEF
DKQLLPSTPNKELTFSGTHMAFVDPAADPEYIFYAYKRYNYVQKDPNFVAEKDLYKDVTD
KPQKPARKVQITNANYKRHGMKPMILGNNDPYDNEFKKEEDHNSKEMEKISKSAKDENAS
KFGSKADNDHGGGH
>ACAKHAOO_01047 Bifunctional protein FolD protein
MVAKILDGKQISKDYRQGLQDQVEALKEKGYTPKLSVILVGNDGASQSYVNSKKKAAEKI
GMISEIVHLDEDTSEEDVLKELDRLNNDDSVSGILVQVPLPKQVSEQKILEAINPEKDVD
GFHPSNIGKLYIDEQTFVPCTPLGIMEILKHADIDLEGKNAVVIGRSHIVGQPVSKLLLQ
ANATVTILHSRTKDMHSHLKDADVIVSAVGQPGLVTKDDVKEGAVIVDVGNTPDENGKLK
GDVEFEEVKEVAGAITPVPGGVGPLTITMVLNNTLLAEKMRRGIE
>ACAKHAOO_01057 Bifunctional purine biosynthesis protein PurH
MKKAILSVSNKSGIVEFAKSLIKLDYELYSTGGTKGALEDASVPVKSVSELTQFPEIMDG
RVKTLHPAVHGGILADRDKPEHLEQLSEQHIDLIDMVVVNLYPFQKTVAKPDVTEAEAIE
NIDIGGPTMLRAAAKNFKHVTTIVHPADYNEVIERIKEDRLDEDFRKELMIKVFAHTNEY
DHAIVSFFKGDSEQLRYGENPQQSARFVRTSNSKHTIAGAKQLHGKALSFNNIKDADSAL
SLVKKFKESAAVAVKHMNPCGVGIGDNIETAFKHAYDADNQSIFGGIIALNRTVTSDLAE
TLHAIFLEVVIAPRFTDEALDILTKKKNIRLLEIDMTIDNREEEFVSVSGGYLVQDKDNF
EVAKEDMKVVTDKAPTDDQWDAMLLGWKVIPSVKSNAVILSNTKQTVGIGAGQMNRVGSA
KIALERAIEINDNVALVSDGFFPMDDTVELAAQHGIKAIIQPGGSIKDQDSIDMANKYGI
AMVTTGMRHFKH
>ACAKHAOO_01062 hypothetical protein
MTNEDAKKAKEVEEKLKEQKEEKTDDIEQTKKDVQDTLD
>ACAKHAOO_01065 hypothetical protein
MGIMNRFSSGVTNKVGNKVLNIEEIKEKSILPTTKEEIAERRAKAETIVKKKSLLSSGMS
VVPIPGLDFGVDIKLMRDIIEDINKIYGLDHKQVNTLGDDVKERILAAAAIQGSSFIGKK
VSSAVLKVIIRDMAKRAAAKQTKWFPVVGQAVSASISYYFMNKLGREHIEKCEKVLHDII
>ACAKHAOO_01067 Phosphoenolpyruvate–protein phosphotransferase
MSKLINGIAASDGVAIAKAYLLVEPDLSFTNEKVTDTDAEIQKFRNALEASKIELTKIRN
NAEKQLGPDKAAIFDAHLLVLDDPELIQPIEDKIANEKVSAPEALDEVTSQFITIFESMD

NEYMRERAADIRDVSKRVLAHLLGVELPNPSMIDESVVIVGNDLTPSDTAQLNKEFVQGF
VTNIGGRTSHSAIMSRSLEIAAVVGTKSVTKEVKQGDMVIVDGITGDVIVDPTEDELIAY
QNKRERFFEDKKELQKLRDAETVTIDGEHAELAANIGTPDDLYGVMENGAEGIGLYRTEF
LYMGRDQMPTEDEQFEAYKKVLETMKDKRVVVRTLDIGGDKELPYLNLPKEMNPFLGYRA
IRLCLDQQDIFRTQLRALLRASAYGKLNIMFPMVATINEFRDAKAILLEEKENLKNDGHD
VSDEIELGIMVEIPSTAALADIFAKEVDFFSIGTNDLIQYTMAADRMSERVSYLYQPYNP
AILRLVKQVIEASHKEGKWTGMCGEMAGDETAIPLLLGLGLDEFSMSATSILKARRQING
LSKNEMSELANRAINCATQEEVKDLVNNISK
>ACAKHAOO_01074 Ribonuclease J 1
MKQLHANEVGVYALGGLGEVGKNTYAIEYKNEIVIIDAGIKFPDDNLLGIDYVIPDYTYL
EQNQDKIVGLFITHGHEDHIGGVPYLLKQINVPIYGGPLALGLIRNKLDEHNLLRNASLN
EITEDSVIKSKHFEISFYLTTHSIPEAYGVIIDTPEGKIVHTGDFKFDFTPVGEPANIAK
MAELGKEGVLCLLSDSTNALVPDFTLSEREVGQNVDKIFRNCKGRIIFATFASNIYRVQQ
AVEAAIKYNRKIVTFGRSMENNIKIGMELGYIKAPPETFVEPNKINNIPKHELLILCTGS
QGEPMAALSRIANGTHKQIKIIPDDTVVFSSSPIPGNTKSINRTINALYKAGADVIHSKI
SNIHTSGHGSQGDQQLMLRLIQPKYFLPIHGEYRMLKAHGQTGVDCGVKEENVFIFDIGD
VLALTHDSARKAGRIPSGNVLVDGSGIGDIGNVVIRDRKLLSEEGLVIVVVSIDFNTNKL
LSGPDIISRGFVYMRESGQLIYDAQRKIKTDVISKLNANPNIQWHQIKSSIIETLQPYLY
DKTARRPMILPVIMKVNED
>ACAKHAOO_01077 hypothetical protein
MKLRKTVAIVAASTILLAGCTTDKKEVKAYNENIQKAFDKEQSINSISKKLNSLEEKKQG
LYKKANSDNEDTRKKAADDILENIKQRQETFDKEVSTLNDSEKQFKKGKSHIDEIKSDDK
KKEVKQLDDAVKNKYKVHDEYASAYKNVLEKEKDLFELIKQDGVTQSQVDEKNDALNKAQ
KNFQNKFKDYSKAMNTVNKEKQDVDNL
>ACAKHAOO_01078 Pyruvate dehydrogenase E1 component subunit alpha
MAPKLQAQFDAVKVLNDTQSKFEMVQILDVDGNVVNEDLVPDLTDEQLVELMERMVWTRI
LDQRSISLNRQGRLGFYAPTAGQEASQLASQYALEKEDFILPGYRDVPQIIWHGLPLTEA
FLFSRGHFKGNQFPEGVNAFSPQIIIGAQYIQTAGVAFGIKKRGKKAVAITYTGDGGSSQ
GDFYEGINFASAYKAPAIFVIQNNNYAISTPRSKQTAATSLAQKAIAVGIPGIQVDGMDA
LAVYQATKEARDRAINGEGPTLIETMTYRYGPHTMAGDDPTRYRTSDEDADWEKKDPLVR
FRKFLENKGLWNEEKENEVIERAKDDIKKAIKEADNTPKQTVIDLMDIMYEDMPQNLAEQ
YEIYKEKESK
>ACAKHAOO_01079 Pyruvate dehydrogenase E1 component subunit beta
MAQMTMVQAINNALKTELQNDENVLVFGEDVGVNGGVFRVTEGLQKEFGEDRVFDTPLAE
SGIGGLALGLTVEGFRPVMEIQFLGFVFEVFDEVAGQIARTRFRSGGSKVAPVTIRAPFG
GGVHTPELHADNLEGILAQSPGIKVVIPSGPYDAKGLLLSSIRSNDPVVYLEHMKLYRSF
REEVPEEEYTIDIGKANVKKEGNDITLIAYGAMVQESEKAAEELEKEGYSVEVIDLRTVQ
PIDIDTLVASVEKTGRAVVVQEAQRQAGVGATVAAELAERAILSLEAPIARVAAADTVYP
FTQAENVWLPNKNDIIEKAKATLEF
>ACAKHAOO_01080 Dihydrolipoyllysine-residue acetyltransferase
component of pyruvate dehydrogenase complex
MAFEFRLPDIGEGIHEGEIVKWFVKAGDTIEEDDVLAEVQNDKSVVEIPSPVSGTVEEVV
VDEGTVAVVGDVIVKIDAPDAEDMQFKGGHDDDASSEEAPAQEEAKTEEAPAASASQDEE
VDENRQIKAMPSVRKYAREKGVNIKAVAGSGKNGRITKEDIDNHLNGGGAQAATASNESA
AASTSEDTSAVQTQSVPEGDFPETTEKIPAMRRAIAKAMVNSKHTAPHVTLMDEIDVQDL
WDHRKKFKEVAAEQGTKLTFLPYVVKALVSALKKYPALNTSFNEEAGEIVHKHYWNIGIA
ADTDRGLLVPVVKNADRKSIFQISDEINELAVKARDGKLTSDEMKGATCTISNIGSAGGQ
WFTPVINHPEVAILGIGRIAQKPIVKDGEIVAAPVLALSLSFDHRQIDGATGQNAMNHIK
RLLNNPELLLMEG
>ACAKHAOO_01081 Dihydrolipoyl dehydrogenase
MVVGDFPIETDTIVIGAGPGGYVAAIRAAQLGQKVTIVEKGNLGGVCLNVGCIPSKALLH
ASHRFVEAQHSENLGVIAESVSLKFDKVQEFKKSVVNKLTGGVEGLLKGNKVEIVKGEAY
FVDNNSLRVMDEKSAQTYNFKHAIIATGSRPIEIPNFEFGKRVIDSTGALNLQEVPGKLV
VVGGGYIGSELGTAFANFGSDVTILEGAKEILGGFEKQMVQPVKKGMKEKGVEIITEAMA

KNAEETENGVKVTYEAKGEEKTIEADYVLVTVGRRPNTDELGLEELGLKFADRGLLEVDK
QSRTSIDNIYAIGDIVPGLPLAHKASYEAKVAAEAISGQASEVDYIGMPAVCFTEPELAQ
VGYTEAQAKEEGLDYKASKFPYAGNGRALSLDDTTGFVKLITLKEDDTVIGAQIAGNGAS
DIISELGLAIEAGMNAEDIALTVHAHPTLGEMSMEAAEKAIGMPIHTM
>ACAKHAOO_01088 hypothetical protein
MSGERYTQIKRPVSRLTEKLLGWFSWIFLLILTVITMFIALVSFSNDTSIQNLENSMNSN
ELIQQILTNNSLNTTQFVIWLQNGVWAIIVYFIVCLLISFLALISMNMRILSGFLFLIAS
IITLPLVLLFVTLIIPIFFFIIAIMMFARKSKVETVPMYGPSHGYENQYYPRDEYDDRYY
GSNRNINDDYDYDDHNQYNDYDEDYVESPPKTKKSERRTRRKKPYYNDDIDSNYRDNNYD
ETTIRKDEEQDLATDTEEDKYNQYPKRAITGEYQSDADDVEATGVLSRQAKYNKKANKKS
QFDNTSDEESYDFAENVVDTEPKVDKREEKAQRKREKAELKAKKKEKRKAYNQRMKERRK
NQPSAVSQRRMNYEERKQILNKDDIESEKDVNNQEEDNKN
>ACAKHAOO_01091 hypothetical protein
MTQYTFKPKDFKAFEVEGLDARMEALNEYVRPQLNQLGDYFSEYFTSQTGETFYAHVAKH
ARRSVNPPVDTWVAFAPNKRGYKMLPHFQIGLFKDHLFLMFGVMHEGKDKAERVKVFDKH
FDVLKQLPEDYQVSLDHMKPEKSYIKDLSDDELHKAIDRVKNVKKGEFFVARSLSPKDAE
LKSDKAFLSFVKETFDEFLKFYE
>ACAKHAOO_01094 GTP-binding protein TypA/BipA
MTNRREDVRNIAIIAHVDHGKTTLVDELLKQSGIFRENEHVDERAMDSNDLERERGITIL
AKNTAIDYKGTRINILDTPGHADFGGEVERIMKMVDGVVLVVDAYEGTMPQTRFVLKKAL
EQDLKPVVVVNKIDKPSARPEGVVDEVLDLFIELEANDEQLDFPVVYASAVNGTASLDSE
KQDENMQSLYETIIDYVPAPLDNHDEPLQFQVALLDYNDYVGRIGVGRVFRGKMRVGDNV
SLIKLDGTVKNFRVTKIFGYFGLKREEIEEAQAGDLIAVSGMEDINVGETVTPTDHQEAL
PVLRIDEPTLEMTFKVNNSPFAGREGDYVTARQIQERLDQQLETDVSLKVTPTDSPDTWV
VAGRGELHLSILIENMRREGFELQVSKPQVILREIDGVLSEPFERVQCEVPSENAGSVIE
SLGARKGEMLDMSTTDNGLTRLIFMVPARGMIGYTTEFMSMTRGYGIINHTFEEFRPRVK
AQIGGRRNGALISMDQGQATAYAIINLEDRGVNFMEPGTEVYEGMIVGEHNRENDLTVNI
TKAKHQTNVRSATKDQTQTMNRPRILTLEEALEYINDDELVEVTPQSIRLRKKILNKSLR
EKEAKRVKQMMQDE
>ACAKHAOO_01099 2-oxoglutarate carboxylase small subunit
MKNIKKLLVANRGEIAIRIFRAATELNIKTVAIYSNEDKNALHRYKADESYLVGKDLGPA
ESYLNIERIIDVAKRAGVDAIHPGYGFLSENKQFAQRCDEEGIKFIGPHIEHLDMFGDKV
KARTTAINAKLPVIPGTDGPIENFEAAKAFAQEAGFPLMIKATSGGGGKGMRIVREEGEL
EDAFHRAKSEAEKSFGNSEVYIERYIDNPKHIEVQIIGDEYGNIVHLYERDCSVQRRHQK
VVEVAPSVGLSKELRERICDAALQLMNNIRYVNAGTVEFLVSGDEFFFIEVNPRVQVEHT
ITEMITGIDIVKTQILVADGANLFDERVSMPQQEEIQTLGYAIQCRITTEDPSNDFMPDS
GTIIAYRSSGGFGVRLDAGDGFQGAEISPYYDSLLVKLSTHAVTFKQAEEKMERSLREMR
IRGVKTNIPFLINVMRNDQFRSGDYTTKFIEETPELFNIAPTLDRGTKTLEYIGNVTING
FPNVEKRLKPDYESTAIPQVPHSKINTLNGTKQLLDSKGPKAVADWVREQNDVLVTDTTF
RDAHQSLLATRVRTKDMMNIASKTAEVFKDSFSLEMWGGATFDVAYNFLKENPWERLERL
RKAIPNILFQMLLRASNAVGYKNYADNVIEKFVKESANAGVDVFRIFDSLNWVDQMKVAN
EAVQNAGKISEGAICYTGDILNPERSNVFTLEYYVKLAKELEREGFHILAIKDMAGLLKP
KAAYELIGELKSAINLPIHLHTHDTSGNGLLIYKEAIDAGVDIIDTAVASMSGLTSQPSA
NSLYYGLNGFNRNLRADIEGLEELSHYWSTVRPYYSDFESDIKSPNTEIYHHEMPGGQYS
NLGQQAKSLGLGERFHEVKDMYRRVNFLFGDIVKVTPSSKVVGDMALYMVQNDLDEQSVI
KEGHKLDFPESVVSYFKGDIGQPVNGFNKELQDVILKGQQPLTERPGEYLDPVNFDEIRQ
ELEAKDYGEVTEQDVISYVLYPKVFDQFMQTKQQYGDLSLLDTPTFFFGMRNGETVEIEI
DTGKRLIIKLETISEPDENGYRTIYYVMNGQARRISIKDENIKTNTNLKPKADKSNPSHI
GAQMPGSVTEVKVTVGEEVKVNQPLLITEAMKMETTIQAPFNGTIKQVTVVNGDAIATGD
LLIEIEKAD
>ACAKHAOO_01105 Glycerophosphodiester phosphodiesterase
MTIKKRWVLNLSLASVSFLGSLFFINKSSKADQTKEIPKFFNGKAPYIFAHRGGMAVRPE
QTQLAFDNAVANNLDGFETDVRLTKDEKLIVFHDATVDRTTNGSGKVSDHRVSELKRLDA
GYHFKDINGQFPYRGHQKAKILTFDELLKMYPNMYINVDLKDAPDSYEGTVAPTKMYEDI

INNQAQDRVLVTSFYKEQNVRFREISKGQVAIGASQKEVAEGFIKFNLGLGNKYQPIADT
FQMPTQFKGIPLTSKRFIQWLNLLNIVPGFYGINSTDLMTDLYHKGVHTLVTDRPDLGKQ
FKSSLNIEK
>ACAKHAOO_01115 Phenylalanine--tRNA ligase beta subunit
MLISNEWLKDYVDAGVKVEDLAERITRTGIEVDDMIDYSKDIKNLVVGYIQSKEKHPDAD
KLNICQVDIGEEEPVQIVCGAPNVDAGQHVIVAKVGGRLPGGIKIKRAKLRGERSEGMIC
SLQEIGISSNVVPKAYENGIFVFPTEVEPGTDALTALYLNDQVMEFDLTPNRADALSMVG
TAYEVAALYQTEMTKPETQSNETSESATNELSVTIDNPEKVPYYSARVVKNVSIEPSPIW
VQARLIKAGIRPINNVVDISNYVLLEYGQPLHMFDQDHIGSKEIVVRQAKDEETMTTLDN
NERKLVDTDIVISNGQEPIALAGVMGGDFSEVTEQTTNVVIEGAIFDPVSIRHTSRRLNL
RSEASSRFEKGIATEFVDEAVDRACYLLQELASGEVLQDRVSSGDLGSFVTPIDITAEKV
NKTIGFNLSNDEIQSIFRQLGFETTLKGETLTVNVPSRRKDITIKEDLIEEVARIYGYDE
IPSSLPVFGEVTSGELTDRQHKTRTLKETLEGAGLNQAITYSLVSKDHAKDFALQERPTI
SLLMPMSEAHATLRQSLLPHLIEATAYNVARKNKDVRLYEIGRVFFGNGEGELPDEVEYL
SGILTGEYVVNAWQGKKEEIDFFIAKGVVDRVAEKLNLEFSYKAGKIEGLHPGRTAIVSL
EGQDIGFIGELHPQVAADNDLKRTYVFELNYDAMMQVAVGYINYEQIPKFPGVTRDIALE
VNHDVPSSELKQIIHNNGEDILQSTLVFDVYEGEHLEKGKKSVAIRLNYLDTEDTLTDER
VSKIHDKILEALQAQGATIR
>ACAKHAOO_01119 DNA polymerase/3'-5' exonuclease PolX
MTKKDVIQLLEKIAIYMELNGENTFKVSAYRKAAQSLEIDERPLDEIEDVTELKGIGKGV
GEVIDEYRKTGESSYLKELQQLVPEGLIPLMKIQGLGSKKIAKLYKELNITDKESLQLAC
ESGKVSELSGFAKKTEQNILEAVKALGAKKDSYPIDTIRGLNGIIIDYLETIDSIERYSE
AGSFRRYKEMSKDLDYIISTEHPDEVQQQLLNIPNKVKDVVVGQTKVSLELTYDDETIGV
DFRLIEPVAFYHTLQHFTGSKDHNIRIRQMAKAQNEKISEYGIEQQDGSLLQLQSEEEIY
QHFGKEWIAPALREDGSEFDKDLSDIITLDDINGDIHMHTTYSDGAFSIRDMIEANINKG
YQFMVITDHSQSLKVANGLQVERLLRQNEEIKKLNEEYSEIDIYSGTEMDILPDGSLDYD
DDVLSQLDYVIAAIHQSFNQSEEEIMKRLENACRNPYVRHIAHPTGRIIGRREGYKPNID
RLMKLAEETNTIMEINANPKRLDLNAETVKKYPNVTLTINTDAHHIEHLDFMKYGVATAQ
KGFVNKDRVINTMSRQAFKDFVGNNKKLKK
>ACAKHAOO_01121 Thioredoxin
MAIVKVTDSNFDENIQSGVKLVDFWATWCGPCKMIAPVLEELAGDYDGKADILKLDVDEN
PSTAAKFEVMSIPTLIVFKDGEPVDKVVGFQPKENLAEVLDKHL
>ACAKHAOO_01143 UDP-N-acetylmuramoylalanine--D-glutamate ligase
MLNYTGLENKDVLVVGLAKSGYEAAKLLIKLGANVTVNDGKDLSQDPHAKDLEALGVKIV
DGGHPLSLLDNEPIIVKNPGIPYTVSIIQEAYQRHLKILTEVELSYLISEAPIIAITGTN
GKTTTSSLIGDMFKKSRLTGRLSGNIGYVASKVAQETSPNEYLITELSSFQLLGVEQYRP
HIAIITNIYSAHLDYHETLENYQNAKKQIYKNQTENDYLICNYHQRHLIESENLKAKTLY
FSTQQEVDGIYIKDGFIVYQGIRIINIDDLVLPGEHNLENILAAVLASILAGVPIKAIID
SLTTFSGIDHRLQYIGTNRTNKYYNDSKATNTLATQFALNSFKQPIVWLCGGLDRGNDFD
ELIPYMKNVRVMVVFGQTQEKFAKLGNSQGKLVVKATDIEDAVKKVQDVVEPNDVVLLSP
ACASWDQYKTFEERGERFIESFRAHLPSY
>ACAKHAOO_01145 Cell division protein FtsA
MEEHYYVSIDIGSSSVKTIVGEKFHNGINVIGTGQTYTSGIKNGLIDDFDIAKQAVKDTI
KKASIASGVDIKEVFLKLPIIGTEVYDEANEIDFYEDTEINGTHIEEVLEGIRQKNDVAD
TDVIDVFPIRFIVDGDNEVSDPKELIARHSLRVEAGVIAIHKSILINMIKCVESCGVDVL
DVYSDAYNYGSILTATEKELGACVIDIGEDLTQIAFYERGELVDADSIELAGRDITDDIA
EELNTTYETAEKIKHQYGHAYANSASDQDVFSVDQVDSDELVEYTQKDLSAVIERTMQDI
FDEVFDVLVDLGLTRVNGGFIVTGGSSNLLGVKELLNTMVNEKIRVHTPSQMGIRKPEFT
SAISTISSSITFDELLDYVTMSYQDNDEFEEEVIEDNEREHTSKSGGFDWFKRKSNKNEN
TYEEDYNEPHERTDKVDDDDKYDSNESHHDTTEHYDDQEQPQKEEGKFKKLMKSLFE
>ACAKHAOO_01146 Cell division protein FtsZ
MLEFEQGFNHLATLKVIGVGGGGNNAVNRMIDHGMNNVEFIAINTDGQALNLSKAESKIQ
IGEKLTRGLGAGANPEIGKKAAEESREQIEDAIQGADMVFVTAGMGGGTGTGAAPVVAKI
AKEMGALTVGVVTRPFGFEGRKRSTQAAAGVESMKAAVDTLIVIPNDRLLDIVDKSTPMM

EAFKEADNVLRQGVQGISDLIAVSGEVNLDFADVKTIMSNQGSALMGIGVSSGENRAVEA
AKKAISSPLLETSIVGAQGVLMNITGGESLSLFEAQEAADIVQDAADEDVNMIFGTVINP
ELQDEIVVTVIATGFEDKPTSQGRKASSTGFGTSATSSTSTQTSTPREESFSQSSSSSRS
SESVSERSHTTKDDDIPSFIRNREERRSRRTRR
>ACAKHAOO_01150 Cell division protein SepF
MAIKDLFNNFFLMDDEEEVESPEERQRRVVQNEENETNNVQQNQPQQSERSYSNQSKLKT
VPQKKTTRNYNSEERNVRMNQPPSKSNGKNVVTMNQTSQSYSGYESSKMCLFEPRVFSDT
QDIADELKNRRATLVNLQRIDKISAKRIIDFLSGTVYAIGGDIQRVGTDIFLCTPDNVEV
AGSITDHIEQMESQHYE
>ACAKHAOO_01153 Septum site-determining protein DivIVA
MPFTPSEIKNKEFTKVKNGLEPAEVSDYLNQLSNEIERLKEEKKQLEKVIEERDTNIKSY
QQVHQSVSDALVQAQQAGEETKLAANKEAEAVISKAQAQADLIVNDAIEKARHLSFQTED
MKRQSKIFRSRFRMLVEAQLDLLKNDDWDYLLNYDIDAEKVTQENFQHLNSQDITPEEQL
SAQSQQSESVLASESNNSKINSLSTSDATSTSENVTSQSSVSQSESTSNK
>ACAKHAOO_01154 Isoleucine--tRNA ligase
MNYKDTLLMPKTDFPMRGGLPNKEPQIQEQWEANNQYQKALEKNKGNQSYILHDGPPYAN
GNLHMGHALNKIIKDIIVRYKTMQGFYAPYVPGWDTHGLPIEQALTKKGVDRKKMSIAEF
REKCKEFALEQIELQKKDFKRLGVRGDFNDPYITLKPEYEAAQIRLFGEMADKGLIYKGK
KPVYWSPSSESSLAEAEIEYHDKRSASIYVAFDVKDTKGVVDQDAQFIIWTTTPWTIPSN
VAITVHPDLKYGQYNVNGKKYIIAQALSEDVAEALEWDKDAIQLEKEFTGKELEYVEAQH
PFLDRISLVINGNHVTTDAGTGCVHTAPGHGEDDYIVGQKYDLPVISPLDDKGVFTEEGG
QFEGMFYDKANKAVTDLLTEKDALLKLNFITHSYPHDWRTKKPVIFRATPQWFASINKVR
QDILDAIEETDFKVDWGKTRIYNMIRDRGEWVISRQRVWGVPLPVFYAENGDIIMTKETV
NHVADLFEEHGSNIWFEREAKDLLPEGFTHPGSPNGTFTKEMDIMDVWFDSGSSHRGVLE
NRPELSFPADLYFEGSDQYRGWFNSSITTAVATRGQAPYKFLLSHGFVMDGEGKKMSKSL
GNVIVPDQVVKQKGADIARLWVSSTDYLADVRISDEILKQTSDVYRKIRNTLRFMLGNIN
DFNPDTDVIPEAELLEVDRYLLNRLREFTASTIEHYDNFDYLNIYQEVQNFINVELSNFY
LDYGKDILYIEEKNAHKRRSMQTVLYQILVDMTKLLAPILVHTAEEVWTHTPHVKEESVH
LADMPKVVEVDQALLDKWNQFMALRDDVNRALEVARNNKVIGKSLEAKVVIGNNDNFKAA
EFLQQFEDLQQLFIVSQVEVSDSVDNAEAYQHGDIRIDHAVGEKCERCWNYTEELGSVGE
LEHLCPRCQEVVKTLV
>ACAKHAOO_01163 Dihydroorotase
MKLIKNAQILDNGELKQASILIDGQHIKEINNNIEVDENVEVIDAQGHFVAPGLIDVHVH
LREPGGEHKETIKTGTKAAARGGFTTVCPMPNTRPVPDSVEHMDRLQQLIQDNAQVRVLP
YASITVRQAGKEHVDFDALAKQGAFAFTDDGVGVQESAMMYEAMQQAAKVNKAVVAHCED
NSLIYGGAMHQGKRSEELGIPGIPNICEAVQIARDVLLAEAANCHYHVCHVSTKESVRAI
RDAKAAGIHVTAEVTPHHLLLTEDDVPGDNAIFKMNPPLRSKEDRDALLEGLLDGTIDCI
ATDHAPHAKEEKAQPMTKAPFGIVGSETAFPLLYTHFVKNGDWSLQQLVDYLTIKPAQTF
DLPYGKLEEGCFADLTIINLDEEREIKGEDFLSKADNTPFIGYKVYGNPILTMVEGEVKF
KEDK
>ACAKHAOO_01165 Carbamoyl-phosphate synthase large chain
MPKRDDIQTILVIGSGPIIIGQAAEFDYAGTQACLALKEEGYRVILVNSNPATIMTDKEI
ADKVYIEPLTHDFIARIIRKEQPDALLPTLGGQTGLNMAIQLHDSGVLEANNVKLLGTEL
KSIQQAEDRELFRSLMNDLNVPVPESDIVNTVEQAFAFKDEVGYPLIVRPAFTMGGTGGG
ICYNDEELKEVVSNGLHYSPATQCLIEKSIAGYKEIEYEVMRDKNDNAIVVCNMENIDPV
GIHTGDSIVVAPSQTLSDVEYQMLRDVSLKVIRALGIEGGCNVQLALDPHSFNYYIIEVN
PRVSRSSALASKATGYPIAKLAAKIAVGLTLDEMLNPVTGTSYAAFEPTLDYVISKIPRF
PFDKFEKGERELGTQMKATGEVMAIGRTYEESLLKAIRSLEYGVHHLGLPNGESFDLDYI
KERISHQDDERLFFIGEAIRRGTTLEEIHNMTQIDYFFLNKFQNIINIEHELKNHPGDLD
YLKYAKDYGFSDRVIAHRFGMTESEVYQLRQDNNIKPVYKMVDTCAAEFESTTPYYYGTY
ETENESIVTDKEKILVLGSGPIRIGQGVEFDYATVHAVWAIQNAGYEAIIVNNNPETVST
DFSISDKLYFEPLTEEDVMNIINLEQPKGVVVQFGGQTAINLADKLAKHGVKILGTTLEN
LNRAEDRKEFEALLHTIDVPQPKGKTATSPKEALENAREIGYPVVVRPSYVLGGRAMEIV
NSDAELENYMEQAVKASPEHPVLVDRYLTGKEIEVDAISDGETVIIPGIMEHIERAGVHS

GDSIAVYPPQTLSQEDIDTLEDYTIKLAKGLNIVGLINIQFVIAHDGVYVLEVNPRASRT
VPFLSKITDIQMAQLAMQAIMGTRLKDLGYKQGVQPYSEGVFVKAPVFSFNKLKNVDVTL
GPEMKSTGEVMGKDLTLEKALYKGLTGSGVEVKDHGTVLMTVSDKDKDEIVKIAHRLNEV
GYKILATQGTAEKLKEDNIPVEVVGKIGGDDDLLTRIQNGEVQIVINTMTKGKEVERDGF
QIRRTTVENGVPCLTSLDTANALTNVIESMTFSMRTM
>ACAKHAOO_01186 putative protein
MTIEISNDYGKIDISNEVIASVVGSKAVESYGIVGMASRQQVRDGIAEILGHENYARGIE
VKENNGVIDIDMYIIVSYGVKISEVANNVQSSVKYTLENSLNVKVNSINIFVQGVRLNNK
GK
>ACAKHAOO_01187 putative protein
MISKINGKLFADMIIQGAQNLSNNADLVDSLNVYPVPDGDTGTNMNLTITSGREEVENNL
SQSIGELGKTFSKGLLMGARGNSGVILSQLFRGFCKNIETENEINAQQLASSFQAGVDTA
YKAVMKPVEGTILTVAKDAAKAAVNKAEETDDCVEVMEYTIAEAEKSLNNTPNLLAVLKE
VGVVDSGGKGLLCVYEGFLKGLTGQTIEAKKEKLNTEELVHEEHDFHGVINTEDIKYGYC
TEMMVRFGKDKRAFDEQTFRTDMSQFGDSLLVINDDEIVKVHVHTETPGEVFNYGQEYGE
LIKLKVENMREQHREVIRKEKLNHHSDEQEESKTVETAIIAISMGDGISELFTSMGATHI
ISGGQTMNPSTEDIVKVIEQSQCKRAIILPNNKNIRMSSDQAATLVEADTVVIPTTSIPK
GIAALFQYDPSSSLEDNHSHMTTALESVKSGSVTFAVRDTKIDGVEIKKGEFMGLSESKI
VTSNDDEFVTVTGLLKSMLNEDSEILTIIAGEDANDDISDKLVEWVESEYPDVEVEEHNG
GQPIYQYLFSVE
>ACAKHAOO_01191 Phosphate acyltransferase
MVKIAIDMMGGDDAPGIVLEAVEKAVNDFKDLEIILFGDQNQYTLNHERIEFRHCSEKIE
MEDEPVRAIKRKKDSSMVRMAEAVKNGEADGCVSAGNTGALMSAGLFIVGRIKGVARPAL
VVTLPTIDGKGFVFLDVGANADAKPEHLLQYAQLGNIYAKKIRGIQNPKVSLLNIGTEPA
KGNTLTKKSYQLLKDDQSFNFDGNIEAKTLMEGNTDVVVTDGYTGNMVLKNIEGTAKSIG
KMLKETFLGSLKNKLAALVLKKDLDTFTKKMDYAEYGGSVLLGLDGTVVKAHGGSNARAF
YSAIRQAKIAGDEKIVDIMRETVGGKDE
>ACAKHAOO_01193 3-oxoacyl-[acyl-carrier-protein] reductase FabG
MTNKNALVTGASRGIGRSIALQLAEDGFNVAVNYAGNKEKAEAVVSEIKAKGVESFAIQA
NVAEGDEVKAMIKEVVSQFGSLDVLVNNAGITRDNLLMRMKEHEWDDVINTNLKGTFNCI
QKATPQMLRQRGGAIINLSSIVGAMGNPGQANYVATKAGIEGLTKSSARELASRGITVNA
VAPGFIVSDMTDALSDDLKSQMLEQIPLSRFGEDTDIANTVAFLASDRAKYITGQTIHVN
GGMYM
>ACAKHAOO_01194 Acyl carrier protein
MENFDKVKDIIVDRLGVDADKVTEDASFKDDLGADSLDIAELVMELEDEFGTEIPDEEAE
KINTVGDAVKYINSLEK
>ACAKHAOO_01197 Signal recognition particle receptor FtsY
MSFFKRLKDKFSTKSTEDIEQELSEEEGNQSSTIHDDNDEAQIEPKKKPRKLSEADFDDD
GLISIEDFEEIEAQKMGAKFKAGLEKSRQNFQEQLNNLIARYRTVDEDFFEALEEMLITA
DVGFNTVMQLTEELRTEAQRRNIQETEDLREVIVEKIVEIYHQEDDQSEAMNLEDGRLNV
ILMVGVNGVGKTTTIGKLAHRYKMEGKKVMLAAGDTFRAGAIDQLQVWGDRVGVEVIRQS
EGSDPAAVVYDAINSAKNKGVDILICDTAGRLQNKSNLMQELDKMKRVISRAVPDAPHEA
LLCLDATTGQNALSQARSFKEVTNVSGIVLTKLDGTAKGGIVLAIRNELHIPVKYVGLGE
KLDDLQPFNPESYVYGLFADMIEQNEDIPDEVAELDFNNEDNPNGEK
>ACAKHAOO_01199 Signal recognition particle protein
MAFEGLSDRLQATIQRMRGKGKVTEADIKAMMREVRLALLEADVNFKVVKEFVKTVSDRA
LGSDVMQSLTPGQQVIKIVQDELTQLMGGENSTIKMANKPPTVVMMVGLQGAGKTTTAGK
LALLMRKKYNKKPMLVAADIYRPAAINQLQTVGKQLDVPVYSEGDQVKPQQIVENALKHA
KEEHLDFVIIDTAGRLHIDEALMNELQEVKEISKPNEIMLVVDAMTGQDAVNVAQSFDDQ
LDVTGVTLTKLDGDTRGGAALSIRSVTQKPIKFVGMSEKMDGLELFHPERMASRILGMGD
VLSLIEKAQQDVDQEKAKDLEQKMRNSTFTLDDFLEQLDQVKNLGPLDDIMKMIPGMNKM
KGMDKLNMDEKQIDHIKAIIQSMTPSERENPAVLNVSRKKRIAKGSGRSLQEVNRLMKQF
NDMKKMMKQFSGAGRKGKKGKRSQMENMLKGMNLPF
>ACAKHAOO_01200 30S ribosomal protein S16

MAVKIRLTRLGSKRNPFYRIVVADARSPRDGRIIEQIGTYNPASVNAPEVKIDEELALKW
LKDGAKPTDTVHNILSKQGILKTFDEQKHAK
>ACAKHAOO_01203 50S ribosomal protein L19
MSNHKLIEAVTKSQLRTDLPTFRTGDTLRVHVRIVEGSRERIQVFEGVVIKRRGGGISET
FTVRKISSGVGVERTFPLHTPKIEKIELKRRGKVRRAKLYYLRSLRGKAARIQEIR
>ACAKHAOO_01208 Succinate--CoA ligase [ADP-forming] subunit beta
MNIHEYQGKEIFRSMGVAVPEGRVAFTAEEAVEKAKELDTEIYVVKAQIHAGGRGKAGGV
KIAKSLSEVETYANELLGKQLVTHQTGPEGKEVKRLYIEQGCDIQKEYYVGFVIDRATDR
ITLMASEEGGTEIEEVAAKTPEKIFKETIDPVVGLSPYQARRIAFNINIPKESINKAAKF
LISLYNVFIEKDCSIVEINPLVTTGEGEVLALDAKINFDDNALFRHKDIQELRDLEEEDP
KEIEASKYDLSYIALDGDIGCMVNGAGLAMATMDTINHFGGNPANFLDVGGGATKEKVTE
AFKIILGDDNVKGIFVNIFGGIMKCDVIAEGIVAAVKEVELTLPLVVRLEGTNVERGKEI
LNESGLAIEPAATMAEGAQKIVKLVKEA
>ACAKHAOO_01209 Succinate--CoA ligase [ADP-forming] subunit alpha
MSVFIDKNTKVMVQGITGSTALFHTKQMLDYGTQIVAGVTPGKGGQVVEGVPVYNTVAEA
KEETGANVSVVYVPAPFAADSILEAADADLDMVICITEHIPVVDMVKVKRYLEGRKTRLV
GPNCPGVITADECKIGIMPGYIHKKGHVGVVSRSGTLTYEAVHQLTEEGIGQTTAVGIGG
DPVNGTNFIDVLKAFNEDEDTKAVVMIGEIGGTAEEEAAEWIKANMTKPVIGFIGGQTAP
PGKRMGHAGAIISGGKGTADEKIKTLNECGVKTADTPSEIGTTLIEAAKEAGIYEELLTI
K
>ACAKHAOO_01212 DNA topoisomerase 1
MADNLVIVESPAKAKTIEKYLGKKYKVIASMGHVRDLPRSQMGVDVEDNYEPKYITIRGK
GPVVKDLKKYAKKAKNVFLASDPDREGEAIAWHLSKILELDDSKENRVVFNEITKDAVKE
SFKHPRGIEMDLVDAQQARRILDRLVGYNISPVLWKKVKKGLSAGRVQSVALRLVIDREN
EIRNFKPEEYWSIEGEFRYKKSKFTAKFLHYKNKPFKLKTKKDVEKVTAELDGDKFEITN
VNKKEKTRNPANPFTTSTLQQEAARKLNFKARKTMMLAQQLYEGIDLKRQGTVGLITYMR
TDSTRISQTAKDEAKQYIEDKYGKDYLSNRTAKGKQGDQDAHEAIRPTSTLRTPYEMKAY
LTRDQHRLYKLIWERFVASQMAPAILDTVALDVTQNNIKFRANGQTIKFKGFMTLYVEAK
DDKDNEKENKLPNLSKGDEVTATQIEPAQHFTQPPPRYTEARLVKTLEELKIGRPSTYAP
TIDTIQKRNYVKLESKRFVPTELGEIVYEQVKDYFPEIIDVEFTVNMETLLDKIAEGDIG
WRKVIDNFYGSFKLDVERAEEEMEKVEIKDEPAGEDCEVCGSPMVIKMGRYGKFMACSNF
PDCRNTKAIVKTIGVTCPKCKDGDVVERKSKKNRLFYGCSNYPECDFISWDKPVGRDCPK
CNHYLMEHKKGRSSQVICSNCDYKEEVQK
>ACAKHAOO_01216 ATP-dependent protease ATPase subunit HslU
MDANGIKLTPKDIVSKLDEYIVGQDDAKRKVAIALRNRYRRSLLDEETKQEIAPKNILMI
GPTGVGKTEIARRMAKVVGAPFIKVEATKFTEVGYVGRDVESMVRDLVDVAVRLVKDQKK
GLVKDEAVNKANEKLVKLLVPSMKKKASNNSNPLESLLGGAIPNFGNNDDEEEETPTEEI
KTKRSEIKKQLLDGKLEEEKVRIKVEQDPGALGMLGTNQNQQMQDMMNQLMPKRKVEREV
PVKTARKILADDFADELIDQETANQEALELAEQMGIIFIDEIDKVATNNANSGQDVSRQG
VQRDILPILEGSMIQTKYGTVNTEHMLFIGAGAFHVSKPSDLIPELQGRFPIRVELESLT
VDDFYRILTEPKLSLIKQYEALLQTEEVTVNFTKEAITRLAEMAYQVNQDTDNIGARRLH
TILEKMLEDLSFEAPSMPNAVVDITPQYVDDKLKSISTNKDLSAFIL
>ACAKHAOO_01217 GTP-sensing transcriptional pleiotropic repressor CodY
MSLLSKTRELNTLLQKHKGIAVDFKDVAQTISSVTVTNVFIVSRRGKILGSSLNELLKSQ
RINEMLESKHIPSEYTELLMDVKQTESNIDIDNELTVFPPEDKEVFSSSRTTVFPILGGG
ERLGTLVLGRVKDDFNENDLVLGEYAATVIGMEILREKHNEVEKEARDKAAITMAINSLS
YSEKEAIEHIFEELGGNEGLLIASKVADRVGITRSVIVNALRKLESAGVIESRSLGMKGT
FIKVKKEKFLDELERNK
>ACAKHAOO_01218 30S ribosomal protein S2
MAVISMKQLLEAGVHFGHQTRRWNPKMKKYIFTERNGIYIIDLQKTVKKVEEAYNFIKQV
SEDGGRVLFVGTKKQAQESVKAEAERAGQFYVNQRWLGGILTNYKTISKRIKRISEIEKM
EEDGLFDVLPKKEVVELKKEYDRLIKFLGGIRDMKSMPQALFVVDPRKERNAIAEARKLN
IPIVGIVDTNCDPDEIDYVIPANDDAIRAVKLLTGKMADAVLEGQQGVSNDEVAAEQNIN

LDEKEESQEAESTEENTTVESN
>ACAKHAOO_01219 Elongation factor Ts
MAISAKLVKELRERTGAGMMDCKKALTETDGDIDKAIDYLREKGIAKAAKKADRIAAEGL
VHVEVKGNEAAIVEINSETDFVARNEGFQELVKEIANQVLDSKAETVDALLETKLSSGKT
VDERMKEAISTIGEKLSIRRFEIRTKSDNDAFGAYLHMGGRIGVLTVVEGSTDEEAAKDV
AMHIAAINPKYVSSEQVKEEEINHEREVLKQQALNEGKPENIVEKMVEGRLRKYLQEICA
VDQNFVKDPDQTVEAFLKSKGGKLVDFVRYEVGEGMEKREENFADEVKGQMK
>ACAKHAOO_01220 Uridylate kinase
MSSLLVYITYFYNREEKTMAQTSKYKRVVLKLSGEALAGDKGFGINPIIIKSVAQQVAEV
AKMDCEIAVIVGGGNIWRGKTGSDLGMDRGTADYMGMLATVMNALALQDSLEQLECDTRV
LTSIEMKQVAEPYIRRRAIRHLEKKRVVIFAAGIGNPYFSTDTTAALRAAEVEADVILMG
KNNVDGVYSADPKVDKNAVKYEHLTHIQMLQEGLQVMDSTASSFCMDNNIPLNVFSIMEE
GNIKRAVMGEKIGTLITK
>ACAKHAOO_01221 Ribosome-recycling factor
MSDIIQDTKARMSKSIDNLSRELANISAGRANSNLLSGVTVDYYGAPTPVQQLASINVPE
ARLLVISPYDKSSVADIEKAIYAANLGVNPTSDGEVIRITVPALTEERRKELVKNVKKIG
EDAKVSIRNIRRDINDQLKKDEKNGDITEDDLRSQTEDVQKATDNSIKEIDQLVEDKEKD
IMSV
>ACAKHAOO_01224 Putative zinc metalloprotease
MSYLITIVSFMIVFGVLVTVHEYGHMFFAKRAGIMCPEFAIGMGPKIFSFRKNETLYTIR
LLPVGGYVRMAGDGLEEPPVEPGMNVKVKLNDKDEITHIILDDQHKFQKIEAIEVKQCDF
KDDLYIEGITSYDNERHHFNIAEKAYFVENGSLIQIAPRHRQFAHKKPLPKFLTLFAGPL
FNFILALILFIALAYFQGTPTTSVGQLADHYPAQQAGLKSGDKIVQVGQYKTKSFDDIQS
AANKIKDNKTTIKFERDNQTKTVDITPKKQVIKQTKLNSETTYILGFQPEKEHTLIKPIA
LGFDQFVSASTLIFKAVGTMIASIFTGQFSFDMLNGPVGIYHNVDSVVKQGIIALTYYTA
LLSVNLGIMNLLPIPALDGGRILFVIYEAIFRRPVNKKAETIIIAAGAIFVLIIMVLVTW
NDIQRYFL
>ACAKHAOO_01225 Proline--tRNA ligase
MKQSKVFIPTRRDVPAEAEALSHQLLLKAGLIKQSTSGIYSYLPLASRVLNNISKIIREE
MESIDAVEILMPALQQAELWEESGRWGAYGPELMRLKDRNGREFALGPTHEEVVTSIVRD
ELKSYKQLPLTLFQIQSKFRDEKRPRFGLLRGREFIMKDAYSFHADEASLDETYQDMYNA
YDRIFKRVGINARPVVADSGAIGGNHTHEFMALSEIGEDTIVYSEHSDYAANIEKAEVVY
HPNEKHTEVAELEKVETPNVKTAQELADFLNRPVDEIVKSMIFKIDGEFIMFLIRGHHEL
NDVKVKAFFETDNVEMATQEEIVNLLGANPGSLGPVHDKDIRIFADNYVRDLNNLVVGAN
EDGSHYINANLDRDFKVDEFGDFRFILEGETLSDGSGEAKFAEGIEVGQVFKLGTKYSEA
MNATFLDNQGKAKPLIMGCYGIGVSRTLSAIVEQNNDENGIIWPKSVTPFDLHLITINPK
KDEQLELGDQLYKELQQQYDVLYDDRKDRAGVKFNDADLIGLPIRIVVGKNASEGIVEVK
VRQTGESEEVHINDLNTHIATLYSNL
>ACAKHAOO_01231 Translation initiation factor IF-2
MSKKRIYEYAKELNVKSKEIIDELKNMNVEVSNHMQALEDDQIKTLDKKFRQQESNNNTK
QNTQNNHQKQKNNNNNKNNNKQSNKGNANQKGNNNNKNNAKNNKNNKNNKNNKNNKNNKG
NKNNKPAAEPKEMPSKITYEEGITVGELADKLNIESSGIIKKLFLLGIVANINQALDEET
LELIADDYGVELEKEVVVNEEDLSIYFDEEEADPDAIERPAVVTIMGHVDHGKTTLLDSI
RHTKVTAGEAGGITQHIGAYQIENAGKKITFLDTPGHAAFTTMRARGAQVTDITILVVAA
DDGVMPQTIEAINHAKEANVPTIVAVNKIDKPTANPDRVMQELTEYGLIPEDWGGETIFV
PLSALSGEGIDDLLEMIGLVAEVQELKANPDKQAVGTVIEAELDKSRGPAASLLVQNGTL
NVGDSIVVGNTYGRIRAMVNDLGQRIKSAGPSTPVEITGINDVPLAGDRFVIFKDEKQAR
RIGEARHEASVIQQRQESKNVSLDNLFEQMKQGEMKDLNVIIKGDVQGSVEALAASLMKI
DVEGVNVRIIHTAVGAINESDVTLANASNGIIIGFNVRPDAGAKRAAEAENVDMRLHRVI
YNVIEEIESAMKGLLDPEFEEQVIGQAEVRQTFKVSKVGTIAGSYVTEGKITRNAGVRII
RDGIVLFEGELDTLKRFKDDAKEVAQGYECGITIEKFNDIKEGDIIEAFEMVEIER
>ACAKHAOO_01236 Polyribonucleotide nucleotidyltransferase
MSQEKKVFKTEWAGRSLTIEIGQLAKQANGAVLVRYGDTVVLSTAVASKEPRDGDFFPLT
VNYEEKMYAAGKIPGGFKKREGRPGDEATLTARLIDRPIRPLFPKGYRHDVQIMNTVLSA

DPDCSPEMAAMIGSSMALSVSDIPFQGPIAGVNVGYVDGKYVINPTVEEKEVSRLDLEVA
GHKDAVNMVEAGASEITEKEMLEAIFFGHDEIKRLVAFQEEVVAHIQPVKKEFVPVERDE
ALVSRVKTLTEEKGLKETVLTFDKQQRDENLDTLKAEIATEFVDEADPENELLIDEVYAI
LNDLVKEEVRRLIADEKIRPDGRKPDEIRPLESEVGLLPRAHGSGLFTRGQTQALSVLTL
GALGDYQLIDGLGPEQEKRFMHHYNFPNFSVGETGPVRAPGRREIGHGALGERALKYIIP
DTTEFPYTVRIVSEVLESNGSSSQASICGSTLALMDAGVPIKAPVAGIAMGLVTREDSYT
ILTDIQGMEDALGDMDFKVAGTKEGITAIQMDIKIDGLTREVIEEALEQARQGRLAIMDH
MLQTIDQPRKELSAYAPKVEIMHIKPEKIRDVIGPGGKKINEIIDETGVKLDIEQDGTIF
IGAIDQDMINRAREIIEDITREAEVGQVYNAKVKRIEKYGAFVELFAGKDALLHISQISK
ERINKVEDVLSIGDSIEVKITEIDKQGRVNASHKALDENK
>ACAKHAOO_01237 Ribonuclease J 2
MDGDFIIGGNILSLVKKKNDDIRIIPLGGVGEIAKNMYIVEVDDEMFMLDAGLMFPEDEM
LGVDIVIPDIQYVIENKEKLKGIFLSHGHEHAIGAVSYILEQIDAPVYGSKLTLALVKEN
MKSRNVKKKVRYYTVNHDSVMRFKNVNITFFNTTHSIPDSLGICIHTSYGSIVYTGEFKF
DQSLQGHYTPDIKRMAEIGEEGVFALISDSTEAEKPGYNTPENVIESHIYDAFAKVKGRL
IVSCYASNFIRIQQVLNTASKLNRKVSFLGRSLESSFNIARKMGYFDIPKDLLIPINEVG
NYPKNEVVIIATGMEGEPIEALSQMAQQKHRIMNIEEGDSVYLAITASANMEVIIADTLN
ELVRAGAYIIPNNKKIHASSHGCMEELKMMINIMKPEYFIPVQGEFKMQIAHAKLANESG
VAPEKIFLVEKGDVIHYDGKDMVLNEKVNSGNILIDGIGVGDVGNIVLRDRHLLAEDGIF
IAVVTLDPKNRRIAAGPEIQSRGFVYVRESEELMKEAEDKVREIVEAGLQEKRIEWSDIK
QNMRDQISKLLFESTKRRPMIIPVISEI
>ACAKHAOO_01238 DNA translocase FtsK
MPQTKKKTTTRKKGAIARKSTKKKQQKKDSSIRYIVAIIIVILSVLGMFQLGIVGRMIDS
FFNYLFGMSRYLTYILLSLITVYIAMQKKFPKTRRTFGLISLQFVLLITTQIIYHFQKGA
ASEREPVLSYVYKAYEHSHFPNFGGGLIGFYLLKIFIPLISIAGVIILTLILLSSSIILL
LKLRHRDVVKASIDKMRNSSQSASSNFKVKREQNRLKKEERQREKEEERERLRQHQLEQE
QLESEQIKDVSDFPEVSTEPEDIPIYGPSESQESKQTTTNKRKKRRFGLNSEDIDKENSS
NDGTNLEDENLPESNSNDNHQMNGTISEAGEESNVAYHIPPLSLLNQPAKQQSTSKSEVQ
RKGQILESTMKNFGVNAKVKQIKIGPAVTQYEIQPAQGVKVSKIVNLHNDIALALAAKDV
RIEAPIPGRSAVGIEVPNDKISLVTLKEVLESKFPASNKLEVGLGRDISGEPMTIQLNEM
PHLLVAGSTGSGKSVCINGIITSILLNAKPHEVKLMLIDPKMVELNVYNGVPHLLIPVVT
NPHKASQALEKVVAEMERRYDLFQHSSTRNIEGYNKFIRRQNEELDEKQAELPYIVVIVD
ELADLMMVAGKEVENAIQRITQMARAAGIHLIVATQRPSVDVITGIIKNNIPSRIAFAVS
SQTDSRTIIGSGGAEKLLGKGDMLYVGNGESAQTRVQGAFLSDQEVQDVVNYVVEQQKAN
YVKEMEPDAPVDKSEMKSEDALYEDAYIFVIEQQKASTSLLQRQFRIGYNRASRLMDDLE
RNQVIGPQKGSKPRQVLVNLDDEEV
>ACAKHAOO_01241 Putative zinc protease AlbF
MRETYYELIDERVFEHELTNGLRLFVIPKNGFQKTFVTYTTQFGSLDNTFKPHNQNDFVT
VPDGVAHFLEHKLFEKDDTEDLFTAFANDNAQVNAFTSFDRTSYLFSATDNVERNILRLL
EMVETPFFSKETVDKEKGIIAEEIKMYQEQPGYKIMFNTLRAMYQKHPIKVDIAGSVESI
YNITKDDLYLCYETFYHPSNMVLFVVGDVNPENIRDIVETHENKRDKTNQPSIERATVDE
PTNVITPFVSEEMKLQSPRLMLGFKNEPTEASPHEYVQHDLEMTLFFELIFGEETEFYQT
LLNEDLIDETFGYQFVLEPTYCFSIITSATQYPDKLKEVLLSELNKYCGNLDDQEAFELL
KKQFIGEFISSLNSPEYIANQYSKLYFEGVSVFDMLDIIDNITLDSINETATKFLNINNM
VDSRLEPKA
>ACAKHAOO_01247 Protein RecA
MDNDRQKALDTVIKNMEKSFGKGAVMKLGDNKGRRVSSVSSGSVTLDNALGVGGYPKGRI
IEIYGPESSGKTTVALHAIAEVQKNGGVAAFIDAEHALDPVYAEALGVDIDNLYLSQPDH
GEQGLEIAEAFVRSGAVDIVVVDSVAALTPKAEIEGEMGDTHVGLQARLMSQALRKLSGA
ISKSNTTAIFINQIREKVGVMFGNPETTPGGRALKFYSSVRLEVRRAEQLKQGQEIVGNR
TKIKVVKNKVAPPFRVAEVDIMYGQGISKEGEVIDLGVENDIVDKSGAWYSYNGDRMGQG
KENVKTYLKENPQVKEEIDRKLREKLGIFDGDVDEKEEDAPQTLFVEE
>ACAKHAOO_01249 Ribonuclease Y
MNLLNLLLILLGIILGVVVGYFVARNLLHQKQLQARQTADDIIKQGQKEADNIKKEKLLE

AKEENQIIREQTESELRERRGELQRQEARLLQKEENLDRKSDLLDKKDEILEQKESKLEE
RQQQVDAKESSVQTLIMKHEQELERISGLTQEEAVNEQLQRVEEELSQDIAVLVKEKEKE
AKEKVDKKAKELLATTVQRLAADHTTESTVSVVNLPNDEMKGRIIGREGRNIRTLETLTG
IDLIIDDTPEAVILSGFDPIRREIARTALVNLVSDGRIHPGRIEEMVDKARKEVDDIIRD
AGEQATFEINVHNMHPDLVKVLGRLNYRTSYGQNVLKHSIEVAHLSGMLAAELGEDVSLA
KRAGLLHDVGKAIDHEVEGSHVEIGVELAKKYAENETVINAIHSHHGDVEPTSIISILVA
AADALSAARPGARKETLENYIRRLERLETLSEGYDGVEKAFAIQAGREIRVIVSPDEIDD
LKSYRLARDIKTQIEDELQYPGHIKVTVVRETRAVEYAK
>ACAKHAOO_01263 Glycerol kinase
MEKYIMSIDQGTTSSRAILFDKEGDIKGVAQREFKQYFPKSGWVEHDANEIWTSVLAVMT
EVLNENEINADQIEGIGITNQRETTVIWDKNTGRPIYHAIVWQSRQTQSICHELKEQGHE
ETFRNKTGLLLDPYFAGTKVKWILDNVDGAREKAENGDLLFGTIDTWLVWKLSGGEAHIT
DYSNASRTLMYNIYDLQWDDELLDLLNVPKQLLPEVKESSEIYAHTKDYHFFGQEVPISG
IAGDQQAALFGQACFERGDVKNTYGTGGFMLMNTGEEPVKSESGLLTTIAYGLDGKVNYA
LEGSIFVSGSAIQWLRDGLRIINSAPQSENYATRVDSTDNVYFVPAFVGLGTPYWDSEAR
GAIFGLSRGTEKEHFIRATLESLCYQTRDVMEAMSKDSKIEVNNLRVDGGAVKNNFIMQF
QADIVNTAVERPEIQETTALGAAYLAGLAVGFWDSKDEIANRWQLETEFTPQMSEEDRTK
LYKGWKKAVEATQVFKLED
>ACAKHAOO_01264 Aerobic glycerol-3-phosphate dehydrogenase
MALSTLNREVIKKNLQNEEYDVVIIGGGITGAGIALDASQRGMKVALVEMQDFAQGTSSR
STKLVHGGLRYLKQAQIKVVAETGKERAIVYENGPHVTTPEWMLLPMHKGGTFGKFTTNL
GLTAYDRLAGVKKYERKKMLSKKQTLNKEPLVKKDGLKGGGYYVEYRTDDARLTIEVMKR
AEENGAEILNHTKSTDFIYDSKSKVRGIEVQDLLTGEMYEINAKKVINAAGPWVDEVRKK
DYTRNNKQLRLTKGVHVVIDQSKFPLRQAVYFDTEKDGRMIFAIPREGKAYVGTTDTFYD
NDKTKPLTTQEDRDYLIDAINYMFPDVNVKDEDIESTWAGVRPLILEDGKDPSEISRKDE
IWEGKSGLLTIAGGKLTGYRHMALEIVDLLAKRLKQEYKLTFAECKTKHTPISGGDVGGS
ANFESFVERKVEEGKAIGLQADVAKRLASKYGSNVDKLYNIAQIAQDKDLKLPLELYVEL
VYSVQNEMVFKPTDFLIRRSGKLYFNINEVKQYKDAVVEELAKLLNYTQSQQNEFTKEIN
IAIEEATRGNEQLAVLK
>ACAKHAOO_01272 Glutamine synthetase
MPKRTFSKDDIRKFAKEENVRYLRLQFTDILGTIKNVEVPVSQLEKVLDNEMMFDGSSIE
GFVRIEESDMYLCPDLDTWVIFPWTAGQGKVARLICDVYTTDGEPFAGDPRNNLKRVLKE
MEDMGYTDFNLGPEPEFFLFKLDEKGEPTLELNDNGGYFDLAPTDLGENCRRDIVLELED
MGFDIEASHHEVAPGQHEIDFKYADAITACDNIQTFKLVVKTIARKHNLHATFMPKPLFG
VNGSGMHFNVSLFKGKENAFFDPNGEMQLTEDAYHFTAGILNNARGFTAVCNPLVNSYKR
LVPGYEAPCYIAWSGKNRSPLVRVPSSRGLSTRIEVRSVDPAANPYMALATILQAGLDGI
KNKTKVPEPVNQNIYEMNREEREAVGIQDLPSTLYTALKAMRENDTVKKALGNHIYNQFI
NSKSIEWDYYRTQVSEWERDQYMKQY
>ACAKHAOO_01347 hypothetical protein
MTLTIILIVIIVILILAFILNQRYMQDRVDTEVYARNQMVTKNATLSNENLELKNQMLSS
NNDVSSHAKRNAKHVLTSILDKYKDEGKLKYYDIITTSNLATKHPFFEYARTFDYIVVSD
VGLINIDVKNWKQKTFYHFDAPVDDDMQIDTSDVNQIVGHYVSKQYHSQFDSPRSEIYTF
VEKIQNNRVIYEFYDHDPYEQAAINSKVLKDGIENHFNHKVQSIGVVYFSDGSVNIIEGS
EERAQYVDTVSTKSSLEAVIKNAIDLSKHPLSEDQVNKIVNSFNN
>ACAKHAOO_01350 Homoserine dehydrogenase
MKELNIALLGLGTVGSGVVKIIEENRQQIIDTLNKDIVIKHILVRDKSKKRPINISRYHL
TEDVNEILNDDSIDIVIEVMGGIEPTVDWLRDALKSGKHVITANKDLLAVHLKLLEDLAE
EKGLALKFEASVAGGIPIVNAINNGLNANNISKFMGILNGTSNFILSKMTREQTTFEDAL
DEAQRLGFAEADPTDDVEGIDAARKVVITSYLSFNQVIKLNDVKVRGISQTTLSDINVAD
KLGYKIKLIGKGTYQNSKVNASVEPTLIHKSHQLATVEDEYNAIYVVGDAVGDTMFYGKG
AGSLATGSAVVSDLLNVALFFESNTHTLPPHFELKTDATREIMDTEDVVAIQEKLNYYIV
LNSDLSKEKFKSLLKETLPFHKSLQVEQRDEQTYGVVIIGLDQSPEDVLSKADFNIEKIY
PVEGV
>ACAKHAOO_01356 Catalase

MAKDDKRLTGLFGHPVSDRENSMTAGPRGPLLMQDAYFLEQMSHFDREVIPERRMHAKGS
GAFGTFTVTNDITKYTNAKIFSEVGKQTEMFARFSTVAGERGAADLERDIRGFALKFYTE
DGNWDLVGNNTPVFFFRDPKLFVSLNRAVKRDPRTNMRSPQNNWDFWTGVPEALHQVTIL
MSDRGMPKDFRHMHGFGSHTYSMYNDEGERVWVKYHFRTQQGIENYTDDEAAEIVGQDRE
SSQRDLYDAIENGDYPKWKMYIQVMTEEQAKNHPDNPFDLTKVWYKGDYPLIEVGEFELN
RNPENYFMDVEQAAFAPTNIIPGLDFSPDKMLQGRLFSYGDAQRYRLGVNHWQIPVNQPK
GVGIENMCPFSRDGQMRFLDGNQGGGPHYYPNNKGVYQSQPEYKKPAFPVDGDGYEYNQR
QDDDNYFEQPGKLFRLQSDDAKERIFTNTANAMDGVSEDVKHRHIRHCYKADPEYGKGVA
KALEIDINDVDLEGTNDETYENF
>ACAKHAOO_01359 GMP reductase
MKIFDYEDIQLIPNKCIVESRSECDTTVQFGPKTFKLPVVPANMQTVMSEELAHWFAKND
YFYIMHRFDEAARIPFIQKMQKDGLFASISVGVKANEFKFIEELADKQLVPEYITIDIAH
GHSDSVIKMIKHIKKYIPKTFVIAGNVGTPEGVRELENAGADATKVGIGPGRVCITKIKT
GFGTGGWQLAALNICSKAARKPIIADGGLRTHGDIAKSIRFGASMVMIGSLFAAHEESPG
ETVELDGKRYKEYFGSASEFQKGEHKNVEGKKMFVEHKGSLKDTLVEMQQDLQSSISYAG
GKDLKSLTTVDYVIVRNSIFNGDKD
>ACAKHAOO_01365 Transketolase
MFNEKDQLAIDTIRALSIDAIEEANSGHPGLPMGAAPMAYTLWTRHLNFNPQSKDYFNRD
RFVLSAGHGSALLYSLLHVSGSLELEELKQFRQWGSKTPGHPEFRHTDGVEVTTGPLGQG
FAMSVGMALAEDHLAGKFNKDDFNIVDHYTYVLASDGDLMEGISHEAASFAGHNQLDKLI
VLYDSNDISLDGELNKAFSEDVKKRFESYGWNHILVKDGNDLDAIDKAITKAKSQNGPTM
IEVKTIIGYGAPNVSGTNGVHGAPLGSDERKLTFEAYGLDPEKRFNVPEEVYEIFQTTML
KRANEHEDAWKALLENYSKQYPELADEFKLAISGKLPKNYRDELPRFDSDHNAATRADSG
EVIQALSKSVPSFFGGSADLAGSNKSNVKEATDYDRNTPEGKNIWFGVREFAMGAAVNGM
AAHGGLHPYGATFFVFSDYLKPALRLSAIMGLNSTFIFTHDSIAVGEDGPTHEPIEQLAG
LRSIPNMNVIRPADGNETRVAWEVALESEQTPTSLVLTRQNLPYLDVDEETVEQGVRKGA
YVVFETETKPEYLLLATGSEVSLAIEAAKDLDKQGKGVRVVSMPNWFAFEQQPEEYKESV
IPKEITKRVAIEMASPLGWHKYVGTEGKVIGIDQFGASAPGDLVVEKYGFTKENVLNQIR
TF
>ACAKHAOO_01371 Glycine betaine transporter OpuD
MNSSSEETKQNKKFSSVFIYSAIVVAIVVSIGAFLPEQFGKITGDISSWITEKLGWYYMI
LTTVIVFFCIFLIFSPIGKLKLGKPNDKPEFNTVSWFAMLFSAGMGIGLVFYGAAEPMAD
FASPPNADPKTTEAYTEALRSTFFHWGFHAWAVYGVVALALAYAQFRKNEPGLISRTLRP
ILGDKVEGPIGTIIDVLSVFATVVGVAVSLGMGALQINGGLHYLFGVPNNVWVQSIIIVV
VTILFLASAWSGLSKGIQYLSNLNIGLGTVLMIIALIIGPTVLILNMFTSSTGSLLNSFL
FNSFDTAALNGQKREWMSSWTLYYWGWWLSWSPFVGVFIARVSKGRSIREFISGVLLVPA
IVSFLWFSVFGVLGIETGKKHPELFKMTPETQLFGVFHHVPLGMALSLIALVLIASFFIT
SADSATFVLGMQTTFGSLEPSSAVKVTWGVSQSLIAFILLLAGGGNGADALNAIQSAAII
SAFPFSFVVIMMMISFYKDANQERKFLGLTLQPNKHRLQDYVKYQQQDYESDILEKRESR
RNAEK
>ACAKHAOO_01372 Aconitate hydratase A
MTSNIKQQAKKTFEANGQSYTYYDLKSLEEQGLTKISKLPYSIRVLLESVLRQEDEFVIT
DEHIKALGNFGNEGNEGEVPFKPSRVILQDFTGVPAVVDLASLRKAMNDVGGDINKINPE
VPVDLVIDHSVQVDSYANPDALERNMKLEFERNYERYQFLNWATKAFDNYNAVPPATGIV
HQVNLEYLANVVHVRDVDGEQTAFPDTLVGTDSHTTMINGIGVLGWGVGGIEAEAGMLGQ
PSYFPIPEVIGVRLTNSLPQGSTATDLALRVTQELRKKGVVGKFVEFFGPGVTDLPLADR
ATIANMAPEYGATCGFFPVDEESLKYMRLTGRKEEHVELVKAYLEQNNMFFTVDKEDPEY
TDVIDLDLSTVEASLSGPKRPQDLIFLSDMKKEFEKSVTAPAGNQGHGLDKSEFDKKANI
NFADGSTATMKTGDIAIAAITSCTNTSNPYVMLGAGLVAKKAVEKGLKVPEFVKTSLAPG
SKVVTGYLRDSGLQEYLDDLGFNLVGYGCTTCIGNSGPLLPEIEKAVAEEDLLVTSVLSG
NRNFEGRIHPLVKANYLASPQLVVAYALAGTVDIDLQNEPIGKGKDGQDVYLNDIWPTIQ
EVADTVDSVVTPELFLEEYKNVYNNNEMWNEIDVTDAPLYDFDPNSTYIQNPSFFQGLSK
EPGTIEPLKDLRVMGKFGDSVTTDHISPAGAIGKDTPAGKYLLDHDVPIRNFNSYGSRRG
NHEVMVRGTFANIRIKNQLAPGTEGGFTTYWPTDEVMPIYDAAMKYKEDGTGLAVLAGND

YGMGSSRDWAAKGTNLLGVKTVIAQSYERIHRSNLVMMGVLPLQFKDGESAESLGLDGKE
AISVDIDETVSPRDTVKVHAKKENGEVVDFEAIVRFDSLVELDYYRHGGILQMVLRNKLA
Q
>ACAKHAOO_01377 DNA topoisomerase 4 subunit A
MSEIIQDLSLEDVIGDRFGRYSKYIIQERALPDVRDGLKPVQRRILFAMHSSGNTYDKNF
RKSAKTVGDVIGQYHPHGDLSVYDAMVRLSQDWKLRHVLIEMHGNNGSIDNDPPAAMRYT
EAKLSQLSEELVRDINKETVAFVPNYDDTTLEPMVLPARFPNLLVNGSTGISSGYATDIP
PHNLAEVIQATLKYIDNPDITVTQLMKYIKGPDFPTGGIIQGVDGIKKAYETGKGKIVVR
SKVDEETLRNGRQQLIVTEIPYEVNKSSLVKRIDELRADKKVDGIIEVRDETDRTGLRIA
IELKKDVNAEAIKNYLFKNSDLQIAYNFNMVAISDGRPKLMGIRQIIDSYLNHQIEVVAN
RTKYDLDHAEKRMHIVEGLMKALSILDEVITLIRNSKNKKDAKDNLVAEFEFTEAQAEAI
VMLQLYRLTNTDIVALENEHNELANLIKEYRHILDNHDALLQVIKSELTDIRKRFKSERL
SSIEAEIAEIKIDKEVMVPSEDVVLSITRQGYIKRTSTRSFNASGVTEVGLKDGDSLLKY
QTVNTQDTVLVFTNKGRYLFIPVHKLADIRWKELGQHVSQIVPLDDSETVVDVYNEQSFK
DDAFYVLATRNGMIKKSNVSLFKTTRYNKPLVAMKVKDNDELINVIRLNEDQLISVLTHK
GMSLTYSSEELSDTGLRAAGVKSINLKDEDFVVMTDVVDSDSSIIMATQRGAVKRISYKI
LQQAKRAQRGITLLKELKKNPHRVVAGYVVKDESMYTLYSESHSEEGQITDIHLSEQYTN
GSFVVDVKEFGDVLDMTID
>ACAKHAOO_01396 Aminoacyltransferase FemA
MKFTNLTATEFGNYTDKMPYSHFTQMTENYEMKVANKTETHLVGIKNKDNEVIAACMLTA
VPVMKFFKYFYSNRGPVIDYDNRELVHFFFNELTKYLKQHNCLYVRVDPYLPYQYLNHDG
EITGNAGNDWFFDKMKHLGFEHEGFTKGFDPIKQIRYHSVLDLKNKTSKDILNGMDSLRK
RNTKKVQKNGVKVKFLSEEELPIFRSFMEDTTETKEFQDRDDSFYYNRYRHFKDRVLVPL
AYIKFDEYIEELQNERETLNKDVNKALKDIEKRPDNKKAFNKKENLEKQLDANQQKLDEA
KKLQAEHGNELPISAGFFFINPFEVVYYAGGTSNKYRHFAGSYAIQWTMINYAIDHGIDR
YNFYGISGNFSEDAEDVGVIKFKKGFNADVIEYVGDFVKPINKPLYSVYKTLKKIKKRFN
>ACAKHAOO_01397 Aminoacyltransferase FemB
MKFTELTVKEFENFVQNPSLESHYFQMKENIQTRENDGFEVVLLGVKDESNKVLAASLFS
KIPTMGSYVYYSNRGPVMDYSDLGLVDFYLRELEKYLHQHQCLYVKIDPYWIYQIYDKDI
NPLEDREKNDAIVNLFKSHGYEHHGFTTEYDTSSQARWMGVSYLKGETPASLRKQFDSQR
KRNINKAINYGVKVRFLGRDEFHIFLDLYRETEARTGFVSKTDEYFYNFFDTYGDKVLVP
LAYIDLDEYIKSLQDALNDKENRRDQMMAKENKNDKQLRKIAELDKQIEHDKKELLQASE
LRQTDGQILNLASGIFFANSYEVNYFSGGSSEKYNNYMGPYMMHWHMMNYCFEHGYDRYN
FYGLSGDFTENSEDYGVYRFKRGFNVYIEELIGDFYKPINKVKYWLFNTLNTIRKKVKK
>ACAKHAOO_01399 putative ABC transporter solute-binding protein YclQ
MKNSFKFIAILFTIIVLLSACGKNSTSESSSSSKDTITVENTYEFKDKNNTHTKGEMKTE
KVKVPVNPDRVAVLDYGALDIMKQMGLEDKIVAIAKGKGAAFLPSSLSEFKKDKYTNLGN
PGQPNYDNLANAKPEVIFTSFRQAHTKTLDEIKKAAPNAKILFVSPNNDHYIDSIKDNTT
LFGKIFKQQNKAKMLNEKLNKKVAETKKVINNDTVLFLNVDDKGVKAFGPTGRFGGFLNR
DLGIKHADTAMKANSAGNSITYEYLNKVNPDKLFVIDRTQKGNDKSLPSALNNDVIKNLK
AIKNDQVYQFESNAWYFSEGGIETTIEQLDKIEKAFKK
>ACAKHAOO_01401 Oligoendopeptidase F, plasmid
MTSGLPFRKDVPELEKWNLNDLFNSDEQFYDTLDNVLNQSKSFNQQYKGKISDAKMIKKV
LSEFENILIQLDRLGNYAELRLSVDTANTDAQTLSAKLSTNYGKISSQLSFVESEILMLP
EEVIKQAIEESDVPHYLEKLIESKPYQLSPEVEQVMASLSPTFQSAYELYGTTKMLDIAF
ESFEHNNVTYPMDYATFENEYEDNANPEFRQKSFKSFSDALKKYQHTTAATYNQHVQQEK
IEADLRGYDSVIDYLLHDQEVTREMYDRQIDVIMSDLAPVMQKYAKLLQRIHGLDKMRFE
DLKVSVDPSYEPEISIEDSKQYIHGALNVLGEDYIQMVEQAYSDRWIDFAQNKGKDTGAY
CASPYYTHSYVFISWTGKMTETFVLAHELGHAGHFTLAQQHQNLLDSEASMYFVEAPSTM
NEMLMANYLFSNSEDPKFKRWVIGSILSRTYYHNMVTHLLEAAYQREVYRKVDNGESLTA
PVLNNIMRDVYEQFFGDAVELTDGTELTWMRQPHYYMGLYSYTYSAGLTIGTVVSQKIKQ
EGQPAVDAWLETLKAGGSQSPIELAKIAGVDITTDAPLKATIKYISDLVNEAEQLTNEIE
QQD
>ACAKHAOO_01403 Phosphate import ATP-binding protein PstB 3

MANTNVKEKELAKHTDQSQESISTVVSSNEVKHNKESDSNKKVVYSTQNLDLWYGDTHAL
QNINLDILENNVTAIIGPSGCGKSTYIKTLNRMVELVPSVKTAGKILYRDQNIFDENYSK
EKLRTNVGMVFQQPNPFPKSIYDNITYGPKTHGIKDKKLLDEIVEKSLRGAAIWDELKDR
LHTNAYGLSGGQQQRVCIARCLAIEPDVILMDEPTSALDPISTLRVEELVQELKENYSII
MVTHNMQQAARVSDKTAFFLNGYVNEYDDTDKIFSNPSDKQTEDYISGRFG
>ACAKHAOO_01404 Phosphate transport system permease protein PstA
MTATTNPKAKTLIDQNKVEKNISSRGKTNKLNKWLFFTCIVIALLVLAALLIQTFVKGVG
YLTPDFFTNFSSSTPSQAGIKGALAGTIWLMISIIPISIVLGVGTAIYLEEYAKDNFFTS
FVKISISNLAGVPSIVFGLLGLTLFVRGGGIQALALGNSVIAAALTMSLLILPIIIVSSQ
EAIRAVPNSVREASYGLGANKWQTIRRVVLPAALPGILTGFILSLSRALGETAPLILIGI
PTILLATPTSIMSMFTALPTQIYTWAKMPQAEFQNVASAAIIILLVILLLMNAVAIFLRN
KFSRKY
>ACAKHAOO_01406 Phosphate-binding protein PstS
MKKWQLVGTTVLGASVLLGACGGGDSSGSGSGDGKDLKGEAKGEGSSTVAPIVEKLNEKW
AKDHPNATISSGQAGTGAGFEKFIAGETDFSQASRPIKDEEKQKLEDKDIKYKEFKIAQD
GVTVAVNKDNDFVKELSKDQLKKIYNGEAKTWKDVNSSWPDKKIKAFSPNSSHGTYDFFE
EEVMDKEDIKAEKNGDTNVIVQSVEKNKEGIGYFGYNFYEQNKDKLKEVKIKDDKGKTTE
PTKKTIKDGSYALSRPLFLYVKEKSLKDNDVMREFIKFTLEDKGKSAEDAGYVASPDKTY
KDELKDLKKYDKKSDK
>ACAKHAOO_01407 Conserved virulence factor B
MALDKDIVGSIEFLEVIGLEGSTYLLKGPNGEQVKLNQSEINDEDELETGEEYSFFIYPN
RSGELFATQNMPDITKDKYDFAKVLKTDRDGARVDVGLPREVLIPWEDLPKVKSLWPQTG
DYVLVTLRIDRENQMFARLASETIVEKMFTPVYDDEKQNELIKARPYRLLRIGSFLLSKE
GYKIFVHESERKEEPRLGEEVEVRIIGHNDKGELNGSFLPLAHERLDDDGQVIFDLLVEY
EGELPFWDKSSPEAIKEVFNMSKGSFKRAIGHLYKHKIINIETGKISLTKKGWSRVEDK
>ACAKHAOO_01408 putative ABC transporter ATP-binding protein YbiT
MLQVTDVSLRFGDRKLFEDVNIKFTEGNCYGLIGANGAGKSTFLKILSGEIDSQTGHVSM
GKDERLAVLKQDHFAYEEERVLDVVIKGHERLYEVMKEKDEIYMKPDFSDEDGIRAAELE
GEFAEMNGWNAEADAASLLSGLGIAADLHDKQMSELENNQKVKVLLAQSLFGEPDVLLLD
EPTNGLDIPAISWLEDFLINFDNTVIVVSHDRHFLNNVCTHIADLDFGKIKLYVGNYDFW
YQSSQLAQKMAQEQNKKKEEKIKELQDFIARFSANASKSKQATSRKKQLEKIELDDIQPS
SRRYPYVKFTPEREIGNDLLTVQGLSKTIDGEKVLDNISFTMNPNDKAILIGDSEIAKTT
LLKILAGEMEPDEGTFKWGVTTSLSYFPKDNSEFFEGVDMNLVDWLRQYAPEDEQTETFL
RGFLGRMLFSGEEVKKKASVLSGGEKVRCMLSKMMLSSANVLLLDEPTNHLDLESITAVN
DGLASFKGSIIFTSYDFEFINTIANRVIDLNQSGGLSKEVPYEEYLQEIGVLQK
>ACAKHAOO_01411 4-hydroxy-tetrahydrodipicolinate synthase
MPHLFEGVGVALATPFTNNEVDYNALEKHVDFLLDNGVKAIIVNGTTAESPTLTEEEKEQ
VLESVIKQVNHRVTIIAGTGTNNTAKSIQASQRAKALGADAIMLITPYYNKTNQRGLVKH
FETIANEVELPVVLYNVPSRTNMTIEPETVETLSHNQYIVAIKDATNDFEYYEDVKSRIN
QEEFALYSGNDDNVVEFYQRGGNGVISVIANAIPKEFQALYDAKQSGQDISNDFEPISKL
LDALSVDVNPIPIKALTSHLGFGNYELRLPLLPLEDADAKVLINVFEQFKAGEL
>ACAKHAOO_01413 2,3,4,5-tetrahydropyridine-2,6-dicarboxylate N-
acetyltransferase
MVQHLSAEEIIQYISDAKKSTPLKVYVNGNFDGVTFPDSFKVFGSDNSKVIFCEADDWKP
FYESNQSTITELEIEMDRRNSAIPLKDLTNTNARIEPGAFIREQAIIEDGAVVMMGATIN
IGAVVGEGTMIDMNATLGGRATTGKNVHVGAGAVLAGVIEPPSASPVVIDDNVLIGANAV
ILEGVHVGEGAIVAAGAIVTQDVPAGAVVAGTPAKVIKQTSEVEDSKREIVSALRKLND
>ACAKHAOO_01416 Diaminopimelate decarboxylase
MAVKYNDYGELTMAGTSLKTLAQSFGTPTIVYDEDEIRNQMRRYHSAFQKSGLKYNISYA
SKAFTCIQMVKLVQEEDLQLDVVSEGELYTALEAKFDPSKIHFHGNNKTKREIQYALESG
VGYFVIDSLEEIDLIDRYASDTVNVVLRVNPGVEAHTHEFIQTGQEDSKFGLSIRHGLAI
EAVNKVRDSKRLHLKGIHFHIGSQIEGTEAMIETAKIVLNWLSENDIKVELLNLGGGFGI
KYVEGDVSFPIEEGIAEITDAIKAETERLNYEVPEIGIEPGRSIVGEAGITLYEVGTIKE
IPEVNKYVSIDGGMSDHIRTALYGAKYQALLVNRHEEADETVTIAGKLCESGDIIIRDAK

LPSSVQRGDYLAILSTGAYHYSMASNYNQMQKPPVFFLKDGKAREVIKRQSLRQLIINDT
K
>ACAKHAOO_01418 Cold shock protein CspA
MKQGTVKWFNAEKGFGFIEVEGENDVFVHFSAINQDGYKSLEEGQSVEFEVVEGDRGPQA
ANVVKL
>ACAKHAOO_01422 TelA-like protein
MPRNDQFNNSHPLDDYIEQQQLNNLDNNSNVSLANQNTVLDDFQQQFSDKEMQKIESISQ
QIKPLDNDGLLSYGSHLQENMSKFSHKMLDEVQTKDIGPVGDSLNQLMTKLKAVNPDELN
PEKQSKLKRFFKRTKASINEVFSRMQSVSSQIDRITIQLDRHKNNLSKDIKLLDGLYQQN
KDYFDDVNLYIAAAKRRKHEIQTNDIPKLQEHANQTGNQMDIQAVADMEQFVDRLDKRIY
DLQLSRQIAIQTAPQIRMIQNVNQALAEKIQSSILTSIPLWKNQMAIALTLMRQRNAVSA
QRAVTDTTNDLLTQNAAMLKQNAIETATENERGIVDIETLKTTQSDIIETIEQTLQIQQN
GRQKRKEAEKELVGLEDELKQHLLSMKKE
>ACAKHAOO_01435 UDP-N-acetylglucosamine--N-acetylmuramyl-(pentapeptide) pyrophosphoryl-undecaprenol N-acetylglucosamine transferase
MSKIAFTGGGTVGHVSVNLSLIPTATDKGHDAFYIGSKTGIEREMIESQLPNIEYYPISS
GKLRRYLSVDNAKDVFKVLKGVIDARKVLKREKPDLLFSKGGFVSVPVVIAARSLNIPTI
IHESDLTPGLANKISLKFAKKIYTTFEDTLKYLPKDKADFVGATIRQDLKEGNQSRGYQL
TGFDASKKVLLVMGGSLGSKKLNQAIRENLEALLQDYQIIHLTGHGLVDSSIDAKGYVQY
EFVKEELTDLLAITDTVVSRAGSNAIYEFLTLRIPMLLIPLGLDQSRGDQIDNAENFESK
GYGRTIPEDQLTQVKLLEQLKEIENDRESIIKQMETYRESYTKEDLFNKILKDAL
>ACAKHAOO_01439 PTS system glucose-specific EIIA component
MFKKLFGKGKEVNKEIEIYAPLTGEYINIENIPDPVFAQKMMGEGFGINPTEGEVVSPIE
GKVDNVFPTKHAIGLKADNGLEILVHIGLDTVQLDGQGFEILVSSGDTVQVGDPLLKFDL
EYIRNNAKDVISPIIITNSDQTESIHVNDVKAVIKGETKVVDVTMS
>ACAKHAOO_01453 Extracellular matrix-binding protein ebh
MNYRDKLQKFSIRKYAVGTFSTLIATLVFLGTHTDQAHASENTNAAVEKAQQNTESQTSR
DKNSNPNDAITIQNDQTSDQTVEINKQSKFVDKTSTEKKALETQTSEKEITNSTSTSIKE
KIEESSPVTKEEKTNELKEKSSPIVGTQSSNQSKQKVKFSSSKSVKQIDDKIDTPRESKS
DNRQKIDSKNISKLTNLTQEIQSKLLEVETIEPSNPNIEEAKKLTVESSSFTQSSNVSQQ
SLLDLVTRLEQTRNSLANVITRSQSGKRDPRNGQQIEKGTNFRFTTLNGRWNAGRNVIVY
QRNYASLPDGRALGTGQQKNGVEGITSRKTVMRAYYKHEGNSKYLVYDVFFNNDGVNFIS
PGSQQRLGMALLLPYKVMKLNSDGSFASDSVRNLSYAAYEKRSGRNSLLSESPSDFIIDP
DNSTQTIDMLRSNQHDFGHTTFYLSFGVRPGRSFNSDANEYFHSNRNNPDLRKAVEDQRG
IFSGWNYGIGIQVDPNHPEGANRAYHMHLEVKLRDNVTTAELENAWSYANTAAMGGVSKS
AYTVLSGRILPEDGALPSIENQPPVKPTINSDLVGKATTTTVIDVSTDPNTKVEIFDKNG
NKIGTGTTDSSGHAYITPTRPIPEGNVTAKAYNHSDESKVSTSDPKFATDTIPPTTPVIN
TRLVYKAGTLTPIDVSTDPNTQVALIDKNGRIFGAGTTDSSGHVIITPDRVIPEGNVTAK
ATDNALHPNSSVSIPVQATTLIPVMKPVINTDVAGKAFSSPVIDITSTPNTRVELLDKNN
NVIGRGITGSNGHVNITPDHYLFEGNITAKAYDQTDETNNATSDPRHVTDTTPPRKPVIN
TNLVNKVGTRTPIDVSTDVLTRVEIFDENGKSYGVVLTEMDGHGIITPREPLPLGKIYAR
ATDGAETPNSIDSDHVPVTDTIPPTVPTVDTDLTGKATTLTPITVTTDPNTRVDLIDKNG
HIIGTGTTDGNGHVIITPTTPIVEGNVIAKAYDPANNVSTSAPRKATDTTPPTKPRVTSP
LGGKAGTTDPVTVTTDPNTNVQLLDKNGQIIGTGTTDSSGRVNITPTRPIPEGNVTAKAI
DNAEHPNSSTSDPVKATDTTPPTKPRVTTPLGGKAGTTDPVTVTTDPNTNVQLLDKNGQI
IGTGTTDSSGRVNITPTRPIPEGNVTAKAIDNAEHPNSSTSDPVKATDTTPPTKPRVTTP
LGGKATTLTPVEVTTDPNTSVQLLDKDGRVIGSGTTGANGRVTITPTRPIPEGNVTAKAT
DNAEHPNSSTSDPVKATDTTPPTKPRVTTPLGGKATTLTPIEVTTDPNTSVQLLDKDGRV
IGSGTTGANGRVTITPTRPIPEGNVTAKATDNAERPNSSTSDPVKATDTTPPTEPVVTND
LTGKATTKTPITVTTDPNTHVDLLDKDNHVIGSGTTDSNGRVTITPTVPIPEGNVRAKAT
DNAEHPNSSLSQPKKATDTTPPGSPIVNTDLTGKATTKTPVDVSSDPNTRIELLDKDNHV
IGSGTTGANGHVIITPTQPIPEGNVTAKAYDNAEHPNVSTSAPKKATDTTPPTEPVVTND
LTGKATTKTPITVTTDPNTHVDLLDKDNHVIGSGTTDSNGRVTITPTVPIPEGNVRAKAT

DNAEHPNSSLSQPKKATDTTPPGSPIVNTDLTGKATTRTPVDVSSDPNTRIELLDKDNHV
IGSGTTDSNGRVTITPTVPIPEGNVRAKATDNAEHPNSSLSQPKKATDTTPPGSPIVNTD
LTGKATTKTPVDVSSDPNTRIELLDKDNHVIGTGTTGANGHVIITPTQPIPEGNVTAKAY
DNAEHPNSSTSQPKKATDTTPPTEPVVTNDLTGKATTKTPITVTTDPNTHVDLLDKDNNV
IGSGTTDSNGRVTITPTVPIPEGNVRAKATDNAEHPNSSLSQPKKATDTTPPGSPIVNTD
LTGKATTKTPVDVSSDPNTRIELLDKDNHVIGSGTTGANGHVIITPTQPIPEGNVTAKAY
DNAEHPNVSTSEPKKATDTTPPTAPVVTSDLTGKATTTDPVEVTTDPNTKVELLDKDGNV
IGSGTTDNTGHVMITPTKPIPEGNVTAKAYDNAEHPNSSTSQPKKATDTTPPTAPVVTSD
LTGKATTTDPVEVTTDPNTKVELLDKDGNVIGSGTTDNTGHVTITPTKPIPEGNVTAKAT
DNAEHPNSSTSQPKKATDTTPPTAPLVTSDLTGEATTTDPVEVTTDPNTKVELLDKDGNV
IGSGTTDNTGHVTITPTKPIPEGNVTAKAYDNAEHPNSSTSQPKKATDTTPPTAPVVTSD
LTGKATTTDPVEVTTDPNTKVELLDKDGNVIGSGTTDNTGHVTITPTKPIPEGNVTAKAT
DNAEHPNSSTSQPKKATDTTPPTAPLVTSDLTGKATTTDPVEVTTDPNTKVELLDKDGNV
IGSGTTDNTGHVMITPTKPIPEGNVTAKAYDNAEHPNSSTSQPKKATDTTPPTAPVVTSD
LTGKATTTDPVEVTTDPNTKVELLDKNGNVIGSGTTDNTGHVMITPTKPIPEGNVTAKAY
DNAEHPNSSTSQPKKATDTTPPTAPVVTSDLTGKATTTDPVEVTTDPNTKVELLDKDGNV
IGSGTTDNTGHVTITPTKPIPEGNVSAKAYDNAEVPNVATSQPKKATDTTPPTTPTLDTD
LDGKAGTQTPITVTTDPNTHVDLLDKDGNIIGSGTTDDTGHVTITPTKPIPEGNVTAKAT
DNAEHPNSSTSQPKKATDTTPPTAPVVTSDLTGKATTTDPVEVTTDPNTKVELLDKDGNV
IGSGTTDDTGHVTITPTRPIPEGNVSAKAYDNAEVPNVAASQPKKATDTTPPTTPTLDTD
LGGKAGTQTPITVTTDPNTHVDLLDKDGNIIGSGTTDDTGHVTITPTKPIPEGNVTAKAT
DNAEHPNSSTSQTKKATDLTPPVKPSVVGTLDGKAGTKDPVEVVTDPNTKVELLDKDGNV
IGSGTTDSTGHATITSTVPIPEGNVTVKATDNAEHPNSSTSDPVKATDTTPPTVPTLDTD
LGGKAGTQTPITVTTDPNTKVELLDKDSNIIGSSTTDDTGHVTITPTKPIPEGNVTAKAT
DNAEHPNSSTSQPKKATDLTPPVKPSVVGTLDGKAGTKDPVEVVTDPNTKVELLDKDGNV
IGSGTTDSTGHATITPTVPIPEGNVTVKATDNAEHPNSSTSDPVKATDTTPPTVPTLDTD
LGGKAGTQTPITVTTDPNTHVDLLDKDGNIIGSGTTDDTGHVTITPTKPIPEGNVTAKAT
DNAEHPNSSTSQPKKATDTTPPTAPVVTSDLTGKATTTDPVEVTTDPNTKVELLDKDGNV
IGSGTTDSTGHATITPTVPIPEGNVTVKATDNAEHPNSSTSDPVKATDTTPPTVPTLDTD
LGGKAGTQTPITVTTDPNTHVDLLDKDGNIIGSGTTDSTGHVTITPTKPIPEGNVTAKAT
DNAEHPNSSTSQPKKATDTTPPGACG
>ACAKHAOO_01462 Penicillin-binding protein 1A/1B
MTENKGSSQPKNGKNGKSKAKKNKNVKRTIIKIIGFLLIAFIVLVLLGILLFAYYAWKAP
AFTESKLQDPKPAEIYDRNGDLVKKLDNGARREHVELKDVPKNMRNAVLATEDNRFYDHG
AIDYKRLFGAIGKNLTGGFGSQGASTLTQQVVKRAFLSEEKSIGRKAQEAYLSYRLEQEY
SKDEIFQMYLNKIYYSDGVYGVKAAAKYYFNKDLKDLNLAEEAYLAGLPQVPNLYNIYDN
PKQAENRKNTVLYLMHYHNRISDKEYNAAKKINLKANLVDRTAKERQIGTDNSDSEYDSY
VNFVKSELMNNKEFKNKNLSDVLQSGIKIYTNMDKDVQKTLQDRIDNGNYYKNEDQRVGS
TILDSKTGGLVAISGGRNFKDVVDNNAATDAHLTGSSLKPFLAYGPAIENMQWATNHAIQ
DESSYSIDGGTFRNYDGNGHGTVTAYDALRQSFNIPALKTWQQVNSQAGDNAVKKFASKV
GLDYQSDIGPSDVLGGGASEFSPTQLASAFAAIANGGTYNNAHSIQKVVTADDDTIEFDH
TSHKAMKDYTSYMLAEMLTGTFKAYGSAYGHGVSGVTMGAKTGTSTYGTEINQQYNLPDN
AAKDVWINGFTPKYTMSIWMGFSEIKEGGVNSFLGHSEQEYPQYLYEDVMSDITSSDDGD
FKKPDSVNGSSADNLSVSGHPDNNTTNREVNGSSGSSSSNSSNSSNNSSNSNTSNTNNSG
NTNSGTTNNNSTTQGTNTVGSTLSNIFNLNSIFDFKKSS
>ACAKHAOO_01466 Asparagine--tRNA ligase
MKTTIKQAKQHLNQEVTIGAWLTNKRSSGKIAFLQLRDGSGFMQGVVVKSEVSDETFQLA
KEITQESSLYVTGVITEDNRSDLGYEMQVKSIEVIHEAHDYPITPKNHGTEFLMDHRHLW
LRSKKQHAVMKIRNEIIRATYEFFNENGFTKIDPPILTASAPEGTSELFHTKYFDEDAFL
SQSGQLYMEAAAMAHGRVFSFGPTFRAEKSKTRRHLIEFWMIEPEMAFTNHVESLEVQEQ
YVSHVVKSVLKNCQLELKALDRDTSKLEKVSAPFPRISYDDAIKFLKEEGFNDIEWGEDF
GAPHETAIANHYDLPVFITNYPTKIKPFYMQPNPENEETVLCADLIAPEGYGEIIGGSER
INDLELLEQRIDEHQLDAESYNYYLDLRRYGSVPHSGFGLGLERTVAWISGVEHVRETAP
FPRLLNRLYP

>ACAKHAOO_01476 3-phosphoshikimate 1-carboxyvinyltransferase
MSNEQMIDIKGPLIGEIEVPGDKSMTHRAIMLASLATGQSTIYKPLLGEDCLRTIEIFKL
LGVNIELAEEKIIVDSPGYNKFKTPHQTLYTGNSGTTTRLLAGLLSGLNLNCVLSGDASI
GKRPMDRVMKPLRLMGANITGIDDNFTPLIIKPASINGITYKMEVASAQVKSALLFASLF
SNDSSKITELDVSRNHTETMFEQFNIPISISGKEITTQPNAIEHIKAKDFYVPGDISSAA
FFIVAALITPGSDITIHNVGINPTRSGIIDIVKQMEGNIECLNIIDTSEPTASIRVKYTP
NLKPVLIEGDIVPKAIDELPIIALLCTQASGTSIIKDAEELKVKETNRIDTTADMLGLLG
FELQPTDDGLIIHPSEFKKSATVDSLTDHRIGMMLAIASLLSDKPLNIRQFDAVNVSFPG
FLPKLMLLENEG
>ACAKHAOO_01479 Nucleoside diphosphate kinase
MERTFLMIKPDAIQRNLVGEIISRIERKGLKLVGAKLMTVPQALAEEHYSEHTDKPFYGK
LISFITSAPVFAMVVEGEDAVDVSRHIIGKTNPSEATPGSIRGDLGLTVGRNVIHGSDSV
ESAQREINLWFKEEELSSYEAPRDTWLYE
>ACAKHAOO_01485 DNA-binding protein HU
MNKTDLINAVAEQADLTKKEAGSAVDAVFESIQQSLSKGEKVQLIGFGNFEVRERAARKG
RNPQTGKEIEIPASKVPAFKAGKALKDAVK
>ACAKHAOO_01486 Glycerol-3-phosphate dehydrogenase [NAD(P)+]
MTKITVFGMGSFGTALANVLAENGHTVLMWGKNEDSVKELNDHHQNKRYLKDVVLDSRIK
ATSDIKEAANFTDIYLMALPTKAMREVTSEIDSLIDSKKTFIHVAKGIENDTFKRVSEMI
EDSISEDHNGGIGVLSGPSHAEEVVIKQPTTVAASSKDEKVSKLIQDLFMNDYLRVYTNN
DLVGVELGGALKNIIAVASGIVAGMGYGDNAKAALMTRGLAEISRLGEKLGADPMTFLGL
GGIGDLIVTCTSTHSRNYTLGFKLGQGQTMDEALNEMNMVVEGIYTTNSVYHLAKQQNVD
MPITNALYKVLFEDNPVKDSVKDLMGRDKKSE
>ACAKHAOO_01487 GTPase Der
MTKPIVAIVGRPNVGKSTIFNRVVGERVSIVEDTPGVTRDRIYSSGEWLTHEFNIIDTGG
IEIGDAPFQTQIRAQAEIAIDEADVIIFMVNVREGLTQSDEMVAQMLYKSKKPVVLAVNK
VDNPEMRNDIFDFYSLGFGDPYPISGSHGLGLGDLLDEVVKHFDEETEDSYDEDTIRLSI
IGRPNVGKSSLVNAILGEDRVIVSNVAGTTRDAVDTEYSYDGQDYVLIDTAGMRKKGKVY
ESTEKYSVLRALKAIERSNVVLIVIDAEEGIIEQDKRVAGYAHEEGKAVVIVVNKWDTVD
KDSNTMKKFADDVRNQFQFLDYAQIAFVSAKEGTRLRTLFPYINEASENHKKRVQSSTLN
EVVTDAISMNPTPTDKGRRLNVFYTTQVANEPPTFVVFVNDVELMHFSYKRYLENQIRHA
FGFEGTPIHIIPRRRN
>ACAKHAOO_01488 30S ribosomal protein S1
MTEEFNESMINDIKEGDKVTGEVQQVEDKQVVVHINGGKFNGIIPISQLSTHHIENPSEV
VKQGDEIEAYVTKIEVDEENDSGVYILSKRQLETEKSYEYLQEKLDNDEIIEAKVTEVVK
GGLVVDVGQRGFVPASLISTDFIEDFSVFDGQTIRIKVEELDPENNRVILSRKAVEQAEN
DVKKASLLESLNAGDVIKGKVARLTNFGAFVDIGGVDGLVHVSELSHEHVDSPEDVVSVG
QEVDVKVKSVEKDAERISLSIKDTLPTPFESIKGQFHEDDVIEGKVVRLANFGAFVEIAP
GVQGLVHISEIAHEHIGTPGEKLEPGQQVNVKILGIDEENERISLSIKATLPKEDVVESD
DATTQSYLSNDSIEDNPTLGDVFGDKFKDLKF
>ACAKHAOO_01492 Elastin-binding protein EbpS
MSKNNFRDDFEKNRQSINSDDQFEDNTNEFDENSNESNDFDNQSDQQFPPRNAQRRQRRR
NQATNKNRKFGNQNSDSNANGSLDDRHDEDSFNELQHNQQENHLDNEPIHKDDKLSSEKD
FNNDASRRNNRHEASNRKHDKDYDNGSLNDDDRHRRNHEEGIDERQDNRNHKDQQNKKSR
HGKDAAIAGGAGVAGAAGAKAAKDKRKKDEHHDSKYNEHKDDRDLNNDNQFDQNRKHDKD
LHDDHRDAKDNHSKEEPKKGNKGKKAAVGAGAAGAAGAAGVAAAKHKKDHKNNNHKDNHS
HNRDHQDDHRNHKHEDGNDGFQAHNGKKKRGLAGILLPLIALLLILAALAIFIGMYLNND
KKDSNQADNKTEQTANKDNNKDSKDKASNDSDKDKASSDKDKDKATNDDDSNDKATTDND
SSNNSSDDNSSSSDNSTSSNSSDNSSSSDNNGNNSNSDNNNGNSQATSNNSSQSNSNNNQ
SNSSNSGQQTHVVSGNENLYRIAIQYYGEGTVENVNKLKQANGLSSNNISNGQKLIIPQ
>ACAKHAOO_01499 Ribosomal large subunit pseudouridine synthase B
MSKELERLQKRIANSGYTSRRKAETLITEGKVKVNGQTVTELGTKVKSSDTVEVEGIKIE
QEDKMYILFYKPTQVITSVSDDRGRTVVTDYFKDLETRIYPVGRLDYDTSGLLLLTNDGE
FTNLMTHPRYHIQKKYVAKLKGYLMREEVKALENGIELEDGFTQPAQVKVKKQDKDKNIT

LVEITISEGRNRQVRRMFEHFGHQVDKLTRIEFGNLNLKGLNAGEGRVLTPHEVKVLRHM
AENGK
>ACAKHAOO_01504 Ferric uptake regulation protein
MEERLNRVKQQLQQSSYKLTPQREATVRVLIENEKDHLSAEDVYLKVKDKAPEIGLATVY
RTLELLAEIKVVDKINFGDGVARFDLRKEGAKHFHHHLVCMECGKVDEIHEDLLPQVEER
VETEYNFKILDHRLTFHGVCAECQAKGK
>ACAKHAOO_01518 6-phosphogluconate dehydrogenase, decarboxylating
MTQQIGVVGLAVMGKNLAWNIESRGYSVSVYNRSSEKTDEMVNESKGKQIHPTYSLEEFV
NSLEKPRKILLMVKAGPATDATIDSLLPLLDDDDILIDGGNTNYQDTIRRNKALAESGIN
FIGMGVSGGEVGALTGPSLMPGGQEAAYNKVSDILDAIAAKAKDGASCVEYIGPNGAGHY
VKMVHNGIEYADMQLIAESYAMMKDLLGMSHEDISKTFKEWNAGELESYLIEITGDIFTK
LDDDNEALVEKILDTAGQKGTGKWTSINALELGIPLTIITESVFARFISSIKQERVNASK
ELNGPKAEFTGNKEEFLEKIRKALYMSKICSYAQGFAQMRKASEENEWNLKLGDLAMIWR
EGCIIRAQFLQKIKDAYDNNSELQNLLLDPYFKGIVTEYQDALRDVVATGVRNGVPTPGF
SASVNYYDSYRSENLPANLIQAQRDYFGAHTYERKDKEGIFHTQWVEE
>ACAKHAOO_01522 hypothetical protein
MDLNFDLYMNGVVEQARNEIENAGYEQLTTADEVDKVLQQNGTSLVMVNSVCGCAGGIAR
PAASHALHYDKLPDRLVTVFAGQDKEATQQARDYFEGYAPSSPSFALIKDGKITEMIERH
QIEGHDVMDVINQLQGLFDKYCDER
>ACAKHAOO_01529 Farnesyl diphosphate synthase
MAKKSMNELINQINSALDGVIETSPLNTNLEESMQYSLNAGGKRIRPLLVFLTLDVLNQD
YKKGKKTALALEMIHTYSLIHDDLPAMDNDDYRRGKLTNHKVYGEWKAILAGDALLTKAF
EIIADDELLESDVKVKILSRLAHNSGHLGMVGGQTLDMQSEDKPVDLDTLEQIHKAKTGA
LLQFAVLSAADIAKVDKSTSQALEDFSNHMGLMFQIKDDLLDVYGDESKLGKKVGSDIEN
HKSTYVSLLGKDGAEDKLNFHKDAAIKSIEQLSSHYDIKPLLDIVELFYTRDH
>ACAKHAOO_01534 2-oxoglutarate carboxylase small subunit
MKKILIANRGEIAVRIIRACHDLGIQTVAIYSEGDKDALHTQIADEAYCVGPTQSKDSYL
NIPNILSIATSTGCDGIHPGYGFLAENGDFAELCEACQLKFIGPSYESIQKMGIKDIAKA
EMIAANVPVVPGSDGLVTTISDAKQIAKEIGYPVIIKATAGGGGKGIRVARTEKELENGY
RMTQQEAETAFGNGGLYLEKFIENFRHIEIQIIGDQHGNVIHLGERDCTIQRRMQKLVEE
SPSPILSDEQREEMGNAAIRAAKAVNYENAGTIEFIYDLNDNQFYFMEMNTRIQVEHPVT
EMVTGIDLVKLQLKVAMGEALPYTQEDITINGHAIEYRINAENPYKNFMPSPGKITQYLA
PGGYGVRIESACYTNYTIPPYYDSMVAKLIVHEPTRDEAIMTGLRALSEYLILGIDTTIP
FHLKLMNNDIFRSGSFNTNFLEQYNIMDDEE
>ACAKHAOO_01538 hypothetical protein
MKKLIVIIGASCLLVGCGSQNLGPLEDKTTKLRDQNHNLKLDIQQLNQDISNQKAQVEAL
NKDKKNVSKTVDNNKEAKFLDASSKYYQDITKVISNYNQLDLSKNKKEDKKQNLEKLNTI
ANGIDDAYGKYKGAVTKKDLSSANKNEDKNIRQINKELQSAFKDIKSGYENNNTNKLNKG
KTKLSQVNMSNNS
>ACAKHAOO_01541 putative protein YibN
MNVSLIIALAIIVIIILYMLGNWFMNKRAVTELEQNEFHKGLRKAQVIDVREKVDYDYGH
INGARNIPISMFSQRYQGLRKDQPIYLCDANGVAGYRAARILKKNGYKDIYMLKGGYKKW
TGKIKTKK
>ACAKHAOO_01542 putative glycine dehydrogenase (decarboxylating)
subunit 2
MVVSKSSPLIFERSREGRYAYSLPQSDIKTDSVESILDDKFIRKNKAEFPEVAELDLVRH
YTELSNKNFGVDSGFYPLGSCTMKYNPKINEKVARIPGFAESHPLQEEGQVQGSLEIVYS
LQEELKEITGMDEVTLQPAAGAHGEWTALMIFKAYHLDNGEGHRDEVIVPDSAHGTNPAS
ASFAGFKAVTVKSNERGEVDIEDLKRVVNENTAAIMLTNPNTLGIFEKNIMEIREIVHEA
GGLLYYDGANLNAIMDKVRPGDMGFDAVHLNLHKTFTGPHGGGGPGSGPVGVKKELASYL
PKPMVIKDGDTFKYDNDIKNSIGRVKPFYGNFGIYLRAYTYIRTMGAEGLREVSEAAVLN
ANYIKASLKDHYEIPYEQYCKHEFVLSGSKQKEHGVRTLDMAKRLLDFGVHPPTIYFPLN
VEEGMMIEPTETESKETLDYFIDAMIQIAEEAKNDPDKVLEAPHSTIIDRLDETTAARKP
VLKFDNLHEEKE

>ACAKHAOO_01543 putative glycine dehydrogenase (decarboxylating) subunit 1
MSHRYIPLTEKDKKEMLDKIGASSINELFGDVPKDILLNRDLNIASGEAETSLLRRLNTV
ANKNVTKETHASFLGAGVYDHYAPAVVDAMISRSEFYTAYTPYQPEISQGELQAIFEFQT
LICELTDMDVANSSMYDGITSFAEACILAFNHTKKNKIVVSKGLHYQALQVLKTYVKVRE
EFEIVEVDLDGTITDLEKLEQAVDDETAAVAVQYPNFYGSVEDLEKIQSFIEGKKALFIV
YANPLALGLLTPPGSFGADIVVGDTQPFGIPTQFGGPHCGYFATTKKLMRKVPGRLVGQT
QDDEGNRGFVLTLQAREQHIRRDKATSNICSNQALNALASSIAMSALGKQGIYDIAVQNL
EHANYAKNQFKDNGFEVLDGTSFNEFVVKFDQPIKDINKKLAEHGFIGGFDLGEASADFE
NHMLIAVTELRTKDEIDTFVKKAGELNGSK
>ACAKHAOO_01544 Aminomethyltransferase
MTSELKKTPLYQNYVDSGAKIVEFGGWAMPVQFTSIKEEHNAVRYEVGMFDVSHMGEISI
KGNDASKFVQYLLSNDTNNLTDTKAQYTALCNEEGGIIDDLVTYKIGDNDYLLIVNAANT
DKDFAWVQKHAPKFDVEVSNVSNQFGQLAVQGPKARDLVSGLVDIDVSEMKPFDFQQNVT
LFGKNVILSQSGYTGEDGFEIYCEAKDTVDIWNGFIEHNVVPCGLGARDTLRLEAGLPLH
GQDLTESITPYEGGIAFAAKPLIEEDFIGKSVLKDQKENGSERRTVGLELLDKGIARTGY
PVLDLDGNEIGEVTSGTQAPSSGKSIAMAIIKRDEFEMGRELLVQVRKRQLKARIVKKNQ
IEK
>ACAKHAOO_01555 Glucokinase
MKDIILAADIGGTTCKLGIFNGELEQLHKWSIKTDTSDHTGKTLLKNIYDSFNETLSTHQ
LKIDDVIGVGIGVPGPVDFETGIVNGAVNLHWPGSVNVRQIFSEFINCPVYVDNDANVAA
LGEKHKGAGQGADDVVAITLGTGLGGGIISNGELVHGHNGSGAEIGHIRTDFDQRFNCNC
GKSGCIETVASATGVVNLVNFYYPKLTFKSSILPLIKDNKVTAKAVFDAAKAGDQFCIFI
TEKVANHIAYLCSIISATSNPKYIVLGGGMSTAGLILIENIKTEYHNLTFTPAQKDTEIV
QAQLGNDAGITGAAGLIKTYILEKGGVK
>ACAKHAOO_01557 Rhomboid protease GluP
MDTEKYFWKSIYYFIKYHNYHIVNIDKNDSEIWLIHKKKNKLVIFRKDIASNQEIQFDKA
KMLDNYQQYEDDLNFKLKSVKYYYFTDQIFDNIKSADSSPIKIESMCISNNNDLHKLIHN
NILTKLMFRQDNKLSNYYKRRVLSQNPIDKHMLRFTPMTYGLIIINVLIWLIMILYLNHF
SDVKLLDLGGLVHFNVVHGEWYRLITSMFLHFNFEHILMNMLSLFIFGKIVESIVGPLRM
LGIYVISGLLGNFISLSFNLHTVSVGASGAIFGLIGSIFAMMFVSKTYSKKTIGQMLIAL
LVLIVLSLFMSNINIMAHLGGFIGGVLITLIGYYFTHNRNLFWIFLIILLVLFVALQVRI
FTIKEENIYDKLIEDAILDYNYKEASSIVNHTIDKGYDDDRTYYLKGLITAATSSRAEGM
ADWERGLKNHPNSGLLNYELAIANRALDDNEKALKYVKKALTINSDDNDYKNLKKELDQS
NESRN
>ACAKHAOO_01561 Superoxide dismutase [Mn/Fe]
MAFELPNLPYATDALEPHIDKQTMEIHHDKHHNTYVTKLNSAVEGTDLESKSIEEIVANL
DSVPEDIQTAVRNNGGGHLNHSLFWELLTPNSEEKGTVVDKIKEQWGSLDEFKKEFADKA
AARFGSGWAWLVVNNGQLEIVTTPNQDNPLTEGKTPILGLDVWEHAYYLKYQNKRPDYIS
AFWNVVNWEKVDELYNATK
>ACAKHAOO_01574 Glycine--tRNA ligase
MVKDMETIVSLAKHRGFVFPGSDIYGGLSNTWDYGPLGVELKNNVKKAWWQKFITQSPFN
VGIDAAILMNPKTWEASGHLGNFNDPMIDNKDSKIRYRADKLIEDYMQKEKGDENFIADG
LSFDEMKRIIDDEGIVCPVSGTANWTDIRQFNLMFKTFQGVTEDSTNEIFLRPETAQGIF
VNYKNVQRSMRKKLPFGIGQIGKSFRNEITPGNFIFRTREFEQMELEFFCKPGEEIEWQN
YWKTFASQWLKDLNINEENMRLRDHDEEELSHYSNATTDIEYRFPFGWGELWGIASRTDF
DLKKHSEHSGEDFKYHDPETNEKYVPYCIEPSLGADRVTLAFLCDAYEEEGVEGSKDART
VMHFHPALAPYKAAVLPLSKKLSEEAIKIFEQLSSKFAIDFDESQSIGKRYRRQDEIGTP
YCITFDFDSLEDNQVTVRDRDSMEQVRMPISELETFLAEKVAF
>ACAKHAOO_01581 hypothetical protein
MNIGIIIFIISIIVTGISAMRDKSHEHRKNQRPPHPKKSTSDKKETHEKGFFEQIEEAFS
ELEKEFTNEDKTSNQQKKDKQTNTKRVYQDQKLEKEIEKESVPEELERSARQRGRVERET
RQSANSRESNRDSNKLQKELEKQLVDDLYNVRTEIDREKEKQLSRIENNARAIINDKNLS
ERTKRYRLKQLLNSKAIEQDMTYQSFQFDNDPVVNGILWQEILNKPKQL

```
>ACAKHAOO_01582 hypothetical protein
MFGLGIIVIAVIIVIALLVLFSFVPVGLWISAIAAGVKVGIGTLVGMRLRRVSPRKVIGP
LIKAHKAGLNLTTNQLESHYLAGGNVDRVVDANIAAQRADINLPFERGAAIDLAGRDVLE
AVQMSVNPKVIETPFITGVAMNGIEVKAKARITVRANISRLVGGSGEETIIARVGEGIVS
TIGSSEHHTQVLENPDNISKTVLSKGLDSGTAFEILSIDIADVDIGKNIGADLQTEQALA
DKNIAQAKAEERRAMAVASEQEMKARVQEMRAKVVEAESEVPLAMAEALREGNLGVKDYY
NLKNVEADTGMRNAINKRTEQNEDESPK
>ACAKHAOO_01584 30S ribosomal protein S21
MSKTVVRKNESLEDALRRFKRSVSKSGTIQEVRKREFYEKPSVKRKKKSEAARKRKFK
>ACAKHAOO_01588 Chaperone protein DnaJ
MAKRDYYEVLGVSKDASKDEIKKAYRKLSKKYHPDINKEEGADEKFKEISEAYEVLSDDN
KKANYDQFGHDGPQGGFGGQGFSGQDFSGFGGGGFEDIFSSFFGGSRQRDPNAPRKGDDL
QYTMTLEFEEAVFGTKKEISIRKDVTCHTCNGDGAKPGTSKKTCSYCNGAGHVSVEQNTI
LGRVRTQQTCPKCDGTGQEFEEPCPTCHGKGIENKTVKLEVTVPEGVDNDQQIRLAGEGI
PGENGGPHGDLYVVFRVKPSDKFERDGDDLYYNLDVSFPQASLGDEIKVPTLNGNVMLTI
PSGTQTGKQFRLKDKGVKNVHGYGHGDLFVNIKVVTPTKLTDRQKEIMREFAELNGESIE
EQPSNFKDRARKFFKGE
>ACAKHAOO_01589 Chaperone protein DnaK
MSKVIGIDLGTTNSCVAVLEGDEPKVIQNPEGARTTPSVVAFKNGETQVGEVAKRQAITN
PNTVQSIKRHMGTDYKVDIEGKSYTPQEISAMVLQNLKNTAESYLGDKVDKAVITVPAYF
NDAERQATKDAGKIAGLEVERIINEPTAAALAYGLDKTDQDQKVLVFDLGGGTFDVSILE
LGDGVFEVLSTAGDNKLGGDDFDQVIIDYLVSEFKKENGVDLSQDKMALQRLKDAAEKAK
KDLSGVSQTQISLPFISAGESGPLHLEISLTRSKFEELADSLIRRTMEPTRQALKDAGLS
TSEIDEVILVGGSTRIPAVQEAVKKEIGKDPHKGVNPDEVVAMGAAIQGGVITGDVKDVV
LLDVTPLSLGIEIMGGRMNTLIERNTTIPTSKSQVYSTAADNQPAVDIHVLQGERPMASD
NKTLGRFQLTDIPPAPRGVPQIEVTFDIDKNGIVNVTAKDLGTNKEQNITIQSSSALSDE
EIDRMVKDAEENAEADKKRREEVDLRNEADSLVFQVEKTITDLGDNISEEDKSNAESKKD
ALKSALEGQDIEDIKAKKEELEKVIQDLSAKVYQQAQQAQQQAQDGAQQTQNDSNVEDAE
FKEVNDDEDKK
>ACAKHAOO_01590 Protein GrpE
MTEKDESVKSNSEYTEEQEVKNEDTSTVENVEDTTSDSDNSSNDSSNEESSEETAVDPKD
EEIQQLQLKANENEEKYLRLYAEFENYKRRIQKENETNKTYQSQRVLTDILPTIDNIERA
LQIEGDDESFKSLQKGVQMVHESLLRALKDNGLEEIESEGQAFDPNFHQAVVQDDNPDFK
SGDITQELQKGYKLKDRVLRPSMVKVNQ
>ACAKHAOO_01594 30S ribosomal protein S20
MPNIKSAIKRVRTTENAEARNISQKNAMRTAVKNAKTAINNNADNKAELVNFAIKSVDKA
SQSNLIHSNKADRIKSQLMSSSK
>ACAKHAOO_01615 Transcription elongation factor GreA
MENQKQYPMTQEGYEQLEKELEELKTVKRPEVVEKIKVARSFGDLSENSEYDAAKDEQGF
IEQDIQRIEHMLRYALIIEDTGDNNVVQIGKTVTFIELPGDEEESYQIVGSAEADAFNGK
ISNESPMAKALIGKSLKDEVRVPLPNGGEINVKIVNIK
>ACAKHAOO_01616 Uridine kinase
MANTTIIGIAGGSGSGKTTVTNEIMKNLEGHSVALLAQDYYYKDQSHLTFEERLETNYDH
PFAFDNDLLIENLKDLRNGHAVEVPTYDYTNHTRSNETIAFEPKDVIIVEGIFALENKTL
RDMMDVKIYVDTDADLRILRRLVRDTKERGRSMESVINQYLNVVKPMHNQFIEPTKKYAD
IIIPEGGSNKVAIDIMTTKIQTLVSKQ
>ACAKHAOO_01623 Alanine--tRNA ligase
MKNLKASEIRQKYIDFFVEKGHMVEPSAPLVPIDDDSLLWINSGVATLKKYFDGRETPRK
PRIVNSQKAIRTNDIENVGFTARHHTFFEMLGNFSIGDYFKKEAIHFAWEFLTSENWMGM
EPEKLYVTIHPEDSEAYRIWNDEIGLEESRIIRIEGNFWDIGEGPSGPNTEIFYDRGDEY
GQDDPSEEMYPGGENERYLEVWNLVFSEFNHNKDNTYTPLPNKNIDTGMGLERMASVSQN
VRTNYETDLFMPIINEVEKIADKKYLVNNEHDIAFKVIADHIRTIAFAIADGALPANEGR
GYVLRRLLRRAVRFSQTLDINEPFMYKLVDIVAEIMEPYYPNVKEKADFIKRVIKSEEER
FHETLQEGLAILNNLLAKAKSSNNEINGSDAFKLYDTYGFPIELTEELASQENISVDMAT
```

FEKEMDQQRTRAREARQSSQSMQIQSEVLKNITTESKFVGYETTDYQTTLTDLIYNGEKV
ESAEPGETVYFILKETPFYAVSGGQVADKGTVGNENFEIEVTEVTKAPNGQNLHQGVIQF
GQVTINSDVDASVNRDERKDIQKNHSATHLLHAALKEVLGEHVNQAGSLVESERLRFDFS
HFGPMTQDEIDQVERRVNEEIWRGISVNIQEMPINEAKQMGAMALFGEKYGDIVRVVNMA
PFSIELCGGIHVDNTAEIGLFKIISESGTGAGVRRIEAFTGKSAFLHLEDIQNKFNSIKD
QVKVKSDNQVFDKIVQLQEEEKNLHKQLEQRNKEITSLKMGNIEDQIEEINGFKVLATEV
EVSNAKEIRQTMDDFKSKQQDAIIILASDLGEKVSLIATVPKEQTDKIKAGDIIKNMAPL
VGGKGGGRPDMAQGGGTQPENISEALRFIKDYIKKL
>ACAKHAOO_01626 tRNA-specific 2-thiouridylase MnmA
MSNKDIRVVVGMSGGVDSSVTAHVLKDQGYDVIGIFMKNWDDTDENGVCTATEDYNDVIA
VCNQIGIPYYAVNFEQEYWDKVFTYFLDEYKKGRTPNPDVMCNKEIKFKAFLEHALKLGA
DYVATGHYARIRRHDDGHVEMLRGVDNNKDQTYFLNQLSQQQLSKVMFPIGDIDKKEVRK
IAEEQDLATAKKKDSTGICFIGERNFKTFLSQYLPAQSGEMRTLNGDKMGMHSGLMYYTI
GQRHGLGIGGDGDPWFVVGKNLEDNILYVEQGFHHDALYSDYLIASDFSFVNPVDLDNGF
ECTAKFRYRQKDTKVFVQRESQNALRVTFDEPVRAITPGQAVVFYDEEVCLGGATIDDVF
KTTGQLSYVV
>ACAKHAOO_01628 Alkanal monooxygenase beta chain
MGNLKVSALNLVPIREGQSDKEAIEDMISLAQKLDSLNYERYWIAEHHNAPNLVSSATSL
LIQHTLEHTKRIKVGSGGIMLPNHAPLIVAEQFGTMETLFPNRVNLGLGRAPGTDMMTAS
ALRRDQHNGVYEFPEEVEQLQTYFGPGNKQAYVRAYPAVGKNVPLYILGSSTDSAHLAAR
KGLPYVFAGHFAPQQMKEAIQIYKELFEPSDVLDKPYMIVCLNVIVAESDEEAEYLASTL
AQVFIGIARGRMQPVQPPTNDLQALLTPREFEMAKQRFNDSLIGSEETVKEKLEAFIEEY
GEIDELMGISYIYDQEKQFESFTRLQNIIEKLNQ
>ACAKHAOO_01629 hypothetical protein
MGFMDKAKDAADKFKNSDNEQVDKAKDKINEYTEKNNDDKKDDK
>ACAKHAOO_01630 HTH-type transcriptional regulator CymR
MKISTKGRYGLTLMISLAKREGQGCVSLKSIAEENSLSDLYLEQLVGPLRNAGLIRSVRG
AKGGYQLRVPADEIKAGDIIRLLEGPITIVESIESEPPAQKQLWLRMRDAVREVLDNTTL
KYLADYVETDDKLDGYMFYI
>ACAKHAOO_01633 Aspartate--tRNA ligase
MSKRTTYCGLVTEELLNQKVTLKGWVHNRRDLGGLIFVDLRDREGIVQIVFNPDFSEEAL
SVAETVRSEYVVEVEGTVTKRDPDTVNSKIKTGQVEVQVSNISIINKSETPPFSINEENQ
NVDENIRLKYRYLDLRRPELAQTFKMRHQTTRAIREYLDNNGFFDIETPVLTKSTPEGAR
DYLVPSRVHEGEFYALPQSPQLFKQLLMISGFDKYYQIVKCFRDEDLRADRQPEFTQVDI
EMSFVDQEDVIQMGEEMLRKVVKDVKGIDVSGPFPRMTYEEAMRRYGSDKPDTRFEMELK
DVSQLGREMDFKVFKDTVENNGEVKAIVAKGAADNYTRKDMDALTEFVNIYGAKGLAWVK
VVDDGLSGPIARFFEDSNVATLKDLTQAESGDLVMFVADKPNVVAQSLGALRIKIAKELG
LIDENKLNFLWVTDWPLLEYDEDAKRYVAAHHPFTSPKQEDISKLDSEPQNAQANAYDIV
LNGYELGGGSIRIADGELQEKMFEVLGFTKEQAREQFGFLLDAFKYGAPPHGGIALGLDR
LVMLLTNRTNLRDTIAFPKTASATCLLTDAPGEVSDKQLEELSLRIRH
>ACAKHAOO_01640 Protein translocase subunit SecDF
MKKFSRIIAFILIVAVLFVGMGLTYKNVVKNVNLGLDLQGGFEVLYQVKPLDGDKKIDEK
ALQSTARTLENRVNVLGVSEPHIQVEDPDRIRVQLAGVKDPDEARKILSSQANLTIRDAN
DKVKLTGKDIVQGSAKQEFKQGTNEPAVTFKLKDRAKFKKVTEEISKKQENMMVVWLDYK
KGDSYHKELEKPEDKRKYVSAASVDQPINSDSVEISGGFHGQQGVERAKQIAELLNAGSL
PVDLKEIYSNSVGAQFGQDALDKTIFASAIGVAIIYLFMVGFYRLPGLVAIIALTVYIYL
TLVAFNFISGVLTLPGLAALVLGVGMAVDANIIMYERIKDELRIGRTLKQAYSKANKSSF
LTIFDSNLTTVIAAGVLFFFGESSVKGFATMLLLGILMIFVTAVFLSRGLLSLLVSSNYF
KKKFWLFGVSKKNRHDINEGVDVHDLKTSYEKWNFVKLAKPLIGFSILILIVGIVILSIF
KLNLGIDFSAGTRVDIDSDTKLSQPKVERTMKDMGLTPDQVQINGSDNKQATVQFKKDLS
KNEVVELNKKVNNEYGHKPTVNTVSPMIGQELAKNAMKALIYAAIGIIIYVSLRFEWRMG
LSSVLALLHDVFMIVALFSLLRLEVDITFIAAVLTIVGYSINDTIVTFDRVRENLHKIKV
ITKPEQIDYIVNRSIRQTMTRSVNTVLTVIVVVVAILIFGASSLFNFSLALLIGLVSGVF
SSVFIAVPLWGIMKKRQLKKADNGKLTVYKEKKSNDEKILV

>ACAKHAOO_01647 GTPase Obg
MFVDQVTISLKAGDGGNGITAYRREKYVPFGGPAGGDGGKGASVVFEVDEGLRTLLDFRY
QRHFKAKKGENGQSSNMHGRGADDLVLKVPPGTIIKSVETDEVLADLVEDGQRAVVARGG
RGGRGNSRFATPRNPAPDFSENGEPGEELDVTLELKLLADVGLVGFPSVGKSTLLSIVSK
AKPKIGAYHFTTIKPNLGVVSTPDNRSFVMADLPGLIEGASDGVGLGHQFLRHVERTKVI
VHMIDMSGSEGRDPFDDYQIINKELVNYKQRLEDRPQIVVANKMDMPDAQDNLTLFKEQV
DDSVTIIPVSTITRDNIEQLLYAIADKLDEVKDIDFSVDNDEEIGVNRVLYKHTPSQDKF
TITRDDDGAYVVSGNAIERMFKMTDFNSDPAVRRFARQMRSMGIDDALRERGCSNGDIVR
ILGGEFEFVE
>ACAKHAOO_01648 50S ribosomal protein L27
MLKLNLQFFASKKGVSSTKNGRDSESKRLGAKRADGQYVTGGSILYRQRGTKIYPGENVG
RGGDDTLFAKIDGVVRFERKGRDKKQVSVYAVAE
>ACAKHAOO_01650 50S ribosomal protein L21
MFAIIETGGKQIKVEEGQEIFVEKLDVNEGDSFTFDKVLFVGGDSVKVGAPTVEGATVTA
TVNKQGRGKKITVFTYKRRKDSKRKKGHRQPYTKLTIDKIKA
>ACAKHAOO_01660 Valine--tRNA ligase
MNMEPKYNPREVEAGRYEEWVKNDYFKPSEDKSKETYTIVIPPPNVTGKLHLGHAWDTTL
QDIITRMKRMQGYDTLYLPGMDHAGIATQAKVDAKLKEQGISRHDIGREKFLEHAWSWKE
EYASFIRQQWAKLGLGLDYSRERFTLDDGLSKAVRKVFVDLYNKGIIYRGERIINWDPEA
RTALSDIEVIHEDVQGHFYHFKYPYADGDGYIEIATTRPETMLGDTAIVVNPNDDRYKDV
IGKKVILPIVGRELPILADEYVDIDFGSGAMKVTPAHDPNDFEIGQRHSLENIIVMDENG
KMNDKADKYAGLDRFECRKQLVEDLKAQDLVIKIEEHVHSVGHSERSGAVVEPYLSTQWF
VKMKPLAQRSLDNQKTDDRIDFYPPRFENTFNRWMEEIRDWTISRQLWWGHQIPAWYHKE
TGEIYVGEEAPKDIDNWVQDEDVLDTWFSSALWPFSTLGWPNIDADDFKRYYPTNALVTG
YDIIFFWVARMIFQGLEFTDRRPFNDVLLHGLVRSEDGRKMSKSLGNGVDPMDVIEEYGA
DSLRYFLATGSSPGHDLRYSTEKVESVWNFINKIWNGARFSLMNIGDEFKFEDIDLTGNL
SLADKWILTRLNETIETVTNLSEKYEFGEVGRALYNFIWDEFCDWYIEMSKIPMNGEDEA
QKQTTRSVLSYTLDQIMRMLHPFMPFVTEKIWQSLPHEGETIVKASWPTVREELVFEESK
QTMQQLVEIIKSVRQSRVEVNTPLSKAIPIYIQAKDENIKATLIENEDYIHKFCNPSDLT
IDTHIDIPEKAMTAVVIAGKVVLPLEGLIDMDKEIARLEKELDKLQKELDRVDKKLSNEN
FVNKAPEKVINEEKEKQQRYQEKYDGVKNRIEQLKA
>ACAKHAOO_01663 Glutamate-1-semialdehyde 2,1-aminomutase 1
MGYEKSIEAMKIAENLMPGGVNSPVRAFKSVDTPAIFMDHAKGSRIYDIDGNEYIDYVLS
WGPLILGHKNEQVIKKLHEAVNRGTSFGASTLEENKLAELVIERVPSIEKVRMVSSGTEA
TLAALRLARGYTGRNKIIKFEGCYHGHSDSLLIKAGSGVATLGLPDSPGVPEGTAKNTIT
VPYNDLEAIKIAFENYGDDIAGIIVEPVAGNMGVVPPKDGFLQGLREITNDYGALLIFDE
VMTGFRVGYNCAQGYFGVTPDLTCLGKVIGGGLPVGAFGGRKEIMDKVAPVGNIYQAGTL
SGNPLAMTSGYETLSQLTPESYEYFQELGDILEEGLKKVFSKHNVPITINRAGSMIGYFL
NEGPVTNFEEANKSNLELFSQMYREMAKEGVFLPPSQFEGTFLSTAHSKEDIEKTIQAFD
TALSRIV
>ACAKHAOO_01664 Delta-aminolevulinic acid dehydratase
MKFDRHRRLRSSESMRNLVRENHVRKEDLIYPIFVVEKDNVKSEIKSLPGVYQISLNLLD
DEINEAYELGIRAIMFFGVPNAKDDVGSGAYDHNGIIQEATRKAKELHKDLLIVADTCLC
EYTDHGHCGVIDDHTHDVDNDKSLPLLVKTAISQVEAGADIIAPSNMMDGFVAEIRKGLD
NAGYYNIPIMSYGIKYASSFFGPFRDAADSAPSFGDRKTYQMDPANRLEAFRELESDLKE
GADMMIVKPALSYLDIVRDVKNNTNIPVIAYNVSGEYAMTKAAAQNGWIDEERVVMEQMV
SMKRAGADMIITYFAKDICHYLDK
>ACAKHAOO_01671 ATP-dependent Clp protease ATP-binding subunit ClpX
MFKFNEDEENLKCSFCGKDQDQVKKLVAGSGVYICNECIELCSEIVEEELAQTTSEEFTE
LPTPKEIMDHLNEYVIGQEKAKKSLAVAVYNHYKRIQQLGPNEDEVELQKSNIALIGPTG
SGKTLLAQTLAKTLNVPFAIADATSLTEAGYVGDDVENILLRLIQAADFDIDKAEKGIIY
VDEIDKIARKSENTSITRDVSGEGVQQALLKILEGTTASVPPQGGRKHPNQELIQIDTTN
ILFILGGAFDGIDEVIKRRLGEKVIGFASNEADKYDEEALLEQIRPEDLQSYGLIPEFIG
RVPIVANLETLDVEALKNILTQPKNALVKQYTKMLELDNVELEFTEEALAAVSEKAIERK

TGARGLRSIIEEALIDIMYDVPSSEDVTKVVITDKTINDEVDPELYDSEGNLLNDSKTSA
>ACAKHAOO_01672 Trigger factor
MTATWEKKEGNEGLLKVTVPAEKVDKALDQAFKKVVKQINVPGFRKGKVPRPIFEQRFGV
EALYQDAVDILLPEAYGEAIDETGINPVAQPEINVTQIEKGKDFEFEATVTVEPEVQLGD
YKGLEIEKQDSELTDEDLQEAIDHSLGHLADMVVKEDGAVENGDTVNIDFDGYVDGEQFE
GGQADGYDLEIGSGSFIPGFEDQLVGVKTGEEKDVVVTFPEEYHAEELAGKKATFKTKVN
EIKYKEVPELDDEIANELDSDANSVDEYKENLRKRLSEQKAEEAENVEKEEAINKATDNA
TIDIPQAMIDTELDRMVQEFGQRIQQQGLDLQTYFQISGQDESQLREQMKDDAEQRIKTN
LTLSAIVDKENIEANDEDIDKELEKMSKQFNISVEDIKNTLGNTDIIKNDVRIQKVIDLL
RDNAKYVESTKEDK
>ACAKHAOO_01675 50S ribosomal protein L20
MPRVKGGTVTRARRKKTIKLAKGYFGAKHTLYKVAKQQVMKSGQYAFRDRRQRKRDFRKL
WITRINAAARQHDMSYSRLMNGLKKADININRKMLSEVAISDEKAFAELVSKAKEALK
>ACAKHAOO_01677 Translation initiation factor IF-3
MREERAFYARSFCLENFVKFWEVSTIAKDQTQVNEKIRAKELRLIGQNGDQIGVKSKREA
LEMAEHVELDLVVVAPNAKPPVARIMDYGKYKFEQQKKEKEMKKKQKVINVKEIRLSPTI
EEHDFQTKLKNGRKFLSKGDKCKVSIRFRGRAITHKEIGQRVLEKFADECKDIATVEQKP
KMEGRQMFIMLAPINEK
>ACAKHAOO_01679 Threonine--tRNA ligase
MDQINVQFPDGNSKAFDKGITTEEIAQSISPGLRKKAVAGKFKNQMVDLTRPLEEDGSIE
IVTPGSDEALEVLRHSTAHLMAQALKRLYGDVKFGVGPVIDGGFYYDFDMDEKVSSDDFE
KIEKTMKQIVDENHKIERKVVSREEAKSFFKDDPYKLELIDAIPEDESVTLYSQGEFTDL
CRGVHVPSTSKIKEFKLLSTAGAYWRGDSNNKMLQRIYGTAFFDKKDLKAHLEMLEERRE
RDHRRIGKDLELFTNNQLVGAGLPLWLPNGATIRREIERYIVDKEVSMGYDHVYTPVLAN
VELYKTSGHWDHYRDDMFPPMKLDETEEMVLRPMNCPHHMMVYNNRPHSYRELPIRIAEL
GTMHRYEASGAVSGLQRVRGMTLNDSHIFVRPDQIKDEFKRVVNMIQEVYSDFGFEDYTF
RLSYRDPEDKEKYMDDDEMWNKAETMLKEAVDEMGLPYVEAIGEAAFYGPKLDVQVKTAM
GKEETLSTAQIDFLLPERFELTYIGSDGENHRPVVIHRGVVSTMERFVAFLTEETKGAFP
TWLAPKQVEIIPVNVDLHYDYARNLQDELKSQGVRVEIDDRNEKMGYKIREAQMQKIPYQ
IVVGDKEVENNQVNVRKYGSQDQETVEKDEFIWNLVDEIRLKKHR
>ACAKHAOO_01689 Isocitrate dehydrogenase [NADP]
MSAEKITRINDGLNVPNEPIIPFIIGDGIGPDIWKAASRVIDAAVEKAYNGEKRIEWKEV
LAGQKAFDETGEWLPQETLDTIKEYLIAIKGPLTTPIGGGIRSLNVALRQELDLFTCLRP
VRWFKGVPSPVKRPQDVDMVIFRENTEDIYAGIEFKEGTDEVKKIIDFLQNEMGATNIRF
PETSGIGVKPVSKEGTERLVRAAIQYAIDNNRKTVTLVHKGNIMKFTEGAFKQWGYDVAH
NEFADKVFTWQQYDEIVEKDGKDAANQAQEKAEQDGKIIIKDSIADIFLQQILTRPAEHD
VVATMNLNGDYISDALAAQVGGIGIAPGANINYESGHAIFEATHGTAPKYAGLNKVNPSS
ELLSSVLMLEHLGWQEAADKITDAIEATIASKVVTYDFARLMDDATEVSTSEFADELIKN
LK
>ACAKHAOO_01690 Citrate synthase 2
MAELQKGLEGVIAAETKISSIIDSQLTYAGYDIDDLAENALFEEVVFLLWNYRLPTQDEL
DALREKFYHYMTLNPRVYKHFEEYATDNVHPMTALRTSVSYIAHFDPDAENETEENRKER
AIRIQAKIASLVTAFARVREGKEPVKPNPELSYAANFLYMLRGELPTDTEVEAFNKALVL
HADHELNASAFTARCAVSSLSDMYSGIVAAVGSLKGPLHGGANEQVMSMLADVKSVDEVD
AYLDEKFKNKEKIMGFGHRVYKDGDPRAKYLREMSRKITSETGRSELYDISIAIEKRMKE
EKGLIPNVDFFSATVYHSMDIPHDLFTPIFAVSRTSGWTAHILEQYRDNRIMRPRANYVG
ETNRKYLPIEER
>ACAKHAOO_01693 Pyruvate kinase
MRKTKIVCTIGPASESEEMLEKLMKAGMNVARLNFSHGSHEEHKARIDTIRKVADRLGKT
IGILLDTKGPEIRTHDMKDGLIMLEKDKEVIVSMSQVEGTPEKFSVTYEDLINDVQVGSY
ILLDDGLVELQVKDIDKTKGEVKCDILNTGELKNKKGVNLPGVKVNLPGITDKDADDILF
GIKEDVDYIAASFVRRPSDVLDIREILERENNHNITIFPKIENQEGIDNIEEILEVSDGL
MVARGDMGVEIPPESVPIVQKDLIRKCNKLGKPVITATQMLDSMQRNPRATRAEASDVAN
AIYDGTDAVMLSGETAAGLYPEEAVKTMRNIAVSAEAAQDYKKLLSDRTKLVETSLVNAI

GVSVAHTALNLNVKAIVAATESGSTAVTISKYRPHSDIIAVTPSEHTARQLALVWGAYPV
IKKGRKTTDDLLNNAVATAVETGRVTNGDLIIITAGVPTGEKGTTNMMKLHLVGDEIAKG
QGVGRGSVVGKTVVANSASDLEGVDLSESVIVTNSVDETLVPYIEQAVGLITEENGITSP
SAIIGLEKSIPTIIGVENATKELKDGILVTVDAAQGKIFEGYANVL
>ACAKHAOO_01694 ATP-dependent 6-phosphofructokinase
MKKIAVLTSGGDSPGMNAAVRAVVRKAIYNNIEVYGIYQGYQGLLDDNIHKLELGSVGDT
IQRGGTFLYSARCPQFKEASVRQKGIENLHKRGIEGLVVVGGDGSYRGAQRISEETKDIK
TIGIPGTIDNDINGTDFTIGFDTALNTIIDSVDKIRDTASSHARTFIVEVMGRDCGDLAL
WAGLSVGAETILVPEANTDIKDIAEKIESGIKRGKKHSIVMVAEGSMSGQECADQLMKYI
NVDARVSVLGHIQRGGSPSGADRVLASRLGGYAVDLLMQGRSGQGVGIMNNKLTATPFDE
IFNGNDHKFNTDIYELAKELSI
>ACAKHAOO_01701 Stress response protein NhaX
MYKNILLGVDTQLKNEKALKEVSKLAGPDTVVTILNAIGEQDAQASIKAGTHINELTEKR
SQLLEKTRTTLEDYGIDYDQIIVRGNPKDELVKHANSGKYEIVVLSNRKAESQKKFVLGS
VSHKVAKRATIPVLIVK
>ACAKHAOO_01703 putative peptidase
MSKIEKITKQLQHEQADAAWITTPLNVFYFTGYRSEPHERLFALLITANGDQTLYCPKME
VEEVKNSPFEGKIIGYLDTENPFEIDPLSFNKLLIESEHLTVKRQRELTQNFGVQHYGDI
DQTIKELRNIKNESEIENIREAAKLADKCIEIGTEFLKVGVTEREVVNHIENEIKKFGVS
EMSFDTMVLFGDHAASPHGTPGERKLVKDEYVLFDLGVIYNHYCSDMTRTVKFGTPSEEA
QTIYNIVLEAETNAIEAIRAGVPLQDIDKIARDIISDAGYGDYFPHRLGHGLGLEEHEYQ
DVSSTNSNLLEAGMVITIEPGIYVPNVAGVRIEDDILVTENGYEILTHYDK
>ACAKHAOO_01705 Putative universal stress protein
MLTYKNILIAVDGSHEAEWAFNKAVDVAKRNDAKLTIVNIIDSRTYSSYEVYDAQFTEKS
RSFSEELLKGYQEVATRAGVTNVETRLEFGSPKAIIPKKLASELGVDLIMCGTSGLNAVE
RFIVGSVSEAIVRHAPCDVLVVRTEEIPEDFQPEVATPEFRKQYS
>ACAKHAOO_01706 Acetate kinase
MSSLVLAINAGSSSLKFQLIRMPEETLVTKGLIERIGIKDSIFTIEVNGEKIKDVKDIKD
HEEAINIMLDSFKQHGIIDDINDIAGTGHRVVHGGELFPTSALVTDKVEEQIESLSELAP
LHNPANLMGIRAFRKLLPNIPHVAVFDTSFHQSMPEQSYLYSLPYQYYKDYGIRKYGFHG
TSHKYVSQRAAEIMNKPIEELRIISCHIGNGASIAAIDGGESIDTSMGFTPLAGVTMGTR
SGNIDPALIPFIMQKTGQNAEEVLNVLNKESGLLGISGTSSDLRDLESDAEEGKERAQLA
LDVFASRIHKYIGSYATRMHGVDVIVFTAGVGENSSTVRAKVLEGLEFMGIYWDPKKNET
IRGEEGFINYPHSPVKVIVIPTNEEVMIARDTVKFGEL
>ACAKHAOO_01708 Thiol peroxidase
MTQITFKNNPIHLAGSEVSEGQHAPDFKVLDNDLNEVSLENYKGQKKLISVVPSIDTGVC
DQQTRKFNEEAAQEDGVVLTISADLPFAQKRWCASNGLDNVITLSDHKDLSFGQQYGVVM
EELRLLARSVFVLDSNDKVVYKELVSEGTDFPNFDAALEAYRNI
>ACAKHAOO_01712 Septation ring formation regulator EzrA
MVLYTILAIIIIVLIIVGIMFYLRSNKRKIVEEAEERKLKVQRLPFEENLKQLSELNLKG
ETRKKYDAFKRDTLDYTNNYLAPVEEKIHDAEIQLDKFQFSGAQSDIDDAHELMDKYEAG
YQQQVEDVNEIVSLHKENEQVYEKCKTNYREMKRDVLANRHQFGDAASPLEKRIESFEPE
LEQYEELKEEGNYVQAHNHIMGLNESMNEVQKYMDEIPELIREAQKELPGQFQDLKYGCR
DLKMNGYDLDHVKIDGTLQSLKTELNFVEPMISRLELDNANDKLEQINDKLDEMYDLIEH
EVKAKNEVEETKEQITDELFKAREMNYTLRTEIDYIRENYFINESDVHSIRQHENEIQNL
VAVYDEILKEMAKSAVRYSEVKDNLEYLDDHVKVINENQEKLQNHLIQLREDEAEAEDNL
LRIQSKKEEVYRRLLASNLTSVPERFIIMKNEIDHEVRETNDQFSERPIHVKQLKDKVAK
IVIQMNTFEDEANDVLVNAVYAERLIQYGNRYRKDHNNVDKSLNEAERLFKNNRYKRSIE
ISEQALESVEPGITKYIEDEVAKG
>ACAKHAOO_01714 30S ribosomal protein S4
MARFRGSNWKKSRRLGISLSGTGKELEKRPYAPGQHGPNQRKKLSEYGLQLREKQKLRYL
YGMTERQFRNTFDIAGKQHGVHGENFMILLASRLDAVVYSLGLARTRRQARQLVGHGHVE
VDGRRVDIPSYSLKPGQVITVREKSQNLDIIKESVEINNFVPEYLDFDADSLKGTFVRFP
ERSELPAEINEQLIVEYYSR

>ACAKHAOO_01718 D-3-phosphoglycerate dehydrogenase
MKHNILVSDPISTDGLQSLLNHSDFNVDIKTDLDEQSLLDIIGDYEGLIVRSQTQVTQQV
IEKASNLKVIARAGVGVDNIDIDAATLQGILVINAPDGNTISATEHSVAMILAMARNIPQ
AHASLKNKEWNRKAFKGVELYQKTLGVIGAGRIGIGVAQRLQSFGMKVLAYDPYLTEDKA
QQLGVKLATIDEIARQADFVTVHTPLTPKTRGIVNADFFSKAKPTLQIINVARGGIINED
DLLNALNNNQIARAALDVFEHEPPTDSPLIEHDKIIVTPHLGASTIEAQEKVAVSVSEEI
IDILENGNVTHAVNAPKISFNDIDEITQQWIEIGELSGELAIQLIEGAPREIKVTFNGDV
AKQETDLITRSIVKQILQQDLGDRVNIINAFALLNEQGVTRNVEKRASQDTFSNYIQVHL
VSDTEEVKIGATVIAGFGARIVRINDYSVDFKPNAYQLVSYHGDKPGMVGLTGQLLGRHN
INIASMSLGRNIQGGQAMMVLSIDQPVTEDIINELYDVGGFDKIYGTTLSVK
>ACAKHAOO_01721 1-acyl-sn-glycerol-3-phosphate acyltransferase
MYQFISRLLDLILVKMAKSLYVIGKENIPKDNKYVVTCTHESYNEVIMLGMALVPNEIHY
MAKKELFNNKWFGQFLTSLNAFPVDRENPGPSTLKRPVNLLKENKTVGIFPTGHRTSAEG
APLKRGASTIAMLGKAPILPAAYVGPKKLHGLITGQALIKFGEPIYQSDIPKDLKRNEKI
DYLTHEIERRTAQLQKELHLIQDSLEK
>ACAKHAOO_01722 Serine protease Do-like HtrA
MSEFNQENQSIYNNYQQPQPGKPKFPWFKTIIVALIAGVIGALLVLGASKIMGLVGIDNG
GAQVQEANNSKGGNVLDGKSDKYKSVNAMIKDVSPAIVGVINMQKANGLEDFLQGKSSSE
SEEAGIGSGVIYQINNNSAYIVTNTHVISGASEIKVQLHSGKQVKAKLIGKDTVSDIAVL
KIDNTKGIKSIKFANSSKVQTGDSVFAMGNPLGLEFANSVTSGIISANERTIESNTTSGG
TKVNVLQTDAAINPGNSGGALVDINGNLVGINSMKIAAEQVEGIGFAIPSNEVKVTIEQL
VKNGKIERPSIGIGLLNLSDIPDSYKKELNTDRDDGIYVAKVSRSSELKTGDIITKIDDK
TVKEDTDLRTYLYQNKKPGETAKLTVIRDGKTLTVNVNLKSQSDISSNQSSDSESDNSRN
SQFIQ
>ACAKHAOO_01725 Tyrosine--tRNA ligase
MSNALIEELQWRGLIYQQTDESSIEELLNKEQISLYCGADPTADSLHIGHLLPFMTLRRF
QEHGHRPIVLIGGGTGMIGDPSGKSEERILQTEDQVEANVEGISAQMHKLFEFGTDKGAI
LVNNKDWLSQISLISFLRDYGKHVGVNYMLGKDSIQSRLENGISYTEFTYTILQAIDFGH
LNRELNCKLQVGGSDQWGNITSGIELMRRMYGQTEAYGLTIPLVTKSDGKKFGKSESGAV
WLDADKTSPYEFYQFWINQSDDDVIKFLKYFTFLDKDEINRLEESKNQEPHLREAQKALA
ENVTEFIHGKEALDDAIRISKALFSGDLKSLSGKELKEGFKDVPQVELSTETSNIIDVLI
ETGIATSKRQAREDVNNGAIYINGERQQSVDYELSNEDKIDDEFTIIRRGKKKYFMVNYK
>ACAKHAOO_01727 Formate--tetrahydrofolate ligase
MAHLSDLDIANQSELKPIGEIAEKAGIPADALEQYGHYKAKIDINQIKPKDNKGKVVLVT
AMSPTPAGEGKSTVTVGLSDAFNELKKNVMVALREPALGPTFGIKGGATGGGYAQVLPME
DINLHFNGDFHAITTANNALSAFIDNHIHQGNELGIDVRRVEWKRVLDMNDRALRHVNVG
LGGPTNGVPREDGFNITVASEVMAILCLARNINDLKEKISRITIGYTRDRKPVTVADLKV
EGALAMILKDAIKPNLVQTIEGTPALVHGGPFANIAHGCNSILATETARDLADIVVTEAG
FGSDLGAEKFIDIKAREAGFEPSAVVLVATVRALKMHGGVAKDDLKEENVEAVKAGIVNL
ERHVNNIRKFGVEPVIALNAFIHDTDAETEAVKAWAKENNVRIALTEVWEKGGKGGVELA
NQVLEVIEQPNDFKFLYDLDQSLEEKIETIVKDIYGGSSVTFSKKAKKQLKEFTDNGWGQ
YPICMAKTQYSFSDDATALGAPTDFDITIRELEAKTGAGFIVALTGAIMTMPGLPKKPAA
LNMDVTEDGHAVGLF
>ACAKHAOO_01732 Protein AroA(G)
MSTKLEQYREEIVSINNQILELLSKRGELAQKIGEEKIKQGTKVYDPQREKEMINELMDR
NQGPFNDNVIKQLFKEIFKASTDLQKSENEKHLYVSRKLKPEDTIVKFDNGGIIGDGNKS
FVFGPCSVESQEQVDAVAKDLQARGEKFIRGGAFKPRTSPYDFQGLGIEGLKILKNVKDK
FGLNVVSEIVNPADFEVADEYLDVFQIGARNMQNFELLKEAGRTNKPILLKRGLSATIEE
FTFAAEYIASQGNKNIILCERGIRTYEKATRNTLDISAVPILKQGTHLPVMVDVTHSTGR
KDIMLPTAKAALAVGADGVMAEVHPDPSVALSDSGQQMDLNEFDQFYNELKPLAELYNSK
KLK
>ACAKHAOO_01733 hypothetical protein
MSKKYNRDAFETTNSGNDLHGKGANQQDNTSQSNATKHYDRDPFATSNTGSDLHGQGANQ
QDSAAQSNATKHYNRDEFEKNNFGHDLHDNGPNQKNGKSRQGLNTEHYNRDEFVTNNTGK

DLHGQGENQNSNNYDVSNSQYSHNKFSSHPSRKDFVISFITGALIGSAVGLFYKNKAEEK
IDGAKTKEKELRNRYQNIKQQTESNIENVKQKIDDFKNRDNSEVSNDELVAQQNAIKAET
SNNLADQSPQAQEIQEAKAEAKKDSKSKEISATELAAQQNAIKAETSDDLSDQSPQAQEI
QEAKADEIQNLRNNEDNSAEEIVAQQNAIKAETSDDLSDKSPQAIEIQEAKAETEKEEEN
EPKEVKVSATELTAQQNAVKVESSNNNLSDSSIKDNASHNKHVTAKNLASAAKTKKSKLD
NDLNVANKTKTLLEEPSVAKSIGNKTVPNLVTKNNNTFNNDEDQVKNNQAHATSKFENGV
ITHDTKSNNEKSTGNSKQSQNEPKAKNKTPKQQQRAEKAKSKIDKRTFND
>ACAKHAOO_01734 hypothetical protein
MDWILPIAGIIAAIAFLVLCIGIVVVLISVKKNLDHVAKTLDGVEGQVQGITRETTDLLH
KANRLTEDIQGKVERLNSVVDGVKGIGDSVQNLNGSVDRVTNSITHNISQNEDKISQVVQ
WSNVAMEIADKWQNRHYRRGSANYRNTSVGNDANHSNENYTTNVEKNF
>ACAKHAOO_01736 DNA translocase SftA
MSWFDDLFSNKENSDEELLRRKSKRRNGDLTQNKDDSLLPENNDIYDRPRGKFRFPIDVG
QDENYEEAIYRDSNDDIGSYRPHSSNDHSSYDSFDYEGHTNQSNNYAKYDSSEDSRRRRR
RNHINQDDTGIPSIKSRSPKSSNKYIDTRESQRFNKHQNNYSSTNVSNYSKANSNHQSRV
KLQSERFKSNYHLSSEPTYHRSSFKTSEVPSAIFGTKKRRPIENGVIPPVKDDEDSKESI
TKYSSSTVEHVPHESNHAIADNSNKEDTKRSSQLDSSITIENESTIESSSNTSNNNERTP
NYSKRDNTVNIENIYASQIVEEIRKERERKVQQKRKFKEALQNKRQQTDEEDSIQRAIDE
MYAKQAEQYTGESSLNQGDNVSNKSNESEIDKSKHSYHSKDKSLADEHNRLVQNQTDEQT
NSDNVDNQTEVSNESLEPYNYEEIDLNQVSSVQQVRQDDVQVKDVLEEQSSKINNNKVES
YSNEKFDDYLEDTNSHEEMLHDDDLHEQVMDDDENEGISNKTTDENNDEKIDDANYREIN
ESESLMQDKANDLKFNDEVNNSENQQNSSENNINNAVRNAVSSDIEYATNEDEENDERLA
QDTNKEDQKLSQSEDIQHESLNNEDVSLTSNKTDDSEHLEKDSLNEDKKAEPSFNKTNKA
PQKMSIKPGSKPFNVVMTPSDKKRVMDAKKNSVSRNKVNVPELKPETKKEAQDEKMNAEF
DNHLNESQLNSDESSDFNVASLEYNESSEHSVEKDNIINDENTRENEHQDVDNSQNNDMP
KGNQFSKVQNSNNQNDNKHDINEFVSKEGYSEVTSTKNHKDGDDANHKAPIRRGPNIKLP
SLDLLEDHEEHEIDESWIEEKKQELNDAFYYFNVPAEVQNVTEGPSVTRFELAVEKGVKV
SRITALQDDIKMALAAKDIRIEAPIPGTSLVGIEVPNLNPTKVNLKSILESPKFKNAESK
LTVAMGNRINNEPLLMDIAKTPHALIAGATGSGKSVCINSILMSLLYKNHPEELRLLLID
PKMVELAPYNDLPHLVSPVITDVKAATQSLKWAVDEMEKRYKLFAQFHVRNITAFNKKAP
YEQRMPKIVIVIDELADLMMMAPQEVEQSIARIAQKARACGIHMLVATQRPSVNVITGLI
KANIPTRIAFMVSSSVDSRTILDSGGAERLLGYGDMLYLGSGMNKPIRVQGTFVSDDEID
DVVDFIKDQREPDYLFEEKELLKKNQTQAQDELFDDVCEFMVKEGHISTSLIQRHFQIGY
NRAARIVDQLEQLDYISGANGSKPRDVFITEADLKKE
>ACAKHAOO_01737 Phenylalanine--tRNA ligase beta subunit
MNLFYNKDGVGDVAFLQIEPTDGPFEYKKQGDIVEISKEGTIVGFNIFEFSRYNKISGNG
HIKLTSELVDAVQKAINKSGLDYQLNADLSPKFVVGYVETKEKHPDADKLSVLKVNVGNE
HLQIVCGAPNVEAGQKVVVAKVGTVMPSGMVIKDAELRGVASSGMICSMKELNLPNAPQE
KGIMVLSNDYEIGQAFFD
>ACAKHAOO_01745 D-alanine aminotransferase
MTKVFINGEFIDQNEAKVSYEDRGYVFGDGIYEYIRAYDGKLFTVTEHFERFIRSASEIQ
LDLGYTVEELIDVVRELLKVNNIQNGGIYIQATRGVAPRNHSFPTPEVKPVIMAFAKSYD
RPYDDLENGINAATVEDIRWLRCDIKSLNLLGNVLAKEYAVKYNAGEAIQHRGETVTEGA
SSNVYAIKDGAIYTHPVNNYILNGITRKVIKWISEDEDIPFKEETFTVEFLKNADEVIVS
STSAEVTPVVKIDGEQVGDGKVGPVTRQLQEGFNKYIESRSS
>ACAKHAOO_01746 Putative dipeptidase
MWKEKVQEYEGQIIDDLKGLLSIESVRDDSKASDETPVGPGPRQALDYMYEIAQRDGFST
HDVDHIAGRIEAGKGDDVLGVLCHVDVVPAGDGWDSDPFNPVVTDDAIIARGTLDDKGPT
IAAYYAVKILNDMKVDWKKRIHIIIGTDEESDWKCTERYFQTEEMPTLGFAPDAEFPAIH
GEKGITTFDLVQNSTSEDQDEPDYELISFESGQRYNMVPDHAQARVFVKENMTDVVQHFE
HYLDQHKLQGESVVDSGELVLTLEGKAVHGMDPSLGVNAGLYLLDFISTLNLNQTAREFV
DFSNRYLHESHFGEKMGMKFHTDVMGDVTTNVGIISYDNKQGGRFGINLRYPQKFEFEEA
IQRFTKEIKAYGFDLELGKVQQPHFVDKNDPFVQKLVKAYRNQTGDMSEPYTIGGGTYAR
NLDKGVAFGAMFEDSEDLMHQKNEYITKKQLFNATSIYLEAIYSLCVEG

>ACAKHAOO_01747 hypothetical protein
MAKTTGLFRIIAGVGAAAVAVVLSRKESRDKLKEQYNKYKEDPEGYKENARGLASQLSSK
ANETIQEVRNNPQDYANRLKNDPKSFLEEEKSKFTNLDANKEDSLEEGKFDDEGGATVNN
NLRVVTEEDLKKNNNALEDKD
>ACAKHAOO_01752 Extracellular matrix-binding protein ebh
MNLFKKQKFSIRKFSVGIFSTVIATLTFLSHPGHAATQEEESNSSQATSQNVKSQTDDKS
IHNNDNYIENEKNSNDSIKEIATAIKINNSEINSKVDNANGTYRIDNEEIKENSIETNQD
EKTIEPELSHQNISKNDSNNTFNENKSIHKRTKRSTDNNVDNNQDQMSINETNSGQIING
TFTDTSNGAVIPTNQTVSEMNQAGKITGWHVKDNDQTEIPLVWGPKALPPYNPYVFDKTN
NKIAAVLSKYPNNLGGVAGDKTVGPIYQDVDVTPGSEVQLYFIGTSMGNTNGINGVKVSI
YDSNNPTDLLYSGRPNTASKSFGVFTGVFNVPNNVSRLRFMFETFEKRSYESANGGRILK
GKNNFGGSGLADVKVNSGAYLKATTTQTKYTVTSPSTSTSLINATLEVSLENKGHSRSNK
TQYKVVLPEGVQFISAQNARANYNSSNRLLTLNVDRIEPGTIKNITYTVSLPTSEPIKKD
FDATLVYVTDGINMNRRNGSRDFGSNADDNYFRYGGYNEYVKTESNQRRGNVTVATQSVT
VEMYKTDLQQKYNQISAQIEHLNPSDYSQEAWSSMQNVLNASRQILNENDNTPINDRKNQ
ATINDLTLRLDKERAKLDVDQAAKSRKEVINNNNDATREEKDTALTLLKATFDEKVRDIE
SATNINEISERKKQAIVDISPIDVIPAIKQQAIQSLNDKADEKLIEIENDTNATFEEREE
AKTSVNEALTNAKNAVRNATTNQLVETVLTSNSNNISQISTHAIARDKARQELDRVIASK
MSEIDNDHSATIEEKNETKAKIDEIAKQARLNIERGVHNDDVKVAKDKALNQLHNVQVDA
IKKNQAKQIITDQANSKKAEIDQTPNATDEEKAAAKAKVDEAVTTAKNAIDQATNNDGVD
TAKTNGVDTINNVQPTVVKKDEAKTAIDKAAEAKKAEIDQTPDATDEEKAAAKAKVDEAV
NNAKASIDQATNNNGVDTAKSEGSDAINHVQPVVVKKDEAKTAIDKAAEAKKAEIDQTPN
ATDEEKIAAKAKVDEAVTTAKNAIDQATNNAGVDTAKSNGLDSINNIQPTVVKKDEAKTA
IDKAAEAKKAEIDQTPNATDEEKAAAKAKVDEAVTTAKNAIDQATNNNGVDTAKTNGVDA
INNVQPTVVKKDEAKTAIENAARAKKAEIDQMPNATDEEKAAAKAKVDEAVNNAKVSIDQ
AINNNGVDTAKTNGVDSINNVQPTVVKKDEAKTAIDKAAEAKKAEIDQTPNATDEEKAAA
KAKVDEAVNNAKASIDQATNNDGVDTAKSNGLDSINNIQPTVVKKDEAKTAIDKAAEAKK
AEIDQTPNATDEEKAAAKAKVDEAVNNAKASIDQVTNNEGVDTAKSNGLDSINNIQPTVV
KKDEAKTAIDKAAEAKKTEIDQTPNATDEEKAAAKAKVDEAVTTAKNAIDQATNNAGVDT
AKTNGVDSINNVQPTVVKKDEAKTAIENAARAKKAEIDQTPNATDEEKVAAKAKVDEAVN
NAKASIDQATNNDGVDTAKSNGLDSINNIQPTVVKKDEAKTAIDKAAEAKKAEIDQTPNA
TDEEKAAAKAKVDEAVTTAKNAIDQATNNAGVDTAKSNGLDSINNIQPTVVKKDEAKAAI
DKAAEAKKAEIDQTPNATDEEKATAKAKIDEAVNNEKASIDQATNNDGVDTAKTNGVDAI
NNVQPTVVKKDEAKTAIENAARAKKAEIDQTPNATDEEKATAKAKVDEAVTTAKNAIDQA
TNNNGVDTAKTNGVDAINNVQPTVVKKDEAKTAIENAARAKKAEIDQMPNATDEEKAAAK
AKVDEAVNNAKVSIDQAINNNGVDTAKTNGVDSINNVQPTVVKKDEAKTAIDKAAEAKKA
EIDQTPNATDEEKAAAKAKVDEAVTTAKNAIDQATNNAGVDTAKTNGVDSINNVQPTVVK
KDEAKTAIENAARAKKAEIDQTPNATDEEKVAAKAKVDEAVNNAKASIDQVTNNEGVDTA
KSNGLDSINNIQPTVVKKDEAKTAIDKAAEAKKTEIDQTPNATDEEKAAAKAKVDEAVTT
AKNAIDQATNNAGVDTAKTNGVDSINNVQPTVVKKDEAKTAIENAARAKKAEIDQTPNAT
DEEKAVAKAKVDEAVTTAKNAIDQATNNNGVDTAKTNGVDAINNVQPTVVKKDEAKTAID
KAAEAKKAEIDQTPNATDEEKAAAKAKVDEAVNNAKASIDQATNNNGVDTAKSEGTDAIN
HVQPVVVKKDEAKVAINKAAEAKKAEIDQTPNATDEEKAAAKAKVDEAVTTAKNAIDQAT
NNAGVDTAKTNGVDSINNVQPTVVKKDEAKTAIENAARAKKAEIDQTPNATDEEKVAAKA
KVDEAVNNAKASIDQVTNNEGVDTAKSNGLDSINNIQPTVVKKDEAKTAIDKAAEAKKTE
IDQTPNATDEEKAAAKAKVDEAVTTAKNAIDQATNNAGVDTAKTNGVDSINNVQPTVVKK
DEAKTAIENAARAKKAEIDQTPNATDEEKVAAKAKVDEAVNNAKASIDQATNNDGVDTAK
SNGLDSINNIQPTVVKKDEAKTAIDKAAEAKKAEIDQTPNATDEEKAAAKAKVDEAVTTA
KNAIDQATNNDGVDTAKSNGLDSINNIQPTVVKKDEAKAAIDKAAEAKKAEIDQILNATD
EEKSAAKAKVDEAVTTAKNAIDQTTNNVGVDAAKESGVESINQVQPAVVKKDQAKAEIDN
VAQAKKAEIDRNSNATEEEKVAAKSKVDEAATTIKQAIDKAVNNSEVDNAIDVGKTAINN
IEADNSAKSKAIKHLQELVKQQMTKIDSNHLATEEEKAKAKQMIKLLFEKAKIEIEKAKT
SYEVTKIDAEYSKLITKTLPENKAKLNAKKKIEKIARQLKNKLNNMNGVSKEEKDRIKVI
IEQIVKKSFKDIDLASRNNTINKIVNDVKIQFANIKINKQNNKKSLINNENASVIITTEQ

HKTNKAYHKVRNEKGRYQLPNTGINNDTSSPLISFTFVSGLFLILRSMRRRASK
>ACAKHAOO_01756 Leucine--tRNA ligase
MGYNHKEIEKKWQNYWADNKTFKTSDNLGQKKFYALDMFPYPSGAGLHVGHPEGYTATDI
VSRYKRMQGYNVLHPMGWDAFGLPAEQYALDTGNDPREFTKQNIQTFKRQIQELGFSYDW
DREVNTTDPEYYKWTQWIFIQLYNKGLAYVDEVAVNWCPALGTVLSNEEVVDGVSERGGH
PVYRRPMKQWVLKITEYADRLLEDLDELDWPESIKDMQRNWIGRSEGARVSFEIENKDAS
IDVFTTRPDTIYGTTFLVLSPEHSLVNEITSEDKLEAVKKYQEDSSKKSDLERTDLAKDK
SGVFTGAYAINPLTGKKLPIWIADYVLSSYGTGAVMAVPAHDERDYEFASKFNLPINEVI
AGGDIQKEAYTGVGEHINSGELNGLDNETAISKAIELLVAKGAGEKKVNYKLRDWLFSRQ
RYWGEPIPVIHWEDGTMTTVPEEELPLLLPETDEIKPSGTGESPLANIDEFVNVIDEKTG
MKGRRETNTMPQWAGSCWYYLRYIDPHNSNMLADPEKLKHWLPVDLYIGGVEHAVLHLLY
ARFWHKVLYDLGVVPTKEPFQKLFNQGMILGEGNEKMSKSKGNVVNPDDIVDSHGADTLR
LYEMFMGPLDAAIAWSENGLDGSRRFLDRVWRLFINEDGSLSNKIVENNDNGLDKVYNQT
VKKVTEDFNTLNFNTAISQLMVFINDCYKAETIYQPYAEGFVKMLAPIAPHIGEELWDRL
GNEDTITYQPWPTYDESLLVDSEVEIVVQVNGKVRAKLNIPKDTSKDEMEALALKDENVK
LSIEGKDIKKVIAVPQKLVNIVAK
>ACAKHAOO_01770 Bifunctional autolysin
MTKHKKGSIISLVGLLIVLVAAGFIFFTMISDQIFFKKVNEEEKVEHLNVTLNKAADKQI
DNYTSQQVSNKNNTAWRDASDNEIKAAMDSSKFIDDDKQKYQFLDLSKYQGIDENRIKRM
LFDRPMLLKHTDAFISAAKEKHVNEVYLISHALLETGSVKSELANGVEIDGKKYYNFYGV
GALDSDPIKTGSEYAKKHGWDTPEKAIKGGADFIHQHFLSHDDQNTLYSMRWNPKNPGEH
QYATDIKWAESNAQIIADFYKEMKTEGKYFKLYVYKDDNKHQK
>ACAKHAOO_01776 Transaldolase
MAKLNVEVFADGADIEQMKAAYKNKEVDGFTTNPSLMAKAGVTDYKSFAEEAVKEIPDAS
ISFEVFADDLETMEKEAEILKQYGDNVFVKIPVVNTKGESTIPLIKKLSADNVRLNVTAV
YTIEQVKEITEAVTEGVPTYISVFAGRIADTGVDPLPLMKESVDVAHSKEGVKLLWASCR
ELFNVIQADEIGADIITCPADVVKKVNTNLGRDINELSVDTVKGFAKDIQSSGLSIL
>ACAKHAOO_01780 Glyoxal reductase
METIEFYNGHTMPKVGIGTFRVENNDECKEAVKHAIVSGYRSIDTAKVYGNEEQVGLGIK
EGLEATGLERKDLFITSKLYFEDFGRENVANAYETSINKLGLDYLDLYLVHWPGTNEAIM
IDTWKGMEDLYKDDKVKNIGVSNFNAEHFEALLAQVSIKPVINQVEFHPYFTQNKLRKYL
EVQNIHMESWSPFMNAQILGDETLNQIGKEVNKSAAQVVIRWNMQHGVIVIPKSVTPQRI
EENINVFDFELTDEQMEQIDSLNKDQRIGPDPATFEGH
>ACAKHAOO_01781 hypothetical protein
MNSFGPIEIGLIVAIVVAIICLILFLVTLKSKNNIKQKTKEEYDLKEKQMKASHDEALEK
ERIQNKKQVTKQKEDFDATVSGKDREIDALKLFSKNKSEYVTDMRLIGIRDRLVKEKRIR
PEDMHIMANIFLPSNEFNEIERISHLVLTRTGLYIIDSQLLKGHVYQGISGKQFNELPMM
EQVFSTLDLDVKSPQTLVLDQNEDKSSLSFVNYSEQLTSIEKLASDLQTQLGAKYTPTAI
LYFNPKHDGDVTISNYAQSSSVKVLVGPDQLDEFFNKFVFHGRIQYNVDELQNVMDKIES
FN
>ACAKHAOO_01782 S-adenosylmethionine synthase
MTYNKRLFTSESVTEGHPDKIADQVSDAILDEILKDDPNARVACETTVTTGMALISGEIS
TSTYVDIPKVVRETIKGIGYTRAKYGYDYQTMAVLTAIDEQSPDIAQGVDKALEYRNDIS
EEEIEATGAGDQGLMFGYATNETETYMPLPIFLSHQLAKRLSDVRKDGILNYLRPDGKVQ
VTVEYDESDKPKRIDTIVVSSQHADDIELEQIQSDIKEHVIYPTVPEGLIDNETKFFINP
TGRFVIGGPQGDAGLTGRKIIVDTYGGYARHGGGCFSGKDPTKVDRSAAYAARYVAKNIV
AAGLADQCEVQLAYAIGVAEPVSISIDTFKTGKVSEAQLVEAVRANFDLRPAGIIKMLDL
KHPIYKQTAAYGHFGRTDVLLPWEKLDKVNVLKDAVQA
>ACAKHAOO_01806 hypothetical protein
MKISRVLFGITAGVASGIAVSWLNRDDQSVKNNTIDAKEPTGSRSELEREIESLKRNFFN
IIDYGKQVKNDGVSYGSEIGDEFKTLIGDFKSDINPNIEKLQSHIENLQNRGEEISNTFS
KDNK
>ACAKHAOO_01808 Foldase protein PrsA
MKIMNKLIVPVTASALLLGACGSNATDSESNTLISSKAGDVKVKDVMNKIGDEQIASTSF

SIALNKILADKYKDKVDTKDIDDEIKKEQKQYGGKDQFESMLKQQGMSLDDYKEQKKLAA
YQKQLLADKVKVSDKELKENTKKASHILIKVKSSSSDKEGLSDKKAKEKAEKIQKEVEKN
PDKFGEIAKKESMDSSSAKKDGSLGYVVKGQMVSKFEKALFKLKEGQVSDVVKTDYGYHV
IKADKESDFDKQKSKLKAKLIEQKVQKDPKILTNAYKDLLKEYDVDYKDSDIKKAVENTI
LNPEKLKQQQSSSDSSSAASGLSS
>ACAKHAOO_01810 putative protein YhaN
MIIKSLEIYGYGQFVHRKIEFNREFTEIFGENEAGKSTIQAFIHSILFGFPTKKEKEPRL
EPRMGNQYGGKLTLILDDQSEIVVERVKGNISGDVKVYLENGLIRDEAWLKKKLNYISKK
TYQGIFSFNVLGLQDIHRNLDEDQLQSYLLEAGALGSSEFTMMNKMVSQKKSTLYKKAGK
NPILNQQLDELKSLEAQIREEESKLDEYHRLVDDRDKSQRHLEHLKANLNQLSKMHESKQ
KQLAIHEQAQEWKDLEQKLNIEPLSFPEKGIERYETASNYKQSLARDISLREEKLKQLEN
EFNAIPALNEDTANHIYHLTRDENEIQQAEMKSSSLKQEIDDYERQEADLKTSIGWQEVY
HNTDTSESMKNYVSKTIKTRNEQKVLKQQIERAIDENQIEQNTLSQEIENLEKDLVSEDS
LDKKKQYNQQKLELHEKENLYNKLKDTFNSDKERKQKRNWWLRLSFIVLSIVGLGLTLFS
FVTQNLIFGVIFAILTVIFIVGTFLVKTKEIDYSESISEEINDLEQQINDLESNYDLNFD
LDQQFQLRERWSNASNNKSVLANKLNHQENLLQNTEQQINDLTQKLNAVKENVKVPNEMT
DDLLIDSFKTMNQLKSNDSYRSKLLDQHQQVNQKLTHFYDKALKNTENEITPFNKASLFN
DMKNWISKYESNKEKKEQLKSQIELLSNELKQLKEQLEENEKTINTLFTYINVSSEEAYY
QSYEQYQLYHQQLARFKDLTAYLENQNFSYEDSSNLSEKTTAQLEEEDALLAKQVDAYND
QYLDKQTEVSDLTAQINYMETDKTLSKLRHEYYNLKNRMNDLAKDWASLSYLEALINEHI
KQIKDKRLPQVINEAVNIFTALTQGNYNMINYENDTIMVKHNNGQMYHPLELSQSTKELL
YIALRISLIKILRPYYPFPIIIDDAFVHFDKQRKSMMLNYLRELAKDYQVLYFTCMKDTA
IPSKEMLILNKLEEGGKR
>ACAKHAOO_01812 hypothetical protein
MAVNLYDYANQLEQALRDSDEYKAIKDAFSKVKDNEESKKLFDEFRETQLSFQQKQMQGE
EIPEEDLAKAQEQAQAIEKDENISELMQAEQKMSQVFQEINQIIVKPLDEIYAD
>ACAKHAOO_01821 Glucosamine-6-phosphate deaminase
MAMNFKVFNDVEHVAEYTADIIRKQFNNNPTTIAGIHLTKDAAPVLDELKKDVDHNAVDF
SQVNILDYDDNRSYYEALGVPASQIYPINLDDDAESLIDDKIKTKENKGKLILQVTSIDE
SGSLNVNVRQGLLKAREVVLVVTGANKREVVKKLYEENGKSSFEPSDLKAHRMVTVVLDR
AAAEGLPEDVKEYFTARFA
>ACAKHAOO_01875 putative protein
MTKKVAIILSNEFEDIELTSPKEAIEEAGFETEIIGDTANAEVVGKHGEKVIVDVSIADA
KPEDYDGLLIPGGFSPDHLRGDAEGRYGTFAKYFTKNDVPAFAICHGPQILIDTDDLNGR
TLTAVLNVRKDLSNAGANVVDESVVVDKNIVTSRTPDDLDDFNREIVKQLQA
>ACAKHAOO_01881 Aminopeptidase PepS
MTSLSYNEKLQQYAELLVKIGMNVQPKQPVFIRSSVDAIDLTRLIVEESYKAGASDVKVN
YSDSKLNRLKFEYESVDYFENQAVKSYEVDERMDYANRGAANLALLSGDPNLLNGIDPEK
LKANQISYSQAFKGYMEGSQKNRFPWVVAAFPSKDWARRVYPDLDEEIALEKFIDEVFDI
VRIDGNDPIENWKKHIENLSVHAKLMQEKNYKALHYQSEGTDLVVGLPKGHIWEDATSYV
NGNQQAFIANLPTEEVFTAPDRNNVNGYVTNKLPLSYNGTIIDGFTLTFKDGQIVDFKAD
KGEDMLRDLINTDEGSKRLGEVALVPDDSPISNRNTIFYNTLFDENASCHLAIGSAYGFN
VEGGTEMTTEEKIASGLNDSNVHVDFMIGSADLTIYGIKQDDTKELVFKNGNWAQ
>ACAKHAOO_01884 hypothetical protein
MENKLIPGILIGAIVGGAATLADKSTRNALVQSVKDVKEGNRSRKPSKFNSIKDEVLYWK
DTIEEIRRNNPELEKSIKDAKDTFVERKNNRIGQ
>ACAKHAOO_01885 hypothetical protein
MSKENKSNSKYLNSIKDEQEKNQDKINVDRTYVEPQEFQSKEPKKDNQVFFVSRLNKPAK
YTKKSNFLSYLIYRIGKDDASGLAAQMTYHFVLAMFPMLIFLLTLLPLFNLDQSQITGLL
SNAPADTSSLIKGVISDVTKNSSGGLLSIGLILAIWSASNGMTAIMNSFNVAYDVEDNRN
GILLKILSVIFTIVLGVVFLVAMALPTMGSVIAHFLFGPLGLDSQVKWIFSLIRVVLPLI
IILILFTILYSVAPNVKTKLKSVLPGAIFTSVIWLLGSFAFGFYISNFGNYSKTYGSIAG
IIILLIWLYLTSFIIIIGAEINAIIHQRHVINGQTPEEAALDHDDNNQNHYNEDTTYEYK
HTATGKDEDYKVDKDPEDKHEEDASLTEKIKDKFTNNDDDNKK

>ACAKHAOO_01886 Response regulator protein VraR
MTIKVLFVDDHEMVRIGISSYLSTQSDIEIVGEGESGKDAIAKAHELKPDLILMDLLMDD
MDGVEATTQIKKDLPHIKVVMLTSFIEDKEVYRALDAGVDSYILKTTSASDIADAVRKTF
NGESVFEPEVLVKMRNRMKKRAELYEMLTEREMEILLLIAKGYSNQEIASASHITIKTVK
THVSNILSKLEVQDRTQAVIYAFQHNLIQ
>ACAKHAOO_01895 UDP-N-acetylmuramoyl-tripeptide--D-alanyl-D-alanine ligase
MRQWTATHLAKLARKASIAAGKKGTDLPGQVARKVDQNILRKLAAQVDDIVFISGTNGKT
TTSNLIGHTLKANHIDIIHNNEGANMAAGITSAFIMQSTKNTKVAIIEIDEGSIPRVLKE
VTPSMMIFTNFFRDQMDRFGEIDIMVNNIAKSISNKGIKLLLNADDPFVSRLKIASDSIV
YYGMKAHAHEFEQSTMNESKYCPNCGKLLVYDYIHYNQIGHYHCSCGFKRELPKYEVSSF
TVSPFLALNINETTFNMKIAGDFNAYNALAAYTVLRELGLNDESIRIGFESYTSDNGRMQ
YFKLDYKEAMINLAKNPAGMNASLSVGEQLVGKKVYVISLNDNAADGRDTSWIYDADFEK
LSEQNIETIIVTGTRAEELQLRLKLAEVNVPIILEKDIYKATAMTMNYEGFTVAIPNYTS
LSPMLEQLNRSFKGGQ
>ACAKHAOO_01896 Bacterial non-heme ferritin
MLSKDLLEALNDQMNHEYFAAHAYMAMAAYCDDASYEGFANFYIQQAKEERFHGKKIYDY
INDRGEHAEFKSIPAPKTEFKSILETFKDGLAQEQDVTRRFYNLSEIAQKDKDYATISFL
NWFLDEQVEEESTFETHIDYLNRIGDDCNTLYLYEKELAARSFDEE
>ACAKHAOO_01902 Aspartyl/glutamyl-tRNA(Asn/Gln) amidotransferase subunit B
MHFETVIGLEVHVELKTDSKMFSPSPAHFGAEPNSNTNVIDLAYPGVLPVVNRRAVDWAM
RASMALNMDIATNSKFDRKNYFYPDNPKAYQISQFDQPIGENGYIDIEVDGETKRIGITR
LHMEEDAGKSTHKDGYSLVDLNRQGTPLIEIVSEPDIRSPKEAYAYLEKLRSIIQYTGVS
DCKMEEGSLRCDANISLRPYGQEEFGTKTELKNLNSFNYVRKGLEYEEKRQEEELLNGGT
IGQETRRFDESTGKTILMRVKEGSDDYRYFPEPDIVPLYVDEEWKECVRQTIPELPDERK
AKYVNDLGLPEYDAHVLTLTKEMSDFFEGAIEKGADVKLTSNWLMGGVNEYLNKNQVELQ
DTKLTPENLAGMIKLIEDGTMSSKIAKKVFPELAENGGDAKQIMEDKGLVQISDEATLTK
FVTEALDNNPQSVEDYKNGKGKAMGFLVGQIMKASKGQANPQKVNQILKQELDKR
>ACAKHAOO_01903 Glutamyl-tRNA(Gln) amidotransferase subunit A
MSIRYESVEKLSEMIKNNEIKPSEVVKDIYDAIEETDPTIKSFLALDKDNAIKKAKELDE
LQAKGQMEGKLFGIPMGIKDNIITKDVETTCASKMLEGFVPIYESTVMNKLHDENAILIG
KLNMDEFAMGGSTETSYFKQTVNPFDHTAVPGGSSGGSAAAVAAGLVPFSLGSDTGGSIR
QPAAYCGVVGMKPTYGRVSRFGLVAFASSLDQIGPITRNVKDNAIVLETISGVDRNDSTS
APVEDVDFTSEIGKDIKGLKVALPKEYLGEGINEDVKEAVKNAVETLKSLGAEVDEVSLP
NTKYGIPSYYVIASSEASANLARFDGIRYGYHSKEAQSLEELYKMSRSEGFGAEVKRRIF
LGTFALSSGYYDAYYKKSQKVRTLIKNDFDKVFENYDVVVGPTAPTTAFNLGDEIDDPLT
MYANDLLTTPVNLAGLPGISVPCGQSNGRPIGLQFIGKPFDEKTLYRVAYQYETQFNLHD
AYEKL
>ACAKHAOO_01908 DNA ligase
MGNIKSRVNELHDLLNQYGYEYYVQDNPSVPDSEYDKLLRELIDIEEAHPEFKSPDSPTV
RVGGEVQSSFEKVNHDTPMLSLGNAFNEQELRRFDQRIREQVGSVEYMCELKIDGLAVSL
KYVEGRFVQGLTRGDGTTGEDITENLRTIHAIPLKINEPLSFEVRGEAYMPRSSFIRLNE
EKEKNEEQPFANPRNAAAGSLRQLDPKLAAKRKLSVFLYSVNDFTDFDATTQSGALDELD
RLGFKTNHERMRVGDIEGVLEYIEKWTKQREQLSYDIDGIVIKVNDIEQQDEMGYTQKSP
RWAIAYKFPAEEVVTTLQDIELSIGRTGVVTPTAILDPVRVAGTTVSRASLHNEDLIHER
DIRIGDSVVVKKAGDIIPEVVKSITERRPEGTLPYSMPTHCPSCDHELVRIEGEVALRCI
NPKCQAQLVEGLIHFVSRQAMNIDGLGTKIIQQLYHHNVINDVADIFYLTEDDLLPLERM
GSKKVENLLKAIEDAKANSLEHLLFGLGIRHLGVKASQVLAEKYETMDRLLEVTEEELIS
IHDIGDKLAQSVVTYLENEDIKALIEKLKYKNVNMVYNGIKTSDIEGHPDFKNKTIVLTG
KLQQLTRKEASAWLELQGAKVTSSVTKKTDLVIAGEDAGSKLTKAEKFGTEIWTEEQFVA
KQNEISS
>ACAKHAOO_01912 Adenylosuccinate lyase
MIDRYSREEMANIWTDQNRYEAWLEVEILACEAWSELGHIPKEDVKKIRQNAKVDVKRAQ

EIEQETRHDVVAFTRQVSETLGDERKWVHYGLTSTDVVDTALSYVIKQANEIIEKDIERF
IKVLEEKAKNYKYTLMMGRTHGVHAEPTTFGVKMALWYTEMKRNLKRFKEVRKEIEVGKM
SGAVGTFANIPPEIEQYVCDHLGIDTASVSTQTLQRDRHAYYIATLALVATSLEKFAVEI
RNLQKTETREVEEAFAKGQKGSSAMPHKRNPIGSENITGISRVIRGYITTAYENVPLWHE
RDISHSSAERIMLPDVTIALDYALNRFTNIVDRLTVFEDNMRNNIDKTFGLIFSQRVLLA
LINKGMVREEAYDRVQPKAMESWETKTPFRQLIEKDESITNVLSKEELDECFNPEHHLNQ
VDTIFKRAGLE
>ACAKHAOO_01921 putative manganese-dependent inorganic pyrophosphatase
MAKTYIFGHQNPDTDAIASAIIMADFEQLTGNSEATPYRLGDINPETKFALDHFEVKAPE
LLSDNLDGQEVILVDHNEFQQSAETISDAEIKHVVDHHRIANFETASPLWYRAEPVGCTA
TILYKMYKERGFEIKPHIAGLMISAIISDSLLFKSPTCTDEDVNAAKDLKDLANVDLDEY
GLEMLKAGASTTDKSAEELLDMDAKSFNMGDYVTRIAQVNTVDIDEVLNRKEDLEKAMLE
TSANEKYDLFVLVVTDIINSDSKILVVGAEKDKVGEAFKVQLDDGMAFLSGVVSRKKQVV
PQITDALIK
>ACAKHAOO_01924 hypothetical protein
MWDLIKGLFKFLLSLVVITSVVVGVGVVAFAYIFKKDFEDIERKTKEIVSDIESNNA
>ACAKHAOO_01929 hypothetical protein
MANLETYFNNSQPLDEYIDSMTVNKDNVLTIYNAFTLPSSDDRINQLKDSSYSKVLVISE
DWCGDAMMNLPILKRISESLQLEVRVFHRDEDTNLIDQYLTNGTARSIPIFIFMNDQFEQ
VSVWGPRAREAQEFVTQLRAEKLPPKDAHNYEDKEKEVHHEITSKYKSDTDLWNQVYDSI
ISKLML
>ACAKHAOO_01938 hypothetical protein
MFENILASIGINSVKVETLVKNKSIHSNDTLEGIVRIEGGSSAQTINKISLTLVERYENP
DKNSQFPVLENELQTFTLHTNVELKEHQTITEEFKFNIYEYEFKSEPKHLILKTHAYVGY
SVDAYDEDKIVFK
>ACAKHAOO_01942 hypothetical protein
MEEKFNNEQEERQFRQFQEYQKQQEEEKKKKRKKSWLFGCGGCLVLLILIIIGVTACTGS
FVNEVDKEINEEGKLDKDKDTKIKSVGETTEIDGVSFTLDSASYTDERNEFAEVQADKVL
KVDMTIKNNSKEEIPVGGDVKVYVDGKQAKSYPITDGLMDSLSPNREISGSEGFAINGNP
EKIELEFQPLTSFSNKRYIYDVKPE
>ACAKHAOO_01949 60 kDa chaperonin
MAKDLKFSEDARQAMLRGVDKLANAVKVTIGPKGRNVVLDKEYVAPLITNDGVTIAKEIE
LEDPYENMGAKLVQEVANKTNEIAGDGTTTATVLAQAMIQEGLKNVTSGANPVGLREGID
KAVRVAVQALHDISQKVENKNEIAQVGAISAADEEIGKYISEAMDKVGNDGVITIEESNG
LDTELEVVEGMQFDRGYQSPYMVTDSDKMIAELERPYILVTDKKISSFQDILPLLEQVVQ
SSRPILIVADEVEGDALTNIVLNRMRGTFTAVAVKAPGFGDRRKAMLEDLAILTGATVIT
DDLGLELKDASIDMLGSANKVEVTKDNTTVVDGDGDDNSIDARVSQIKAQIEETDSDFDR
EKLQERLAKLAGGVAVIKVGAASETELKERKLRIEDALNSTRAAVEEGIVAGGGTALVNI
YNKVDEIEAEGDVATGVNIVLKALSAPVRQIAENAGLEGSVIVERLKHADAGVGFNAATN
EWVNMLEEGIVDPTKVTRSALQHAASVAAMFLTTEAVVATIPEPDNNDNPGMGGMPGMM
>ACAKHAOO_01950 10 kDa chaperonin
MLKPLGNRVIIERKEQEQTTKSGIVLTDSAKEKSNEGIVVAVGTGRVLDNGEKVAPEVKE
GDRVVFQEYAGSEVKRGDKTYLILNVEDLLAIIED
>ACAKHAOO_01974 Ketol-acid reductoisomerase (NADP(+))
MTKVYYDQSVEKDALQGKKIAIIGYGSQGHAHAQNLKDNGYDVIVGIRPGHSFDKAKEDG
FDVYPVAEAVKQADVIMVLLPDEIQGNVYKNEIEPNLEAGNALAFAHGFNIHFEVIKPPK
DVDVFLVAPKGPGHLVRRTFVEGTAVPALFGVQQDATGQARDISLSYAKGIGATRAGVIE
TTFKEETETDLFGEQAVLCGGIHKLIQSGFETLVEAGYQKELAYFEVLHEMKLIVDLMYE
GGMENVRYSISNTAEYGDYVSGPRVITPDVKDNMKAVLKDIQNGNFANSFVKDNENGFKE
FYKLREQQHGHEIEAVGRELRKMMPFIKSKSIQK
>ACAKHAOO_01989 Serine-protein kinase RsbW
MPIKNDYIEMKLPASAEYVSLIRLTLSGVFSRAGASYDDIEDSKIAVSEAVTNAVKHAYK
NDETNGMINLCFELFDDKIKIVISDQGESFDYETTKSQLGPYDENENIDFLREGGLGLFL

IESLMDEVKVFKESGVTISMIKYIKKEQVPNNDERVEIS
>ACAKHAOO_01999 DEAD-box ATP-dependent RNA helicase CshA
MQNFKELGISDKTVETLEAMGFKEPTPIQKDSIPYTLEGKDILGQAQTGTGKTGAFGIPL
IEKVVGQSGVQALILAPTRELAMQVAEQLREFSRGQNVQVVTVFGGMPIDRQIKALKRGP
QIVVGTPGRVIDHLNRRTLKTNGIHTLILDEADEMMNMGFIDDMRFIMDKIPAEQRQTML
FSATMPKAIQTLVQQFMKSPQIVKTMNNEMSDPQIDEYYTIVKELEKFDTFTNFLDVHQP
ELAIVFGRTKRRVDELTSALLSKGYKAEGLHGDITQAKRLEVLKKFKNDQIDILVATDVA
ARGLDISGVSHVYNFDIPQDTESYTHRIGRTGRAGKEGIAVTFVNPIEMDYIRQIEDSNG
RRMNALRPPHRKEVLKAREDDIKDKVKNWMSRESEARLKRISSELLEEYDSTELVASLLQ
ELVEANDEVEVQLTFEKPLARKNRQGKGNGSRRGGKRNNKFDNKNKRSKGNFNKKKGKKP
DRRERQDKGRSTMKGRTFADLQK
>ACAKHAOO_02001 D-alanine--D-alanine ligase
MVKENICIVYGGKSAEHDVSKLTAQNVLNAIDKERYLVDIIYITNDGLWKKKENITEEIK
EIESLNMTDIEAGEITILLKESSNGKPYDAIFPLLHGPNGEDGTIQGLFEVLDLPYVGNG
VLAASSSMDKLVMKQLFEHRGLPQLPYISFLRSEYEKYEGNIIKLVKDKLTYPVFVKPAN
LGSSVGISKCNNEDELKSGIEEAFQFDRKLVIEQGINAREVEVAVLGNDYPETTWPGEVI
KDVAFYDYKSKYKDGKISLQIPAELDEEVQMTLRNMALEAFKATDCSGLVRADFFVTEDN
QIFINETNAMPGFTAFSMYPSLWENMGLSYSDLITKLIDLAKERHEDKKKNKYTID
>ACAKHAOO_02015 putative transglycosylase SceD
MKKTIIASSLAVGLGVVAGNAGHADASEAQVNKAELAQLAQSNDQSLNDSPIQEGAYNVT
FDYEGFTYHFESDGTNWSWNYAQSGQASQQQDVSAQASTVSNQTSAEQVGSQQQSSQAQP
TQTQQAPQTEQTQQPQTEATTSNSSSSNNNASSGSSVNVNSHLQQIAQRESGGDITAINP
SSGAAGKYQFLQSTWDSVAPDEYKGVSPAQAPEDVQDAAAVKLYNTAGASQWVTA
>ACAKHAOO_02019 UDP-N-acetylglucosamine 1-carboxyvinyltransferase 1
MDKIVIKGGNRLTGEVKVEGAKNAVLPVLTASLLASEGQSKLVNVPDLSDVVTINNVLST
LNANVEYNKEEGAVLVDASTTLKEEAPYEYVSKMRASILVMGPLLARLGHAIVALPGGCA
IGARPIEQHIKGFEALGAEIHLENGNIYASTKDGLKGTDIHLDFPSVGATQNIIMAASLA
KGKTVIENVAKEPEIVDLANYINEMGGKVTGAGTDTITIHGVEKLRGVEHSIIPDRIEAG
TLIIAAAITRGDVFVRDAVKEHMTSLIYKLEEMGVNLDFQEDGVRVTAEDELKPVDVKTL
PHPGFPTDMQSQMMALLLTAEGHKVITETVFENRFMHVAEFRRMNANITVEGRSAKIQGK
SQLQGAQVKATDLRAAAALILAGLVAEGTTQVTELKHLDRGYVNFHEKLKSLGANIERVN
Y
>ACAKHAOO_02021 ATP synthase epsilon chain
MSTVNLDIVTPNGSVYEKDDVELVVFQTTAGEMGVMSGHIPTVAALKTGHVKVNFRNGTE
YIAVSGGFVEIRQHKVSVIVQTAETASEIDVERAKLARQRAQSHLEDQDNSDINRAKRAL
ARAENRLRVAELK
>ACAKHAOO_02022 ATP synthase subunit beta
MSNGRVTQVMGPVIDVRFEHNEVPEINNALIIEVPKEDGTFELTLEVALQLGDDVVRTIA
MDSTDGVQRGMEVQNTGKDISVPVGEVTLGRVFNVLGDTIDLEDKLDGSVRRDPIHRQSP
NFDELSTEVEILETGIKVVDLLAPYIKGGKIGLFGGAGVGKTVLIQELINNIAQEHGGIS
VFAGVGERTREGNDLYYEMRDSGVIKKTAMVFGQMNEPPGARMRVALSALTMAEYFRDEQ
GQDVLLFIDNIFRFTQAGSEVSALLGRMPSAVGYQPTLATEMGQLQERITSTNKGSVTSI
QAVFVPADDYTDPAPATAFAHLDATTNLERKLTEMGIYPAVDPLASTSRALEPAIVGQEH
YEVARDVQSTLQKYRELQDIIAILGMDELSEEDKLTVERARRIQFFLSQNFHVAEQFTGQ
KGSYVPVKTTVADFKDILDGKYDHIPEDAFRLVGSMEDVIAKAKDMGVEV
>ACAKHAOO_02023 ATP synthase gamma chain
MASLKEIDGRIKSTKKMKQITKAMNMVSSSKLRRAEKNTKQFEPYMEKMQDAITAIAGAS
KNSSHPMLRPRQVQRSGYLVITSDKGLAGAYSSNVLKRLINDIKEKHTSSDEYSIIVLGQ
SGVDFLKNRGYEIENSLVDVPDQPSFKSIQAIAKHAIDLFSEEHIDELKIYYSHYVSVLE
NKPTTKQVLPLSREDSSQGQGQMSSYEFEPDKESILSVILPQYVESLIYGTILDAKASEH
AARMTAMKNASDNATELIDDLSLQYNRARQAEITQQITEIVGGSAALE
>ACAKHAOO_02024 ATP synthase subunit alpha
MAIKAEEISALLRSQIENYESEMSVTDVGTVLQIGDGIALIHGLNDCMAGELVEFSNGVL
GLAQNLEESNVGVVILGPYTEITEGDEVRRTGRIMEVPVGEELIGRVVNPLGQPIDGQGP

INTTKTRPVEQKATGVMARKSVDEPLQTGIKAIDALVPIGRGQRELIIGDRQTGKTTIGI
DTILNQKGLDTICIYVAIGQKDSTVRANVEKLRQAGALDYTIVVSASASEPSPLLYIAPY
SGVTMGEEFMFNGKHVLIVYDDLTKQAAAYRELSLLLRRPPGREAYPGDVFYLHSRLLER
AAKLNDDLGGGSITALPIIETQAGDISAYVPTNVISITDGQIFLQSDLFFSGVRPAINAG
QSVSRVGGSAQIKAMKKVAGTLRLDLASYRELESFAQFGSDLDEFTARKLERGKRTVEVL
KQDQNKPLPVENQVLIIYALTKGYLDDIPVEDITRFEDELNSWTKSNGSDLLNEIRETGG
LPSDDKFEATINEFKKSFSKSE
>ACAKHAOO_02025 ATP synthase subunit delta
MANVANKYAKALFDVAIDKDRLDLMYDELSEVSEATKNYGEDLRAIDSNPNQPASERRKF
VGIVFGDANYYLKNMLMILANNRHLVLINSIFKEFKSLYNEYHNEDSAIVESVYQLSDEE
LDRIKDLILKQTNLSQVHITTKINPELIGGFRVKVGTTVLDGSVKKDLEQIERKFRRVN
>ACAKHAOO_02026 ATP synthase subunit b
MPVNALTNSFVLGAAGGGVEWGTVIVTVITFAILLALLKKFAWGPLKEVMDKRERDINRD
IDEAEEAKLNAQKLEEENKKTLKQTQDEVQRILEDARVQARKQHEEIIHEANIRANGMIE
TAQSEINSEKERALADINNQVSELSVLIASKVLKKEISEQDQKELVDKYLKEAGDK
>ACAKHAOO_02027 ATP synthase subunit c
MGLIAAAIAIGLSALGAGIGNGLIVSRTVEGVARQPEARGQLMSIMFIGIGLVEALPIIG
VVIAFMTLFQ
>ACAKHAOO_02031 Uracil phosphoribosyltransferase
MSKVHVFDHPLIQHKLSYIRDMNTGTKEFRELVDEVGMLMAYEVTRDLELQDVEIKTPVT
TMTAKRLAGKKLAIVPILRAGLGMTDGVLSLVPAARVGHIGLYRDPNTLKAVEYFAKLPQ
DIDERQIIVVDPMLATGASAIEAINSLKKRGAKNIRFMCLIAAPEGVEKMQEAHDDVDIY
IAALDEKLNDKAYITPGLGDAGDRLFGTK
>ACAKHAOO_02032 Serine hydroxymethyltransferase
MSYIQNQDKAVYEAIQNEYNRQNNNIELIASENFVSEAVMEAQGSVMTNKYAEGYPGRRY
YGGCDYVDVTETIAIERAKALFGAEHVNVQPHSGSQANMAVYLVALEMGDTVLGMNLSHG
GHLTHGSPVNFSGKFYNFVDYGVDKETEKIDYEVVRQLAHEHKPKLIVAGTSAYSRQLDF
KKFKEIADEVGAKLMVDMAHIAGLVAAGLHPNPVEHADFVTTTTHKTLRGPRGGLILCKE
EYKKDIDKTIFPGIQGGPLEHVIAAKAVAFGEALEQDFKVYQEQVIKNAKVLSQTLQEEG
FRIVSGGTDNHLLSVDVKNSVNVTGKEAEATLDSIGITCNKNTIPFDQEKAFVTSGIRLG
TPAATTRGFDEEAFKEVGRIISLALKNPNNDTKLKEARERVSRLTAKYPLYE
>ACAKHAOO_02039 50S ribosomal protein L31 type B
MRQGIHPDYHKVIFLDTTTNFKFLSGSTKTSSETMEWEDGNEYPVIRLDVSSDSHPFYTG
RQKFAAADGRVERFNKKFGLKSNNN
>ACAKHAOO_02040 Transcription termination factor Rho
MPEKVRTSPQYESFHELYKNYTTKELTQKAKTLKLTNYSKLNKSELVLAIMEAQMEKDGN
YYMEGVLDDIQPDGYGFLRTVNYSKGEKDIYISASQIRRFEIKRGDKVTGKVRKPKDNEK
YYGLLQVDFVNDENAEVVKKRPHFQALTPLYPEERIVLETDPQNYSTRIMDLVTPIGLGQ
RGLIVAPPKAGKTSLLKEIANAISKNKPDAKLFILLVGERPEEVTDIERSVESAEVVHST
FDEPPEHHVKVAELLLERAKRLVEIGQDVIILMDSITRLARAYNLVIPPSGRTLSGGLDP
ASLHKPKAFFGAARNIEAGGSLTILATALVETGSRMDDMIYEEFKGTGNMELHLDRKLSE
RRIFPAIDIGRSSTRKEELLISKSELDSLWQLRNMFTDSTDFTERFIRKLKRSKSNKEFF
QQLQKAAEESTKTGKPII
>ACAKHAOO_02041 Putative aldehyde dehydrogenase
MRNYTQQYINGEWIDSDSNETIEVINPATEEVIGKVAKGNSNDVEKAVEAANNVYLEFRH
SSVKERKELLDKIVEEYKNRKQDIIEAITDELGAPLTLSENVHYQMGLNHFEEASRALDS
FEFEERRGDALVTKEAIGVSGLVTPWNFPTNQTSLKLAAAFAAGSPVVLKPSEETPFAAV
ILAEIFDKVGVPKGVFNLVNGDGEGVGNPLSEHPKVRMMSFTGSGRTGSKIMEKASKDFK
KVSLELGGKSPYIVLDDVDVKEAAKATTGKVVNNTGQVCTAGTRILIPESKKEDFLTALK
EEFSKVKVGDPREEGTQVGPIISKKQFDTVQSYIDKGIEEGAELFYGGPGKPEGLNTGYF
ARPTIFINVDNDMTIAQEEIFGPVASVITYNNLDEAIKIANDTKYGLAGYVIGKDKETLQ
KVARSIEAGRIEINEAGNQPDLPFGGYKQSGIGREWGDYGIEEFLEVKSIAGYFS
>ACAKHAOO_02044 Fructose-bisphosphate aldolase
MPLVSMKEMLIDAKENGYAVGQYNLNNLEFTQAILEASQEENAPVILGVSEGAARYMSGF

YTVVKMVEGLIHDLNITVPVAIHLDHGSSFEKCKEAIDAGFTSVMIDASHSPFEENIEIT
SKVVEYAHQHGVSVEAELGTVGGQEDDVVGGIIYADPKECQELVERTGIDTLAPALGSVH
GPYKGEPKLGFKEMEEIGASTGLPLVLHGGTGIPTKDIQKAIPYGTAKINVNTENQIASA
KAVREVLNNDQDVYDPRKYLGPAREAIKETVKGKIREFGTSNRAK
>ACAKHAOO_02048 Spermine/spermidine acetyltransferase
MTIKKQFENITIQEFEEKYRQDVVNFELSERQQIYSSLPKSVLDDALTDENRVANIAINE
YGDVVGFFVLHQYYQHEGYDTPENVVYVRSLSINEKFQGNGYGTTMMMYLPQYVQDLFPD
FNHLYLVVDAENKGAWNVYERAGFMHTATKEEGPIGKERLYYLDLDSKHVSSLRLTENEA
SSISSYSVVDLLKDNQKVGFIALEKTDNRINIVAIEVYKEQRKEGIAESALRQIPTYVRK
HFKQAKTIMITLFGENNELKSLCVNSGFVEIDQSDDLIIFEKYVNY
>ACAKHAOO_02050 hypothetical protein
MSYKNEAYFKDVLINEVFYVANKNKKLVRLNSNNQDYVGVWTQEGQAEDYLKHASIDYDR
VLKIDIDTFVTYELDDLFDEDDQVIINQTSQETGQIVKVVKMTDELMSELDKIRIKEFVK
DVAKEDQVFGLSKHDENHFILISDDSEEKPQIMPVWSLKNRALKVRDEDFEECELIEIEG
SVFSEWLDKLRDNDQVVAIDLKPGVVGTVISAQKLSNELTF
>ACAKHAOO_02054 hypothetical protein
MKVLRYLLGLAFGTAGVLHFTNERSFRNIVPEYLPLRKTAVLVTGVFEIFFGIMLLLKRP
ANWLKTGINLFLLAVLPANIYMARKELPLGDKQVPKWALYSRLPMQFVLIALVKKL
>ACAKHAOO_02055 Phosphopentomutase
MTTPFKRVHLIVMDSVGIGEAPDAAAFNDEGSHTLKHTLEGFNQTLPNLEKLGLGNIEEL
PVVNKVEQPGAFYTKLSEASVGKDTMTGHWEIMGLNIMQPFKVYPNGFPDELIQEIEEMT
GRKVVANKPASGTAIIDELGEHQMKTGDLIVYTSADPVLQIAAHEDIIPLEELYDICEKV
RELTKDPKYLIGRVIARPYVGEPGNFTRTSNRHDYALKPFGKTVMNTLKDNNYDVIAIGK
INDIYDGEGVTEAIRTKNNMDGMDQLINVVKKDFNGISFLNLVDFDALYGHRRDKEGYAQ
AIKDFDERLPELIDNLQEDDLVIITADHGNDPIADGTDHTREYIPVLMFSPKIDKYHELS
GDSTFSSIGATIADNFNVELPEFGKSYLNEMGVEH
>ACAKHAOO_02056 Pyrimidine-nucleoside phosphorylase
MRMVDIIEKKRDGKSLSKEEIEFFIKGYTNGDIPDYQASSLAMAIFFQDMNDEERAALTM
AMVNSGDVIDLSKINGIKVDKHSTGGVGDTTTLVLAPLVAAVGVPVAKMSGRGLGHTGGT
IDKLESIKGFHVEISEEDFIKLVNENQVAVIGQSGNLTPADKKLYALRDVTGTVNSIPLI
ASSIMSKKIAAGADAIVLDVKTGNGAFMKTLEDAEALAHAMVSIGNNVGRNTMAIISDMS
QPLGRAIGNALELKEAIDTLNGKGPEDLTELVLTLGSQMVVLANRANTLEEARQLLNEAI
ENGSALEKFKTFLENQGGDASVVDAPELLPTATYQIEYKAQSSGVVSELIANEIGVASMM
LGAGRQTKEDEIDLSVGIVLNKKVGDVVKEGESLLTIHSNRENVDDVIKKLDESIEIQAQ
ATTPTLIHKIITE
>ACAKHAOO_02057 Deoxyribose-phosphate aldolase 1
MNYAKFIDHTLLKPESTRQQIDQIIDEAKEYNFKSICVNPTHVKYAAERLNDSGVLVCTV
IGFPLGATTTATKIFETEDAIKNGATELDMVINIGALKDGRFEDVQKDIEGVVGAANGKT
VKVIIETVLLSDEEKVKASELAKAAGADFVKTSTGFAGGGATPEDVKLMKDTVGDELEVK
ASGGVRSLEDFNKMIDAGATRIGASAGVQIIQGLESDSDY
>ACAKHAOO_02064 putative sugar epimerase YhfK
MSLLVIGANGGVGSKLVKQLKEDQVDFTAGVRKNEQIETLKQDNIEATLVDVEKDSIEDL
TETFNGYDKVLFTVGSGGSTGADKTIIVDLDGAIKTIEASKQANVKHYIMVSTYDARREA
FDPSGDLKPYTIAKHYADEHLKNSGLNYTIVHPGALEDKEGTSKIETDLYFDGKGSIPRE
DVASVLKEVALSENKFINKEFQVITGNQTISEALSSFE
>ACAKHAOO_02068 hypothetical protein
MFKYHSLALKNAKPQILKTILFSIVSFIVVFAVFAVARTFFMQYFMQMQLAAQLQQSSGP
YLIIMILALLLGLLFFIFAGYQLLAGAINVIAKAISREDVHFNDLFVAFKKGKYGKSLIL
SLISIGLFIVMYLIIMALNYLFNLALTPLFTSLQSAVSSPDNAIAILLTIQIIILIIIGL
ITSIVSWFFFILMINYTVSLVRESNYSAMSHFKDGFRGIRNGHKTWFKFFIAVLLINLII
IIITQPLGTILSVTTGNMSQKVATVILYVAQVIIVVLRLILYFVLMMGAVQYFLKRGEKL
NKTTNEKKKKHKNKDKSVTNKNETLSNQQKIDNTHQPSKNNVEGHSNNVTNNVKQKSENA
KDEVSHNFNKDK
>ACAKHAOO_02073 Glutamine--fructose-6-phosphate aminotransferase

[isomerizing]
MCGIVGYIGYDNAKELLLSGLEKLEYRGYDSAGIAVANDNGTTVFKEKGRIAELRKVADN
NDTDGHVGIGHTRWATHGVPSTVNSHPHQSNNERFTLVHNGVIENYEELKSEYLSDVTFQ
SETDTEVIVQLVEHFSNKGLETEEAFSKVVSLLHGSYALGLLDNQDSDTIYVAKNKSPLL
VGVGEGFNVIASDALAMIKVTNEYKEIHDHEIVIVKKDSVTIKDLDGNVQDRDTYTAQID
ASDAEKGIYDHYMLKEINEQPAVMRRIIQEYEDEKGDLKIDPEIVKDVAAADRIYIIAAG
TSYHAGLVGKEYLEKWAGVPTEVHVASEFVYNMPLLSEKPLFIYISQSGETADSRAVLVE
TNKLGYKSLTVTNVAGSTLSREADHTLLLHAGPEIAVASTKAYTAQIAVLSILSQVVAKA
HGRDNDIDLLRELAKVTTAIETIVDDTPVMEQIAKDFLETTRNAFFIGRTMDYNVSLEGS
LKLKEISYIQAEGFAGGELKHGTIALIEDGTPVIALSTQEKVNLSIRGNVKEVVARGAKP
CIISMEGLEKEGDTYVIPHVHELLTPLVSVVTLQLIAYYAALHRGLDVDKPRNLAKSVTV
E
>ACAKHAOO_02076 Cyclic di-AMP synthase CdaA
MTVVIIIGIIMTMENIFGGDAMDFSNFFDNLSTLKIVTSVLDLLIVWYVLYLLITVFKGT
KAIQLLKGILFIVIGQQVSKILNLTATSKLFDIVIQWGVLALIVIFQPEIRRALEQLGRG
SLFKRYTNTYSHDEEKLIEAVSKAVQYMAKRRIGALIVFEKETGLQDYIETGIAMNSEIS
QELLINVFIPNTPLHDGAMIVQNSKIASAASYLPLSDSAKISKSLGTRHRAAVGISEVSD
AFTIVVSEETGSISVTFDGKLRRDISTEVFEELLAEHWFGTRFQKKGVK
>ACAKHAOO_02087 Iron-sulfur cluster carrier protein
MLTVDQVKEIVGALKDPIIDAPLKETDGIVEVTIKEEIEHVSVKVAMAQLGGQPQLDLQM
AIVKALKDNGANTVGIRFEELPAETVEQYTGKKEEQPKTIEGLLSKDNPVEFIAIASGKG
GVGKSTVAVNLAVALAREGKRVGLVDADIYGFSVPDMMGIDEKPGVQGKEIIPVERHGVK
VISMAFFVEENAPVIWRGPMLGKMLTNFFVEVKWGELDYLLLDLPPGTGDVALDVHSMLP
SSKEIIVTTPHPTAAFVAARAGAMAKHTEHSILGVIENMSYFESKETGNKEYVFGKGGGR
KLADELNTQLLGELPLEQPTWNPKDFSPSIYQADDRLGEIYTSIAQKVIASTIKK
>ACAKHAOO_02092 putative uridylyltransferase
MLDQNQLKKYNQEHLSEYEKLMSSNEKEKLESKVNELDLESIQQLFQDLYVNRQSISDVS
SVSEVKYQRKTELTDQEGAKYEQKGIEAIRNGEFAVLLMAGGQGTRLGYKGPKGSFEIKG
VSLFELQARQLLKLKKETGHLINWYIMTSDINHEETLSYFEQHDYFGYNPDNVHFFKQEN
MVALCETGQLVLNEQGYIMETPNGNGGVFKSLEKNGYLDKMASDGVKFIFLNNIDNVLVK
VLDPLFAGFTVVNDCDVTSKSIQPKDGESVGRLVNQNSKDTVLEYSELDEAVANTFDNAN
IGIHAFKVAFIKQAVNNDLPYHLAVKKLKQLDEDFGVVEKPTLKFELFYFDIFRYATSFV
TLQVNREDEFSPLKNKEGKDSVETATSDLERLNLI
>ACAKHAOO_02095 Heme oxygenase (staphylobilin-producing) 2
MFVVTNRITVKKGFAEKMAPRFTKGGKIEALQGFHKIEVWKVTRDHENEDMYVNTWWETE
KDFEAWTKSDAFKEAHQNRDKTSSESSPVISSEIVKATVLSTLN
>ACAKHAOO_02099 Fe(3+)-citrate-binding protein YfmC
MKGFKFAGIVALLFALVLVTACGNVSNNGSGDSGNKSSSKDSIKIKHELGTTKVPKDAKR
VVALEFSFVDALAALNVKPVGVADDNKPNRIIKPLKEKIGDYKSVGARKQPNLEEISKLK
PDLIIADSNRHKGIYKELSKIAPTIELKSFDGDYNDNIDAFKTIAKALNKEDEGKKRLDE
HKEKIAKYKDEIQFDKNEKVLPAVASKSGLLAHPSESYVGQFLSQLGFKEALNKDVTKGL
SKYLQGPYLQLNAETLKDVNPERMFIMTDGASPKEPSYQEMKKDPVWDTLDAVKHNRVSI
VSRDTWARARGLISSEEMAKELVEISKKDKEK
>ACAKHAOO_02106 Alkaline shock protein 23
MSVDNNNKAKQAYNNQTGVNEKEREEQQKQAEQYREQNEQQQFENKLTFSDEVVEKIAGI
AAREVKGILDMKGGFADNFTNAFSSGNNVTTGVSVEVGEKQAAIDLKVILEYGESAPKIF
RKVTELVKEQVKYITGLQVVEVNMQVDDVMTKKEWQQKNEKNNNNNNNNSERSGLQ
>ACAKHAOO_02108 hypothetical protein
MKRLKNFILGLLIVAIVGFLLFMYIKDSRISKYQDFFLQFNWFQPLLIGLAALLILIGLI
LVFSIFKPTYRKPGLYKDYDDGHIYVSRKAVEKSAYDTLTKYDQVRQPNVVAKLYNKKSK
SYIDIKADFLVPNDVQVKSLTESIRSDIKHNVEYFTELPVRKLEVNVRDQKTAGQRVL
>ACAKHAOO_02122 Lactose phosphotransferase system repressor
MNKYDRLEEITKLVNQKGTVRTNEIVEELNVSDMTVRRDLAELEEKGLLTKIHGGARSNS
AFQIKEKSHQEKHTENIDEKRAVARKAVNLIEENDTIFLGPGTTIELLAQLIERESLTVI

TNCFPVFQILFEKRSLNFKVYLLGGEMRELTESFVGEMTNTLLKTKRFSKMFFSCNGIKD
ADVLTSTIDEAYTQQLALHRSLEKYLLIDTSKIGKEDFTQLCHLEDLTAVVVDKNDEDNV
QKLKAYTEVIH
>ACAKHAOO_02124 30S ribosomal protein S9
MAQVEYRGTGRRKNSVARVRLVPGEGNITVNERDVREYLPFESLILDLNQPFDVTETKGN
YDVLVNVHGGGFTGQAQAIRHGIARALLEADPEYRGSLKRAGLLTRDPRMKERKKPGLKK
ARRSPQFSKR
>ACAKHAOO_02125 50S ribosomal protein L13
MRQTFMANESNIERKWYVIDAEGQTLGRLSSEVASILRGKNKVTYTPHVDTGDYVIIINA
AKIELTGKKESDKIYYRHSNHPGGIKSVTAGELKRNNPERLLETSIKGMLPSSRLGEKQG
KKLFVYGGAEHPHAAQQPENYELRG
>ACAKHAOO_02130 50S ribosomal protein L17
MGYRKLGRTSDQRKAMLRDLATSLIVSERIETTEARAKEVRSVVEKLITLGKKGDLASRR
NAAKTLRNVEILNEDETTQTALQKLFGEIAERYTERQGGYTRILKVGPRRGDGAESVIIE
LVD
>ACAKHAOO_02131 DNA-directed RNA polymerase subunit alpha
MIEIEKPRIETIEISEDAKFGKFVVEPLERGYGTTLGNSLRRILLSSLPGAAVKYIEIEG
VLHEFSAIDNVVEDVSTIIMNIKKLALKIYSEEDKTLEIDVRDEGEVTASDITHDSDVEV
LNPELKIATVSKGGHLKIRLVANKGRGYALAEQNNTSDLPIGVIPVDSLYSPVERVNYTV
ENTRVGQSSDFDKLTLDVWTNGSITPQESVSLAAKILTEHLNIFVGLTDEAQNAEIMIEK
EEDQKEKVLEMSIEELDLSVRSYNCLKRAGINSVQELADKSEADMMKVRNLGRKSLEEVK
YKLEDLGLGLRKED
>ACAKHAOO_02132 30S ribosomal protein S11
MARKQVSRKRRVKKNIENGVAHIRSTFNNTIVTITDEFGNALSWSSAGALGFKGSKKSTP
FAAQMASETASKSAMEHGLKTVEVTVKGPGPGRESAIRALQSAGLEVTAIRDVTPVPHNG
CRPPKRRRV
>ACAKHAOO_02133 30S ribosomal protein S13
MARIAGVDIPREKRIVISLTYVYGIGTSTAKKIVEEANVSADTRVKDLTDDELGRIREVV
DSYKVEGDLRREQNLNIKRLMEISSYRGIRHRRGLPVRGQKTKNNARTRKGPVKTVANKK
K
>ACAKHAOO_02138 50S ribosomal protein L15
MKLHELKAAEGSRRVRNRVGRGAGSGNGKTSGRGQKGQKARSGGGVRPGFEGGQLPLFRR
LPKRGFTNINRKEYAIVNLDQLNKFEDGTEVTPALLVETGVVKNEKSGIKVLGNGSLDKK
LTVKAHKFSASAVEAIDAKGGAHEVI
>ACAKHAOO_02139 50S ribosomal protein L30
MAKLQITLTRSVIGRPETQRKTVEALGLKKTNSSVVVEDNPAIRGQINRVRHLLTVEEK
>ACAKHAOO_02140 30S ribosomal protein S5
MARREEETKEFEERVVTINRVAKVVKGGRRFRFTALVVVGDKNGRVGFGTGKAQEVPEAI
KKAVEAAKKDLVVVPRVEGTTPHTITGRYGSGSVFMKPAAPGTGVIAGGPVRAVLELAGI
TDILSKSLGSNTPINMVRATINGLQNLKNAEDVAKLRGKSVEELYN
>ACAKHAOO_02141 50S ribosomal protein L18
MISKIDKNKVRLKRHARVRTKLSGTAEKPRLNVYRSNKHIYAQIIDDVKGETLVQASSKD
KDIASDSTSKVDLSTKVGEAIAKKASDKGIKEIVFDRGGYLYHGRVKALAEAARESGLEF
>ACAKHAOO_02142 50S ribosomal protein L6
MSRVGKKIIDIPSDVTVTFDGHTATVKGPKGELTRTFNERMTFKQEENTIEVVRPTESKE
DRTVHGTTRALLNNMVQGVSQGFEKTLELVGVGYRAQMQGSDLVLNVGYSHPVEIKAEDG
ITFAVEKNTTVRVSGVSKEQVGAIASNIRSVRPPEPYKGKGIRYQGEYVRRKEGKTGK
>ACAKHAOO_02143 30S ribosomal protein S8
MTMTDPIADMLTRVRNANMVRHEKLELPASNIKKQIAEILKSEGFIKNVEYVEDDKQGVI
RLFLKYGQNNERVITGLKRISKPGLRVYAKANEVPKVLNGLGIALVSTSEGVITDKEARK
RNVGGEIIAYVW
>ACAKHAOO_02145 50S ribosomal protein L5
MNRLKERYNTEVTENLVKKFNYSSVMEVPKIEKIVVNMGVGDAVQNSKVLDNAVEELELI
TGQKPLVTKAKKSVATFRLREGMPIGAKVTLRGERMYEFLDKLIAVSLPRVRDFQGVSKT

AFDGRGNYTLGIKEQLIFPEIDYDKVSKVRGMDIVIVTTANTDEEARELLTNFGMPFRK
>ACAKHAOO_02146 50S ribosomal protein L24
MHIKKGDNVKVIAGKDKGKEGKVIATEPKKDRVVVEGVNVIKKHQKPTQLNPEGGILETE
AAIHVSNVQLLDPKTNEPTRVGYKFVDGKKVRIAKKSGEEIKTNN
>ACAKHAOO_02147 50S ribosomal protein L14
MIQQETRLKVADNSGAREVLTIKVLGGSGRKTANIGDVIVCTVKNATPGGVVKKGDVVKA
VVVRTKSGVRRNDGSYIKFDENACVIIRDDKGPRGTRIFGPVARELREGNFMKIVSLAPE
VL
>ACAKHAOO_02148 30S ribosomal protein S17
MSERNDRKVYVGKVVSDKMDKTITVLVETYKTHKLYGKRVKYSKKYKTHDENNSAKLGDI
VKIQETRPLSASKRFRLVEIVEESVII
>ACAKHAOO_02149 50S ribosomal protein L29
MKAKEIRDLTTSEIEEQIKSSKEELFNLRFQLATGQLEETARIRTVRKTIARLKTVARER
EIEESKANQ
>ACAKHAOO_02151 30S ribosomal protein S3
MGQKINPIGLRVGIIRDWEAKWYAEKDFASLLHEDLRIRKFIDNELKEASVSHVEIERAA
NRINIAIHTGKPGMVIGKGGSEIEKLRNKLNNLTDKKVHINVIEIKKIDIDARLVAENIA
RQLENRASFRRVQKQAISRAMKLGAKGIKTQVSGRLGGADIARAEQYSEGTVPLHTLRAD
IDYAHAEADTTYGKLGVKVWIYRGEVLPTKNTSEGGK
>ACAKHAOO_02152 50S ribosomal protein L22
MEAKAVARTIRIAPRKVRLVLDLIRGKNAGEAIAILKLTNKASSPVIEKVLMSALANAEH
NYDMNTDELVVKEAYANEGPTLKRFRPRAQGRASAINKRTSHITIVVSDGKEEAKEA
>ACAKHAOO_02154 50S ribosomal protein L2
MALKKYKPITNGRRNMTSLDFAEITKSTPEKSLLQPLPKKAGRNNQGKLTVRHHGGGHKR
QYRVIDFKRNKDGITAKVDSIQYDPNRSANIALLVYADGEKRYIIAPKGLQVGQVVESGA
DADIKVGNALPLQNIPVGTVIHNIELKPGKGGQLARSAGASSQVLGKEGKYVLIRLRSGE
VRMILSTCRATIGQVGNLQHELVNVGKAGRSRWKGIRPTVRGSVMNPNDHPHGGGEGRAP
IGRPSPMSPWGKPTLGKKTRRGKKSSDKLIVRGRKKK
>ACAKHAOO_02155 50S ribosomal protein L23
MEARDVLKRPVITEKSSEAMAEDKYTFDVDTRANKTQVKIAVEEIFDVKVANVNIINYKP
KKKRMGRYQGYTNKRRKAIVTLKEGSIDLFN
>ACAKHAOO_02156 50S ribosomal protein L4
MANYDVLKVDGSKSGSVELSDSVFAIEPNNSVLFEAINLQRASLRQGTHAVKNRSAVRGG
GRKPWRQKGTGRARQGTIRAPQWRGGGIVFGPTPRSYAYKMPKKMRRLALRSALSFKVKE
NNFTIVDNFGFEAPKTKEFKNVLTTLEQPKKVLVVTDSEDVNVELSARNIPGVQVSTAQG
LNVLDITSADSVIITESAAKKVEEVLG
>ACAKHAOO_02157 50S ribosomal protein L3
MTKGILGRKIGMTQVFGENGELIPVTVVEASQNVVLQKKTEEVDGYNAIQVGFEDKKAYK
KDAKSNKYANKPAEGHAKKAGAAPKRFIREFRNVNVDEYEVGQEVTVDTFEAGDIIDVTG
TSKGKGFQGAIKRHGQARGPMAHGSHFHRAPGSVGMASDASRVFKGQKMPGRMGGNTVTV
QNLEVVQVDTENNVILVKGNVPGPKKGFVEIQTSIKKGNK
>ACAKHAOO_02158 30S ribosomal protein S10
MAKQKIRIRLKAYDHRVIDQSAEKIVETAKRSGAEVSGPIPLPTEKSVYTIIRAVHKDKD
SREQFEQRTHKRLIDIVNPTPKTVDALMGLNLPSGVDIEIKL
>ACAKHAOO_02164 Glucose 1-dehydrogenase
MFSDLEGKVVIITGAGSGIGKSFAENFGKSKAKVVLNYRSDRHLDEIEEIKHMISNAGGE
AIAVQADVAVEEDVKRLVQSAVKEFGTLDIMINNAGFEKPIPTHKMSVDEWQKMIDINLT
GAFVGSREAVNQFLKEDKKGIIINTSSVHDTIPWPNYVNYAASKGGLKLMMETMSMEYAQ
YGIRINNISPGAIVTEHTKEKFSDPETRAETLEMIPAKEIGEAQDVANVALFLASDLANY
IHGTTIYVDGGMTNYPAFMGGKG
>ACAKHAOO_02168 Swarming motility protein SwrC
MIKKMLTFSLGNKFAIFLMVVLVILGGVYSSAKLKLELLPDAENPVISVQTTMPGATPQT
TQDEISSKIDNQVRSLAYVKSVETQSIQNASIVTVEYNNGTDMDKAEEVLKKEIDKLDFK
DGVSEPELTRNSMDAFPIVAYSFTNKNDDLKTTTKEINDQLIPKLQTVDGVQNAQLNGQT

TRQVTLKFKQNKLDEAGLTADDVENYIKTATRETPLGLFQFGKNEKSLVVDGQFKSVKAL
KDLEIPLTISGQSQSGSSDDQSGSEMSSTGDNASSSSSQSQMSNQSQASNGEMPSVPLSE
LATITVGDERESISKTNGKDAVNVQIMKAQDANTVQVAKEAQNKIDEFVKNNDDIKATKT
MDTAKPIEDSLYTMVEKAALGTIVAIIVILLFLRNIRTTAISIVSIPLSILIALIALKLS
DVSLNILTLGALTIAISRVIDDSIVVVENIYRRLSDPKEELSGNNLVISATREVFKPILS
STIVTIIVFLPLAFVSGSVGEMFRPFALAIAFSLLASLLVSITVVPALASTFFKKGIKLK
RNSQRQNDRGLGVVSKSYRKILNWSLNHKWIVLVISVVILVGSIALGGAKLGTSYISTGD
DKFLAVTYTPKPGETEKSVLDHAKQAQKYLQSKDKVKTVQYSVGGPSPADPTGSTNSMAI
MLEYDKDTPNFEEEPDKVISHLSKMNQPGEWKNQDMGNGSGNDSVEVTVKGPSTNAIKDT
VAKVEQKMKSVNGITNVKSDLSQTYDQYEIKVDQNKATEKGISAGQLALNLNENLPEKTI
TTVKENDNKVDVKVKQNKQTYWSRNKLNNMELKSPTGETVKLKDIAELERTTTPSKLVQE
DGDYATTVSGKITDSDVGGVSSKVMNKINNIDKPDNVKVNVGGATDDINQAMTQLAFAML
VAIVIVYLVLVITFKGGLAPFTILFSLPFTIIGVVLALLITGETISVPSLIGMLMLIGIV
VTNAIVLIDRVITNGHEGMSMKEALLEAGGTRIRPILMTAIATIGALLPLLFGQDSSILI
SKGMAATVIGGLISSTLLTLIVVPVIYEILFTLKGKLTRK
>ACAKHAOO_02169 Lipid II:glycine glycyltransferase
MEKMNITNQEHDAFVKAHPNGDLLQLTKWAETKRLTGWYSKRVAVGEDGEIKGVGQLLFK
KIPKLPFTLCYVSRGFVTDYSDKAALEQLLEETKKVAKAEKAYAIKIDPDVEVDKGIDAL
KNLNALGFKHKGFKEGLSKDYIQPRMTMITPIDKSDEEIFQSFERRNRSKVRLSLKRGTK
VERSNREGLKNFAELMKITGERDGFLTRDLSYFQNIYDSLHEDGDAELFLVKLEPKPVLD
DIDNELKELESEKTQLQNKYERKQVKKTKNKLNDVEAKIQKSIERKDDMTDLLAKHPNGI
YLSGALLMFAGSKSYYLYGASSNDYRDFLPNHHMQYEMMKFAREHGAKTYDFGGTDNNPD
KDSEHYGLWAFKRVWGTYLSEKIGEFDYVLNQPLYQLIEQVKPRLTKAKIKISRKLKGK
>ACAKHAOO_02180 Molybdopterin molybdenumtransferase
MSVEKRNPIPVKEAINRVVQQDIYLNTAEVKLEDSLGYVLAEDIVATYDIPRFNKSPYDG
FAIRSKDSAGAYSENRKQFKVIDHIGAGSVSEKILGQNEAVRIMTGAQIPEGADAVVMFE
QTVEDGDTFTIRKSFEANENVSLKGEETTTGDIVLKKGQVINPGAIAVLATYGYSKVPVT
QRPSIAVIATGSELLDVDDELQPGKIRNSNGPMIKALSEKAGLNVEAYKIQQDDLQSSIS
VVKDAMSKHDIVITTGGVSVGDFDYLPEIYKAVNAEVLFNKVAMRPGSVTTVAVANGQYL
FGLSGNPSACFTGFELFVKPAIQHMMGATAYYPQVVKATLMEDFTKANPFTRFIRASATF
SQSGATVVPSGFNKSGAVVAIAHSNSMIMLPGGTRGFKAGHTVNVILTESNVYETEMTL
>ACAKHAOO_02191 Iron(3+)-hydroxamate-binding protein FhuD
MKKLILPLLVLIIVLAACGNNGGSGKSDSKEETKSYKLDSGKTIKIPKDPKRIAVVAPTF
AGGLHKLGANIVAVNNQVDQSPILKEKFKDTTKIGEADVEKVAKKKPDLIIVYSTDKNIK
KYQKIAPTIVVDYGKHKYLEQQEMLGKIMGKEDEVKKWEDKWKKQTEKDGKEIKDAIGKD
STVSILDEFDKKVYTYGDNWGRGGEVLYQAFGLNMPKGQQDLVKKEGWAEVSQEKIEDVA
GDYIVSTGEGKSKPSYETTNIWKNLPAVKNNNVIEVKAETYWYNDPYTLDFMRKDLKDKL
LKASK
>ACAKHAOO_02197 Staphylococcal secretory antigen SsaA
MKKIATATIATAGVATIAIAGHGHEAHAAEQGYNPNDPTSYSYSYTIDQQGNYHYTWKGN
WSPNQVNHSSQSNYSYNNYSYNYNNYNNYNNYSNNTQSYSANTQQTGGLGASYSTSDRNI
KVTTTTAPSSQSTGVSISRTSSSGSNLYTAGQCTYYVFDRVGGKIGSTWGNANNWASAAA
ASGYTVNNSPSAGAILQTSQGAYGHVAYVESVGSDGSVTVSEMNYGHGAGVVTSRTISAS
QAASYNYIH
>ACAKHAOO_02201 Opine dehydrogenase
MKIAVVGSGNGAVTAAVDMVNQGHDVKLYCRNSSIKKFNNALEKGGFDFNNEGAESFVPF
TEISDDIEYVLKDAEIIQVIIPSSYIEYYAEIMAEHVTSDQLIFFNIAAAMASIRFINVL
EDKHIEVEPKFAEANTLTYGTRVDFENARVDLSLNVRKVFFSTYNKKELSDSFEKVSQIY
PYLVKEENLWKTNLENGNPEVHPGPTLLNVGRIDYADSFALYKEGITKHTVRLLHAIELE
RLTLGRKLGFELLTAKEARIQRGYLERKDEDEPLNRLFNTSPVFSQIPGPNSVQNRYLTE
DIAYGLVLWSSLGRAIDVETPNIDAVIMIASTILERDFFDEGLKVEELGLEKLGLE
>ACAKHAOO_02206 Putative 2-hydroxyacid dehydrogenase
MEKVYIAGAIPEVGLNLLKEHFEVEMYEGEGIIDKATLMEGVKDASALISILSTNVDQEV
IDSASNLKIIANYGAGFNNVDVKYAREKDIDVTNTPKASTASTAELTFGLVLAVARRIVE

GDKLSRTQGFDGWAPLFFRGREVSGKTIGIIGLGEIGSAVAKRAKAFDMDILYTGPHQKK
EKEREIGAKYVDLNTLLENADFITINAAYNPDLHHMIDTEQFKLMKSTAYLINAGRGPIV
NEEALVKALEDKQIEGAALDVYEFEPEITEGLKSLDNVVITPHIGNATYEARDMMSKIVA
NDTIKKLNGETPQFIVNK
>ACAKHAOO_02207 Aurachin C monooxygenase/isomerase
MKIAIIGAGIGGLTAGALLLEKGHDVSIFERQSNISEVGAGIGIGDNVIKKLGKHDLAKG
IKNAGQNLSAMNVLDDKGNILSAVKLKEATLNVTLARQTLIELIQSYVNPQCIYTDHDVI
KVENVEQHTMVHFSNHASQSFDLCIGSDGLHSVVRQAIHQNAKILYQGYTCFRGLVDDAD
LHNIDIASEYWGKRGRVGIVPLINNQAYWFITINASEKDPKYQTFEKPHLQAYFNNYPEP
VRQILDKQSETGIQKHDLYDMKPLKSFVNQRILLLGDAAHATTPNMGQGAGQAMEDAIVL
VNCLAEYDIEKALKRYDKLRVKHTAKVIKRSRKIGKIAQKDNKLVISLRNGVMKRTPNRL
LSGQTKFLYKAKHK
>ACAKHAOO_02231 putative oxidoreductase YghA
MGAQDPRTKFKTSDYEKQEQELPGLQSELTPQPDCGETSYEGHNRLLDYKMLVTGGDSAI
GRAAAIAYAKEGADVAINYLPSEEQDAQEVKAVIEKAGRKAVLIPGDIRDEQFNYDLVEQ
AYKELGGLDNVTLVAGHQQYHDKLEEFDTQSFKETFETNVYPVFWTIQKALDYLQPGASI
TTTSSVQGYNPNPLIHDYAASKAAIISLTKSFSEQLGEQGIRVNCVAPGPFWSPLQITGG
QPQSAIPQFGQDTPLGRAGQPVELSGTYVLLASEESSYTTGQVFGVTGGIQIN
>ACAKHAOO_02236 Lysostaphin resistance protein A
MKTNRISGFQWALTIFVFFVVTMALSLILRDFQASVGIKRFVFDITDLAPFIAAIVCIIA
FKDKRTQLAGLKFSVDIRVIERILLALILPLVIFMIGMFSFNTFADSFILLQATDLSVSV
PTIIIGHILMAFFAEFGFRSYLQNIVENKVNTFFASIIVGLIYSIWAANTTYGMEYAGYH
FLYTFMFSIIIGELIRATKGRTIYIALVFHASMSFAQVFLFSEETGDLFSMKVIALSTTL
VGIVFIILSLIIRFIVYKTTNRSLDEVEPNNYLDHMNDDDTTTSNETKSEDHEHNDKKDS
FTESQLNEDHVELKSQSENQTDSSNEKLKENTSYKEDRRSSVVDDAKDEIDQMKDTSSHK
TEK
>ACAKHAOO_02245 Isopentenyl-diphosphate delta-isomerase
MSDFQREQRKNEHVEIAMAQSDAPQSDFDRVRFVHHSIPNINVDEVDLTSRTTDFDMTYP
IYINAMTGGSEWTKQINAKLAVVARETGLAMAVGSTHAALRNPKMAESFSIARQTNPEGI
IFSNVGADVPVDKAVEAVSLLDAQALQIHVNAPQELVMPEGNREFSTWLDNVAAIVQRVD
VPVIIKEVGFGMSKELYKDLIDVGVTYVDVSGKGGTNFVTIENERRSNKDMDYLANWGQS
TVESLLESSAYQDSLNVFASGGVRTPLDVVKSLALGAKAVGMSRPFLNQVENGGITTTIE
YVESFIEHTKSIMTMLNARDISELKQSKFVFDHKLMSWIEQRGLDIHRG
>ACAKHAOO_02278 putative protein YcnI
MIKKLLATSFILFFTLGFFKVADAHVTLNPKAVDPESYEKVDVRVPVEQKDHTKKVELEV
PKEVQVVNIQPVEGYKYKLDKDKKGNITKIIWTARGKGIGPDEFMDFPIVVASPKDEGKY
AFKAIQTYDNDDKVKWTGKENSEHPAPTLEVKKNANAADVKDEKSKDTASEQTSSGGSIA
LWIVSIIAIILSLVALFKHSRK
>ACAKHAOO_02295 Acid shock protein
MNNREFFEQSIFKNNPKDVFRDLGEQVFNQFSSKSFPTNIYNQTNQYVLEAELPGVNKSE
IELKFEHAALTIKVQKHVSEQTGSVQLSERASGELVRHFEFNDIDKSQIKASYEDGILVV
ILPKEVSAQDQSTTITVE
>ACAKHAOO_02315 Aminoacyltransferase FemA
MKFVTLSPEEFEKFTSSHFSHYTQSRIHFENRNELKGDVHVVGVKDDSDNVIAATLMTEA
RALKVFKYFYTHRGPVMDYSNIELVHFFFKSLTSYLKKHNCLYVLVDPYILENLRNADGE
ILESYDNRAVIKTLEDLGYKHQGWTVGYSTMSQIRWLSVLDLKDKSEDQLLKEMDYQTRR
NIKKTYEMGVKVRTLPMDETDTFFELFQMAEEKHGFKFRDKPYFYEMQKTYKDHAMLKLA
YIDLKDYLSTLQQKHDSLIEQLAEVDAVLEENPNSKKNKNKRTQIQQQVDSNERKLNETK
NKIAEEGETLNLAAALYLYNEHEVYYLSSGSNPKYNAYMGAYRLQWDMIKFAKEHNVDRY
NFYGITGDFSEDAEDYGVQQFKKGFNANVYEYIGDFIKPIHPLAYKVKQLLERK
>ACAKHAOO_02327 hypothetical protein
MKRTDKYKDSYKYDEQYRKRNRQSADGLSRHERRESQEAYNRSNSRYRDDDPRYRNDDRR
YQDDYDYREDYRRRNDEYDEDGNRYYNDRDFRREQQLEEENEKNKKSKKWLIAIIAILLI
IVAVFATRAILNNNSSDNQQTNNASNDNNVSKDYKNEVQNQSDNIKQQVEDAKNDIKDKV

DTDSRIKQIQEDVNNLKNSEQTGEDSKLTKFYQEQVNKLKEANNAQQNNESQSKVNDLLN
DVNTKFDEIKEKLNSILGNSSDTNSQ
>ACAKHAOO_02366 Putative aminopeptidase YsdC
MKESIELLKSLTDVNGIAGYERNVKAKMKEYLEPVSDEIIEDGLGGIFGKKASDNGTKSL
MVAGHLDEIGFIVTKIDNNGFIKFQPIGGWWNQVMLSQKVTITTDDGTEVRGLIGSKPPH
VLDPEERKKPVQIKDMFIDIGVRSKEDAENHGIVVGNMITPYSEFEELANGKYLTAKAFD
NRYGCALAIDVLKRLKDEQIGVDLYAGATVQEEVGLRGAKVAANKIKPDLAIAVDVAVAY
DTPGMNNLGSETTLGNGPVVILMDASNVAHQGLRQHIKEVARRHYITVQWDTTAGGGTDA
GSIHVANEGIPTISIGVALRYMHSNVSVLHTDDYENSVRLVTEIVRSLNDDAYERIKW
>ACAKHAOO_02370 Polyphosphate kinase
MQTQLGENDISLPQYYNNRELSWLDFNYRVLQEAYDKNNPLLEKLNFISIFSSNLDEFFM
VRVAGLKDQVKMGYDKPENKAQMTPQEQVDAIQEKGKKYVDTQYERYNELMTELQDYEIV
MCEPHELSEPLLSKLERDFKLTILPTLTPLGIDAYHPFPKLNNKSLNIFVDIDTEDAINS
AIVQIPSLIPRFLTYNEGAKQYVVMVEDVITYFINYLFTGYEVLNTFTFRITRNADLTIH
EDGAEDLLIEIERFLKERKSGSAVRLEVDGRTENPDDLDWLIETLEVDKQDVYFLNGPLD
LTFIFGLVDHLSHKLNYLTYEKYSPQIPRSLGNNNLYELALKRDIFFHHPYESFEPIVDF
IREAADDPNTIAIKQTLYRVSKDSPIIKSLKEAAEKGKQVTVLVELKARFDEENNVHWAR
MLEDAGCHVIYGMTHLKTHSKIALVVKRIGGELTSFVHLGTGNYNDKTAKLYTDMGIITT
NEQIAEDAINFFNYLSGYSVKPEYNKLIVAPFDIRDVFIDRIDKEISSHLQHGNGKIMMK
MNSLTDKAIIEKLFEASQAGVKIQLIIRGICCLKPGIPGISENIEVVSIVGRLLEHSRIY
YFHNNGDERIYLSSADVMTRNMIKRVEILFPVEDKEIGKRLVDFMDLQLSDNQKGRYQDE
HGHYHYVENNLSPLNSQVYLMQEAIKYGQELKKRSAQPSGMPVVSKRRANWMNRIRRSLK
K
>ACAKHAOO_02412 Putative aldehyde dehydrogenase AldA
MAKVNVRDFIEEQYGLFINGEFQASESGDTLTVTNPANGEDLAKVAKASKSDVDKAVQAA
QDAFDSWSKTSKEERADYLLEISRRIHEKVEHFATIESLQNGKPYRETSTIDVPLTANQF
KYFASVLTTDEGSVNEIDENTMSLVVNEPVGVVGAVVAWNFPILLASWKLAPALAAGNTI
VIQPSSSTPLSLIELAKIFQEVLPKGVVNVLTGKGSESGDAIFNHEGVNKLSFTGSTDVG
YGVAKAGAERIVPTTLELGGKSANIIFDDANLDQVVEGAQLGILFNQGEVCSAGSRLLVQ
SSIYDKVMPKLKEAFENIKVGDPFDEDVKMSAQTGPEQLEKIESYVKIAEEDSNANILTG
GHRLTDNGRDKGYFFEPTIIEIKDNSHQLAQEEIFGPVVVVEKFEDEAEAIKIANDSEYG
LAGGIFTTNINRALNVAKAMRTGRIWINTYNQFPAGAPFGGYKKSGIGREIYKDAIKNYQ
QVKNIFIDTSNQTKGLY
>ACAKHAOO_02415 D-lactate dehydrogenase
MTKIMFYGTRDYEKNDALNWGKANNVEVVTTEEILSEDTVDLAKGFDGVTTMQFGKLADS
VYPKLEEYGIKQIAQRTAGFDMYDLDLAKKHGIIISNVPSYSPETIAEYSVSIALQLVRR
FPAIEKRVQEHNFKWAAPIMSTPVKNMTVAIIGTGRIGAATGKIYAGFGAKVVGYDAYPN
NSLDFIEYKDTVEEAIADADIISLHVPANKESFHLFDADMFSKVKKGAVLVNAARGAVID
TPELIKAVNDGTLYGAAIDTYENEAPYFTYDWTGKEIEDETLLELIKHENILVTPHIAFF
SDEAVRNLVEGGLNAALSVINTGKCETQLN
>ACAKHAOO_02433 Monoterpene epsilon-lactone hydrolase
MKKSMITVATSAILGTLAFIKVKEKRSYKSFITEKYIRMSGMKKTFENEADAKKALEETK
EITAGKYGGTSYEFKHDVRTKSWNGCVTYIVNDQRNHQQKVVLYIHGGAWFQDPLDNHFN
FIDELAGQLDAKVIMPIYPKVPHRDYRTTFELLKLIYDKQAAKVESSEQLIVMGDSAGGQ
IALSFAEYLKAETTLPQPGHIVMLSPVLDGTLSNPDAKTYERIDPMLGIEGSKYFIKLWA
GQLPIEDYRISPINGDLEGLGRLSIFIGTKETLYPDALKLSQMLNDKGIEHDFMPGYNLF
HIYPIFPLPERQKFFTQLKSIIL
>ACAKHAOO_02438 PTS system glucose-specific EIICBA component
MFKKLFGQLQRIGKALMLPVAILPAAGLLLAIGTAFQGEALQQYLPFIKNGVIQNIANMM
TGAGGIIFDNLPIIFALGVAIGLAGGDGVAAIAAFVGFIIMNKTMGAFLNVTPAQLEDPS
KGFANVLGIPTLQTGVFGGIIIGALAAWCYNKFYNISLPSYLGFFAGKRFVPIMMATTSF
ILAFPMAWIWPFIQNGLNAFSTGLLDSNTGLAVFLFGFIKRLLIPFGLHHIFHAPFWFEF
GSWKNAAGEIIRGDQRIFIEQIREGVHLTSGKFMQGEFPVMMFGLPAAALAIYQTAKPEN
KKVVGGLMLSAALTSFLTGITEPLEFSFLFVAPLLFFIHAVLDGLSFLTLYLLNLHLGYT

FSGGFIDFVLLGILPNKTPWWLVIPVGLVYAVIYYVVFRFLIVKFNFKTPGREDKQASVA
NTSASKLPFDVLDAMGGKENIKHLDACITRLRVEVNDKSKVDVEGLKALGASGVLEVGNN
MQAIFGPKSDQIKHDMARIMNGDITKPSETTVTEDTSDEPVQLEEVKETDIYAPGTGHII
PLSEVPDKVFSEKMMGDGIGFVPEKGGIVAPFDGTVKTIFPTKHAIGLESDTGIEVLIHI
GIDTVKLNGEGFESLVDVNEPVTQGQPLMKINLAYLKEHAPSVVTPVIITNQGDKTLTFD
DVDSVDPGKRIMTIK
>ACAKHAOO_02441 Putative thiamine pyrophosphate-containing protein YdaP
MAKIKANEALVKALEAWDIDHLYGIPGDSIDAVVDSLRTVRDSFKFYHVRHEEVASLAAA
SYTKMTGKIGVALSIGGPGIVHLLNGMYDAKMDRVPQLILAGQTNSTLLGTKAFQETNIT
DMVSDVSVYNHQIQKGDNVFEIVNEAIRTAYEKRGVAVVVCPNDLLTQKIKDTTHRAIDT
TKPKAPIPTLRSIKKAAKLIDKSKKPVMIIGVGAQNAKDELREFVEAAKIPVIHTLPAKT
IIPDDHPYSIGNLGKIGTKTSYQTIQDADLLIMAGTNYPYVDYLPKKNIKAIQIDTNPDV
IGHRFSVNVGIVGDAKLAFHQLTDAIKHVPQRPFLEKTLERKAVWDKWMEQDMNQESTPI
RPERLMKSISKYSDDDAVFSIDVGTSTVWSTRYLNLSVNNKFIISSWLGTMGCGLPGAIA
SKIAFPKRQAISISGDGAFQMVMQDFATAVQYDLPMTIFVMNNKQLSFIKYEQQAAGELE
YAIDFSDMDHAKFAEAAGGKGYVLKDPSRIDEVVEAALNENVPTIVDVHVDPNAAPLPGK
IVNEEAINYGKWAYRSITEDKKLDFDEIPPISVAAKRFL
>ACAKHAOO_02447 3-hydroxy-3-methylglutaryl-coenzyme A reductase
MKSLDKTFRHLSREDKLKQLVDYGWLTDESYDVLLKNPLINEEVANSLIENVIGQGTLPV
GLLPKIIVDDKEYVVPMMVEEPSVVAAASYGAKLVNNTGGFKTVKSERLMIGQIVFDDVS
DTDALAQAIYDLEPQIKQIAAEAYPSIIERGGGYRRIEIDTFPENQLLSLKVFVDTKDAM
GANMLNTILEAITAHMKNEFPNRDVLMSILSNHATASVVRVQGEIDIKDLNKGDRSGEEV
AQRMERASVLAQVDIHRAATHNKGVMNGIHAVVLATGNDTRGAEASAHAYASRDGQYRGI
ATWKFDKERGRLVGTIEVPMTLAIVGGGTKVLPIAKASLELLNVQSAQELGQVVAAVGLA
QNFSACRALVSEGIQKGHMSLQYKSLAIVVGAQGDEIARVAEALKTAPKANTATAQQILK
DLRQQ
>ACAKHAOO_02449 Hydroxymethylglutaryl-CoA synthase
MSIGIDKINFYVPKYYVDMAKLAEARQVDPNKFLIGIGQTQMAVSPVSQDIVSMGANAAK
DIITDDDKKHIGMVIVATESAIDNAKAAAVQIHNLLGIQPFARCFEMKEACYAATPAIQL
AKDYIEKRPNEKVLVIASDTARYGIQSGGEPTQGAGAVAMLISNNPSILELNDDAVAYTE
DVYDFWRPTGHKYPLVAGALSKDAYIKSFQESWNEYARREDKTLSDFESLCFHVPFTKMG
KKALDSIINDADETTQERLTSGYEDAVYYNRYVGNIYTGSLYLSLISLLENRSLKGGQTI
GLFSYGSGSVGEFFSATLVEGYEKQLDIEGHKALLNERQEVSVEDYESFFKRFDDLEFDH
ATEQTDDDKSIYYLENIQDDIRQYHIPK
>ACAKHAOO_02457 1-pyrroline-5-carboxylate dehydrogenase
MVVPFKNEPGIDFLVQENVERFQKTLEQVKNELGQTLPIVIDGEHITKDDTFDSINPANT
SELIAKVSKATKEDVDKAFESSNKAYKAWRQWSHKDRAELLLRVAAIIRRRKEEISAVMV
YEAGKPWDEAVGDAAEGIDFIEYYARSMMELADGKPVLDREGEHNKYFYKSIGTGVTIPP
WNFPFAIMAGTTLAPVVAGNTVLLKPAEDTVLTAYKLIEILEEAGLPKGVVNFVPGDPKE
IGDYLVDSVHTHFVTFTGSRATGTRIYERSAVVQEGQTFLKRVIAEMGGKDAIVVDENID
TDLAAESIVTSAFGFSGQKCSACSRAIVHSSVYDEVLEKAVALTKELTVGNTVDNTFMGP
VINKKQFDKIKKYIEIGRKEGKIEIGGEADDSTGYFIKPTIISGLKSSDQVMQEEIFGPV
VGFTKFDNFEEAIEIANDTDYGLTGAVITNNRENWIKAVNEFDVGNLYLNRGCTAAVVGY
HPFGGFKMSGTDAKTGSPDYLLNFLEQKVVSEMF
>ACAKHAOO_02459 General stress protein 39
MNLLEFHKQIKGYTQNRQPGTERDMDPRPISELDEYKAAGKLQGKVALITGGDSGIGRSV
AILFAKEGANVAIGYYDEHEDAEETVNRLKEIGVKAKAYAHDLKDAQQSKDLVQQVVQEF
GGLNILVNNGAVQYPQDSFMDITPEQFKDTFETNIFGMFFLSQAAVPYLSDNDTIINTTS
VTAYRGSGHLIDYASTKGAIVSFTRSLATTLMEKNIRVNGVAPGPIYTPLIPATFDEDKV
ENQGGNTPMGRRGQPAELAPAYVFLATNADSSYITGQIIHVNGGDYITT
>ACAKHAOO_02462 Copper chaperone CopZ
MINKVINVEGMSCDHCRNAVESALAKLNGVTSAEVDLDKNQVRVDYDENRVSVEQMKEAI
EDQGYDVK

>ACAKHAOO_02463 D-lactate dehydrogenase
MTKIKIMSVRDEDIPYIEEWAEQNNVEVELNKEILTEDNVDDVQGFDGLSLSQQHPISED
VFAKLQQFGIKHIAQRSAGFDTYDLNLATQYGIIISNVPSYSPSSIAEFAVTQAINIVRN
QNDIQRKLKDYDFRWEPSILSRSISDLTVAVIGTGRIGSIAASIFAKGYRSHVVAYDPFP
NEKVAQYVEYKDTLEEALRDADIVTVHIPATKYNPHLFNRDLFSKFKKGAVFVNAARGSI
VDTRALLDAIDSGHIKGAALDTYEHERGLFPGDYRNKAINDDLLDELIAREDIWLTPHIA
FYTDAAVKNLIVDALDAVIDVMNTGTTKLRVN
>ACAKHAOO_02469 putative transglycosylase IsaA
MKKTVIASSLAVALGVTGYALTTDNSAHASESTTNYAQLANLAQNNPSELNAHPVQAGAY
NITFVKDGFKYNFTSDGQSWSWNYTYVGGADTVATTQAAPAAQSTDYSASYSNEASTQSV
SSNQQSSNTNVEAVSAPKTTSYSASTSSSSSASTGGSVKAQFLANGGTEAAWNAIVMPES
GGNPNAVNPAGYRGLGQTMESWGTGSVASQTKGMINYANNRYGSLDAAIAFRANHGWW
>ACAKHAOO_02482 Dihydroorotate dehydrogenase (quinone)
MYKLIKPLLFKFDPEKAHGMTIDALKLGQKRPFLLPIMHKIFNYEDPSLTQTIKGITFNN
PIGLAAGFDKSCEVPKALENVGFGALELGGITPKPQDGNPKPRMYRLVEDNALINRMGFN
NIGMNRALWNLRRHSYNIPVGLNVGVNKTTPYNERYQDYIKVIDTFKADVSFFTVNISSP
NTENLQSFHDKDEFSMLCKALKDFKANTEVNVPIFLKLTSDLELDGFKNILPAITETFDG
MILANTTRKRDGLTSSNKVEDGGLSGQPLFKRNLELVKWAYQQTNGKFLIIGTGGIFSAK
DAIQMMRNGASLVQIYSSLVIEGPGLTKKINKDIAAYLKQHNYNNVSDIIGLDAK
>ACAKHAOO_02502 Fructose-bisphosphate aldolase class 1
MNKEQLEKMTNGKGFIAALDQSGGSTPKALKEYGVNEDEYSNDDEMFQLVHDMRTRVVTS
PSFSPDKILGAILFEQTMDREVEGKYTGDYLADKGVVPFLKVDKGLAEQQNGVQLMKPID
DLDDTLDRAVERHIFGTKMRSNILELNKQGIKDVVEQQFEFAKKIIAKGLVPIIEPEVNI
NAKDKAEIEEVLKAELKKGLDALNDDQLVMLKLTIPTKANLYKELADHPNVVRVVVLSGG
YSRDEANKLLKDNDELIASFSRALASDLRASQSQEEFDKALGDAVDSIYDASVNKN
>ACAKHAOO_02508 putative malate:quinone oxidoreductase 2
MAESKDVILIGAGVLSTTFGSMLNELEPNWNIKLYERMDRPGLESSNERHNAGTGHAALC
ELNYTVQQPDGSIDIDKAKEINEEFEISKQFWGHLVKAGHIENPREFINPLPHISFVRGV
NNKEFLKKRYEAMKQSPMFDNIEYTEDIEVMRKWIPLMMQGRVDDGKMAASKIDEGTDVN
FGELTRKMARHLELDEHVEVKYSHQVLDFERLSNGKWQVKIKDLKTGSVFEEVTDYVFIG
AGGAAIPLLQKTGIPESKHLGGFPITGQFLACTNPQVIEAHDAKVYGKEPPGTPPMTVPH
LDARYIEGKRTLLFGPFANVGPKFLKNGSNLDLFKSIKPYNITTMLAAAVKNLPLIKYSF
DQIIMTKEGCMNHLRTFYPEARDEDWELYTAGKRVQVIKDTEKEGKGFIQFGTEVVNSED
HSVIALLGESPGASTSVSVALEVLEKNFPEQIGQWNAKIKEMIPSYGQSLIEDVELMRKI
RRQTSKDLELGYYEDAK
>ACAKHAOO_02515 Acetyl-coenzyme A synthetase
MDNSSLLAPETYNIVSEIEKHASNTSKKALIYENGVDEPITVTYSELIKNANKVGHVLLN
HGLKKGDKVLIMMPRAIMTYELYLAALKLGIAIIPSSEMLRTKDLQYRITHGEIKAVIAK
SDFIEEFKGVKEYDSLTKFIIDGHETDWINIEDEKASQSDALDIEKTSRDDLAILSYTSG
TTGNPKAVTHSHGWGFAHMKMAPEHWLCIKEDDLVWATAAPGWQKWVWSPFLSIMGSGAT
AFVYNGKFNPSRYLELLQGFEINVLCCTPTEYRMMAKLDNLQEYNLEHLHSAVSAGEPLN
REVVEQFRNNFNLTVRDGYGQTESTLLIGFLKDTKSRPGSMGKEIPGSRVTIVDDEGQPV
ETNVKGNIALPLDFPGLFKGYYKDEERTKAAQAGDYYITGDLAHIDEDGYFWFEGRRDDI
IISSGYTIGPFEVEDALTNHPAVKECAVVASPHEIRGNIVKAFVILQDNYQGNDDLVKEL
QTFAKNEVAPYKYPRAIEFVDSLPKTNSGKIRRVELRDAEREKYNKENGFK
>ACAKHAOO_02522 Oxygen-dependent choline dehydrogenase
MSKKNSYDYVIIGGGSAGSVLGNRLTEDKDKEVLVLEAGRSDYPWDLFIQMPAALMFPSG
NRFYDWIYQTEEEPHMGRKVDHARGKVLGGSSSINGMIYQRGNPMDYEGWAEPEGMESWD
FAHCLPYFKRLEKTYGATPFDQFRGHHGPIKLKRGPATNPLFKSFFDAGVEAGYHKTKDV
NGYRQEGFGPFDSQVHNGRRVSASRAYLHPAMKRKNLTVKTRAFVTKIHFDGNKATGVTF
KRNGRYHTVDAGEVILSGGAFNTPQLLQLSGIGDAEFLKSKGIEPRMHLPGVGENFEDHL
EVYIQHECKEPVSLQPSLDVKRMPWIGLQWIFARKGAAASNHFEGGAFVRSNNQVAYPNL
MFHFLPIAVRYDGQKAPVAHGYQVHVGPMYSNSRGSLKIKSKDPFEKPSIVFNYLSTKED
EQEWVEAIRVARNILAQKAMDPYNGGEISPGPSVQTDEEILDWVRRDGETALHPSCSAKM

GPASDPMSVVDPLTMKVHGMENLRVVDASAMPRTTNGNIHAPVLMLAEKAADIIRGKKPL
EPQYVDYYKHGVSDENAGAMEFDPYYQH
>ACAKHAOO_02549 Polyisoprenyl-teichoic acid--peptidoglycan teichoic acid transferase TagU
MKKKKSTIFKVLLTIIIIVVILAIIAGVYLFSKIHSLSDSITNPLDRDKSELRDKPAKEG
DPMTVVLYGIDDDAQREQQGMGQRSDSIVLMSINPKDKKTVMVSVPRDTRAKIVGKGTTE
KINHAYAYGGPKMAVNSLEKLMDVPVDHYISIDMDGVKTVVDELGGVTITSNGSFITKDS
TNTYQFTKGEQYKMDGKEALAYMRSRKEDGAGGDEGRQLRQQQVITAVAREAFSVNSVTK
LNGIFKAAQDNLKTDLSFIQLNRFKSDYDKAQDNVERLTINGQNALGDDNLYYFYPDKNS
LKEVKEKLKENLNLN
>ACAKHAOO_02551 UDP-N-acetyl-D-mannosamine dehydrogenase
MKLTVVGLGYIGLPTSIMFAKHGVDVLGVDINQKTIDSLQSGKVNIEEPGLQEVFEEVLE
AGKLKVSTQPAEADAFIISVPTPNNDDEYESCDISIVLSAVNSVLPHLKKGDTIIVESTI
APRTMDDHVKPLIEEKGFTIGEDVYLVHCPERVLPGKILEELVYNNRIIGGVTPNCVEAG
KRVYSTFVQGEMIETNARTAEMSKLMENTYRDLNIALANEITKISNNLDVNVLDVIEMAN
KHPRVNIHSPGPGVGGHCLAVDPYFIIAKDPEHSPLIQTGRKVNRSMPEYVVENVKRILS
DVEDAKVSVFGLTYKGDVDDIRESPAFDIYKLLQEESLEVTAYDPHVELDFVEKDIKNAT
ENASLVLILSDHSEFKSFKDSDFVNMKNKIIFDTKNVVKSSFDEVSYFNYGNLYETRNIK
LNK
>ACAKHAOO_02557 hypothetical protein
MKILNIVSSNIVQDPRILKQMETIKSLTNQHLMVGMNNNKVTKDRLSKLDFNYMLFGKKN
DTKTIIGKLLKRIIYAKNVISTIKSYNPDIIHANDFDVLFMVYLSGKKNANIIFDAHEIY
AKNAFINRFSFISSIVEKIEKYIIAKRVNSFITVSHAAKSYYEQRGYKKTPYVITNAPIG
DEERKFEKKEKINEVVYQGQIVANRGYEEFVKASTEQFKESPMYIVRGFGPLEEEIRQLI
KETNANAKLDNPVEIRELVDKLTESEVGVVLTKPISINFEYTVSNKIFECIHAGLPVILS
PVKEHYYLNEKYDFGIVIDEVTPKKIAEAVQSLIDNKNLYDRLRKNAIEASKILNWQNES
KKLKKIYLNN
>ACAKHAOO_02583 Arginine deiminase
MTQGPIQVNSEIGRLKTVLLKRPGKELENLVPDHLSGLLFDDIPYLKVAQEEHDKFAQTL
RDEGVEVVYLEKLAAEAIADKDVREQFIDDILAESQKTVLGHEAEIKTFFAKLSDQELID
KIMAGVRKEEIELKTTHLVEYMDDRYPFYLDPMPNLYFTRDPQASVGRGMTINRMYWRAR
RRESLFMTYILKYHPRFKDADVPVWLDRNSPFNIEGGDELILSKEALAIGISERTSAQAI
ERLARNIFKDESTTFKKVIAIEIPNSRTFMHLDTVFTMIDYDKFTVHSAIFKEENNMNLF
TIEYDEAKDDIKITHSNKLRETLADVLGVEKIEFIPTGNGDVIDGAREQWNDGSNTLCIR
PGVVVTYDRNYVSNQLLRDKGIKVLEITGSELVRGRGGPRCMSQPLFREDI
>ACAKHAOO_02587 Immunodominant staphylococcal antigen B
MNKVVKTIASTTLAFGTLLGVSSAVLPVQDTAHAATQTKGYYYSYNGYVDKDAKFLTDKN
FINAIKHDNIKFNGIKLAKTNSTTVKEKYNQKFTGVTSNGKKANKLQFIVKGDLTYSQLK
KAYGKDLKKVKGNNNVKGSGIYVYKPNKDGLATSFVLNNNKVVEVDISYVGFTTSK
>ACAKHAOO_02589 ESX secretion system protein YueB
MKNALKLFITDLKRVAKTPAVWVILTGLAILPSFYAWFNLWAMWDPYSHTGHIKVAVVNE
DKGDKVRGKKINVGNTLEDNLKKNDKFDWQFVSREKADHEIKMGKYYAGIYIPKEFSHQI
TGTLRKKPQKADIEYKVNQKINAVAPKMTDTGSTVIVDEANKQFNETVTKALLQEANKVG
IQLEDEVPTINKIKNAVYAAHDSLPQINKIADRIEYLNDHQDDLDKYANQFRALGNYKGD
ILDAQQKLNDVNAAIPSLNEKAKLILALNEYMPNIEKLLDVASNDIPAQFPKINRGVDIA
SEGLDLANTRLNDAQGYLTSAQQRVGDYQEAAGRAQEVNNQANSALRQQSTSGLPQYQIQ
KLSTDSSQDTVNDNQIVSNNDVKSMNSALAEALLTLSSNSDNQAKATQSDIKALKDISYG
VIGSNRPTEFNDMLRNLKTRLENSSKSNQQLIDVLKELEKREHVDLSSQIKQVESANNRI
SDSLRSTNQLIDALKNGSSGKAEAVNVLRSLPDLNKALGNYRDFIKNDLNNRLLVVSNQI
TQELSKGQNTLSDVQSKLNTINRVIIAGQDIVTDGQNRIANIQSELPALEQTYINAMQTA
QKYFPTVKKDVANAADFVRNDLPDLEQQLANATATVNTNLPTLFNKYDNAVDLLNQNQPR
AKEALANVANFSENRLPDIEKDLNKANKIFKKLDEDDAVDKVIDALKNDLKKQADVVANP
IHKKQVDVFPVKDYGSGMTPFYTSLSIWVGALLLVSLLSVDNKHEALEDELTKRQIYLGK
GAFFIMMGIIQALIVTIGDLVILKAQVESPTLFILVALCGAIVFNTIVYTCVSLLGNPGK

AIAIILLVLQIAGGGGTFPVVTAPKFFQTISPYLPFTYVIDSLRETVGGIVPEILITKMI
ILILFGLGFFILGILVKPVLDPIMRKISKRVAESKVTE
>ACAKHAOO_02592 hypothetical protein
MTHTNEVTNSTSKGRKKRFSLKMPGAFTILFILTVIAVMATWMIPAGAYSKLSYDGGAHE
FKIVDAHNKSKTVPGTQEQLDKLGVKIDVDQFKSGAINKPISIPGTYERLEQKPAGPDQI
TSSMVNGTIEAVDVMVFILVLGGLIGVVQKSGSFESGLLALTKKTKGHEFLLVFMVSVLM
VLGGTLCGIEEEAVAFYPILVPIFIAMGYDSIICVGAIFLASSVGSTFSTVNPFSVVIAS
NAAGTTFVDGLYWRIVGLIVATIFTVSYLYWYAKKIKKDPKASYTYEDKTKFENQWSVIN
DDGGEKPFTLRKKIILILFILPFPIMVWGVMTQGWWFPIMASMFLAFTIVIMLMVALGKD
GLGEKVVVDAFVEGASSLVGVSLIIGLARGINMIMNDGLISDTILNFSSSLVQGVSGPIF
IVILLIIFFFLGFIIPSSSGLAVLAMPIFAPLADTVGIPRFVIVTAYQFGQYAMLFLAPT
GLIMATLQMLDMKYSHWLKFVWPVVAFVFIFGGGMLITQVLIYS
>ACAKHAOO_02593 hypothetical protein
MKKLRQHSKFNSYLKVSCSVLLMSGTLVGYGFTKDGFAQSNDRIDNVSSEMTSVQNKLDK
AIDKAKAKIDRLKYLQATDIKSYKEDIEDARNQSEIDQILRDAQEEDRISNEESTKETGE
KASTSNESSLSTAKQSSTNEENKLDELDKVIADLDSLSEKVDTHQQNGDMKSEQDSTNNN
ENSQISSGQSASSQNNKNQINDKNDDTSILDEMDNVKNDIESTKESAHSSVEDIRDQTDS
SIQDDNSTESQSKNNTNTASDKSILSGIKQIDKDEDSHKSNKIDSKEGHVDALTDELSAN
QKIDQAITKVENQQDNTSKRYSDHKLKQLRQLEQQVKQNSSLTNEQKKNVEKNIRNVRQN
VKANRDEISGRLEQSSNKQATVEQILGSVFSKNEAQKIAKQIKTNGQSDKQITDQMMKHI
DNLKTTTSDDILASMFDQAPDKEALIKTLLSTRLGNNEASQIAKQLAKENLSSSELVNQV
KQKINANQKITADDILKDVLDKSSDPKQTIETLLATKLNQTQAKALADLIARAQTDKADA
LDLVKNALNGTAGDLLQLQNKLDTAKNNLSYILDPITNRPSLFDRINGNASSSTPLNQGS
HLLDGLTGSSLLDGLNSGGSLLDNIEDIPNPVKGLSLGQLGDDDGFLSGLFDDEGNLSLP
NTGEVVKKSWLPVTVLLVIAGGTLIGLGRRKQQNTKH
>ACAKHAOO_02597 Membrane protein YdfJ
MAKLLYKLGKFIAKNKWLSVIGWLVILGVIITPLMINSPKFDSDITMNGLKSLDTNDKIS
KEFHQDSEKASMKIVFHSNKNDGLNNKDTKKDIEDALDNIRQNDDYIQNISNPYDSGQVN
DEGDTAIANVSYVVPQTGLKDSSKHIIDKELKDITDNHNVQIEKTQGGAMNAEPGGTSEI
VGIVVAFVILLITFGSLIAAGMPIISAIIGLGSSVGIIALLTYIFDIPNFTLTLAVMIGL
AVGIDYSLFILFRFKELKKKGVDTVEAIATAVGTAGSAVIFAGLTVMIAVCGLSLVGIDF
LAVMGFASAISVLFAVLAALTLLPALISIFHKSIKIKDKPSKSKDPKDHPWAKFIVGKPV
IAVIVSLIILILAAIPVSGMRLGIPDDSLKPTNSSEYKAYKLISDNFGEGYNGQIVMLVN
TKDGGSKSTIERDLNNMRSDLEDIDNVDTVSKAQLTDNNNYALFTIIPEKGPNSQSTENL
VYDLRDYHSQAQEKYDYGTEISGQSVINIDMSEKLNNAIPVFAGVIVVLAFFLLMIVFRS
ILVPLKAVLGFILSLMATLGFTTLVIQHGFMGSLFGIENTGPLLAFLPVITIGLLFGLAI
DYELFLMTRVHEEYSKTGDNDHSIRVGIKESGPVIVAAALIMFSVFIAFVFQDDSGIKSM
GIALGFGVLFDAFVVRMTLIPALTKLFGKASWYLPKWLGAVLPNVDVEGKALEEDNHHDT
SSENGHANDRNNDYTRQDKDNHSYQNDNRKHNRNYNDEDYNRSVHLDNHHDQHHRQHHYD
NQRDDIDYESLYTQNGDHTHYDERNYNDRHYQDNYDRNNDYRHSNHVQHNDNHDDYHDSN
FDKTTNLYKELTDSNIDQDVLFKALMLYARENNKGVYDRYNRSSQHRHDDELRD
>ACAKHAOO_02598 Glycine betaine/carnitine transport binding protein GbuC
MLKRRSSKFLGLLATLALAIVLSACGNSGSGSGSSDDSQTSLGNKEIEIPYIASDNSTPR
SLVIAEVLKKAGYDVTTTPVTASGPLYAAVSENSDSFHASGIFPTTDKSYYDKFKSKLTI
YDDKHLIDNAKVGLAVPKYVQDVDSISDLKNSAFGKSVDWTIQGTDARNGVMQETKDELD
SDNLDKYSLKESSDQDQFKKIQSAFKQQEPIVFTAMEPSWFSKELDVKMLKDPDNIYGNN
NQHIDLVFNKDFKEAHPAAYTIATRMADDWSKSDEDKLAKKIFVDNKNPEQVAKDYVDDN
DNKVDEWLEGIETN
