# Supplementary material for: Identification of surface proteins in a clinical Staphylococcus haemolyticus isolate by bacterial surface shaving
Source: BMC Microbiol. 2020 Apr 7;20:80. doi: 10.1186/s12866-020-01778-8 (PMC7137321; doi:10.1186/s12866-020-01778-8)
Supplement: Supplementary file 3 — Additional file 3: Table S3. Workflow for bacterial protein surface shaving samples. X = performed, − = not performed [file 12866_2020_1778_MOESM3_ESM.docx]

*Supplementary table 1 -* *Overview of workflow for bacterial protein surface shaving samples. X = performed, - = not performed*

| Workflow in chronological order | HaCaT colonization | Control group |
| --- | --- | --- |
| Primary TSB culture | **X** | **X** |
| Subculture in DMEM with 10% FBS | **X** | **X** |
| Wash twice with DPBS | **X** | **X** |
| Resuspended in DMEM with 10% FBS | **X** | **-** |
| Incubate *S. haemolyticus* with HaCaT cells in tissue culture plates | **X** | **-** |
| Wash four times with DPBS | **X** | **-** |
| Resuspended in DPBS | **X** | **X** |
| FACS | **X** | **X** |
| Ultra centrifugation after FACS | **X** | **X** |
| LPI^TM^ Flow Cell | **X** | **X** |
