# Supplementary material for: Identification of surface proteins in a clinical Staphylococcus haemolyticus isolate by bacterial surface shaving
Source: BMC Microbiol. 2020 Apr 7;20:80. doi: 10.1186/s12866-020-01778-8 (PMC7137321; doi:10.1186/s12866-020-01778-8)
Supplement: Supplementary file 4 — Additional file 4: Table S4. Manufacturer’s and modified gradient using the Pierce High pH Reversed-Phase Peptide Fractionation Kit. [file 12866_2020_1778_MOESM4_ESM.docx]

*Supplementary table 2 – Manufacturer’s and modified gradient using the Pierce High pH Reversed-Phase Peptide Fractionation Kit.*

| **Protocol according to manufacturer**  Elution enough for 1 sample = 300 µl   \| Fraction No. \| Acetonitrile (%) \| Acetonitrile (µL) \| Triethylamine (0.1%) (µL) \| \| --- \| --- \| --- \| --- \| \| 1 \| 10.0% \| 100 \| 900 \| \| 2 \| 12.5% \| 125 \| 875 \| \| 3 \| 15.0% \| 150 \| 850 \| \| 4 \| 17.5% \| 175 \| 825 \| \| 5 \| 20.0% \| 200 \| 800 \| \| 6 \| 22.5% \| 225 \| 775 \| \| 7 \| 25.0% \| 250 \| 750 \| \| 8 \| 50.0% \| 500 \| 500 \|   **Modified protocol**  TMT gradient enough for 3 samples = 1000 µl, wash 3% | | | |
| --- | --- | --- | --- | --- | --- | --- | --- | --- | --- | --- | --- | --- | --- | --- | --- | --- | --- | --- | --- | --- | --- | --- | --- | --- | --- | --- | --- | --- | --- | --- | --- | --- | --- | --- | --- | --- | --- | --- | --- |
| Fraction No. | Acetonitrile (%) | Acetonitrile (µL) | Triethylamine (0.1%) (µL) |
| 1 | 7.0% | 70 | 930 |
| 2 | 9.0% | 90 | 910 |
| 3 | 10.0% | 100 | 900 |
| 4 | 11.0% | 110 | 890 |
| 5 | 12.0% | 120 | 880 |
| 6 | 14.0% | 140 | 860 |
| 7 | 16.0% | 160 | 840 |
| 8 | 18.0% | 180 | 820 |
| 9 | 20.0% | 200 | 800 |
| 10 | 22.0% | 220 | 780 |
| 11 | 25.0% | 250 | 750 |
| 12 | 50.0% | 500 | 500 |
| **13=extra** | 75.0% | 750 | 250 |
